# Thermally and Base-Triggered “Debond-on-Demand” Chain-Extended Polyurethane Adhesives

**DOI:** 10.1021/acs.macromol.4c02775

**Published:** 2025-01-02

**Authors:** Matthew J. Hyder, Jessica Godleman, Ann M. Chippindale, James E. Hallett, Thomas Zinn, Josephine L. Harries, Wayne Hayes

**Affiliations:** † Department of Chemistry, University of Reading, Whiteknights, Reading RG6 6AD, U.K.; ‡ Domino UK Ltd., Trafalgar Way, Bar Hill, Cambridge CB23 8TU, U.K.; § Diamond Light Source, Diamond Light Source Ltd., Harwell Science & Innovation Campus, Didcot OX11 0DE, U.K.

## Abstract

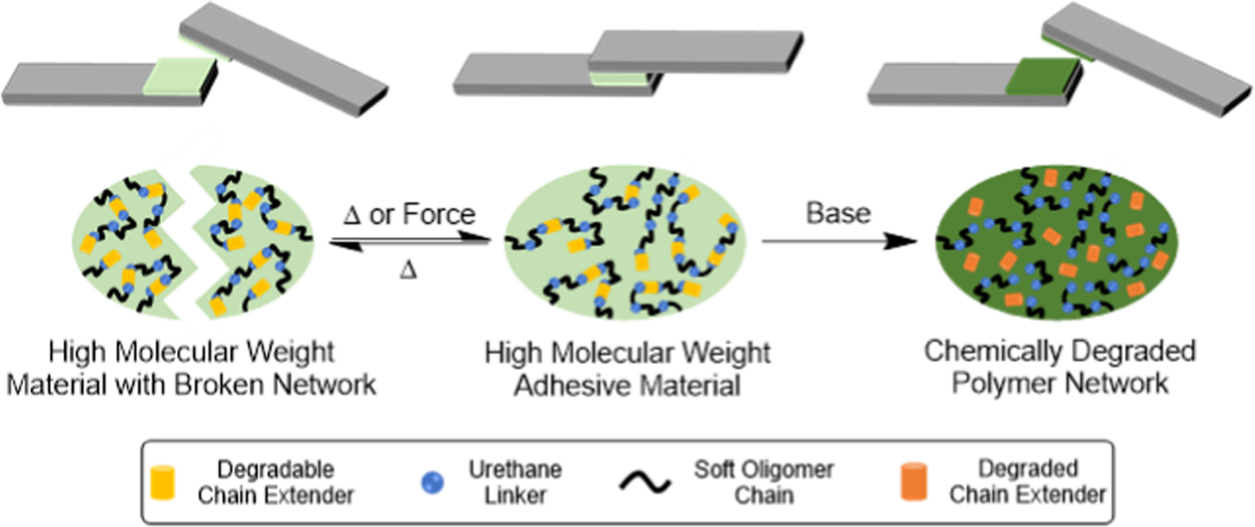

A series of novel chain-extended polyurethanes (CEPUs) featuring degradable sulfonyl ethyl urethane chain-extenders that permit degradation under base-triggered conditions to afford “debond-on-demand” elastomeric adhesives are reported. Exposure of the CEPUs to *tetra*-butylammonium fluoride (TBAF) triggered the degradation of the sulfonyl ethyl urethane chain-extenders. Lap shear adhesion tests of the CEPUs exposed to TBAF revealed reductions in shear strength of up to 65% for both aluminum and glass substrates, from 2.18 to 0.76 MPa and from 1.13 to 0.52 MPa, respectively. The selective depolymerization of these polymers makes them suitable candidates as debondable binders for inkjet inks and coatings, enabling removal of inks and adhesive residues from substrates before they enter the recycling process, to prevent surface contaminants decreasing the quality of the recycled material.

## 1 Introduction

Modern society and government policies now demand that polymeric materials, whether used within complex multicomponent devices or in simple, everyday items such as shopping bags or water bottles, are designed and made with an ever-increasing shift toward recyclability and sustainability.^1−5^ New biodegradable and recyclable polymers have thus been developed to meet these demands. One class of polymer that has been developed to meet these needs are stimuli-responsive polymers (SRPs).^6^ SRPs change their chemical and physical properties at specific locations within their molecular architectures in response to specific external physical, biological, or chemical stimuli.^6−8^ This characteristic of SRPs allows for their use in a wide range of applications such as sensors,^8^ drug delivery,^9^ self-healing materials,^10^ shape-changing materials,^11,12^ and adhesives.^10,13,14^ The use of SRPs as adhesives allows for so-called “debond-on-demand” behavior.^14,15^ On incorporation of supramolecular^16−20^ and dynamic covalent bonding units,^21−23^ polymeric adhesives can be realized with the ability to undergo multiple adhesion/separation/adhesion cycles without loss in adhesive strength.^10,24^

Hydrogen bonding has been widely exploited in supramolecular adhesives. The highly directional and reversible nature of these noncovalent bonds renders them prime candidates for use in “debond-on-demand” adhesives. Binding motifs such as the quadruple hydrogen-bond-forming 2-ureido-4[1H]-pyrimidinone (UPy) unit, first developed by Meijer and co-workers,^25^ has been applied to thermally and light-triggered “debond-on-demand” adhesives by Weder and co-workers.^17,19,20^ Another extremely effective hydrogen-bond receptor has been inspired by the adhesion mechanism of 3,4-dihydroxyphenylalanine (DOPA) and utilized in adhesives; it takes advantage of the catechol moiety which mediates robust adhesion through combinations of hydrogen bonding, metal coordination, and π–π interactions.^26−29^

Dynamic covalent bonding processes have also proven effective in the discovery of reversible adhesives.^30^ Common routes for introducing dynamic covalent bonds include the use of thermally and UV reversible disulfide bonds,^31,32^ as well as (retro-)Diels–Alder chemistry, the latter being exemplified by Slark and co-workers with network formation from dimaleimide and trifuran units.^33^ Other successful avenues include studies reported by Huang and co-workers, who have described a thermally self-healable adhesive based on a poly(1,2,3-triazolium) vitrimer which exhibits shear strengths up to 23.7 MPa.^34^ Through dynamic quarternization of the cross-links, retention of 51% of the shear strength was achieved after 20 readhesion cycles.

Self-immolative polymers (SIPs) are a unique class of SRPs which possess the ability to undergo depolymerization to monomeric^35,36^ or oligomeric^37^ units in either a stepwise or concerted manner upon the removal of covalently labile groups.^38^ The origin of many SIP systems stems frequently from the detailed assessment of highly atom-efficient protecting-group chemistries.^39,40^ The efficacy of such protection/deprotection methods renders them suitable for effective depolymerization processes and readily permits the introduction of so-called “triggering units” within the polymer architecture to allow for tailoring of selective degradation upon exposure to the appropriate stimuli.^38^ Self-immolative spacers can be used for amplification of reporter release in self-immolative systems. Indeed, self-immolative spacers such as 2,6-bis(hydroxymethyl)-*p*-cresol have been used in both polymeric and dendritic systems in conjunction with photolabile^37,41,42^ and hydrogen peroxide,^43,44^ glutathione,^45^ and fluoride^46−48^ labile protecting groups.

Degradable oligomers and polymers which do not employ self-immolative chemistries have also been widely explored.^49−51^ DelRe et al. have realized semicrystalline polyesters which undergo near-complete depolymerization through chain-mediated progressive depolymerization from nanoscopically dispersed enzymes.^52,53^ Through base-catalyzed thiol-thioester exchange processes, Bowman and co-workers have successfully developed a degradable 3D-printable polymer composite which allows for the recovery of 91% of the filler in the composite after 12 h.^54^ Incorporation of the degradable dibenzo[*c*,*e*]oxepin-5(7H)-thione (DOT) monomer into a pressure-sensitive adhesive (PSA) copolymer allows for selective breakage and dissolution of cross-linked polymeric networks via aminolysis and thiolysis.^55^

2-Methylsulfonylethyl carbamate (Msc)^56−58^ and its derivatives^58,59^ are established protecting groups used in peptide and carbohydrate chemistry for the protection of alcohols and amino groups. Treatment of the Msc protecting group with basic media leads to rapid and efficient cleavage via an β-elimination process. This approach has been applied to base-triggered depolymerizable systems in poly(olefin sulfone)s (POSs) by Shinoda et al.^60^ who observed decomposition of poly(4-hydroxystyrene sulfone) in aqueous NaOD to quantitatively afford trans-2-(4-hydroxyphenyl)ethylene sulfonic acid and 4-hydroxystyrene. Lobez and Swager^61^ prepared cross-linked POS-silicone composites with tailorable properties which depolymerized upon the addition of piperidine. Sasaki and co-workers^62−66^ have also developed the photoinduced depolymerization of POSs through the introduction of photobase-generating groups present on pendant side chains. Chain-extended and cross-linked polyurethane (CEPU) adhesives that degrade and debond when exposed to fluoride ions have been reported by Greenland and co-workers that are composed of novel silyl-protected biscarbamate^47,48^ and tricarbamate^46^ cross-linkers. Basic conditions are used in modern recycling processes to treat and recover aluminum, glass, and plastic waste. Inspired by the above studies, we have designed and synthesized a series of novel CEPUs featuring Msc-based chain-extenders that degrade under basic triggered conditions to afford “debond-on-demand” elastomeric adhesives.

## 2 Results and Discussion

“Debond-on-demand” adhesives required for commercial applications are highly sought-after, with the need for such materials to be synthetically simple to produce, possess high shear strength, and rapidly debond upon exposure to an appropriate stimulus.^5^ The incorporation of the commercially available 2,2′-sulfonyldiethanol unit as a chain-extender affords CEPUs that rapidly degrade upon exposure to base. To this end, a series of CEPUs that thermally debond-on-demand have been realized comprising a hydrogenated poly(butadiene) polyol with varying ratios of diisocyanates and diol chain-extender components.

**Scheme 1:**
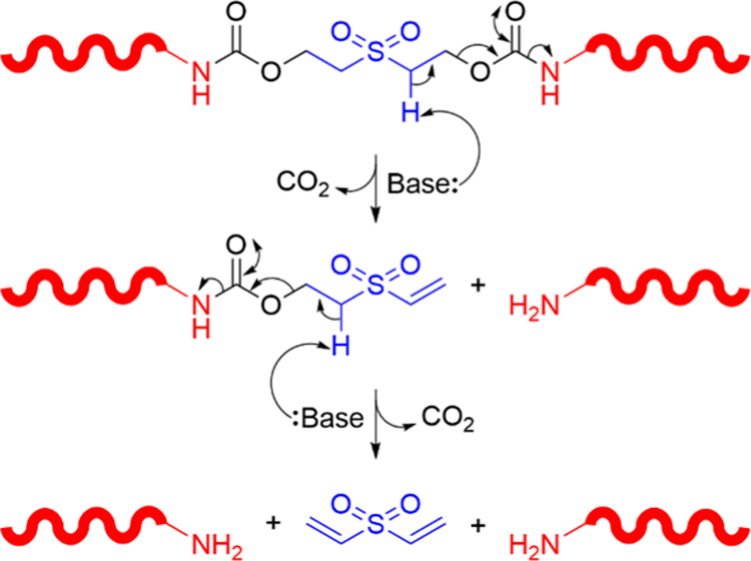
Schematic Representation of the Base-Triggered Degradation of a CEPU Containing the 2,2′-Sulfonyldiethanol Chain-Extender Unit

The proposed self-immolative degradation of these CEPUs proceeds via β-elimination of the ethyl-sulfone linker to form a vinylic species followed by decarboxylation and release of an amine-functionalized prepolymer chain (Scheme 1). This degradation pathway is then repeated to afford divinyl sulfone and release of a second prepolymer chain.

### 2.1 Synthesis and Characterization of Model Small-Molecule Analogues

**Figure 1:**
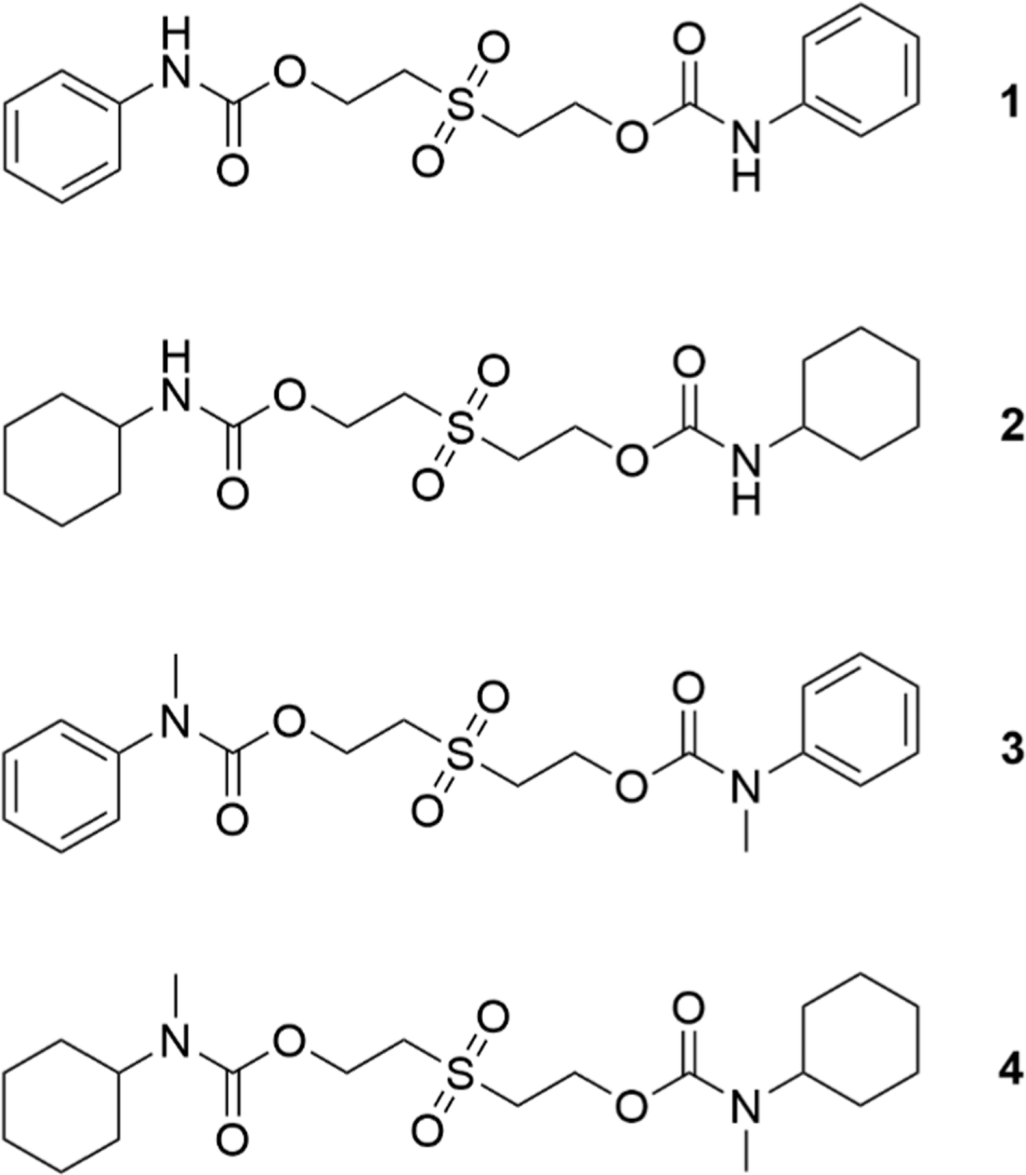
Model bisurethanes **1**–**4** studied.

Before synthesizing and testing the chain-extended polymer systems containing the degradable sulfonyl ethyl urethane (SEU) unit, it was important to understand the degradation pathway and establish the nature of any intermediates and byproducts.^38,43,67,68^ A series of model bis-urethane compounds were thus synthesized (see **1** and **2** in Figure 1) with derivatives comprising aromatic or aliphatic reporter moieties to mimic the key degradable units in the proposed polymers. Indeed, bisurethanes **1** and **2** are chemically representative model compounds for the chain-extended backbone of the CEPU studied. The reactions to afford urethanes **1** and **2** were monitored by FTIR spectroscopy to observe the consumption of the isocyanate group, νN=C=O_(stretch)_ 2275–2250 cm^–1^, and the formation of the corresponding urethane bonds. To establish that degradation occurred via β-elimination and not through direct hydrolysis of the urethane linkages, *N*-methylated urethane derivatives were also synthesized, **3** and **4** (Figure 1).^69^ The synthetic protocols used to afford compounds **1**–**4** and the associated characterization data can be found in the Supporting Information.

The model SEU compounds also provide an insight into the interchain interactions in the CEPUs as single crystals of model urethanes **1**–**4** were grown via vapor diffusion or slow evaporation and studied by X-ray crystallographic analysis (see the Supporting Information for the solid-state structures and data). The solid-state structures of compounds **2**–**4** reveal that the oxygen atoms of the sulfone moieties act as hydrogen-bond acceptors for alkyl (**2**–**4**), aromatic (**3**), and carbamate (**2**) hydrogen-bond donors. Ordered one-dimensional growth through carbamate–carbamate hydrogen bonding was observed for urethane **1**. Interestingly, urethane **2** exhibits intramolecular carbamate–carbamate hydrogen bonding. Furthermore, π–π interactions were also observed in the solid-state structures of the model compounds featuring aromatic units; for example, displaced parallel π–π interactions were evident for urethane **1** (C···C distances ∼3.4 Å) and T-shaped interactions (C···C distances ∼3.7 and 3.9 Å) were present in urethane **3**.

**Figure 2:**
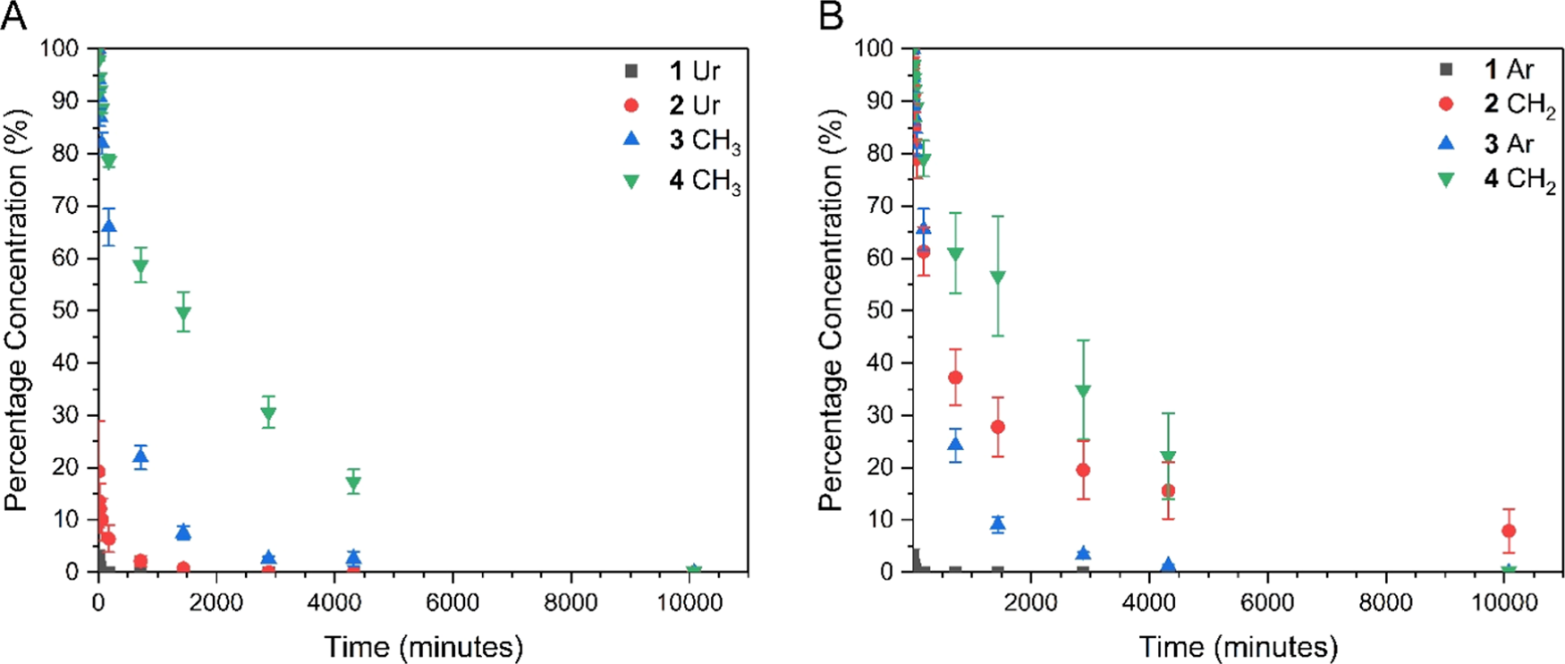
Degradation kinetics of model urethanes **1**–**4** calculated from the ^1^H NMR spectra (20 °C) obtained following the addition of 5 molar equiv of 40 wt % NaOD in D_2_O to a 10 mg mL^–1^ solution of model urethane in MeCN-*d*_3_. (A) Urethane hydrogen resonance (**1** and **2**) and *N*-methyl urethane hydrogen resonance (**3** and **4**); (B) aromatic hydrogen resonance (**1** and **3**) and methylene resonance (**2** and **4**). The error shown is the standard deviation of the three repeats for each sample.

Degradation analysis was conducted by addition of 5 mol equiv of either NaOD (Figure 2) or TBAF (Figure S32) to solutions (10 mg mL^–1^) of model urethane **1**–**4** in MeCN-*d*_3_. In the presence of NaOD, hydrolysis of the urethane was observed in addition to β-elimination for the model compounds **1** and **2**, as well as an increase in the rate of degradation when compared to that of the *N*-methylated urethane derivatives **3** and **4**. Degradation initiated by the addition of TBAF resulted in a significant increase in the rate of degradation for model urethanes **2**–**4**, with all four systems experiencing greater than 90% loss within 5 min, see Figures S32–S40 and Tables S16–S19. This increase in rate can be attributed to the improved miscibility between the acetonitrile solution and base solution. When exposed to TBAF, the rate of hydrolysis was reduced with a less than 2% variation between the urethane and *N*-methylated urethane derivatives. The presence of an aromatic reporter moiety (**1** and **3**) was observed to increase the rate of degradation when compared to that of the aliphatic reporter systems (**2** and **4**).

For all model urethanes, after addition of base, the formation of divinyl sulfone was observed with resonances correlating to the vinyl units evident in the ^1^H NMR spectra at 6.72, 6.33, and 6.16, respectively. In addition, the formation of secondary products was observed through side reactions with divinyl sulfone. These secondary products result from oxa-Michael and Michael addition reactions to form a range of secondary products including 1,4-oxathiane-4,4-dioxide^70−72^ as well as oligomeric^72,73^ and macrocyclic species.^73,74^ The formation of these species was confirmed by mass spectrometry; see Figure S48.

The exposure of the model urethanes **1**–**4** to basic conditions (NaOD or TBAF) revealed that degradation occurs via the proposed β-elimination/decarboxylation/amine release pathway and confirmed the suitability of the bis-urethane sulfone system as a degradable unit in CEPU backbones. However, in the absence of *N*-methylated urethanes, the exposure of urethanes **1** and **2** to NaOD revealed that degradation also occurs via hydrolysis of the urethane linkage.

### 2.2 Synthesis and Characterization of CEPUs

**Scheme 2:**
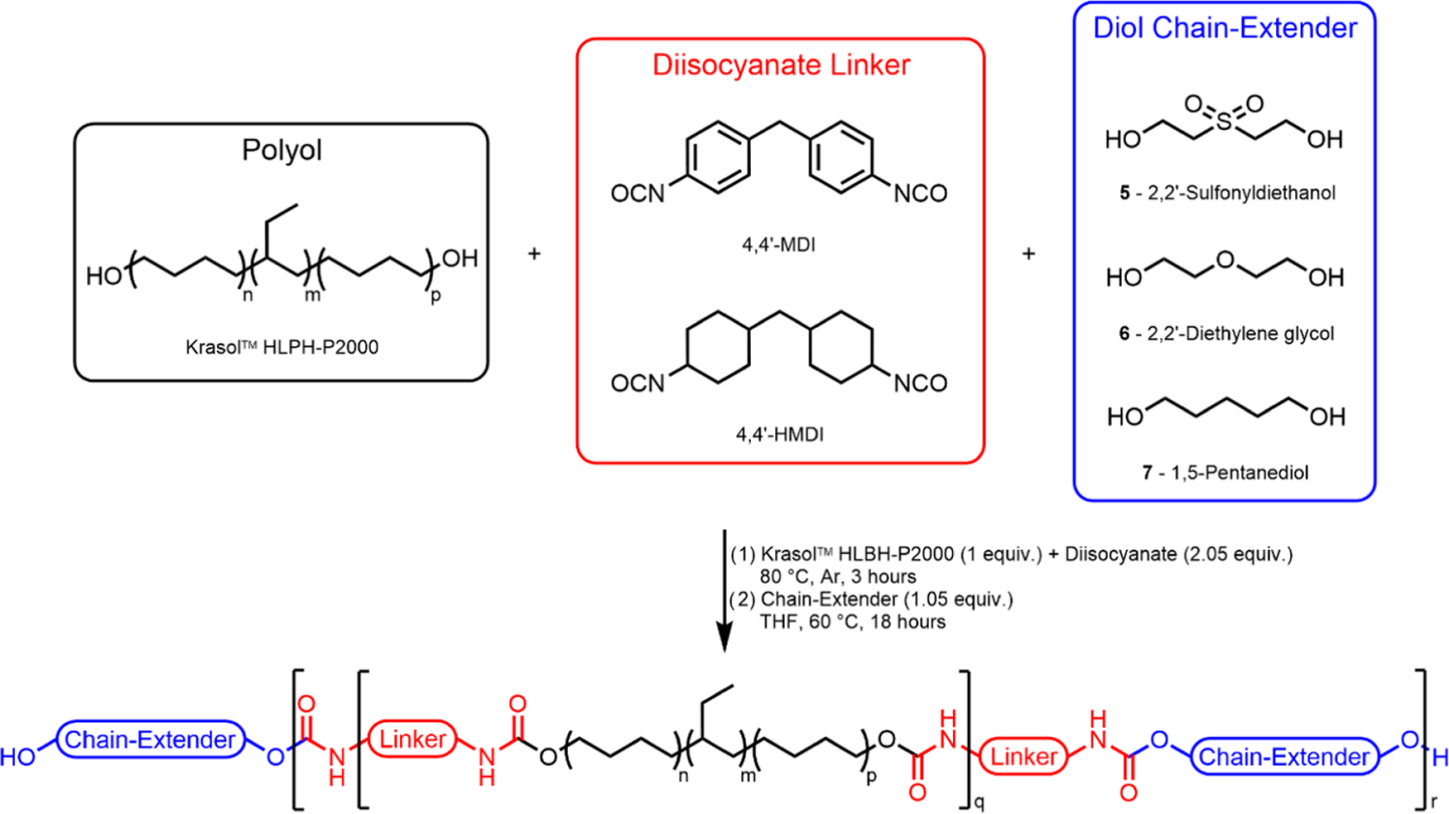
General Synthetic Protocol to Afford **CEPU1**–**CEPU6** Composed of Different Diisocyanates and Chain-Extenders

Following the studies of the model urethanes, a series of novel CEPUs, **CEPU1**–**CEPU6**, were generated via a one-pot, two-step synthesis that has previously been employed to generate fluoride-specific degradable CEPU adhesives^47,48^ and healable materials.^24,75,76^ Briefly, Krasol HLBH-P2000 was reacted in bulk with 2.05 equiv of diisocyanate linker at 80 °C for 3 h to afford an isocyanate-terminated prepolymer. The diisocyanate linkers and diol chain-extenders (**5**–**7**) used are detailed in Scheme 2, and the constituents of each CEPU adhesive are detailed in Table 1. Each reaction vessel was then cooled to room temperature and solvated with THF, and 1.05 equiv of the diol chain-extender was added before the contents were brought to and maintained under reflux to afford the CEPUs (**CEPU1**–**CEPU6**). Each reaction was monitored by FTIR spectroscopy, and upon disappearance of the isocyanate band, the polymers were then purified by repeated precipitations into methanol. Polymer films were subsequently solvent cast from a concentrated solution of THF, and the influence of varying the chain-extender and diisocyanate on the CEPU could be assessed through mechanical property and degradation testing.

**Table 1:** Chemical Composition of **CEPU1**–**CEPU6** Containing Different Diisocyanates or Chain-Extenders (Yields Are Shown in Parentheses), GPC Molecular Weight and Dispersity Data, *Đ*, for **CEPU1**–**CEPU6** (Error Shown Is the Standard Deviation between the Three Repeats of Each Sample), and Thermal Properties of **CEPU1**–**CEPU6**

CEPU	diisocyanate linker	diol chain-extender	*M*_n_ (g mol^–1^)	*M*_w_ (g mol^–1^)	*Đ*	*T*_g_ (°C)^b^	*T*_m_ (°C)^a^	*T*_m_ (°C)^b^
**CEPU1** (95%)	4,4′-MDI	**5**	30,100 ± 300	124,900 ± 0	4.15	–47.2		102.4
**CEPU2** (92%)	4,4′-HMDI	**5**	44,700 ± 200	140,400 ± 700	3.14	–47.1	50.1	
**CEPU3** (94%)	4,4′-MDI	**6**	27,700 ± 0	86,900 ± 1600	3.14	–46.2	56.2; 82.2	
**CEPU4** (91%)	4,4′-HMDI	**6**	57,400 ± 500	197,000 ± 300	3.43	–44.2	53.0	
**CEPU5** (96%)	4,4′-MDI	**7**	26,600 ± 100	90,900 ± 900	3.42	–46.0		101.6
**CEPU6** (90%)	4,4′-HMDI	**7**	64,800 ± 800	199,500 ± 100	3.08	–45.1	45.7	

a First heating run, 10 °C min^–1^.

b Second heating run, 10 °C min^–1^.

^1^H NMR spectroscopic analysis revealed a ratio of 1:1 for the resonances associated with the prepolymer urethanes and chain-extender urethanes, which is consistent with the feed ratios (see the NMR spectroscopic data in the Supporting Information, Figures S9–S20 and Table S1). ^13^C NMR spectroscopy was also used to confirm the formation of the urethane linkages; prepolymer urethane linkages were observed at ca. 154.5 ppm (**CEPU1**, **CEPU3**, and **CEPU5**) or ca. 156.6 ppm (**CEPU2**, **CEPU4**, and **CEPU6**). FTIR spectroscopic analysis of the CEPUs showed characteristic absorbance bands at approximately 3300 and 1700 cm^–1^ for N–H and C=O stretches, respectively. GPC analysis of the polymers (Figures S49–S54 and Table 1) was employed to identify the molecular weights of the CEPUs. All CEPUs exhibited broad monomodal distributions in molecular weight with values for *M*_n_ > 30,000 g mol^–1^. The thermal properties of **CEPU1**–**CEPU6** were assessed by DSC analysis, see Table 1. In the second heating cycle, all of the CEPUs exhibited a *T*_g_ of ca. −45 °C that is associated with the glass transition of the poly(ethylene-*co*-butylene) soft domain.^24,47,48,76^ All of the CEPUs experienced weak irreversible melt transitions in either the first or second heating cycle, with the CEPUs consisting of aliphatic diisocyanates, **CEPU2**, **CEPU4**, and **CEPU6**, exhibiting low-temperature *T*_m_ transitions. For the CEPUs composed of aromatic diisocyanates, **CEPU1**, **CEPU3**, and **CEPU5**, the *T*_m_ transitions occur at higher temperatures. TGA analysis was employed to determine the maximum processing temperatures; samples were heated from 20 to 550 °C at a rate of 10 °C min^–1^ under a nitrogen atmosphere. **CEPU2** exhibited the lowest temperature for the onset of degradation at 229 °C, and all CEPUs had fully degraded upon reaching 475 °C (Figures S65–S70).

**Figure 3:**
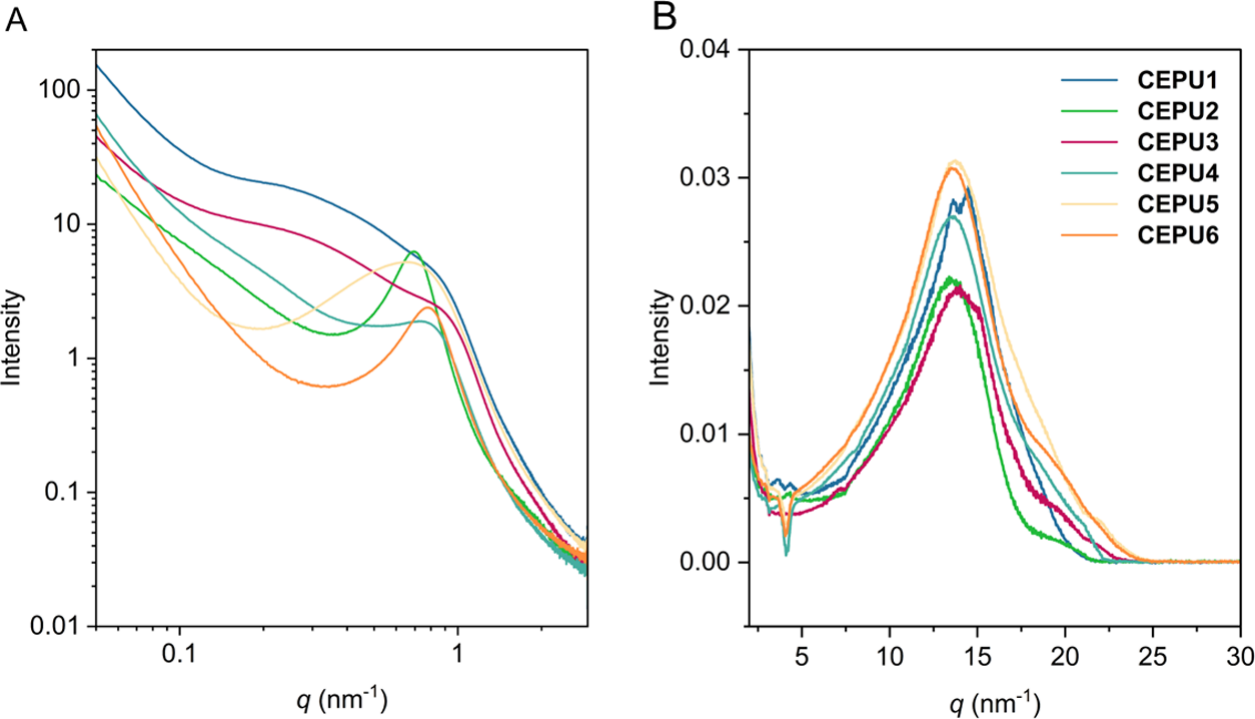
(A) SAXS and (B) WAXS intensity profiles of **CEPU1**–**CEPU6**. Data was acquired at 20 °C.

The microphase-separated morphology of the CEPUs is critical to their physical and mechanical properties, thus small-angle X-ray scattering (SAXS) was employed to investigate their microphase separation at room temperature (see Figures 3A and S73–S78). All CEPUs exhibit broad scattering peaks corresponding to nonuniform-microphase-separated domains arising from the immiscibility between the hard hydrogen bonding units and the soft poly(butadiene) domains, with scattering vector, *q*_peak_, ranging from 0.9 to 0.6 nm^–1^, where *q* is the scattering vector, corresponding to *d*-spacings of 7.4–9.8 nm, for all *q*_max_ and *d*-spacing values (see Table S20). These domain sizes are comparable to those reported by Greenland and co-workers^48^ in the case of their degradable CEPUs, as well as those from Feula et al.^76^ for their supramolecular polyurethanes, all of which contain hydrogenated poly(butadiene) soft segments. Sharper scattering peaks are observed for the aliphatic diisocyanate CEPUs (**CEPU2**, **CEPU4**, and **CEPU6**) corresponding to more uniform microphase separation when compared with their aromatic counterparts. The SAXS profiles of **CEPU1** and **CEPU3** both show a second broad peak with *q*_max_ at 0.2 nm^–1^ (*d*-spacing of 31.4 nm), corresponding to the aggregation or coalescence of the microphase-separated domains.

Wide-angle X-ray scattering (WAXS) was simultaneously performed to investigate the ordering within the hard domains of the CEPUs (Figure 3B). All CEPUs exhibit a broad diffraction peak at approximately 13.6 nm^–1^, relating to a spacing of 0.46 nm and corresponding to the hydrogen bonding urethane residues.^77^**CEPU1** and **CEPU3** both show well-resolved peaks at 14.5 nm^–1^ (length scale = 0.43 nm) and 15.1 nm^–1^ (length scale = 0.42 nm), respectively, suggesting the presence of regions with a certain degree of ordering and may correspond to urethane lateral spacings.^78^

**Figure 4:**
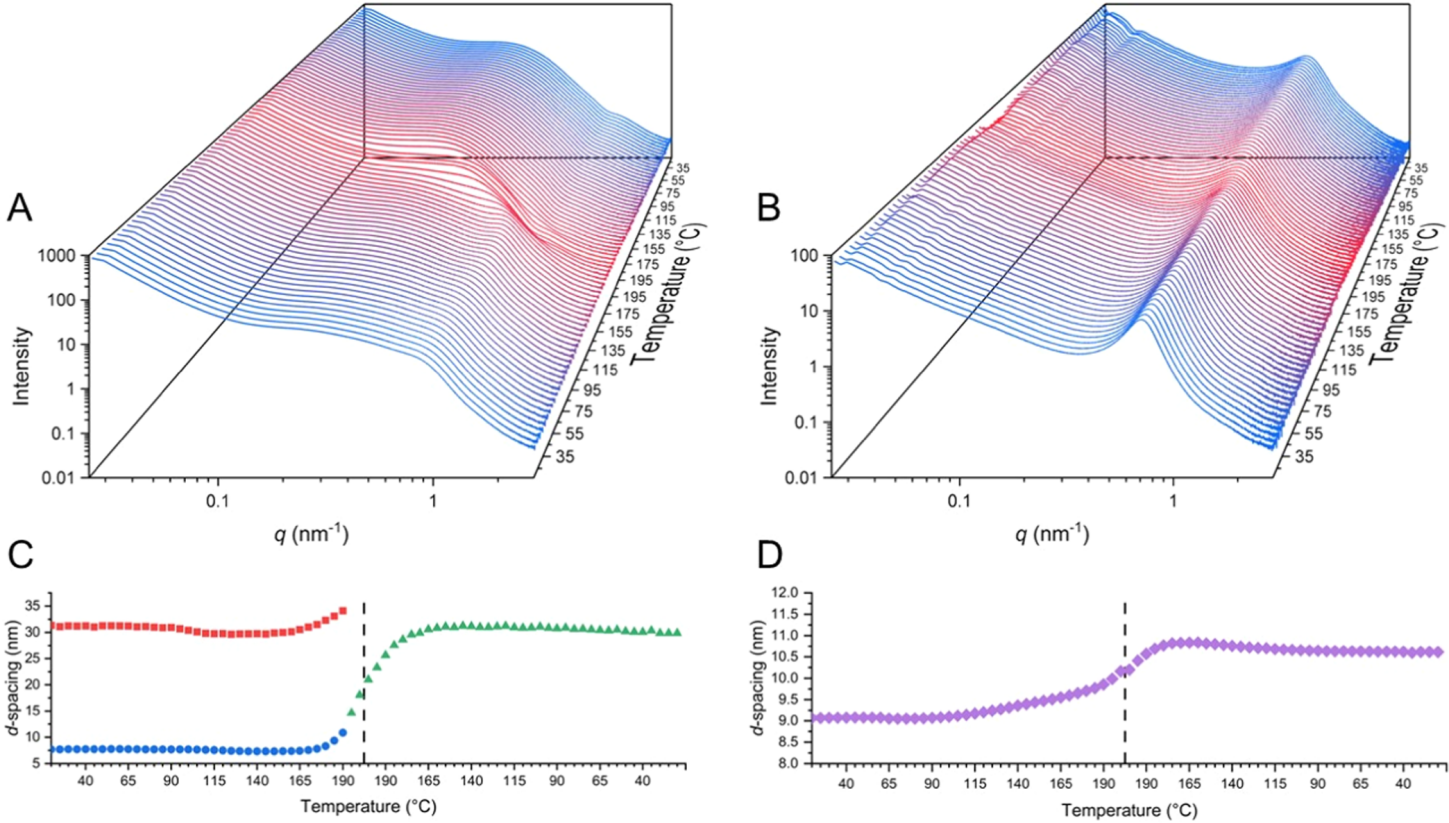
vt-SAXS of **CEPU1** (A) and **CEPU2** (B) recorded at 5 °C intervals from 20 to 200 °C at a heating and cooling rate of 10 °C min^–1^. Shifts in *d*-spacing for **CEPU1** (C) and **CEPU2** (D) over the heating and cooling cycles; dashed line represents transition from heating to cooling cycle.

The temperature susceptibility of the microphase-separated morphology and supramolecular associations was probed via variable-temperature (vt) SAXS and WAXS at 5 °C intervals from 20 to 200 °C with a heating and cooling rate of 10 °C min^–1^ (see Figures 4, S85, and S86). **CEPU1** shows the most significant changes in the SAXS: on heating above 190 °C, convergence of the two peaks occurs, corresponding to the loss of smaller phase separation within the CEPU and the generation of larger phase-separated morphologies. Upon cooling, there is no reformation of the small phase-separated morphologies in **CEPU1** and **CEPU2**. The vt-WAXS of **CEPU1** shows the retention of the defined reflections at 13.6 and 14.5 nm^–1^ over both the heating and cooling cycles with only broadening of the main scattering peak observed. vt-WAXS analysis of **CEPU2** reveals the broadening of the scattering peak and a shift in *q*_max_ corresponding to a length-scale shift from 0.47 to 0.49 nm, which upon cooling returned to 0.47 nm and is attributed to subtle changes in the hydrogen-bond length between the urethane moieties (see Figure S86).

**Figure 5:**
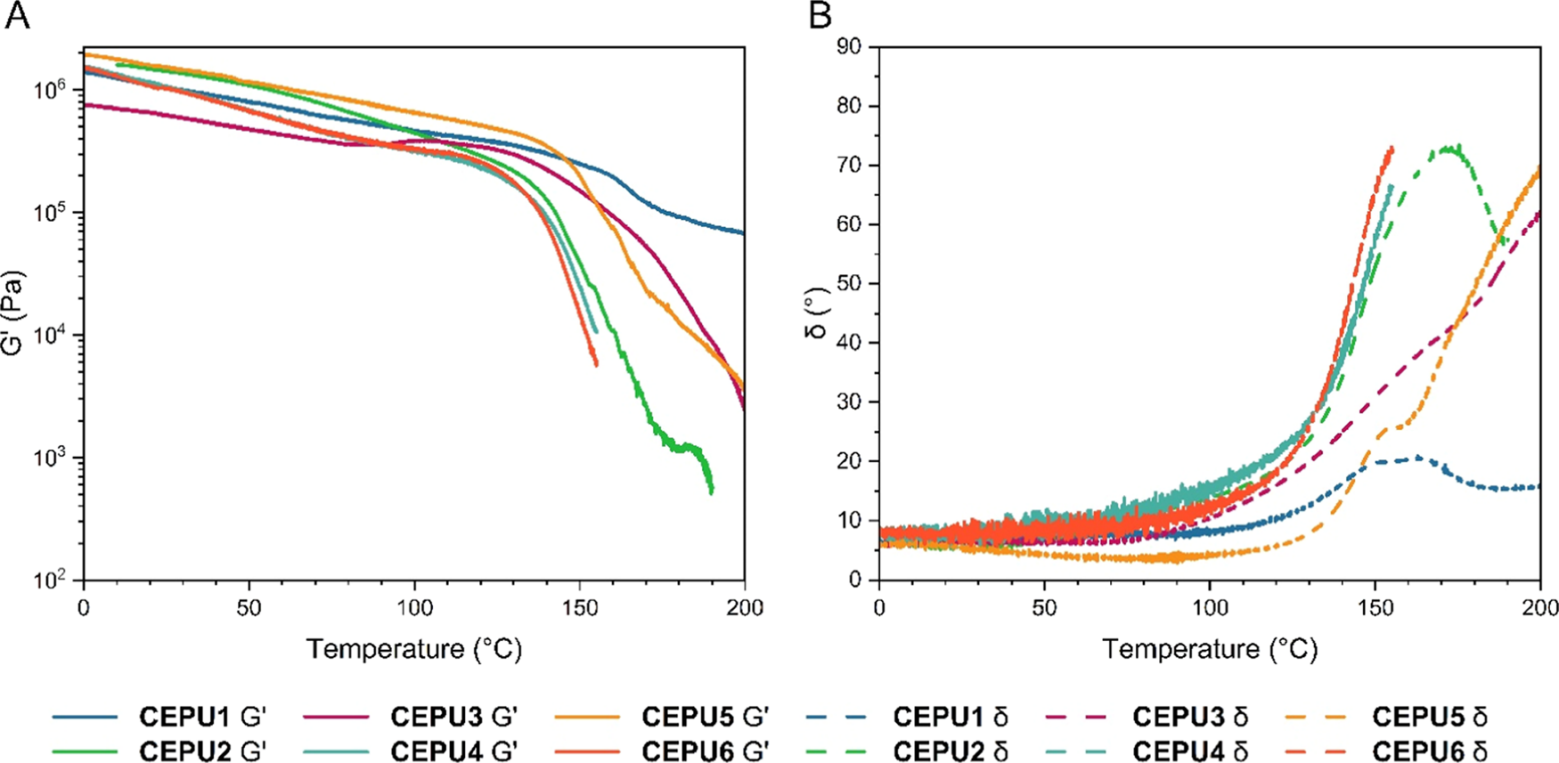
Temperature sweep analysis of **CEPU1**–**CEPU6** over a temperature range of 0–200 °C, measured using a normal force of 1 N and a frequency of 1 Hz. (A) storage modulus (*G*′) versus temperature, (B) phase shift (δ) versus temperature.

Dynamic rheological testing of the **CEPU1**–**CEPU6** polymers was conducted to assess their viscoelastic properties (see Figure 5). At temperatures below 100 °C, the storage modulus (*G*′) dominates, with the CEPUs behaving as viscoelastic solids. As the temperature was increased, the effect that the composition of CEPU has on the supramolecular network dissociation became apparent. Incorporation of the aliphatic 4,4-HMDI in **CEPU2**, **CEPU4**, and **CEPU6** resulted in material flow and the crossover between *G*′ and the loss modulus (*G*″) at lower temperatures when compared to their CEPU counterparts which feature the aromatic 4,4-MDI, i.e., **CEPU1**, **CEPU3**, and **CEPU5**. The presence of hydrogen bonding acceptor moieties in the chain-extenders of **CEPU2**, sulfone, and **CEPU4**, ether, shift the temperature at which crossover between *G*′ and *G*″ is observed to higher temperatures when compared to **CEPU6**; i.e., at 145.8, 144.3, and 141.1 °C, respectively. **CEPU3** and **CEPU5** both resist terminal flow and crossover between *G*′ and *G*″ until higher temperatures when compared to their aliphatic counterparts, at 177.5 and 176.1 °C, respectively. This difference is attributed to the increased order of the hard domains. Thus, **CEPU1**, **CEPU3**, and **CEPU5** undergo an initial relaxation event at ca. 120 °C, presumably softening of the hard domains, with **CEPU3** and **CEPU5** both undergoing a second relaxation event at ca. 160 °C, which results in terminal flow and the crossover between *G*′ and *G*″. However, a new rubbery plateau was evident for **CEPU1** and crossover was not observed. The occurrence of a rubbery plateau and reordering of the hard domains in **CEPU1** is also observed for the aliphatic **CEPU2** suggesting that the incorporation of the sulfone chain-extender has a significant influence on the thermal and rheological characteristics of these CEPUs.

**Figure 6:**
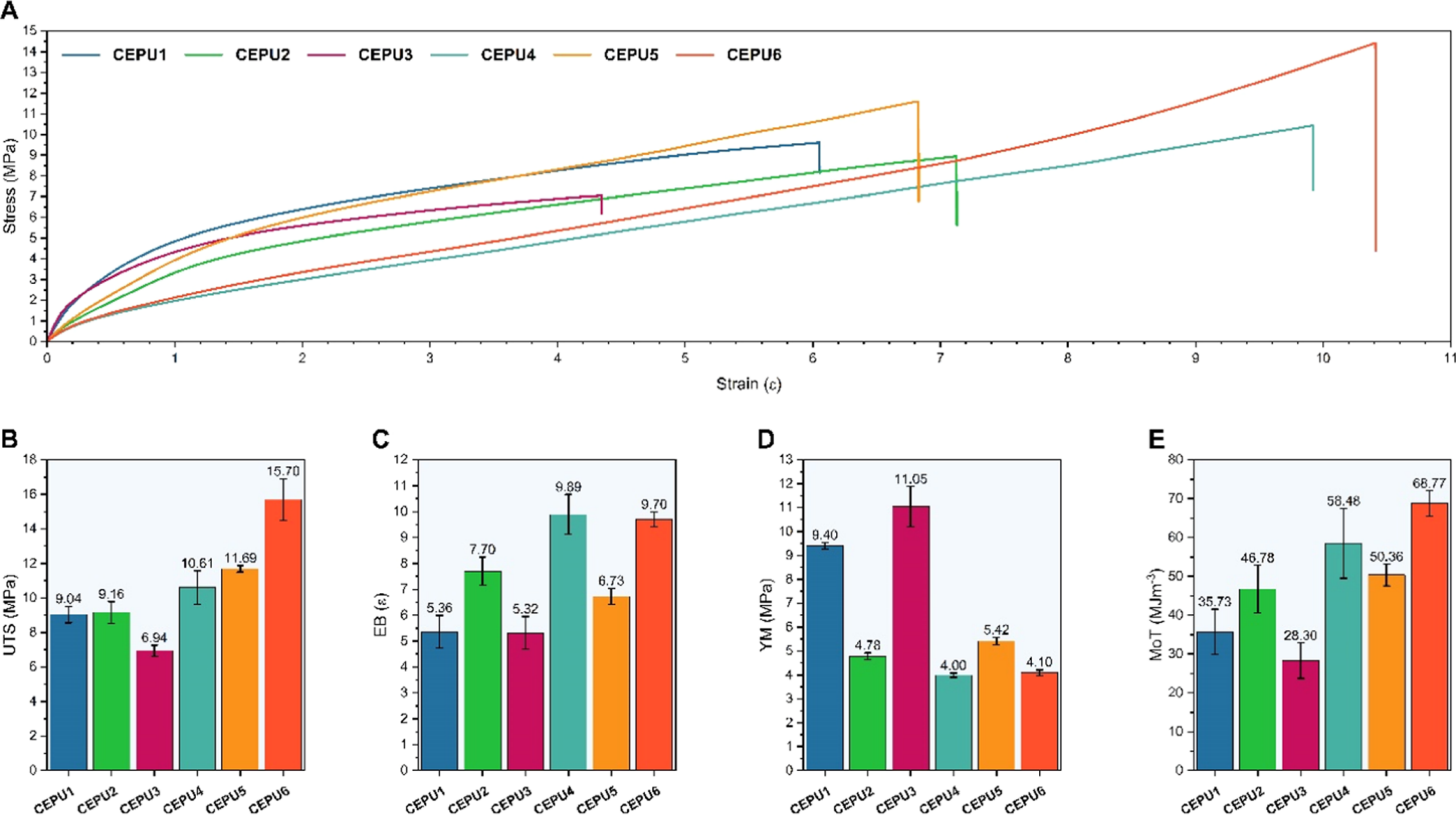
(A) Representative stress–strain curves for **CEPU1**–**CEPU6**. Comparison of (B) ultimate tensile strength (UTS), (C) elongation at break (EB), (D) Young’s modulus (YM), and (E) modulus of toughness (MoT). The error shown is the standard deviation between the three repeats for each sample.

Probing the mechanical properties of the CEPUs through tensile stress–strain measurements at 10 mm min^–1^ illustrates how varying the composition of the CEPUs affects their unique mechanical properties (Figure 6). Characteristic lower ultimate tensile strengths (UTS), lower elongations at break (EB), and increased Young’s moduli (YM) were observed for the aromatic CEPUs (**CEPU1**, **CEPU3**, and **CEPU5**) resulting from the increased supramolecular interactions through π–π stacking and increased phase separation between the hard and soft domains, see Table 2.^79^ Increased hydrogen-bond acceptor functionality of the chain-extenders in **CEPU1**–**CEPU4** resulted in a decrease in the UTS and EB values when compared with those of **CEPU5** and **CEPU6** which possess a simple alkyl chain-extender. Similar behavior was also observed by Oprea et al.^80^ Significant increases in YM were also observed for the aromatic CEPUs: there is an increase in hydrogen-bond acceptor functionality for **CEPU1** and **CEPU3** when compared to **CEPU5**, 9.40 ± 0.1, 11.05 ± 0.8, and 5.42 ± 0.2 MPa, respectively, with **CEPU3** exhibiting a more than 2-fold increase.

**Table 2:** Effect of Polymer Composition on the Mechanical Properties of the CEPUs^a^

CEPU adhesive	UTS^b^ (MPa)	EB^c^ (ε)	Young’s modulus (MPa)	modulus of toughness (MJ m^–3^)
**CEPU1**	9.04 ± 0.5	5.36 ± 0.6	9.40 ± 0.1	35.73 ± 5.8
**CEPU2**	9.16 ± 0.6	7.70 ± 0.6	4.78 ± 0.1	46.78 ± 6.1
**CEPU3**	6.94 ± 0.3	5.32 ± 0.6	11.05 ± 0.8	28.30 ± 4.6
**CEPU4**	10.61 ± 1.0	9.89 ± 0.8	4.00 ± 0.1	58.48 ± 9.0
**CEPU5**	11.69 ± 0.2	6.73 ± 0.3	5.42 ± 0.2	50.36 ± 2.9
**CEPU6**	15.70 ± 1.2	9.70 ± 0.3	4.10 ± 0.1	68.77 ± 3.3

a The values recorded are the averages of three separate samples for each CEPU. The error shown is the standard deviation for the three repeats of each sample.

b UTS, ultimate tensile strength

c EB, elongation at break.

### 2.3 Solution and Solid-State CEPU Degradation Studies

**Figure 7:**
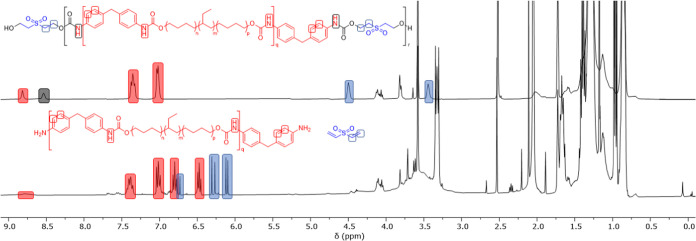
^1^H NMR spectra showing the solution degradation of **CEPU1** with 1 M TBAF in acetone, top pristine and bottom after 30 min (400 MHz, THF-*d*_8_).

To confirm that base-initiated degradation occurs in the polymeric system, solution-state NMR studies were conducted by addition of excess base, either NaOD or TBAF, to a sample of **CEPU1** and **CEPU2** in THF-*d*_8_ (5:1 molar equiv of base to degradable unit). The reaction was monitored by ^1^H and ^13^C NMR spectroscopy, see Figures 7 and S95–S101. Within 30 min of exposure to TBAF, the methylene resonances and chain-extender urethane resonance rapidly diminish and the vinylic protons of divinyl sulfone are evident at 3.43, 4.50, and 8.82 ppm for **CEPU1**, 3.26–3.41, 4.33, and 6.46–6.50 ppm for **CEPU2**, and at 6.11, 6.29, and 6.77 ppm for divinyl sulfone. The chain-extender urethane resonance in the ^13^C NMR spectra was also no longer evident post degradation, but there is the emergence of divinyl sulfone resonances at 154.1 ppm for **CEPU1**, 156.1 ppm for **CEPU2**, and 139.2 and 129.3 ppm for divinyl sulfone. Partial hydrolysis of the prepolymer urethane resonances for **CEPU1**, 8.54 and 154.5 ppm, and **CEPU2**, 5.95–6.07 and 156.6 ppm, was observed on the addition of NaOD; however, this was not observed from the addition of TBAF.

**Figure 8:**
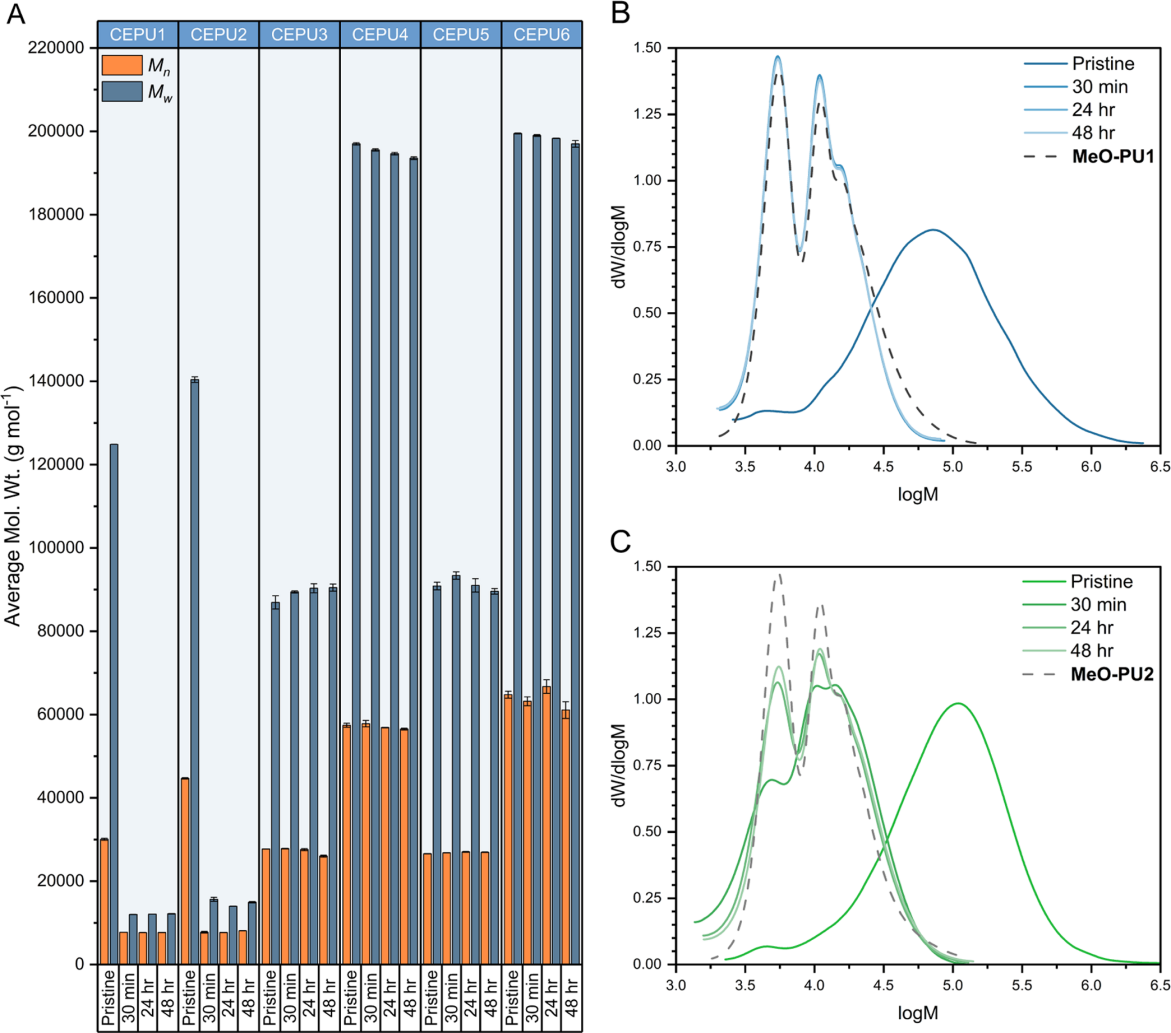
(A) *M*_n_ (orange) and *M*_w_ (gray) of **CEPU1**–**CEPU6** as pristine samples and 30 min, 24 h, and 48 h post addition of TBAF acquired from a THF GPC; the recorded are averages of three separate samples of each CEPU. The error shown is the standard deviation between the three repeats of each sample. (B) GPC eluogram of **CEPU1** in THF (blue) as a pristine sample and 30 min, 24 h, and 48 h post addition of TBAF and **MeO-PU1** (gray dashed). (C) GPC eluogram of **CEPU2** in THF (green) as a pristine sample and 30 min, 24 h, and 48 h post addition of TBAF and **MeO-PU2** (gray dashed).

GPC solution degradation studies of the CEPUs were conducted by the addition of TBAF (Figure 8). In the case of the CEPUs that did not contain the sulfone diol chain-extender **5** (**CEPU3**–**CEPU6**), exposure to TBAF did not afford any change in the *M*_n_ or *M*_w_ of the polymers (see Figure 8A). In stark contrast, inclusion of **5** in both **CEPU1** and **CEPU2** led to a dramatic drop (**CEPU1** Δ*M*_n_ ca. 22,400 g mol^–1^ and 74%) in both *M*_n_ and *M*_w_ after 30 min post addition of TBAF (e.g., **CEPU1***M*_n_(Pristine) 30,100 ± 300 to 7700 ± 0 g mol^–1^ and **CEPU2***M*_n_(Pristine) 44,700 ± 200 to 7800 ± 200 g mol^–1^). A marginal narrowing of the molecular weights was observed from 30 min to 48 h for both **CEPU1** and **CEPU2** exemplifying the rapid degradation of the sulfone moiety when in solution (for full details on the molecular weight data, see Table S21). The isocyanate-terminated prepolymers of the aromatic (**CEPU1**, **CEPU3**, and **CEPU5**) and aliphatic (**CEPU2**, **CEPU4**, and **CEPU6**) derivatives were end-capped with methanol (**MeO-PU1** and **MeO-PU2**, respectively). The molecular weights of **MeO-PU1** and **MeO-PU2** correlate to the molecular weights of their corresponding degraded CEPUs (see Table S21). GPC eluograms of **CEPU1** and **CEPU2**, Figure 8B,C, respectively, reveal a transition from a monomodal pristine polymer to the multimodal degraded polymer, which correlates with the methoxy-terminated polymers. This multimodal nature of the degraded polymers, **MeO-PU1** and **MeO-PU2**, corresponds to varying degrees of chain-extension in the prepolymer synthesis with the difference in the two main peaks attributed to the addition of a single prepolymer repeat unit.

The effect of solid-state degradation on the thermal properties of **CEPU1** and **CEPU2** was investigated using DSC, whereby cut sections of CEPUs (**CEPU1** and **CEPU2**) were submerged in solutions of 1 M TBAF in acetone or 40 wt % NaOH_(aq)_ at either room temperature (TBAF) or 50 °C (NaOH). The polymer samples were then washed with either acetone (TBAF) or deionized water (NaOH) and then dried at room temperature for 12 h under vacuum; the full experimental procedure can be found in the Supporting Information. Post degradation, all samples exhibited *T*_g_ transitions ca. −45 °C in the second heating cycle consistent with the *T*_g_ of the pristine samples, see Tables 1 and S22. Besides the NaOH degradation of **CEPU1**, the degradation of the sulfone chain-extender within the hard domains resulted in the disappearance of the irreversible *T*_m_ transitions of the pristine **CEPU1** and **CEPU2** at 102.4 and 50.1 °C, respectively. Low-temperature *T*_m_ and *T*_c_ transitions corresponding to **MeO-PU1** and **MeO-PU2** are not observed in the degraded samples, see Figures S63, S64, and S109–S112 and Table S22.

**Figure 9:**
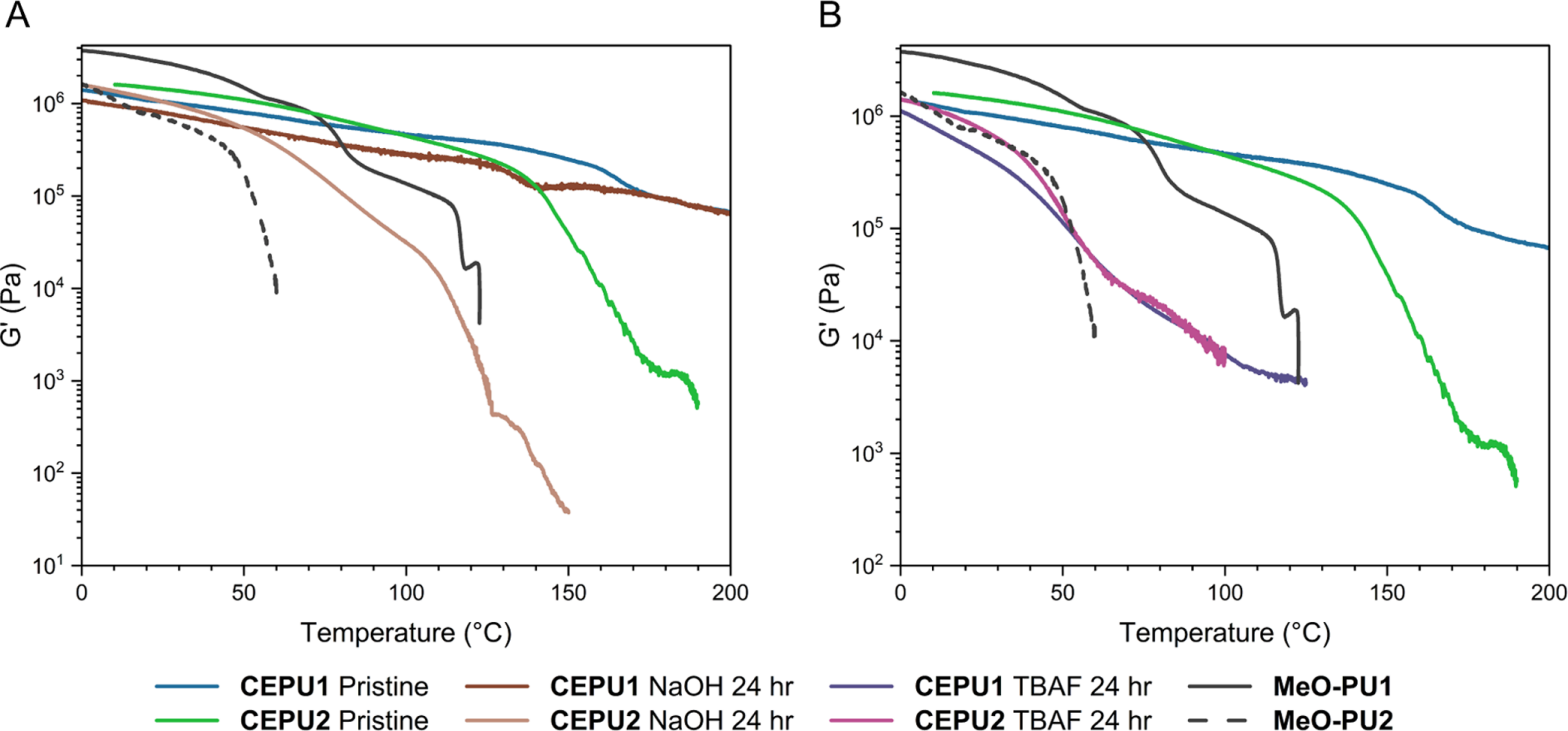
Temperature sweep analysis of solid-state degraded CEPUs using 40 wt % NaOH_(aq)_ or 1 M TBAF in acetone over a temperature regime of 0–200 °C, using a normal force of 1 N and a frequency of 1 Hz. (A) 24 h NaOH degraded *G*′ versus temperature, (B) 24 h TBAF degraded *G*′ versus temperature.

Changes in the viscoelastic properties of **CEPU1** and **CEPU2** were monitored by dynamic rheological testing after degradation of the solid-state polymer films. At *T* = 0 °C, the pristine and exposed samples of **CEPU1** all exhibit similar *G*′ values, see Figure 9. **CEPU1** was exposed to NaOH, for both 30 min and 24 h, and underwent an initial relaxation similar to the pristine material at ca. 120 °C. However, reordering of the hard domains occurs at lower temperatures with both samples. When exposed to aqueous base for 24 h, the sample exhibits a decrease in *G*′ at a lower temperature, ca. 125–140 °C, but a rheological profile comparable to that of the pristine material occurs from 170 to 200 °C. Lack of penetration by the aqueous base into the relatively hydrophobic polyurethane matrix was attributed to the inhibition of bulk degradation of the CEPU and the retention of the viscoelastic properties. When compared to exposure of the films of **CEPU1** to NaOH_(aq)_, contact with TBAF affected a significant change in the viscoelastic behavior of this material over the temperature regime investigated. Significant relaxations were observed for the sample after exposure for 30 min and 24 h. After 24 h in contact with TBAF, a viscoelastic transition was evident at 48.3 °C, followed by a transition back to a viscoelastic solid at 121.0 °C, see Figures 9B and S113. The high-temperature relaxation and reordering, analogous to those of the pristine sample, in the **CEPU1** sample exposed to TBAF for 30 min result from ordered hard domains consisting of the sulfone chain-extender still present within the bulk of the polymer. The increased number of urethane functionalities of the aromatic **MeO-PU1** shift the viscoelastic transition to 122.7 °C, when compared to **CEPU1** that had been degraded for 24 h with TBAF, resulting from the retention of the hard domains.

As in the case of **CEPU1**, the degraded samples of **CEPU2** all exhibit similar *G*′ values to the pristine sample from 0 to 10 °C, see Figure 9. Exposure of **CEPU2** with NaOH_(aq)_ for 30 min resulted in negligible change in *G*′ or δ when compared to the pristine material; both exhibit a significant relaxation event at ca. 120 °C. However, unlike **CEPU1**, increasing the degradation time to 24 h resulted in a noticeable change in the viscoelastic properties, with the relaxation of the hard domains occurring at ca. 35 °C and viscoelastic transition observed at 77.0 °C, see Figures 9A and S113. Consistent with **CEPU1**, degradation from TBAF results in increased changes in the viscoelastic properties compared to the pristine sample and NaOH_(aq)_ degradation. Degradation for 30 min and 24 h led to the onset of relaxations to occur at ca. 20–30 °C. Unsurprisingly, the longer exposure time shifts the viscoelastic transition to lower temperatures when compared to the sample that had been exposed for only 30 min, 45.2 and 88.2 °C, respectively. As evident with **MeO-PU1**, the presence of increased numbers of urethanes in **MeO-PU2** shifts the viscoelastic transition to higher temperatures when compared to the degraded samples, 55.8 °C. The variation in viscoelastic properties observed with the degraded samples from NaOH and TBAF for both **CEPU1** and **CEPU2** can be associated with the ability of acetone to better solubilize the degraded polymer chains which in turn allows the TBAF to penetrate better into the CEPU film than NaOH_(aq)_.

**Figure 10:**
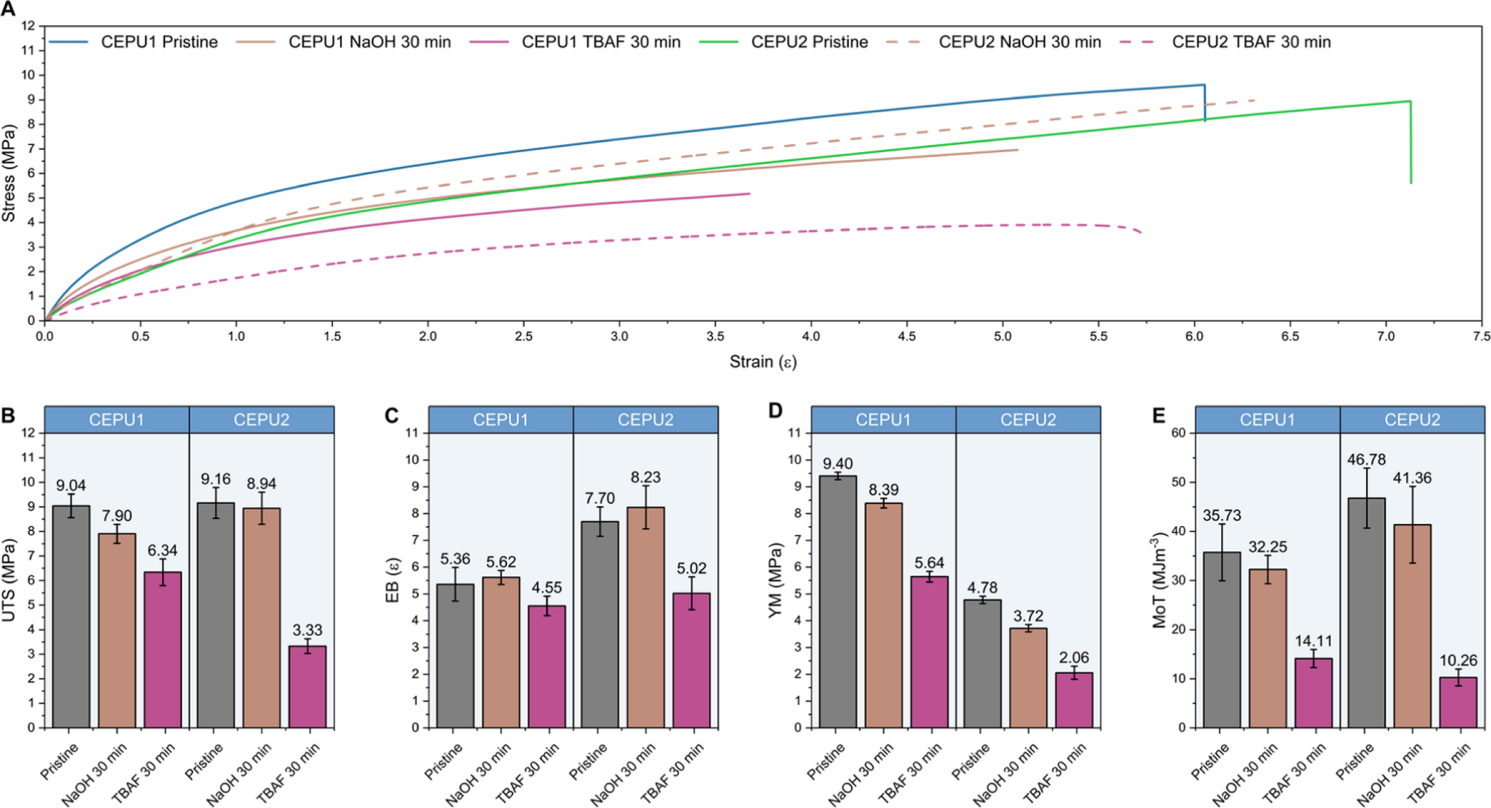
(A) Representative stress–strain curves of pristine and solid-state degraded **CEPU1** and **CEPU2** using 40 wt % NaOH_(aq)_ or 1 M TBAF in acetone for 30 min. Comparison of (B) ultimate tensile strength (UTS), (C) elongation at break (EB), (D) Young’s modulus (YM), and (E) modulus of toughness (MoT). The error shown is the standard deviation between the three repeats for each sample.

The mechanical properties of **CEPU1** and **CEPU2** post solid-state degradation were investigated through tensile analysis (Figure 10 and Table 3). Degradation of the polymer films was only conducted for 30 min to ensure sufficient structural integrity for testing. Treatment of **CEPU1** and **CEPU2** with NaOH resulted in ca. 10% decreases in the UTS, YM, and MoT with an increase of ca. 6% in the EB compared to the pristine CEPUs, see Table 3. This is consistent with the surface degradation of the CEPUs with the majority of the bulk polymer remaining intact. Conversely, consistent with the rheological analysis, degradation of **CEPU1** and **CEPU2** with TBAF resulted in significant changes in the bulk polymer properties. Decreases in the UTS, EB, YM, and MoT were all observed, with **CEPU2** experiencing the greatest loss in all mechanical properties; see Figure 10 and Table 3. This further demonstrates the increased ability for TBAF to initiate degradation of sulfone chain-extender **5** for solid-state apolar polyol-based samples when compared with exposure to aqueous NaOH.

**Table 3:** Effect of Solid-State Degradation on the Mechanical Properties of the CEPUs^a^

CEPU adhesive	base	UTS^b^ (MPa)	EB^c^ (ε)	Young’s modulus (MPa)	modulus of toughness (MJ m^–3^)
**CEPU1**		9.04 ± 0.5	5.36 ± 0.6	9.40 ± 0.1	35.73 ± 5.8
	NaOH	7.90 ± 0.4	5.62 ± 0.3	8.39 ± 0.2	32.25 ± 2.9
		–13 ± 1%	+5 ± 1%	–11 ± 0%	–10 ± 2%
	TBAF	6.34 ± 0.5	4.55 ± 0.4	5.64 ± 0.2	14.11 ± 1.9
		–30 ± 3%	–15 ± 2%	–40 ± 2%	–61 ± 13%
**CEPU2**		9.16 ± 0.6	7.70 ± 0.6	4.78 ± 0.1	46.78 ± 6.1
	NaOH	8.94 ± 0.7	8.23 ± 0.8	3.72 ± 0.1	41.36 ± 7.8
		–2 ± 0%	+7 ± 1%	–22 ± 1%	–12 ± 3%
	TBAF	3.33 ± 0.3	5.02 ± 0.6	2.06 ± 0.2	10.26 ± 1.7
		–64 ± 7%	–35 ± 5%	–57 ± 7%	–78 ± 16%

a The order of the data in the table for each entry is as follows: pristine CEPU; degraded CEPU after 30 min; % change after 30 min. The error shown is the standard deviation between the three repeats of each sample. The percentage error shown is the standard error between the pristine and degraded averages for each sample.

b UTS, ultimate tensile strength.

c EB, elongation at break.

### 2.4 Adhesion Studies

**Figure 11:**
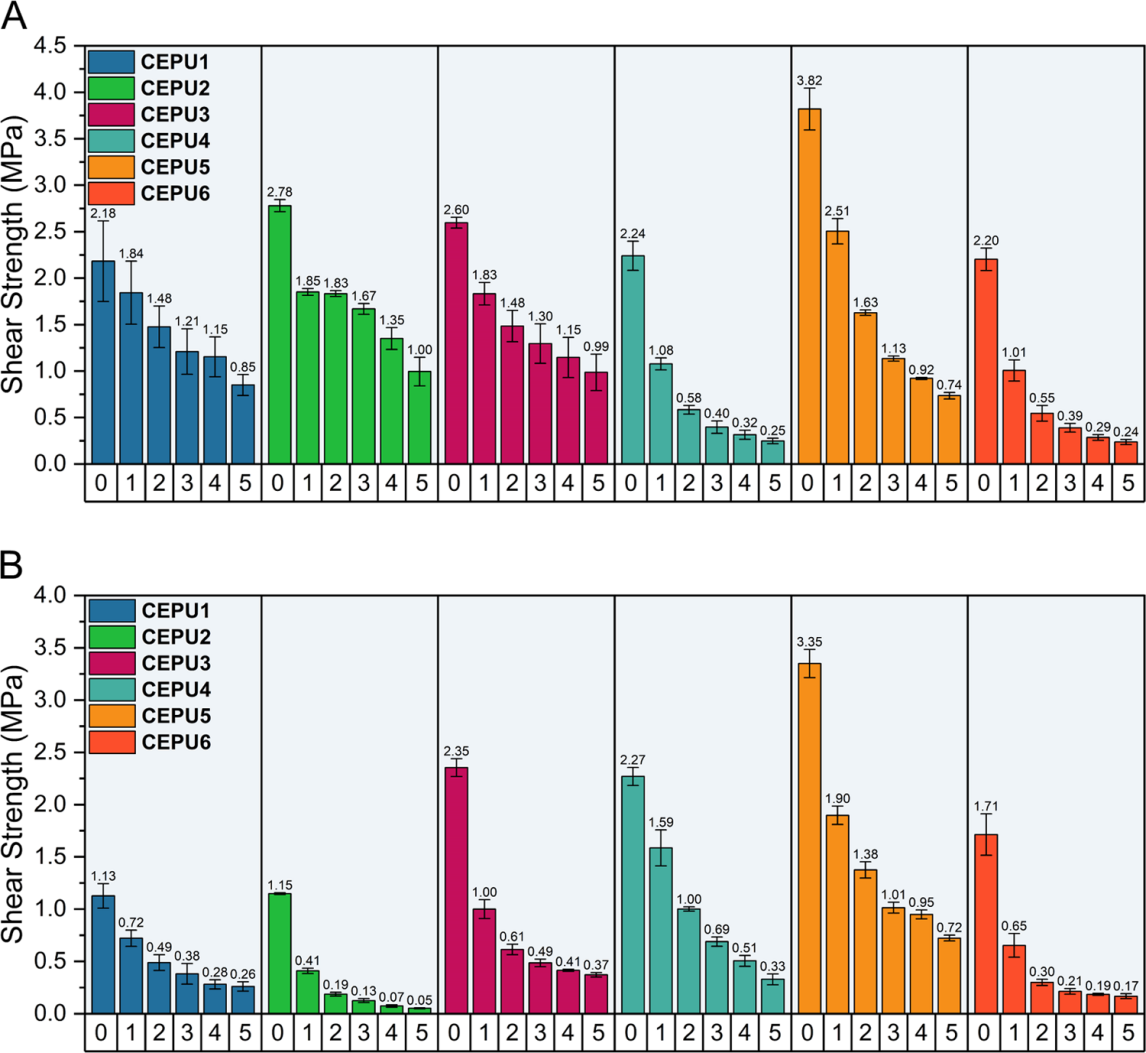
Shear strength of **CEPU1**–**CEPU6** over five readhesion cycles on (A) aluminum and (B) glass. The error shown is the standard deviation between the three repeats for each sample.

The hot melt adhesive capabilities of **CEPU1**–**CEPU6** were investigated using lap shear adhesion tests with aluminum and glass substrates. The CEPUs were adhered at 150 °C for 30 min, with each sample being tested in triplicate. Adhesion to aluminum provided the higher shear strengths for all CEPUs tested, with **CEPU5** exhibiting the highest shear strength of 3.82 ± 0.2 MPa. With the exception of **CEPU1**, the inclusion of the aromatic diisocyanate, 4,4′-MDI, increases the shear strength of the CEPU adhesive on both aluminum and glass when compared to their aliphatic counterparts, see Figure 11. To place this data in context, Wilker and co-workers explored a hydrolytically degradable catechol-poly(lactic acid) copolymer with a maximum shear strength of 2.6 MPa when adhered to aluminum, making it comparable to Elmer’s glue (3.0 MPa) and Gorilla glue (2.8 MPa).^27^ Similarly, catechol-functionalized polymers by Du and Li and co-workers undergo UV-triggered degradation with shear strengths of up to 0.5 MPa on glass being observed.^81^ Kihara and co-workers developed a series of cured epoxy resins which undergo oxidative degradation, achieving shear strengths up to 1.5 MPa on aluminum.^82^

To investigate the reusability of these CEPUs as adhesives, readhesion of **CEPU1**–**CEPU6** on both aluminum and glass substrates was conducted, see Figure 11 and Table 4. All CEPUs exhibited increasing cohesive failure over the 5 times of cycling, resulting in a decrease in the shear strength required for debonding. Over 5 readhesion cycles on aluminum, **CEPU1** exhibited the smallest %loss in shear strength of 61 ± 15%, from 2.18 ± 0.4 to 0.85 ± 0.1 MPa, with **CEPU4** exhibiting the greatest %loss in shear strength when adhered to glass, 95 ± 9% from 2.27 ± 0.1 to 0.33 ± 0.1 MPa (Tables S23 and S24). GPC analysis of **CEPU3** was conducted post 5 readhesion cycles on glass, and an 85 ± 4% reduction in molecular weight, from *M*_w_ = 181,200 ± 1500 g mol^–1^ to *M*_w_ = 27,000 ± 1100 g mol^–1^, was observed resulting from chain scission during the debonding process (Figure S134 and Table S25).

**Table 4:** Percentage Loss in Shear Strength of **CEPU1**–**CEPU6** over Five Readhesion Cycles to Aluminum and Glass^a^

CEPU adhesive	% loss in shear strength aluminum	% loss in shear strength glass
**CEPU1**	61 ± 15%	77 ± 16%
**CEPU2**	62 ± 12%	84 ± 6%
**CEPU3**	81 ± 6%	78 ± 4%
**CEPU4**	64 ± 10%	95 ± 9%
**CEPU5**	89 ± 12%	86 ± 14%
**CEPU6**	89 ± 12%	90 ± 16%

a The percentage error shown is the standard error between the pristine and degraded averages for each sample.

**Figure 12:**
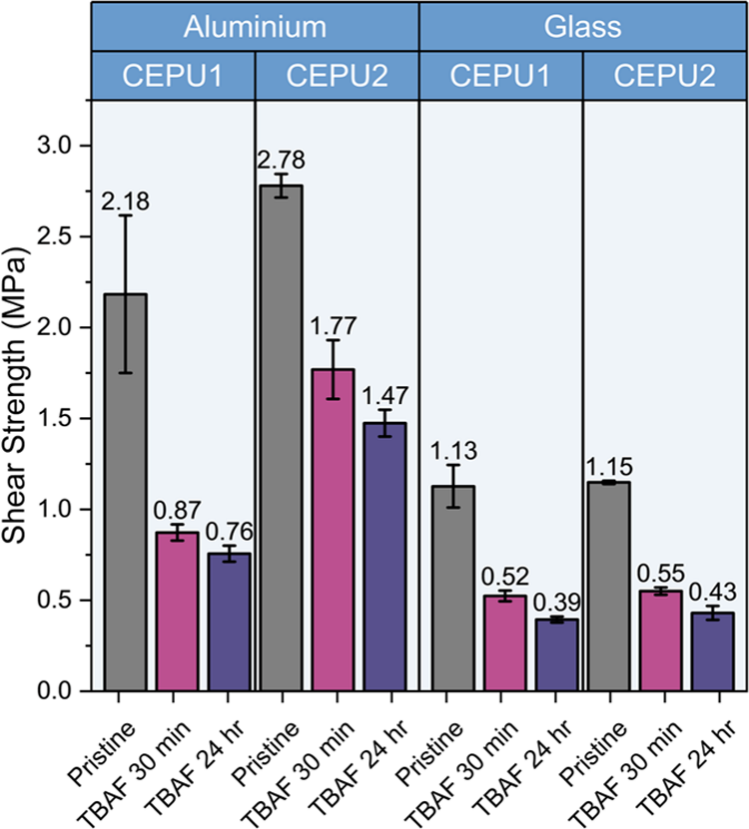
Shear strength of **CEPU1** and **CEPU2** on aluminum and glass after exposure to 1 M TBAF in acetone for 30 min and 24 h.

The stimuli-triggered debond-on-demand properties of **CEPU1** and **CEPU2** were investigated through exposure of lap shear samples to 1 M TBAF in acetone for 30 min or 24 h post adhesion. The adhered lap shear samples were first submerged in 1 M TBAF at room temperature for 30 min or 24 h, then washed with acetone and dried at room temperature under vacuum before being subject to tensile testing, the full experimental procedure can be found in the Supporting Information. After only 30 min **CEPU1** exhibited the greatest loss in shear strength for both aluminum and glass substrates, **CEPU1** 60 ± 12 and 54 ± 6%, respectively; **CEPU2** 36 ± 3 and 52 ± 2%, respectively, see Figure 12 and Table S26. Extension of the degradation time to 24 h only resulted in a 5–11% decrease in the shear strength; this marginal decrease in the shear strength results from the slow diffusion of the base though the bulk polymer. Lap shear samples of **CEPU3** and **CEPU4** on aluminum underwent the same TBAF exposure as **CEPU1** and **CEPU2** and experienced no loss in shear strength after 24 h, see Figures S143 and S144 and Table S27, highlighting that the loss in shear strength experienced by **CEPU1** and **CEPU2** results from the degradation of the sulfone chain-extender **5**. The glass lap shear sample for **CEPU1** exposed to TBAF for 24 h was analyzed via GPC after the adhesion test; this showed a 62 ± 10% drop in molecular weight, from *M*_n_ = 30,100 ± 300 g mol^–1^ to *M*_n_ = 11,400 ± 1800 g mol^–1^. This data exemplifies that the partial retention of shear strength results from the incomplete degradation of the CEPUs. In a comparable TBAF degradable CEPU composed of the same polyol and diisocyanate as **CEPU1**, a shear strength loss of only 41% after 24 h was observed,^47^ compared to a 60% loss after only 30 min with the use of chain-extender **5**.

## 3 Conclusions

Many conventional routes to achieve degradable polymeric adhesives within the literature require the design and synthesis of degradable chain-extenders or monomers. However, in this paper, we have shown that a commercially available diol, 2,2′-sulfonyldiethanol, **5**, provides a direct route to such degradable materials. Through model small molecules, **5** has been shown to undergo self-immolation upon exposure to both NaOD and TBAF. The incorporation of this sulfone diol into linear CEPU adhesives successfully afforded “debond-on-demand” behavior. GPC and ^1^H NMR spectroscopic studies confirm that **CEPU1** and **CEPU2** degrade to the prepolymer units in solution, and partial degradation occurs in the solid state on the addition of base. Tensile and rheological analysis shows that degradation initiated by TBAF brings about greater losses in mechanical and viscoelastic properties when compared to exposure of the polymer to NaOH. The readhesion capabilities of the CEPUs were investigated on both aluminum and glass substrates. Over 5 readhesion cycles, **CEPU1** exhibited the lowest %loss in shear strength of 61% when adhered to aluminum. The base-triggered “debond-on-demand” characteristics of **CEPU1** and **CEPU2** were investigated with TBAF, after only 30 min, a 60% reduction in shear strength is observed when **CEPU1** is adhered to aluminum.

## 4 Experimental Section

### 4.1 Materials

Krasol HLBH-P2000 was kindly provided by Total Cray Valley for this study. 2,2′-Sulfonyldiethanol (SDE) was purchased from Fluorochem and dried by azeotropic distillation *in vacuo* with toluene and then dried over phosphorus pentoxide prior to use. Tetrahydrofuran (THF) and acetonitrile (MeCN) were dried prior to use using an MBRAUN SP7 system fitted with activated alumina columns. All other reagents and solvents were purchased from Sigma-Aldrich, Fisher Scientific, Fluorochem, and Tokyo Chemical Industry and used as received.

### 4.2 Characterization

^1^H NMR and ^13^C{H} NMR spectra were recorded on either a Bruker Nanobay 400 or a Bruker DPX 400 spectrometer operating at 400 MHz for ^1^H NMR or 100 MHz for ^13^C{H} NMR, respectively. The data were processed using MestReNova Version 14.2.1-27684. Samples for NMR spectroscopic analysis were prepared in MeCN-*d*_3_ and THF-*d*_8_, and dissolution of the samples was aided by gentle heating. Chemical shifts (δ) are reported in parts per million relative to the residual solvent resonance (δ 1.94 ppm) for MeCN-*d*_3_ and (δ 3.58 ppm) for THF-*d*_8_ in ^1^H NMR. Infrared (IR) spectroscopic analysis was carried out using a PerkinElmer 100 FTIR (Fourier Transform Infrared) instrument with a diamond-ATR sampling accessory. Mass spectrometry (MS) was conducted using a Thermo Scientific LTQ-Orgitrap-XL Fourier Transform Mass Spectrometer (FTMS). The sample was introduced by an Agilent 1100 HPLC, and sample ionization was achieved by electrospray ionization (ESI). Melting points were recorded using Stuart MP10 melting point apparatus and are uncorrected. Gel permeation chromatography (GPC) analysis was conducted on an Agilent Technologies 1260 Infinity system using HPLC-grade THF at a flow rate of 1.0 mL min^–1^, calibration was achieved using a series of near monodisperse polystyrene standards, and samples were prepared at a concentration of 1 mg mL^–1^. Thermogravimetric analysis (TGA) was carried out on a TA Instruments TGA Q50 instrument with aluminum Tzero pans. The sample was heated from 20 to 550 °C at 10 °C min^–1^ under nitrogen gas at a flow rate of 100 mL min^–1^. Differential scanning calorimetry (DSC) measurements were performed on a TA Instruments DSC Q2000 adapted with a TA Refrigerated Cooling System 90, using aluminum TA Tzero pans and lids from −80 to 200 °C with a heating and cooling rate of 10 °C min^–1^. Rheological measurements were performed on a Malvern Panalytical Kinexus Lab+ instrument fitted with a Peltier plate cartridge and 8 mm parallel plate geometry and analyzed using rSpace Kinexus v1.76.2398 software. Tensile tests were carried out using a Thümler Z3-X1200 tensometer at a rate of 10 mm min^–1^ with a 1 kN load cell and THSSD-2021 software. The modulus of toughness was calculated by integrating the recorded plot to give the area under the curve. The trapezium rule was applied to calculate the area between zero strain and strain at break for each sample. The error reported is the standard deviation of the three repeats for each sample.

Crystals of **1**–**4** were mounted under Paratone-N oil and flash cooled to 100 K under nitrogen in an Oxford Cryosystems Cryostream. Single-crystal X-ray intensity data were collected using a Rigaku XtaLAB Synergy diffractometer (Cu Kα radiation (λ = 1.54184 Å)). The data were reduced within the CrysAlisPro software.^83^ The structures were solved using the program Superflip,^84^ and all nonhydrogen atoms were located. Least-squares refinement against *F* was carried out using the *CRYSTALS* suite of programs,^85^ The nonhydrogen atoms were refined anisotropically. All of the hydrogen atoms were located in the difference Fourier maps. The positions of the hydrogen atoms attached to nitrogen were refined with a *U*_iso_ of ca. 1.2–1.5 times the value of *U*_eq_ of the parent N atom. The hydrogen atoms attached to carbon were placed geometrically with a C–H distance of 0.95 Å and a *U*_iso_ of ca. 1.2–1.5 times the value of *U*_eq_ of the parent C atom, and the positions were refined with riding constraints.

SAXS/WAXS experiments were conducted on I22 beamline at Diamond Light Source (Harwell, U.K.).^86^ Samples were mounted in modified DSC pans in a Linkam DSC stage for temperature control. SAXS data was collected with a Pilatus P3-2 M detector, and WAXS data was simultaneously collected with a Pilatus 3-2M-DLS-L detector. vt-SAXS/WAXS experiments were conducted from 20 to 200 °C with a heating and cooling rate of 10 °C min^–1^ with spectra collected at 5 °C intervals. SAXS data was reduced^87^ and azimuthally averaged to obtain the scattering intensity *I* as a function of magnitude of the scattering vector *q*, where *q* = 4π/λ sin(θ/2) [2*θ is the scattering angle and λ is the used X-ray wavelength (12.4 keV)] using the software package DAWN.^88^ All peaks were modeled according to a shape-independent broad peak function or a joint broad peak-broad peak function to obtain *q*_max_ provided by SASView.5.0.6. (www.sasview.org/).

*d*-spacing was calculated using the following equation:
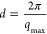


## Supporting Information Available

Experimental procedures, degradation protocols, and additional data from NMR, GPC, TGA, DSC, MS, single-crystal XRD, SAXS, WAXS, rheological experiments, and tensile testing (PDF)
