# Supplementary material for: Thermally and Base-Triggered “Debond-on-Demand” Chain-Extended Polyurethane Adhesives
Source: Macromolecules. 2025 Jan 2;58(1):681–96. doi: 10.1021/acs.macromol.4c02775 (PMC11741135; doi:10.1021/acs.macromol.4c02775)
Supplement: Supplementary file 1 — ma4c02775_si_001.pdf [file ma4c02775_si_001.pdf]

Supporting Information

Thermally and base triggered 'debond-on-demand' chain-extended polyurethane adhesives

Matthew J. Hyder,[a] Jessica Godleman,[b] Ann M. Chippindale,[a] James E. Hallett,[a] Thomas Zinn,[c] Josephine L. Harries,[b] Wayne Hayes[a,*]

[a]Department of Chemistry, University of Reading, Whiteknights, Reading, RG6 6DX, UK

[b]Domino UK Ltd, Trafalgar Way, Bar Hill, Cambridge, CB23 8TU, U.K.

[c]Diamond Light Source, Diamond Light Source Ltd, Harwell Science & Innovation Campus, Didcot, OX11 0DE, UK

*Corresponding author: email address w.c.hayes@reading.ac.uk

Contents

General Synthetic protocol for the synthesis of sulfonyl ethyl urethanes (**1** and **2**) 14

Synthesis of sulfonylbis(ethane-2,1-diyl) bis(phenylcarbamate) (**1**) 14

Synthesis of sulfonylbis(ethane-2,1-diyl) bis(cyclohexylcarbamate) (**2**) 15

General synthetic protocol for the synthesis of *N*-methylated sulfonyl ethyl urethanes (**3** and **4**) 15

Synthesis of sulfonylbis(ethane-2,1-diyl) bis(methyl(phenyl)carbamate) (**3**) 16

Synthesis of sulfonylbis(ethane-2,1-diyl) bis(cyclohexyl(methyl)carbamate) (**4**) 17

General synthetic protocol for CEPUs (**CEPU1**-**CEPU6**) 17

Synthesis of polymer **CEPU1** 18

Synthesis of polymer **CEPU2** 18

Synthesis of polymer **CEPU3** 19

Synthesis of polymer **CEPU4** 20

Synthesis of polymer **CEPU5** 20

Synthesis of polymer **CEPU6** 21

General synthetic protocol for methoxy terminated pre-polymers (**MeO-PU1** and **MeO-PU2**) 22

Synthesis of polymer **MeO-PU1** 22

Synthesis of polymer **MeO-PU2** 23

Protocol for casting CEPUs (**CEPU1**-**CEPU6**) and methoxy terminated pre-polymers (**MeO-PU1** and **MeO-PU2**) 24

Protocol for NMR solution state degradation 24

Protocol for GPC solution state degradation 24

Protocol for solid state degradation 25

Figure S 1 ^{1}H NMR spectrum of **1** (400 MHz, MeCN-d_3, 298 K). 26

Figure S 2 ^{13}C {H} NMR spectrum of **1** (100 MHz, MeCN-d_3, 298 K). 26

Figure S 3 ^{1}H NMR spectrum of **2** (400 MHz, MeCN-d_3, 298 K). 27

Figure S 4 ^{13}C {H} NMR spectrum of **2** (100 MHz, MeCN-d_3, 298 K). 27

Figure S 5 ^{1}H NMR spectrum of **3** (400 MHz, MeCN-d_3, 298 K). 28

Figure S 6 ^{13}C {H} NMR spectrum of **3** (100 MHz, MeCN-d_3, 298 K). 28

Figure S 7 ^{1}H NMR spectrum of **4** (400 MHz, MeCN-d_3, 298 K). 29

Figure S 8 ^{13}C {H} NMR spectrum of **4** (100 MHz, MeCN-d_3, 298 K). 29

Figure S 9 ^{1}H NMR spectrum of **CEPU1** (400 MHz, THF-d_8, 298 K). 30

Figure S 10 ^{13}C {H} NMR spectrum of **CEPU1** (100 MHz, THF-d_8, 298 K). 30

Figure S 11 ^{1}H NMR spectrum of **CEPU2** (400 MHz, THF-d_8, 298 K). 31

Figure S 12 ^{13}C {H} NMR spectrum of **CEPU2** (100 MHz, THF-d_8, 298 K). 31

Figure S 13 ^{1}H NMR spectrum of **CEPU3** (400 MHz, THF-d_8, 298 K). 32

Figure S 14 ^{13}C {H} NMR spectrum of **CEPU3** (100 MHz, THF-d_8, 298 K). 32

Figure S 15 ^{1}H NMR spectrum of **CEPU4** (400 MHz, THF-d_8, 298 K). 33

Figure S 16 ^{13}C {H} NMR spectrum of **CEPU4** (100 MHz, THF-d_8, 298 K). 33

Figure S 17 ^{1}H NMR spectrum of **CEPU5** (400 MHz, THF-d_8, 298 K). 34

Figure S 18 ^{13}C {H} NMR spectrum of **CEPU5** (100 MHz, THF-d_8, 298 K). 34

Figure S 19 ^{1}H NMR spectrum of **CEPU6** (400 MHz, THF-d_8, 298 K). 35

Figure S 20 ^{13}C {H} NMR spectrum of **CEPU6** (100 MHz, THF-d_8, 298 K). 35

Table S 1 Key ^{1}H NMR and ^{13}C NMR spectroscopic resonances for **CEPU1-CEPU6** in THF-d_8. 36

Figure S 21 ^{1}H NMR spectrum of **MeO-PU1** (400 MHz, THF-d_8, 298 K). 37

Figure S 22 ^{13}C {H} NMR spectrum of **MeO-PU1** (100 MHz, THF-d_8, 298 K). 37

Figure S 23 ^{1}H NMR spectrum of **MeO-PU2** (400 MHz, THF-d_8, 298 K). 38

Figure S 24 ^{13}C {H} NMR spectrum of **MeO-PU2** (100 MHz, THF-d_8, 298 K). 38

Figure S 25 The molecular structure of **1** obtained from single-crystal X-ray diffraction analysis. Thermal ellipsoids drawn at 50% probability. [symmetry code: (i) 1 – *x*, *y*, ½ – *z*]. 39

Table S 2 Crystallographic details for **1**. 39

Table S 3 Selected bond lengths (Å) and angles (°) in **1**. 40

Table S 4 Hydrogen-bond geometry (Å, °) in **1**. 40

Table S 5 π-π interactions (Å) in **1**. 40

Figure S 26 Packing diagram for compound **1**. 41

Table S 6 Crystallographic details for **2**. 41

Figure S 27 The asymmetric unit of **2** obtained from single-crystal X-ray diffraction analysis contains two distinct molecules (**A** and **B**). Atom labelling schemes are shown for each of the molecules. Thermal ellipsoids drawn at 50% probability. 42

Table S 7 Selected bond lengths (Å) and angles (°) in **2**. 43

Table S 8 Hydrogen-bond and close-contact geometry (Å, °) in **2**. 45

Figure S 28 Packing diagrams for compound **2**. 46

Figure S 29 The molecular structure of **3** obtained from single-crystal X-ray diffraction analysis. Thermal ellipsoids drawn at 50% probability. 47

Table S 9 Crystallographic details for **3**. 47

Table S 10 Selected bond lengths (Å) and angles (°) in **3**. 48

Table S 11 Hydrogen bond and close-contact geometry (Å, °) in **3**. 49

Table S 12 π-π interactions (Å) in **3**. 49

Figure S 30 Packing diagram for compound **3**. 49

Figure S 31 The molecular structure of **4** obtained from single-crystal X-ray diffraction analysis. Thermal ellipsoids drawn at 50% probability. [symmetry code: (i) 1 – *x*, 1 – *y*, *z*]. 50

Table S 13 Crystallographic details for **4**. 50

Table S 14 Selected bond lengths (Å) and angles (°) in **4**. 51

Table S 15 Hydrogen-bond and close-contact geometry (Å, °) in **4**. 51

Figure S 32 Degradation kinetics of model urethanes **1**-**4**. 52

Figure S 33 ^{1}H NMR spectra recorded overtime following the addition of 5 molar equiv. of 40 wt.% NaOD in D_2O to a 10 mg mL^{-1} solution of model urethane **1**. 52

Figure S 34 ^{1}H NMR spectra recorded overtime following the addition of 5 molar equiv. of 1 M TBAF in THF to a 10 mg mL^{-1} solution of model urethane **1**. 53

Table S 16 Degradation kinetics of model urethanes **1**. 55

Figure S 35 ^{1}H NMR spectra recorded overtime following the addition of 5 molar equiv. of 40 wt.% NaOD in D_2O to a 10 mg mL^{-1} solution of model urethane **2**. 54

Figure S 36 ^{1}H NMR spectra recorded overtime following the addition of 5 molar equiv. of 1 M TBAF in THF to a 10 mg mL^{-1} solution of model urethane **2**. 55

Table S 17 Degradation kinetics of model urethanes **2**. 55

Figure S 37 ^{1}H NMR spectra recorded overtime following the addition of 5 molar equiv. of 40 wt.% NaOD in D_2O to a 10 mg mL^{-1} solution of model urethane **3**. 56

Figure S 38 ^{1}H NMR spectra recorded overtime following the addition of 5 molar equiv. of 1 M TBAF in THF to a 10 mg mL^{-1} solution of model urethane **3**. 57

Table S 18 Degradation kinetics of model urethanes **3**. 57

Figure S 39 ^{1}H NMR spectra recorded overtime following the addition of 5 molar equiv. of 40 wt.% NaOD in D_2O to a 10 mg mL^{-1} solution of model urethane **4**. 58

Figure S 40 ^{1}H NMR spectra recorded overtime following the addition of 5 molar equiv. of 1 M TBAF in THF to a 10 mg mL^{-1} solution of model urethane **4**. 59

Table S 19 Degradation kinetics of model urethanes **4**. 59

Figure S 41 ^{1}H NMR spectrum of divinyl sulfone (400 MHz, MeCN-d_3, 298 K). 60

Figure S 42 ^{13}C {H} NMR spectrum of divinyl sulfone (100 MHz, MeCN-d_3, 298 K). 60

Figure S 43 LC-MS of the solution state degradation of **1**. 61

Figure S 44 Structures of secondary degradation products from the NaOD degradation of **1** observed in LC-MS. 62

Figure S 45 LC-MS of the solution state degradation of **1**. 63

Figure S 46 Structures of secondary degradation products from the TBAF degradation of **1** observed in LC-MS. 63

Figure S 47 LC-MS of the solution state degradation of **2**. 64

Figure S 48 Structures of secondary degradation products from the NaOD degradation of **2** observed in LC-MS. 65

Figure S 49 GPC eluogram of CEPU1 in THF. 66

Figure S 50 GPC eluogram of CEPU2 in THF. 66

Figure S 51 GPC eluogram of CEPU3 in THF. 67

Figure S 52 GPC eluogram of CEPU4 in THF. 67

Figure S 53 GPC eluogram of CEPU5 in THF. 68

Figure S 54 GPC eluogram of CEPU6 in THF. 68

Figure S 55 GPC eluogram of MeO-PU1 in THF. 69

Figure S 56 GPC eluogram of MeO-PU2 in THF. 69

Figure S 57 DSC thermogram of **CEPU1**. 70

Figure S 58 DSC thermogram of **CEPU2**. 70

Figure S 59 DSC thermogram of **CEPU3**. 71

Figure S 60 DSC thermogram of **CEPU4**. 71

Figure S 61 DSC thermogram of **CEPU5**. 72

Figure S 62 DSC thermogram of **CEPU6**. 72

Figure S 63 DSC thermogram of **MeO-PU1**. 73

Figure S 64 DSC thermogram of **MeO-PU2**. 73

Figure S 65 TGA thermogram of **CEPU1** at 10 °C min^{-1} under nitrogen. 74

Figure S 66 TGA thermogram of **CEPU2** at 10 °C min^{-1} under nitrogen. 74

Figure S 67 TGA thermogram of **CEPU3** at 10 °C min^{-1} under nitrogen. 75

Figure S 68 TGA thermogram of **CEPU4** at 10 °C min^{-1} under nitrogen. 75

Figure S 69 TGA thermogram of **CEPU5** at 10 °C min^{-1} under nitrogen. 76

Figure S 70 TGA thermogram of **CEPU6** at 10 °C min^{-1} under nitrogen. 76

Figure S 71 TGA thermogram of **MeO-PU1** at 10 °C min^{-1} under nitrogen......................... 77

Figure S 72 TGA thermogram of **MeO-PU2** at 10 °C min^{-1} under nitrogen......................... 77

Figure S 73 SAXS profile of **CEPU1** and corresponding fitline at 20 °C............................. 78

Figure S 74 SAXS profile of **CEPU2** and corresponding fitline at 20 °C............................. 78

Figure S 75 SAXS profile of **CEPU3** and corresponding fitline at 20 °C............................. 78

Figure S 76 SAXS profile of **CEPU4** and corresponding fitline at 20 °C............................. 79

Figure S 77 SAXS profile of **CEPU5** and corresponding fitline at 20 °C............................. 79

Figure S 78 SAXS profile of **CEPU6** and corresponding fitline at 20 °C............................. 79

Table S 20 q_{max} and corresponding *d*-spacing for **CEPU1-CEPU6** at 20 °C........................ 80

Figure S 79 SAXS fitting residuals for **CPEU1** at 20 °C.. 80

Figure S 80 SAXS fitting residuals for **CPEU2** at 20 °C.. 80

Figure S 81 SAXS fitting residuals for **CPEU3** at 20 °C. 81

Figure S 82 SAXS fitting residuals for **CPEU4** at 20 °C. 81

Figure S 83 SAXS fitting residuals for **CPEU5** at 20 °C. 81

Figure S 84 SAXS fitting residuals for **CPEU6** at 20 °C. 82

Figure S 85 VT-SAXS (**A** and **B**) and VT-WAXS (**C** and **D**) profiles of **CEPU1** 83

Figure S 86 VT-SAXS (**A** and **B**) and VT-WAXS (**C** and **D**) profiles of **CEPU2** 83

Figure S 87 Temperature sweep analysis of **CEPU1** 84

Figure S 88 Temperature sweep analysis of **CEPU2** 84

Figure S 89 Temperature sweep analysis of **CEPU3** 85

Figure S 90 Temperature sweep analysis of **CEPU4** 85

Figure S 91 Temperature sweep analysis of **CEPU5** 86

Figure S 92 Temperature sweep analysis of **CEPU6** 86

Figure S 93 Temperature sweep analysis of **MeO-PU1** 87

Figure S 94 Temperature sweep analysis of **MeO-PU2** 87

Figure S 95 ^{1}H NMR spectra showing the solution degradation of **CEPU1** with 40 wt.% NaOD in D_2O, (400 MHz, THF-d_8) 88

Figure S 96 ^{13}C {H} NMR spectra showing the solution degradation of **CEPU1** with 40 wt.% NaOD in D_2O, (400 MHz, THF-d_8). 89

Figure S 97 ^{13}C {H} NMR spectra showing the solution degradation of **CEPU1** with 1M TBAF in acetone, (400 MHz, THF-d_8) 90

Figure S 98 ^{1}H NMR spectra showing the solution degradation of **CEPU2** with 40 wt.% NaOD in D_2O, (400 MHz, THF-d_8) 91

Figure S 99 ^{13}C {H} NMR spectra showing the solution degradation of **CEPU2** with 40 wt.% NaOD in D_2O, (400 MHz, THF-d_8). 92

Figure S 100 ^{1}H NMR spectra showing the solution degradation of **CEPU2** with 1M TBAF in acetone, (400 MHz, THF-d_8). 93

Figure S 101 ^{13}C {H} NMR spectra showing the solution degradation of **CEPU2** with 1M TBAF in acetone, (400 MHz, THF-d_8).. 94

Figure S 102 ^{1}H NMR spectrum of divinyl sulfone (400 MHz, THF-d_8, 298 K). 95

Figure S 103 ^{13}C {H} NMR spectrum of divinyl sulfone (400 MHz, THF-d_8, 298 K). 95

Figure S 104 GPC eluogram of **CEPU3** in THF as a pristine sample and 30 min, 24 hr, and 48 hr post addition of TBAF.. 96

Figure S 105 GPC eluogram of **CEPU4** in THF as a pristine sample and 30 min, 24 hr, and 48 hr post addition of TBAF.. 96

Figure S 106 GPC eluogram of **CEPU5** in THF as a pristine sample and 30 min, 24 hr, and 48 hr post addition of TBAF.. 97

Figure S 107 GPC eluogram of **CEPU6** in THF as a pristine sample and 30 min, 24 hr, and 48 hr post addition of TBAF.. 97

Table S 21 M_n and M_w of **CEPU1**-**CEPU6** as pristine samples and 30 min, 24 hr, and 48 hr post addition of TBAF acquired from a THF GPC.. 98

Figure S 108 Solid state degradation of **CEPU2** film using 1 M TBAF in acetone at room temperature over time. 98

Figure S 109 DSC thermogram of **CEPU1** after 24 hours of solid state degradation with 40 wt.% $NaOH_{(aq)}$. 99

Figure S 110 DSC thermogram of **CEPU1** after 24 hours of solid state degradation with 1 M TBAF. 99

Figure S 111 DSC thermogram of **CEPU2** after 24 hours of solid state degradation with 40 wt.% $NaOH_{(aq)}$. 100

Figure S 112 DSC thermogram of **CEPU2** after 24 hours of solid state degradation with 1 M TBAF. 100

Table S 22 Thermal properties of **CEPU1** and **CEPU2** post degradation and the thermal properties of **MeO-PU1** and **MeO-PU2**........ 101

Figure S 113 Temperature sweep analysis of solid state degraded CEPUs using 40 wt.% $NaOH_{(aq)}$ or 1M TBAF........ 102

Figure S 114 Temperature sweep analysis of **CEPU1** after 30 minutes of solid state degradation with 40 wt.% $NaOH_{(aq)}$. 103

Figure S 115 Temperature sweep analysis of **CEPU1** after 24 hours of solid state degradation with 40 wt.% $NaOH_{(aq)}$........ 103

Figure S 116 Temperature sweep analysis of **CEPU1** after 30 minutes of solid state degradation with 1 M TBAF........ 104

Figure S 117 Temperature sweep analysis of **CEPU1** after 24 hours of solid state degradation with 1 M TBAF. 104

Figure S 118 Temperature sweep analysis of **CEPU2** after 30 minutes of solid state degradation with 40 wt.% $NaOH_{(aq)}$. 105

Figure S 119 Temperature sweep analysis of **CEPU2** after 24 hours of solid state degradation with 40 wt.% $NaOH_{(aq)}$........ 105

Figure S 120 Temperature sweep analysis of **CEPU2** after 30 minutes of solid state degradation with 1 M TBAF........ 106

Figure S 121 Temperature sweep analysis of **CEPU2** after 24 hours of solid state degradation with 1 M TBAF. 106

Figure S 122 Representative stress-strain curves of the adhered **CEPU1** to aluminium over five re-adhesion cycles........ 107

Figure S 123 Representative stress-strain curves of the adhered **CEPU2** to aluminium over five re-adhesion cycles.. 107

Figure S 124 Representative stress-strain curves of the adhered **CEPU3** to aluminium over five re-adhesion cycles.. 108

Figure S 125 Representative stress-strain curves of the adhered **CEPU4** to aluminium over five re-adhesion cycles.. 108

Figure S 126 Representative stress-strain curves of the adhered **CEPU5** to aluminium over five re-adhesion cycles.. 109

Figure S 127 Representative stress-strain curves of the adhered **CEPU6** to aluminium over five re-adhesion cycles.. 109

Figure S 128 Representative stress-strain curves of the adhered **CEPU1** to glass over five re-adhesion cycles. 110

Figure S 129 Representative stress-strain curves of the adhered **CEPU2** to glass over five re-adhesion cycles. 110

Figure S 130 Representative stress-strain curves of the adhered **CEPU3** to glass over five re-adhesion cycles. 111

Figure S 131 Representative stress-strain curves of the adhered **CEPU4** to glass over five re-adhesion cycles. 111

Figure S 132 Representative stress-strain curves of the adhered **CEPU5** to glass over five re-adhesion cycles. 112

Figure S 133 Representative stress-strain curves of the adhered **CEPU6** to glass over five re-adhesion cycles. 112

Table S 23 Shear strength of **CEPU1**-**CEPU6** over five re-adhesion cycles on Aluminium. The error shown is the standard deviation between the three repeats of each sample............. 113

Table S 24 Shear strength of **CEPU1**-**CEPU6** over five re-adhesion cycles on Glass. The error shown is the standard deviation between the three repeats of each sample. 113

Figure S 134 GPC eluogram of **CEPU3** in THF after five re-adhesion cycles to glass. 114

Table S 25 GPC molecular weight and dispersity data for **CEPU3** after five re-adhesion cycles to glass 114

Figure S 135 Representative stress-strain curve of the adhered **CEPU1** to aluminium after 30 minutes of solid-state degradation with 1 M TBAF in acetone at room temperature. 115

Figure S 136 Representative stress-strain curve of the adhered **CEPU1** to aluminium after 24 hours of solid-state degradation with 1 M TBAF in acetone at room temperature. 115

Figure S 137 Representative stress-strain curve of the adhered **CEPU1** to glass after 30 minutes of solid-state degradation with 1 M TBAF in acetone at room temperature. 116

Figure S 138 Representative stress-strain curve of the adhered **CEPU1** to glass after 24 hours of solid-state degradation with 1 M TBAF in acetone at room temperature....................... 116

Figure S 139 Representative stress-strain curve of the adhered **CEPU2** to aluminium after 30 minutes of solid-state degradation with 1 M TBAF in acetone at room temperature. 117

Figure S 140 Representative stress-strain curve of the adhered **CEPU2** to aluminium after 24 hours of solid-state degradation with 1 M TBAF in acetone at room temperature. 117

Figure S 141 Representative stress-strain curve of the adhered **CEPU2** to glass after 30 minutes of solid-state degradation with 1 M TBAF in acetone at room temperature. 118

Figure S 142 Representative stress-strain curve of the adhered **CEPU2** to glass after 24 hours of solid-state degradation with 1 M TBAF in acetone at room temperature....................... 118

Table S 26 Shear strength of **CEPU1** and **CEPU2** on aluminium and glass after exposure to 1 M TBAF in acetone for 30 minutes and 24 hours. 119

Figure S 143 Representative stress-strain curve of the adhered **CEPU3** to aluminium after 24 hours of solid-state degradation with 1 M TBAF in acetone at room temperature. 119

Figure S 144 Representative stress-strain curve of the adhered **CEPU4** to aluminium after 24 hours of solid-state degradation with 1 M TBAF in acetone at room temperature. 120

Table S 27 Shear strength of **CEPU3** and **CEPU4** on aluminium after exposure to 1 M TBAF in acetone for 24 hours. 120

Figure S 145 GPC eluograms of **CEPU1** in THF after 24 hours of solid-state degradation with 1 M TBAF in acetone at room temperature when adhered to glass. 121

Table S 28 GPC molecular weight and dispersity data for adhered **CEPU1** to glass after 24 hours of solid-state degradation with 1 M TBAF in acetone at room temperature. 121

General Synthetic protocol for the synthesis of sulfonyl ethyl urethanes (**1** and **2**)

2,2’-Sulfonyldiethanol (1 equiv.) was dissolved in anhydrous THF (30 mL) to which isocyanate (2 equiv.) was added dropwise at 0 °C under an argon atmosphere before being heated at 40 °C. The progress of the reaction was monitored via IR spectroscopy and once the isocyanate absorbance band at 2275-2250 cm^{-1} ($\nu N{=}C{=}O_{stretch}$) was not evident the reaction was deemed to have reached completion. The solvent was removed *in vacuo* and the crude product was recrystallised from a minimum amount of boiling ethanol. The product was isolated as a colourless crystalline solid.

Synthesis of sulfonylbis(ethane-2,1-diyl) bis(phenylcarbamate) (**1**)

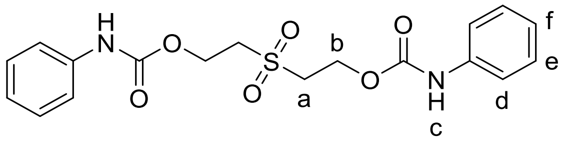

Sulfonylbis(ethane-2,1-diyl) bis(phenylcarbamate) was synthesised following the general synthetic protocol described above for sulfonyl ethyl urethanes. Sulfonylbis(ethane-2,1-diyl) bis(phenylcarbamate) (2.31 g, 5.90 mmol, 91 %) was obtained as a colourless crystalline solid from 2,2’-sulfonyldiethanol (1.00 g, 6.49 mmol, 1 equiv.), and phenyl isocyanate (1.55 g, 13.05 mmol, 2 equiv.). Mp 160-161 °C; FTIR ATR (cm^{-1}): 3338 (νN-H), 3060 ($\nu C\text{-}H_{aromatic}$), 3045 ($\nu C\text{-}H_{aromatic}$), 2973 ($\nu C\text{-}H_{alkyl}$), 2932 ($\nu C\text{-}H_{alkyl}$), 1694 ($\nu C{=}O_{urethane}$), 1596 ($\nu C{=}C_{stretch}$), 1320 ($\nu S{=}O_{stretch}$); ^1H NMR (400 MHz, CD_3CN) δ 7.97 (s, 2H, H_c), 7.43 – 7.37 (m, 4H, H_d), 7.30 – 7.24 (m, 4H, H_e), 7.07 – 7.01 (m, 2H, H_f), 4.52 (t, *J* = 5.7 Hz, 4H, H_b), 3.49 (t, *J* = 5.7 Hz, 4H, H_a); ^{13}C NMR (100 MHz, CD_3CN) δ 154.1, 139.3, 129.9, 124.4, 119.8, 59.6, 54.6; FTMS (ESI) *m/z* [M + Na^+] calculated for $C_{18}H_{20}N_2NaO_6S$ = 415.0934, found = 415.0899.

Synthesis of sulfonylbis(ethane-2,1-diyl) bis(cyclohexylcarbamate) (**2**)

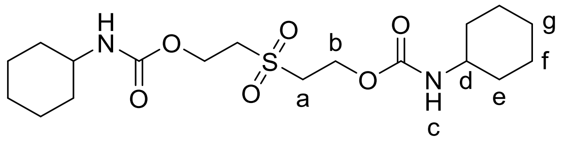

Sulfonylbis(ethane-2,1-diyl) bis(cyclohexylcarbamate) was synthesised following the general synthetic protocol described above for sulfonyl ethyl urethanes. Sulfonylbis(ethane-2,1-diyl) bis(cyclohexylcarbamate) (1.18 g, 2.92 mmol, 86 %) was obtained as a colourless crystalline solid from 2,2'-sulfonyldiethanol (0.53 g, 3.41 mmol, 1 equiv.), and cyclohexyl isocyanate (0.85 g, 6.82 mmol, 2 equiv.). Mp 146-147 °C; FTIR ATR (cm^{-1}): 3316 (vN-H), 2933 (vC-H_{alkyl}), 2853 (vC-H_{alkyl}), 1687 (vC=$O_{urethane}$), 1317 (vS=$O_{stretch}$); ^{1}H NMR (400 MHz, CD_3CN) δ 5.87 (d, *J* = 8.0 Hz, 2H, H_c), 4.32 (t, *J* = 5.7 Hz, 4H, H_b), 3.35 (t, *J* = 5.7 Hz, 6H, H_a + H_d), 1.89 – 1.79 (m, 4H), 1.76 – 1.66 (m, 4H), 1.64 – 1.52 (m, 2H), 1.38 – 1.25 (m, 4H), 1.25 – 1.10 (m, 6H); ^{13}C NMR (100 MHz, CD_3CN) δ 155.9, 59.2, 54.8, 50.9, 33.8, 26.2, 25.6; FTMS (ESI) *m/z* [M + H^+] calculated for $C_{18}H_{33}N_2O_6S$ = 405.2054, found = 405.2020.

General synthetic protocol for the synthesis of *N*-methylated sulfonyl ethyl urethanes (**3** and **4**)

2,2'-Sulfonyldiethanol (1 equiv.) was dissolved in mixture of anhydrous THF (10 mL) and anhydrous acetonitrile (10 mL) and added dropwise to a solution of 15 wt.% phosgene in toluene (4 equiv.) at -10 °C under an argon atmosphere. The solution was allowed to react at room temperature for 3 hours, the solvent was then removed *in vacuo*. The resulting chloroformate was re-dissolved in anhydrous toluene (15 mL) and added dropwise to a solution of *N*-methylamine (2 equiv.) in anhydrous toluene (15 mL) at 0 °C under an argon atmosphere before being refluxed at 120 °C for 16 hours. The solution was allowed to cool to room temperature, filtered then the solution was washed with water (3 x 30 mL), then 0.5 M citric acid (3 x 30 mL), then water (30 mL). The organic phase was dried with $MgSO_4$ and the

solvent was removed *in vacuo*. The resulting oil was cooled in ice to induce crystallisation, the crude product was recrystallised from a minimum amount of boiling ethanol. The product was isolated as a colourless crystalline solid.

Synthesis of sulfonylbis(ethane-2,1-diyl) bis(methyl(phenyl)carbamate) (**3**)

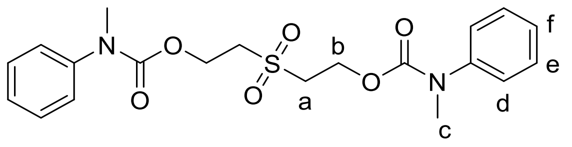

Sulfonylbis(ethane-2,1-diyl) bis(methyl(phenyl)carbamate) was synthesised following the general synthetic protocol described above for *N*-methylated sulfonyl ethyl urethanes. Sulfonylbis(ethane-2,1-diyl) bis(methyl(phenyl)carbamate) (1.62 g, 3.85 mmol, 40 %) was obtained as a colourless crystalline solid from 2,2'-sulfonyldiethanol (1.50 g, 9.73 mmol, 1 equiv.), 15 wt.% phosgene in toluene (28 mL, 39.24 mmol, 4 equiv.), and *N*-methylaniline (2.09 g, 19.50 mmol, 2 equiv.). Mp 110-111 °C; FTIR ATR (cm^{-1}): 3062 ($\nu C\text{-}H_{aromatic}$), 2965 ($\nu C\text{-}H_{alkyl}$), 2939 ($\nu C\text{-}H_{alkyl}$), 2936 ($\nu C\text{-}H_{alkyl}$), 2896 ($\nu C\text{-}H_{alkyl}$), 1701 ($\nu C{=}O_{urethane}$), 1595 ($\nu C{=}C_{stretch}$), 1478 ($\nu C\text{-}H_{alkyl}$), 1453 ($\nu C\text{-}H_{methyl}$), 1344 ($\nu S{=}O_{stretch}$); ^1H NMR (400 MHz, CD_3CN) δ 7.38 (td, *J* = 7.3, 1.5 Hz, 4H, H_e), 7.30 – 7.21 (m, 6H, H_d + H_f), 4.19 (s, 4H, H_b), 3.23 (s, 6H, H_c), 2.86 (s, 4H, H_a); ^{13}C NMR (100 MHz, CD_3CN) δ 155.27, 144.1, 130.1, 127.62, 127.4, 60.0, 53.9, 38.4; FTMS (ESI) *m/z* [M + H^+] calculated for $C_{20}H_{25}N_2O_6S$ = 421.1428, found = 421.1387.

Synthesis of sulfonylbis(ethane-2,1-diyl) bis(cyclohexyl(methyl)carbamate) (**4**)

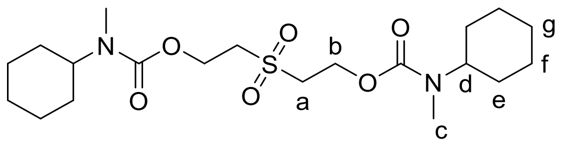

Sulfonylbis(ethane-2,1-diyl) bis(cyclohexyl(methyl)carbamate) was synthesised following the general synthetic protocol described above for *N*-methylated sulfonyl ethyl urethanes. Sulfonylbis(ethane-2,1-diyl) bis(cyclohexyl(methyl)carbamate) (1.25 g, 2.89 mmol, 30 %) was obtained as a colourless crystalline solid from 2,2'-sulfonyldiethanol (1.50 g, 9.73 mmol, 1 equiv.), 15 wt.% phosgene in toluene (28 mL, 39.24 mmol, 4 equiv.), and *N*-methylcyclohexylamine (2.20 g, 19.43 mmol, 2 equiv.). Mp 108-109 °C; FTIR ATR (cm^{-1}): 2937 ($vC\text{-}H_{alkyl}$), 2853 ($vC\text{-}H_{alkyl}$), 1688 ($vC{=}O_{urethane}$), 1475 ($vC\text{-}H_{alkyl}$), 1447 ($vC\text{-}H_{methyl}$), 1314 ($vS{=}O_{stretch}$); ^1H NMR (400 MHz, CD_3CN) δ 4.41 (t, *J* = 5.7 Hz, 4H, H_b), 3.95 – 3.74 (m, 2H, H_d), 3.39 (t, *J* = 5.7 Hz, 4H, H_a), 2.74 (s, 6H, H_c), 1.78 (d, *J* = 13.2 Hz, 4H), 1.61 (d, *J* = 13.2 Hz, 6H), 1.44 (qd, *J* = 12.1, 3.1 Hz, 4H), 1.37 – 1.21 (m, 4H), 1.09 (qt, *J* = 12.8, 3.6 Hz, 2H); ^{13}C NMR (100 MHz, CD_3CN) δ 155.9, 59.3, 56.1, 54.5, 30.6, 28.8, 26.5, 26.1; FTMS (ESI) *m/z* [M + H^+] calculated for $C_{20}H_{37}N_2O_6S$ = 433.2367, found = 433.2336.

General synthetic protocol for CEPUs (**CEPU1**-**CEPU6**)

Hydrogenated poly(butadiene) (Krasol HLBH-P 2000), molecular weight as supplied = 2100 g mol^{-1}, was dried under vacuum in the oven at 80 °C for 2 hours. In the bulk, Krasol HLBH-P 2000 (1.00 equiv.) was mixed with diisocyanate (2.05 equiv.) and dibutyltin dilaurate (10 drops, **CEPU2**, **CEPU4**, and **CEPU6** only) at 80 °C under argon for 3 hours with gentle stirring. The colourless pre-polymer obtained was dissolved in dry THF (40 mL) and the diol chain-extender (1.05 equiv.) was then added to the solution which was then brought to and maintained under reflux for 18 hours under argon. The chain-extended polymer solution was precipitated into ice cold methanol (3 x 1000 mL), the solid material filtered off and then dried *in vacuo*.

Synthesis of polymer **CEPU1**

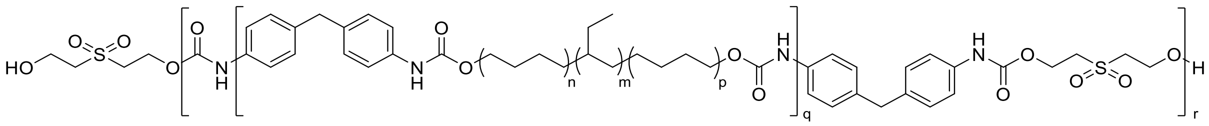

The synthesis was carried out according to the general synthetic protocol described above for CEPUs. The polymer was isolated as a transparent colourless elastomeric solid (6.66 g, 95 %) from Krasol HLBH-P 2000 (5.30 g, 2.52 mmol, 1.00 equiv.), 4,4'-methylenebis(phenyl isocyanate) (1.30 g, 5.19 mmol, 2.05 equiv.), and 2,2-sulfonyldiethanol (0.41 g, 2.66 mmol, 1.05 equiv.). T_g = -47.2 °C, T_m = 102.4 °C; FTIR ATR (cm^{-1}): 3332 ($vN\text{-}H_{stretch}$), 2959 ($vC\text{-}H_{alkyl}$), 2920 ($vC\text{-}H_{alkyl}$), 2852 ($vC\text{-}H_{alkyl}$), 1738 ($vC{=}O_{urethane}$), 1708 ($vC{=}O_{urethane}$), 1598 ($vC{=}C_{stretch}$), 1461 ($vC\text{-}H_{alkyl}$), 1318 ($vS{=}O_{stretch}$); ^{1}H NMR (400 MHz, THF-d_8) δ 8.82 (s, 1H), 8.54 (s, 1H), 7.35 (dd, *J* = 12.3, 7.7 Hz, 5H), 7.03 (d, *J* = 6.8 Hz, 3H), 4.50 (t, *J* = 6.4 Hz, 2H), 4.18 – 4.02 (m, 2H), 3.81 (d, *J* = 8.1 Hz, 2H), 3.64 (s, 1H), 3.43 (d, *J* = 6.4 Hz, 2H), 2.11 – 1.86 (m, 2H), 1.69 – 0.65 (m, 199H); ^{13}C NMR (100 MHz, THF-d_8) δ 154.5, 154.1, 138.9, 138.0, 137.2, 136.4, 136.3, 130.1, 130.0, 130.0, 129.9, 119.5, 119.1, 65.3, 63.6, 59.3, 54.5, 41.4, 40.3, 40.1, 39.7, 39.5, 39.0, 37.4, 34.6, 34.4, 31.8, 31.3, 31.2, 30.9, 30.3, 27.9, 27.7, 27.5, 27.1, 26.9, 11.4, 11.3, 11.2, 11.1, 11.0; GPC (THF) M_n = 30100 ± 300 g mol^{-1}, M_w = 124900 ± 0 g mol^{-1}, Đ = 4.15.

Synthesis of polymer **CEPU2**

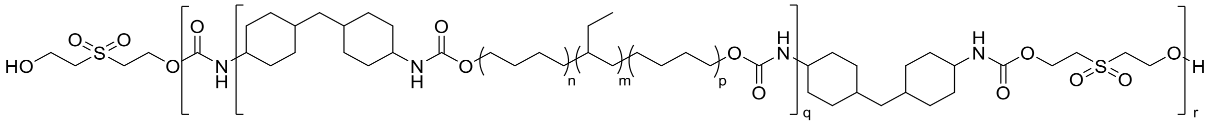

The synthesis was carried out according to the general synthetic protocol described above for CEPUs. The polymer was isolated as a transparent colourless elastomeric solid (6.53 g, 92 %) from Krasol HLBH-P 2000 (5.32 g, 2.53 mmol, 1.00 equiv.), 4,4'-methylene-bis(cyclohexyl isocyanate) (1.37 g, 5.22 mmol, 2.05 equiv.), and 2,2-sulfonyldiethanol (0.41 g, 2.66 mmol,

1.05 equiv.). T_g = -47.1 °C, T_m = 50.1 °C; FTIR ATR (cm^{-1}): 3343 (vN-$H_{stretch}$), 2960 (vC-H_{alkyl}), 2920 (vC-H_{alkyl}), 2852 (vC-H_{alkyl}), 1727 (vC=$O_{urethane}$), 1708 (vC=$O_{urethane}$), 1461 (vC-H_{alkyl}), 1379 (vC-H_{alkyl}), 1322 (vS=$O_{stretch}$); ^{1}H NMR (400 MHz, THF-d_8) δ 6.64 – 6.50 (m, 1H), 6.07 – 5.95 (m, 1H), 4.33 (m, 2H), 4.09 – 3.88 (m, 2H), 3.76 – 3.62 (m, 1H), 3.41 – 3.26 (m, 2H), 2.13 – 1.84 (m, 5H), 1.68 – 0.63 (m, 182H); ^{13}C NMR (100 MHz, THF-d_8) δ 156.6, 156.1, 64.6, 63.0, 59.4, 54.9, 51.5, 51.3, 48.5, 40.1, 39.6, 39.5, 39.0, 37.4, 35.1, 34.6, 34.4, 34.2, 33.5, 33.4, 31.8, 31.3, 31.2, 30.9, 30.6, 30.5, 29.1, 27.9, 27.7, 27.5, 27.2, 27.1, 26.9, 23.7, 11.4, 11.3, 11.2, 11.1, 11.0, 10.8; GPC (THF) M_n = 44700 ± 200 g mol^{-1}, M_w = 140400 ± 700 g mol^{-1}, Đ = 3.14.

Synthesis of polymer **CEPU3**

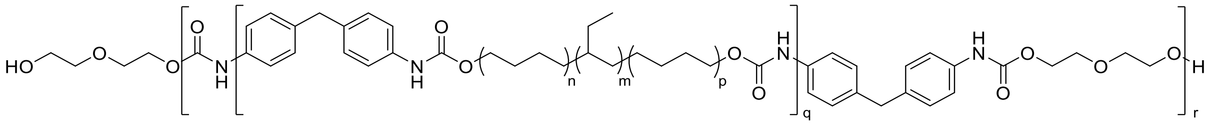

The synthesis was carried out according to the general synthetic protocol described above for CEPUs. The polymer was isolated as a transparent colourless elastomeric solid (7.06 g, 94 %) from Krasol HLBH-P 2000 (5.79 g, 2.76 mmol, 1.00 equiv.), 4,4'-methylenebis(phenyl isocyanate) (1.42 g, 5.67 mmol, 2.05 equiv.), and diethylene glycol (0.31 g, 2.92 mmol, 1.05 equiv.). T_g = -46.2 °C, T_m = 56.2, T_m = 82.2 °C; FTIR ATR (cm^{-1}): 3324 (vN-$H_{stretch}$), 2960 (vC-H_{alkyl}), 2921 (vC-H_{alkyl}), 2852 (vC-H_{alkyl}), 1733 (vC=$O_{urethane}$), 1706 (vC=$O_{urethane}$), 1597 (vC=$C_{stretch}$), 1461 (vC-H_{alkyl}); ^{1}H NMR (400 MHz, THF-d_8) δ 8.73 (s, 1H), 8.53 (s, 1H), 7.36 (d, J = 8.0 Hz, 4H), 7.03 (d, J = 8.2 Hz, 4H), 4.20 (t, J = 4.8 Hz, 2H), 4.09 (dt, J = 18.3, 6.8 Hz, 2H), 3.82 (s, 2H), 3.70 – 3.62 (m, 2H), 2.02 (dd, J = 12.7, 6.5 Hz, 2H), 1.70 – 0.64 (m, 146H); ^{13}C NMR (100 MHz, THF-d_8) δ 154.5, 154.4, 138.8, 138.7, 136.6, 136.4, 130.0, 129.9, 119.2, 119.0, 70.4, 64.5, 63.6, 41.4, 40.3, 40.1, 39.7, 39.5, 39.0, 37.4, 37.4, 37.3, 37.0, 34.6, 34.4, 31.8, 31.3, 31.2, 30.9, 30.3, 27.9, 27.7, 27.5, 27.4, 11.4, 11.3, 11.2, 11.2, 11.1, 11.0, 10.8; GPC (THF) M_n = 27700 ± 0 g mol^{-1}, M_w = 86900 ± 1600 g mol^{-1}, Đ = 3.14.

Synthesis of polymer **CEPU4**

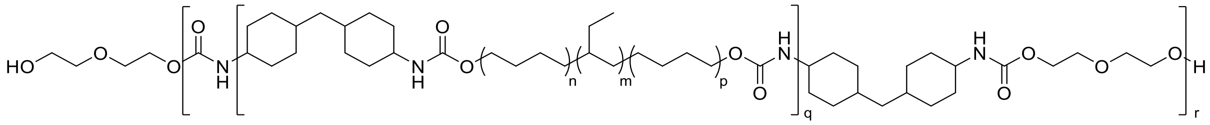

The synthesis was carried out according to the general synthetic protocol described above for CEPUs. The polymer was isolated as a transparent colourless elastomeric solid (6.01 g, 91 %) from Krasol HLBH-P 2000 (5.04 g, 2.40 mmol, 1.00 equiv.), 4,4'-methylene-bis(cyclohexyl isocyanate) (1.30 g, 4.96 mmol, 2.05 equiv.), and diethylene glycol (0.27 g, 2.54 mmol, 1.05 equiv.). T_g = -44.2 °C, T_m = 53.0 °C; FTIR ATR (cm^{-1}): 3325 ($\nu N\text{-}H_{stretch}$), 2959 ($\nu C\text{-}H_{alkyl}$), 2920 ($\nu C\text{-}H_{alkyl}$), 2852 ($\nu C\text{-}H_{alkyl}$), 1722 ($\nu C{=}O_{urethane}$), 1698 ($\nu C{=}O_{urethane}$), 1461 ($\nu C\text{-}H_{alkyl}$), 1379 ($\nu C\text{-}H_{alkyl}$); ^{1}H NMR (400 MHz, THF-d_8) δ 6.24 (dd, J = 16.2, 7.2 Hz, 2H), 6.01 (s, 2H), 4.07 (d, J = 5.1 Hz, 3H), 3.97 (d, J = 17.8 Hz, 1H), 3.64 (s, 1H), 3.56 (s, 3H), 3.37 – 3.24 (m, 1H), 2.07 – 1.87 (m, 9H), 1.69 – 0.63 (m, 275H); ^{13}C NMR (100 MHz, THF-d_8) δ 156.5, 156.4, 70.6, 64.6, 64.1, 63.0, 51.3, 48.3, 45.6, 40.3, 40.1, 39.7, 39.5, 39.0, 37.4, 37.3, 35.1, 34.6, 34.4, 34.2, 33.8, 33.5, 33.4, 31.8, 31.3, 31.2, 30.9, 30.6, 30.5, 29.1, 27.9, 27.7, 27.5, 27.3, 27.2, 27.1, 11.4, 11.3, 11.3, 11.2, 11.2, 11.1, 11.0, 10.8; GPC (THF) M_n = 57400 ± 500 g mol^{-1}, M_w = 197000 ± 300 g mol^{-1}, Đ = 3.43.

Synthesis of polymer **CEPU5**

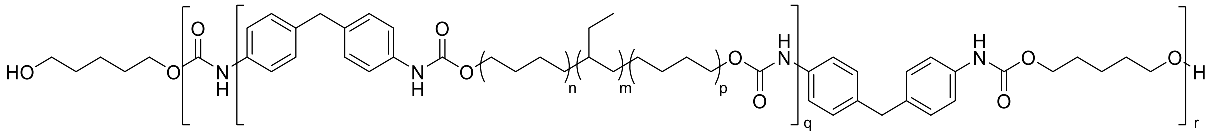

The synthesis was carried out according to the general synthetic protocol described above for CEPUs. The polymer was isolated as a transparent colourless elastomeric solid (6.88 g, 96 %) from Krasol HLBH-P 2000 (5.54 g, 2.64 mmol, 1.00 equiv.), 4,4'-methylenebis(phenyl isocyanate) (1.35 g, 5.39 mmol, 2.05 equiv.), and 1,5-pentanediol (0.29 g, 2.78 mmol, 1.05 equiv.). T_g = -46.0 °C, T_m = 101.6 °C; FTIR ATR (cm^{-1}): 3336 ($\nu N\text{-}H_{stretch}$), 2959 ($\nu C\text{-}H_{alkyl}$),

2921 (νC-H_{alkyl}), 2853 (νC-H_{alkyl}), 1735 (νC=$O_{urethane}$), 1704 (νC=$O_{urethane}$), 1597 (νC=$C_{stretch}$), 1462 (νC-H_{alkyl}); ^{1}H NMR (400 MHz, THF-d_8) δ 8.58 (s, 1H), 8.54 (s, 1H), 7.36 (d, J = 8.1 Hz, 4H), 7.03 (d, J = 8.3 Hz, 4H), 4.09 (h, J = 7.5 Hz, 4H), 3.82 (s, 2H), 2.02 (q, J = 6.4 Hz, 1H), 1.71 – 0.67 (m, 140H); ^{13}C NMR (100 MHz, THF-d_8) δ 154.5, 154.4 138.8, 136.5, 136.4, 129.9, 119.2, 119.0, 65.2, 65.1, 63.6, 41.4, 40.1, 39.7, 39.5, 39.0, 37.4, 37.4, 34.6, 34.4, 31.8, 31.3, 30.9, 30.3, 29.9, 27.9, 27.7, 27.5, 27.3, 27.1, 26.9, 11.4, 11.3, 11.2, 11.1, 10.9; GPC (THF) M_n = 26600 ± 100 g mol^{-1}, M_w = 90900 ± 900 g mol^{-1}, Đ = 3.42.

Synthesis of polymer **CEPU6**

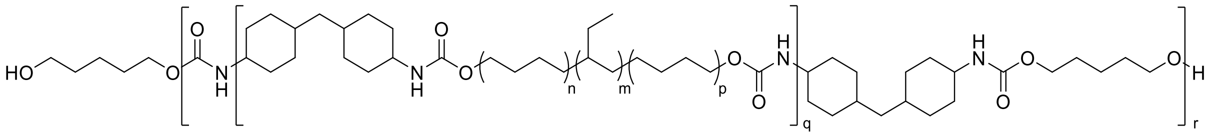

The synthesis was carried out according to the general synthetic protocol described above for CEPUs. The polymer was isolated as a transparent colourless elastomeric solid (6.54 g, 90 %) from Krasol HLBH-P 2000 (5.54 g, 2.64 mmol, 1.00 equiv.), 4,4'-methylene-bis(cyclohexyl isocyanate) (1.42 g, 5.41 mmol, 2.05 equiv.), and 1,5-pentanediol (0.29 g, 2.78 mmol, 1.05 equiv.). T_g = -45.1 °C, T_m = 45.7 °C; FTIR ATR (cm^{-1}): 3337 (νN-$H_{stretch}$), 2959 (νC-H_{alkyl}), 2920 (νC-H_{alkyl}), 2853 (νC-H_{alkyl}), 1724 (νC=$O_{urethane}$), 1701 (νC=$O_{urethane}$), 1461 (νC-H_{alkyl}), 1378 (νC-H_{alkyl}); ^{1}H NMR (400 MHz, THF-d_8) δ 6.07 (s, 1H), 6.02 (s, 2H), 5.91 – 5.66 (m, 1H), 5.41 – 4.68 (m, 1H), 3.95 (d, J = 6.2 Hz, 5H), 3.68 – 3.58 (m, 2H), 3.35 – 3.17 (m, 2H), 2.10 – 1.84 (m, 6H), 1.64 – 0.64 (m, 248H); ^{13}C NMR (100 MHz, THF-d_8) δ 156.6, 156.5, 64.6, 51.3, 48.3, 40.3, 40.1, 39.7, 39.5, 39.0, 37.4, 37.4, 37.3, 35.1, 35.1, 34.6, 34.4, 34.3, 33.8, 33.7, 33.5, 33.4, 31.8, 31.3, 31.2, 30.9, 30.6, 30.5, 30.1, 29.1, 27.9, 27.7, 27.5, 27.4, 27.2, 27.1, 26.9, 23.6, 11.4, 11.3, 11.3, 11.2, 11.2, 11.1, 11.0, 10.8; GPC (THF) M_n = 64800 ± 800 g mol^{-1}, M_w = 199500 ± 100 g mol^{-1}, Đ = 3.08.

General synthetic protocol for methoxy terminated pre-polymers (**MeO-PU1** and **MeO-PU2**)

Hydrogenated poly(butadiene) (Krasol HLBH-P 2000), molecular weight as supplied = 2100 g mol^{-1}, was dried under vacuum in the oven at 80 °C for 2 hours. In the bulk, Krasol HLBH-P 2000 (1.00 equiv.) was mixed with diisocyanate (2.05 equiv.) and dibutyltin dilaurate (10 drops, **MeO-PU2** only) at 80 °C under argon for 3 hours with gentle stirring. The colourless pre-polymer obtained was precipitated into ice cold methanol (3 x 1000 mL), the solid material filtered off and then dried *in vacuo*.

Synthesis of polymer **MeO-PU1**

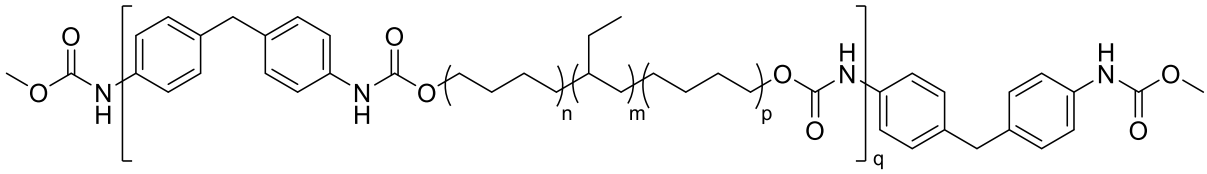

The synthesis was carried out according to the general synthetic protocol described above for methoxy terminated pre-polymers. The polymer was isolated as a transparent colourless elastomeric solid (12.27 g, 98 %) from Krasol HLBH-P 2000 (10.06 g, 4.79 mmol, 1.00 equiv.), and 4,4'-methylenebis(phenyl isocyanate) (2.46 g, 9.83 mmol, 2.05 equiv.). T_g = -45.6 °C, T_m = 41.2 °C, T_m = 66.0 °C, T_c = 34.0 °C, T_m = 38.2 °C, T_m = 75.1 °C, T_c = 49.1 °C; FTIR ATR (cm^{-1}): 3331 ($vN\text{-}H_{stretch}$), 2960 ($vC\text{-}H_{alkyl}$), 2921 ($vC\text{-}H_{alkyl}$), 2853 ($vC\text{-}H_{alkyl}$), 1740 ($vC{=}O_{urethane}$), 1707 ($vC{=}O_{urethane}$), 1599 ($vC{=}C_{stretch}$), 1462 ($vC\text{-}H_{alkyl}$); ^{1}H NMR (400 MHz, THF-d_8) δ 8.58 (s, 1H), 8.53 (d, J = 6.7 Hz, 1H), 7.36 (dd, J = 8.6, 2.4 Hz, 3H), 7.03 (dd, J = 8.6, 2.4 Hz, 4H), 4.17 – 4.09 (m, 1H), 4.12 – 4.04 (m, 1H), 3.82 (s, 2H), 3.64 (s, 3H), 2.09 – 1.86 (m, 2H), 1.68 – 0.78 (m, 153H); ^{13}C NMR (100 MHz, THF-d_8) δ 154.87, 154.49, 138.83, 138.73, 136.55, 136.43, 129.95, 129.93, 119.23, 119.03, 63.55, 51.96, 41.44, 40.31, 40.25, 40.13, 39.65, 39.53, 39.03, 38.87, 37.36, 37.02, 34.62, 34.35, 33.71, 31.75, 31.31, 30.88, 30.31, 27.87, 27.70, 27.53, 27.36, 27.12, 26.94, 11.43, 11.25, 11.21, 11.16, 11.12, 11.00, 10.81; GPC (THF) M_n = 8500 ± 0 g mol^{-1}, M_w = 16300 ± 600 g mol^{-1}, Đ = 1.92.

Synthesis of polymer **MeO-PU2**

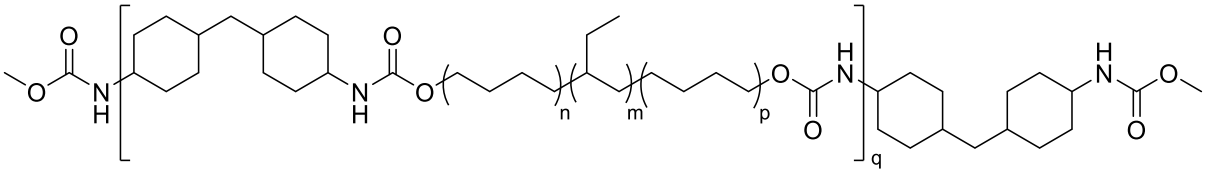

The synthesis was carried out according to the general synthetic protocol described above for methoxy terminated pre-polymers. The polymer was isolated as a transparent colourless elastomeric solid (12.53 g, 97 %) from Krasol HLBH-P 2000 (10.26 g, 4.89 mmol, 1.00 equiv.), and 4,4'-methylene-bis(cyclohexyl isocyanate) (2.63 g, 10.02 mmol, 2.05 equiv.). T_g = -44.3 °C, T_m = 38.3 °C, T_m = 37.4 °C, T_c = 16.8 °C; FTIR ATR (cm^{-1}): 3326 ($\nu N\text{-}H_{stretch}$), 2959 ($\nu C\text{-}H_{alkyl}$), 2920 ($\nu C\text{-}H_{alkyl}$), 2854 ($\nu C\text{-}H_{alkyl}$), 1713 ($\nu C{=}O_{urethane}$), 1691 ($\nu C{=}O_{urethane}$), 1459 ($\nu C\text{-}H_{alkyl}$), 1379 ($\nu C\text{-}H_{alkyl}$); ^{1}H NMR (400 MHz, THF-d_8) δ 6.15 – 6.03 (m, 1H), 6.03 – 5.91 (m, 1H), 4.09 – 3.87 (m, 2H), 3.64 (s, 1H), 3.52 (t, J = 5.7 Hz, 3H), 3.35 – 3.23 (m, 1H), 2.13 – 1.96 (m, 2H), 1.96 – 1.86 (m, 3H), 1.68 – 0.78 (m, 92H); ^{13}C NMR (100 MHz, THF-d_8) δ 156.93, 156.55, 68.01, 67.79, 67.35, 67.13, 64.63, 62.91, 51.50, 51.35, 51.25, 48.35, 45.62, 43.99, 40.29, 40.13, 39.66, 39.54, 39.03, 37.42, 37.36, 37.29, 35.07, 34.63, 34.35, 34.28, 33.80, 33.69, 33.47, 33.44, 33.40, 31.76, 31.31, 30.88, 30.61, 29.13, 27.87, 27.70, 27.53, 27.34, 27.19, 27.12, 26.94, 25.90, 25.70, 25.50, 25.30, 25.10, 11.43, 11.25, 11.22, 11.12, 10.96, 10.83; GPC (THF) M_n = 7900 ± 100 g mol^{-1}, M_w = 13700 ± 300 g mol^{-1}, Đ = 1.73.

Protocol for casting CEPUs (**CEPU1**-**CEPU6**) and methoxy terminated pre-polymers (**MeO-PU1** and **MeO-PU2**)

The precipitated polymer was dissolved in a minimum volume of THF (approximately 3 mL per 1 g of polymer) at 40 °C whilst stirring. Once fully dissolved, the polymer solution was poured into a 15 cm x 15 cm mould with a PTFE base. The solvent was allowed to slowly evaporate over 24 hours at room temperature and pressure. The mould was placed into a vacuum oven at 60 °C for 24 hours, then under partial vacuum (approximately 800 mbar) at 60 °C for 24 hours, the polymer film was then allowed to reach room temperature before being removed from the mould.

Protocol for NMR solution state degradation

Model small molecules (**1**-**4**) were made to 10 mg mL^{-1} in MeCN-d_3 and CEPUs (**CEPU1** and **CEPU2**) were made to 50 mg mL^{-1} in THF-d_8. The addition of 5 equiv. (**1**-**4**, **CEPU1**, and **CEPU2**) of NaOD (40 wt.% in D2O) or TBAF (1 M in THF) was added directly to the NMR tube. The ^1H NMR spectra were recorded at regular time intervals. Urethane (**1** and **2**), N-methyl (**3** and **4**), aromatic (**1** and **3**), and methylene (**2** and **4**) signals were used to calculate the percentage of the different species remaining over time (i.e. before the addition of NaOD or TBAF, time point = 0) for small molecules **1**-**4**.

Protocol for GPC solution state degradation

Polymer solutions of CEPUs (**CEPU1**-**CEPU6**) were made to 1 mg mL^{-1} in HLPC grade THF, 1 M TBAF in THF (5 equiv.) was added to the solutions and shaken prior to testing. All reported molecular weights are the averages of three separate samples of each CEPU, the error shown is the standard deviation between the three repeats of each sample.

Protocol for solid state degradation

Cut sections of CEPUs (**CEPU1** and **CEPU2**) were submerged in solutions of 1M TBAF in acetone or 40 wt.% $NaOH_{(aq)}$ for set periods of time at either room temperature (TBAF) or 50 °C (NaOH). The polymer samples were then washed with either acetone (TBAF) or deionised water (NaOH) then dried at room temperature for 12 hours under vacuum, the polymer samples were then allowed to rest at room temperature for 48 hours before being analysed.

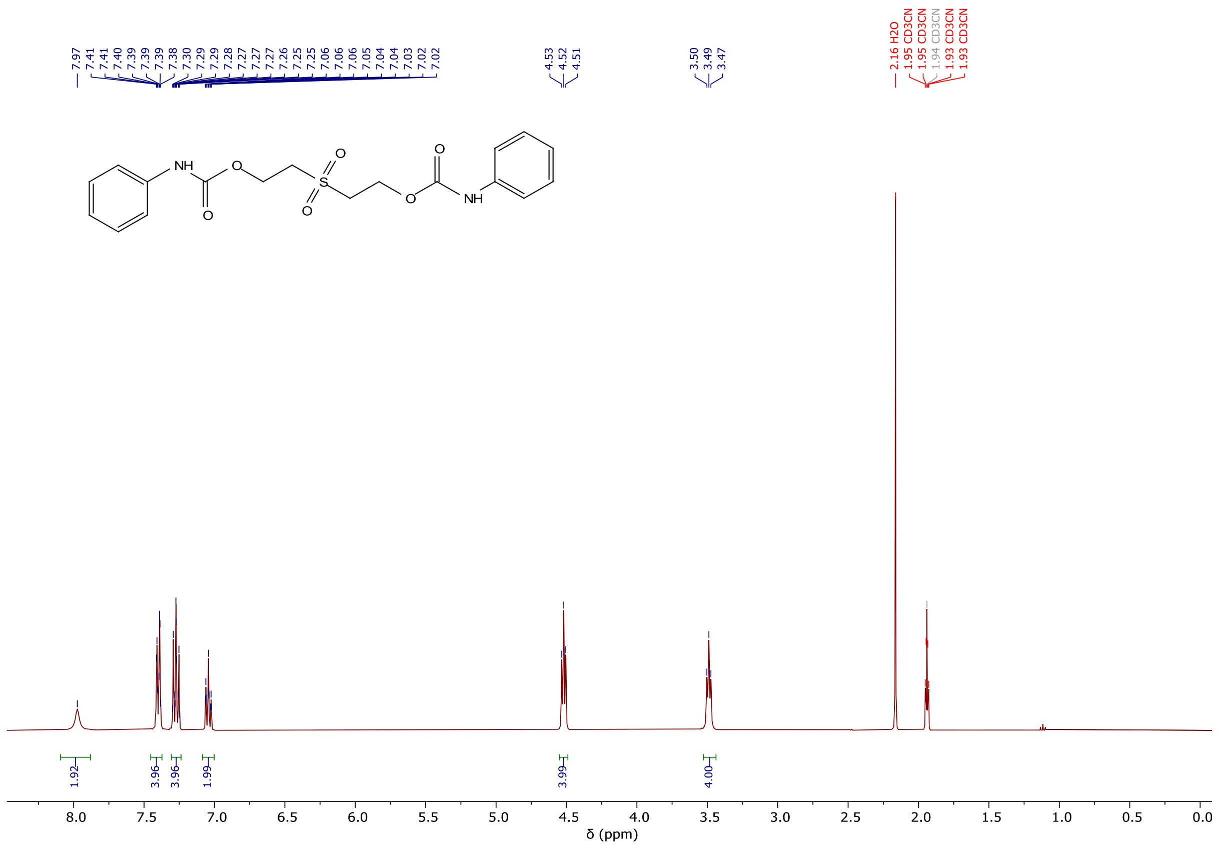

Figure S 1 ^{1}H NMR spectrum of **1** (400 MHz, MeCN-d_3, 298 K).

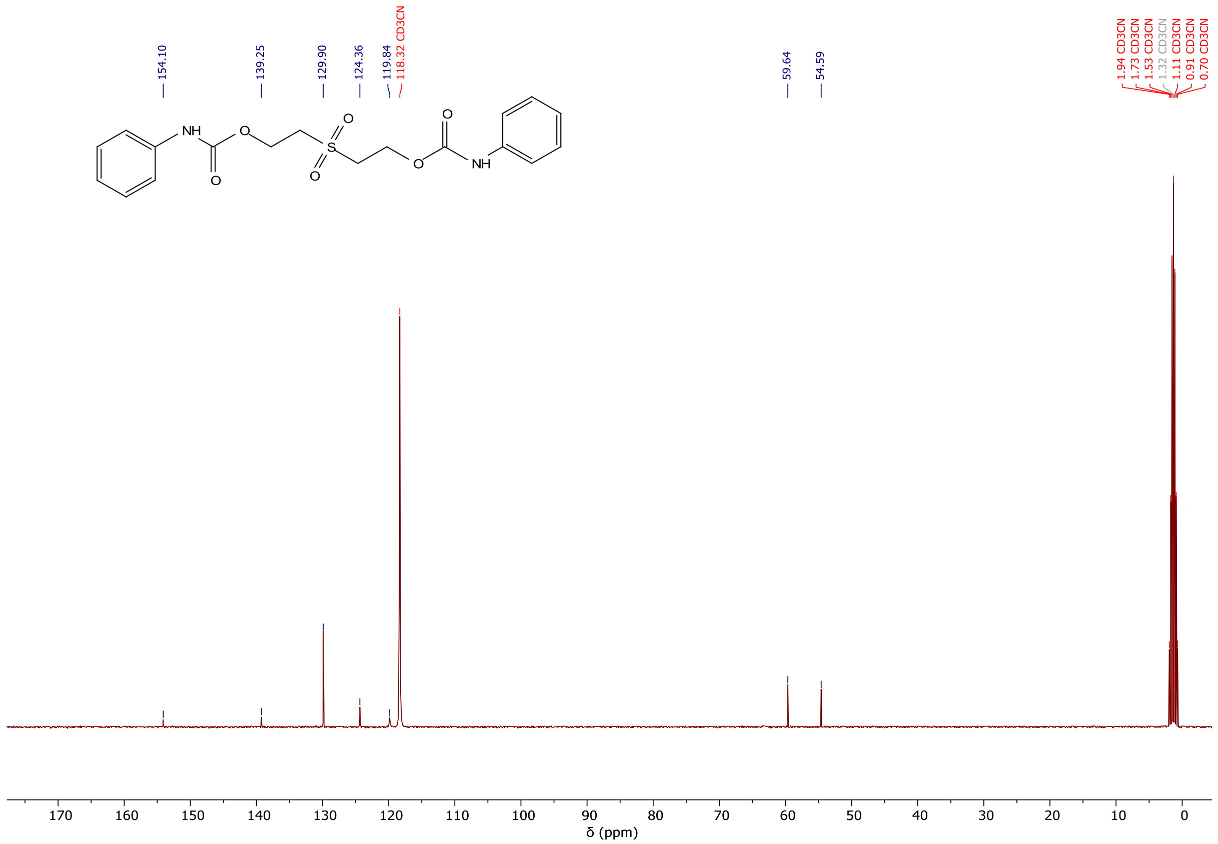

Figure S 2 ^{13}C {H} NMR spectrum of **1** (100 MHz, MeCN-d_3, 298 K).

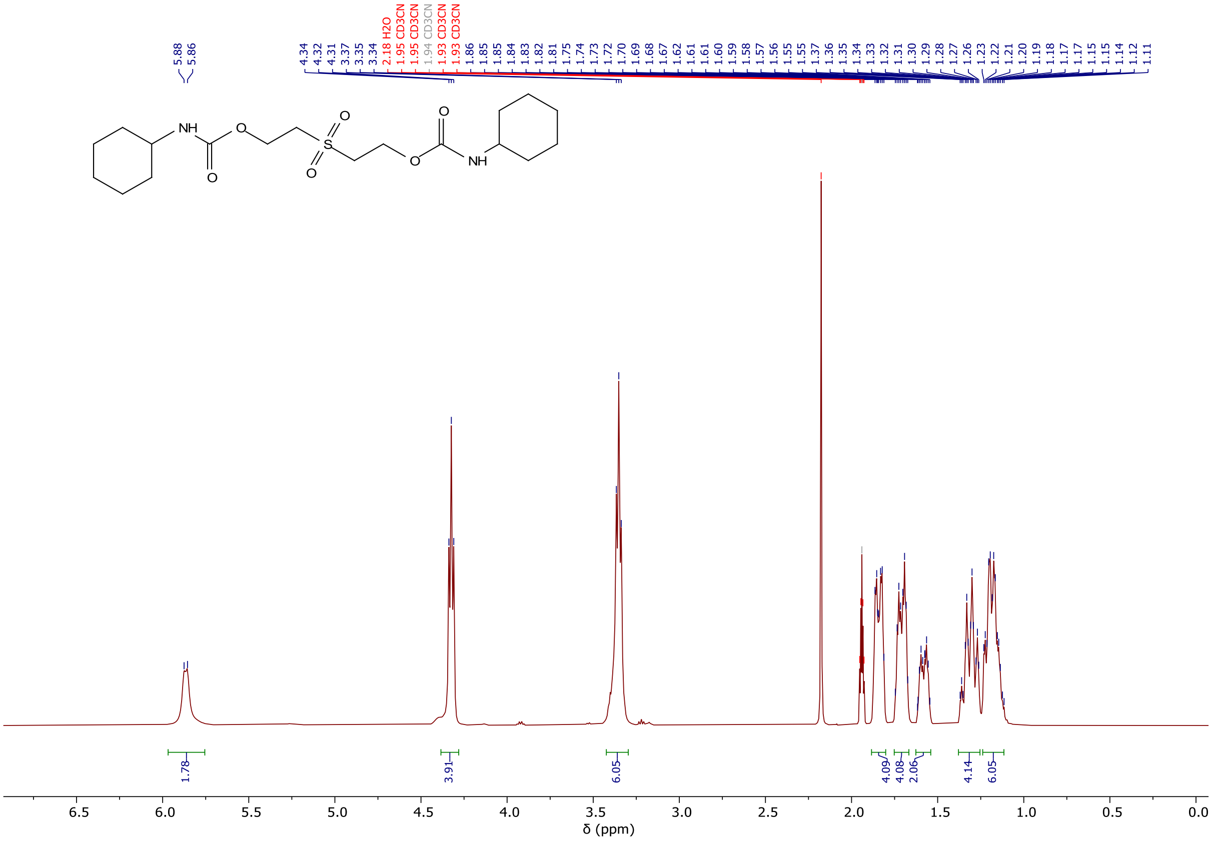

Figure S 3 ^{1}H NMR spectrum of **2** (400 MHz, MeCN-d_3, 298 K).

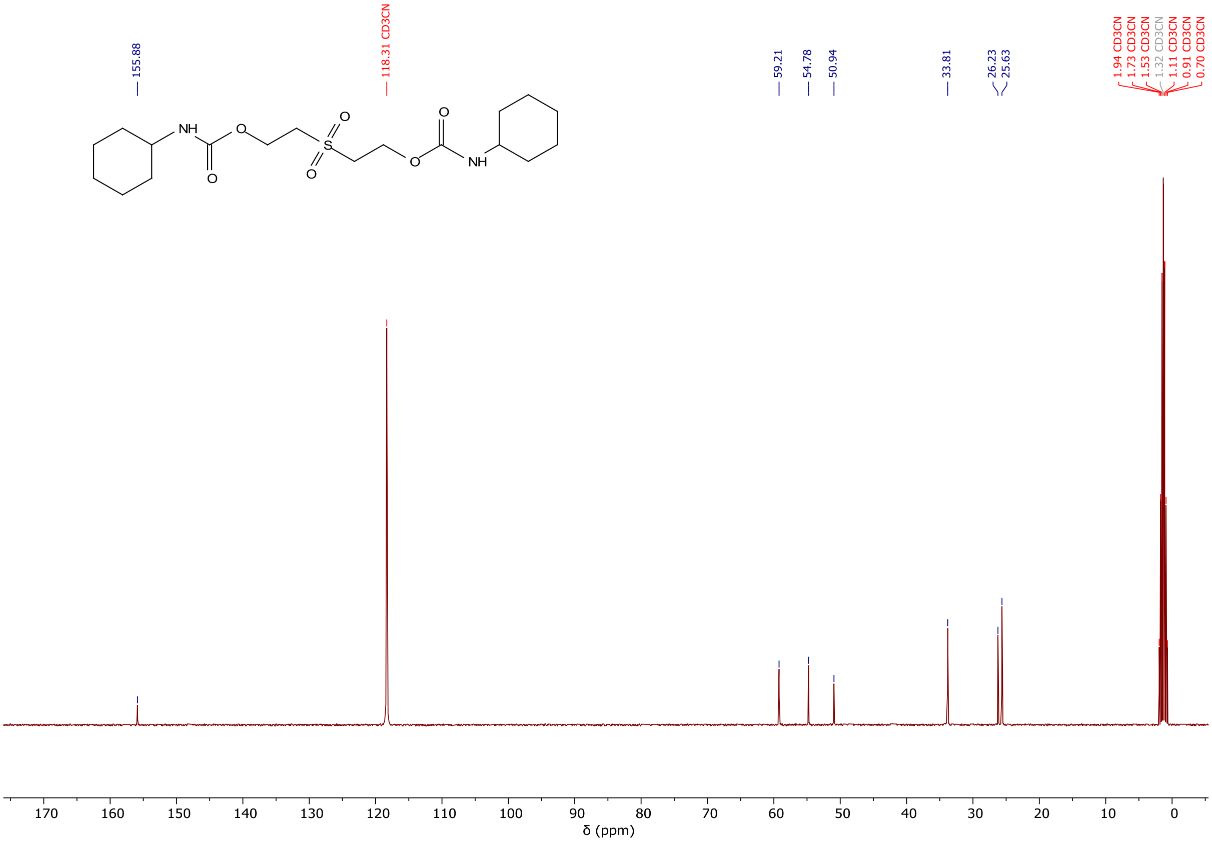

Figure S 4 ^{13}C {H} NMR spectrum of **2** (100 MHz, MeCN-d_3, 298 K).

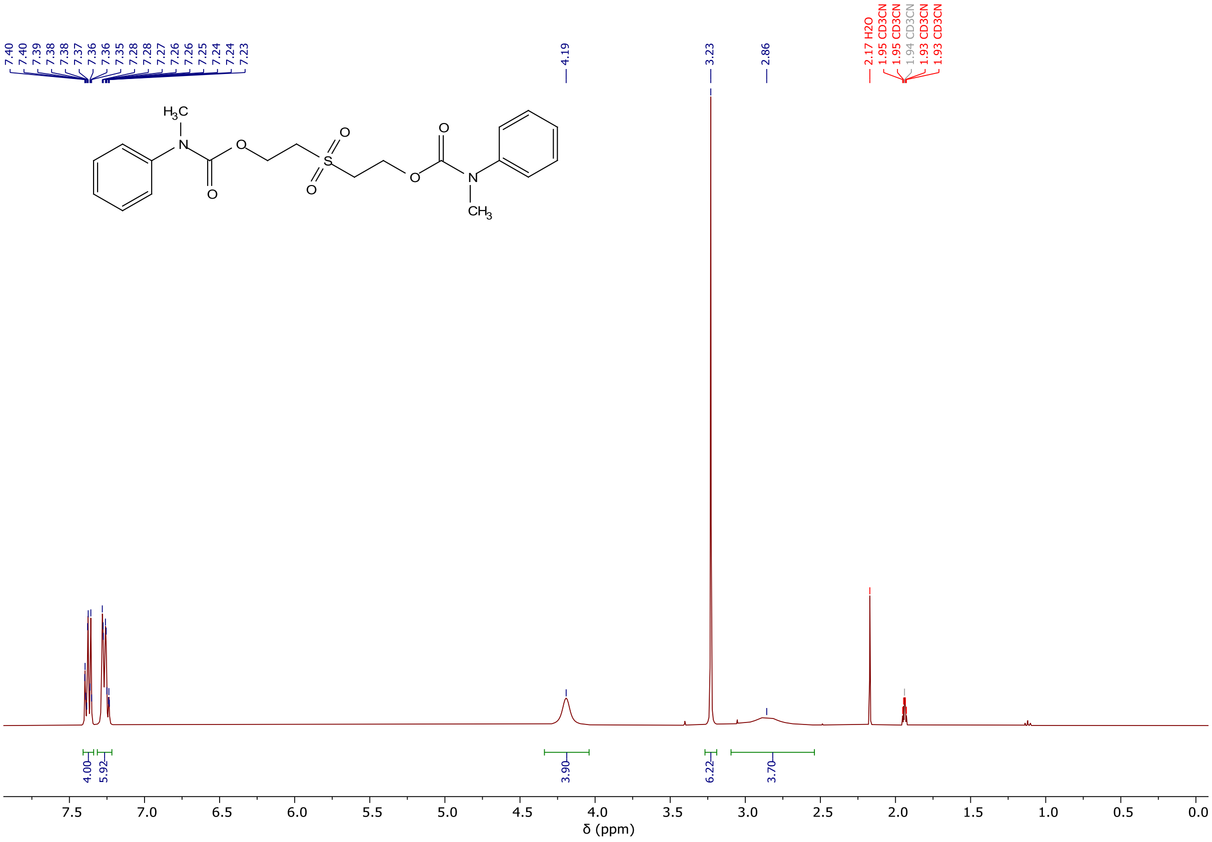

Figure S 5 ^{1}H NMR spectrum of **3** (400 MHz, MeCN-d_3, 298 K).

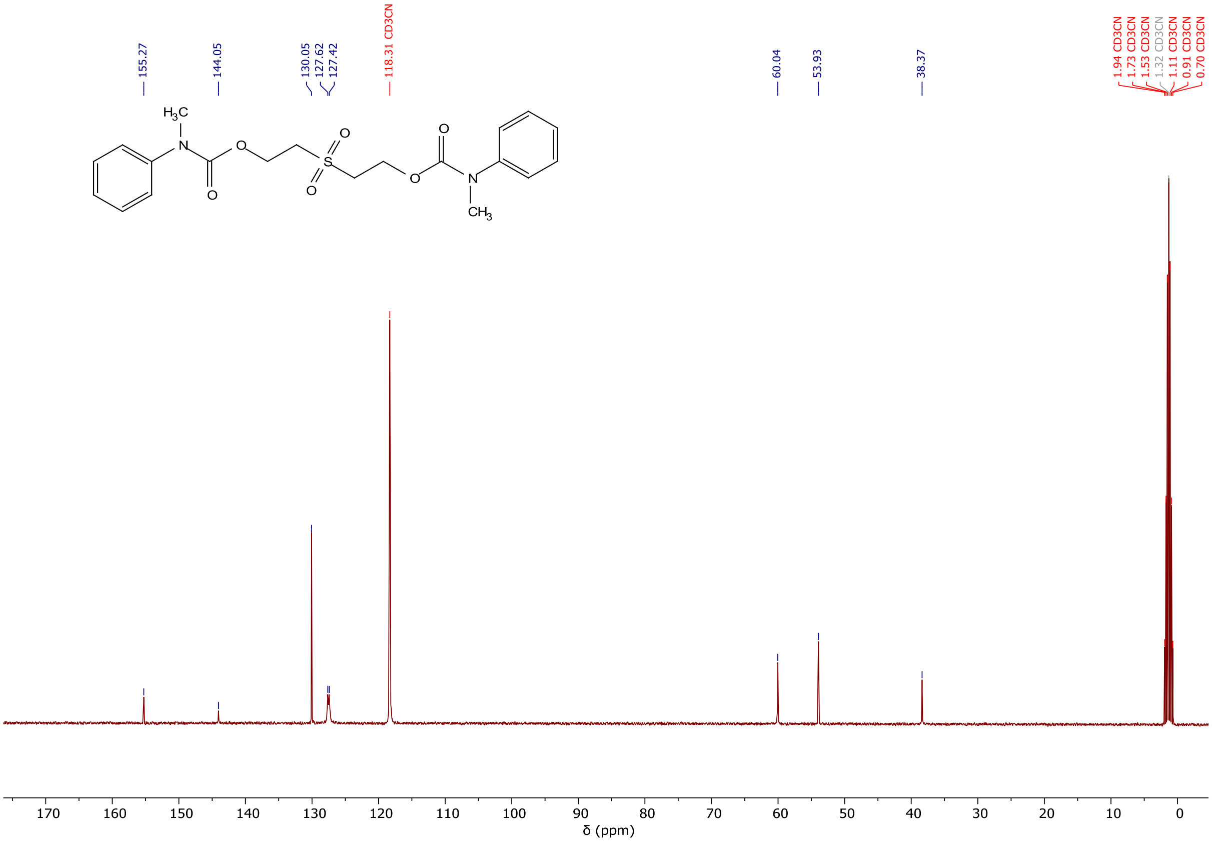

Figure S 6 ^{13}C {H} NMR spectrum of **3** (100 MHz, MeCN-d_3, 298 K).

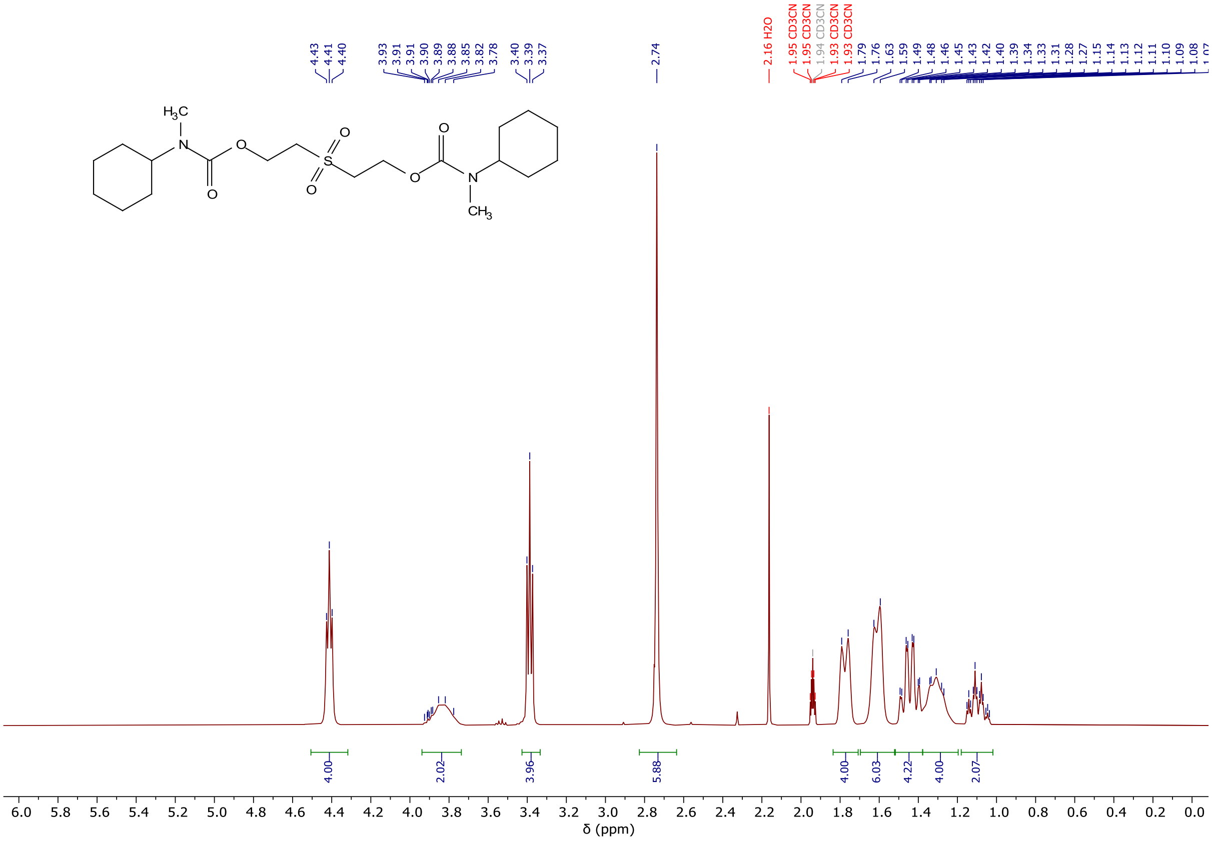

Figure S 7 ^{1}H NMR spectrum of **4** (400 MHz, MeCN-d_3, 298 K).

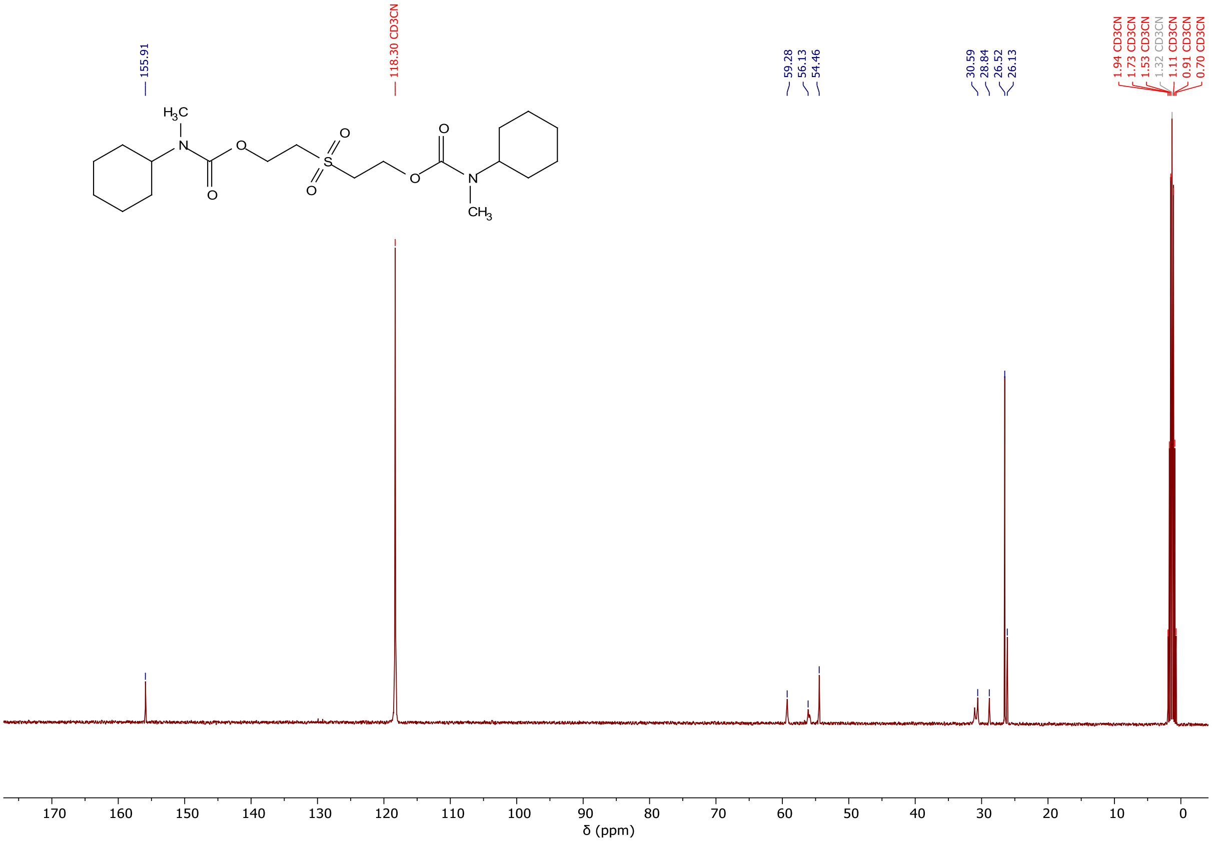

Figure S 8 ^{13}C {H} NMR spectrum of **4** (100 MHz, MeCN-d_3, 298 K).

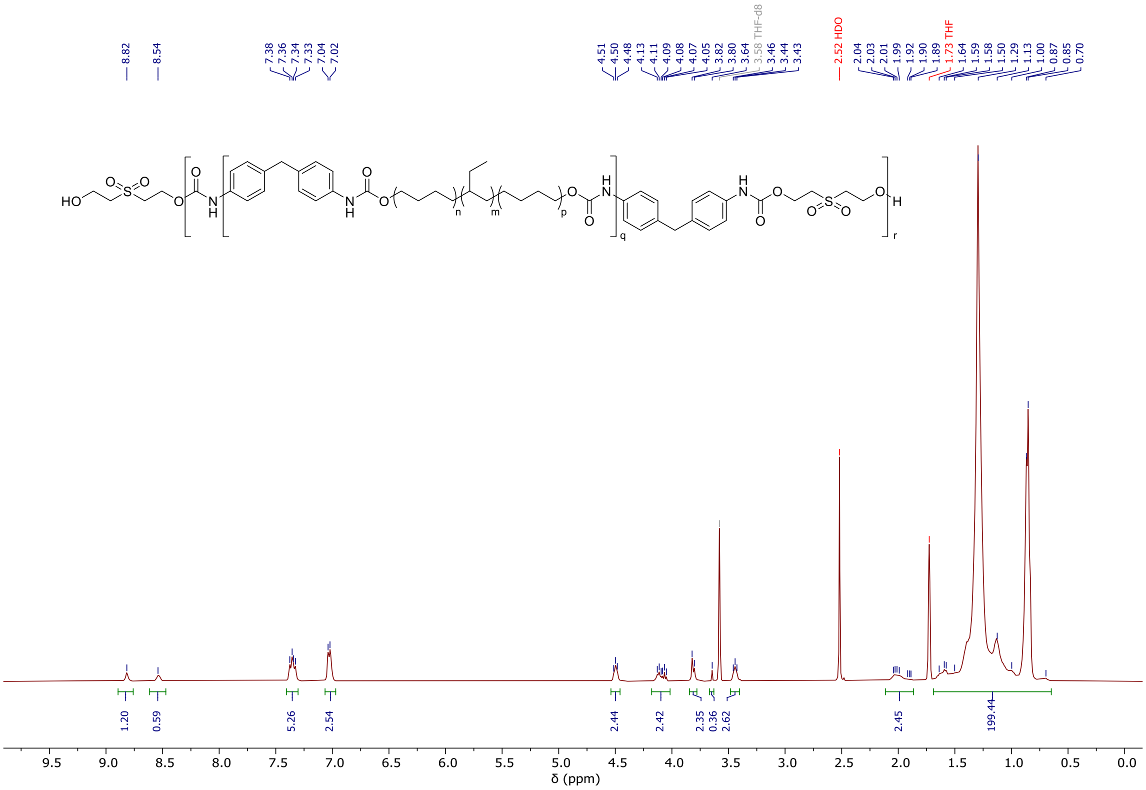

Figure S 9 ^{1}H NMR spectrum of **CEPU1** (400 MHz, THF-d_8, 298 K).

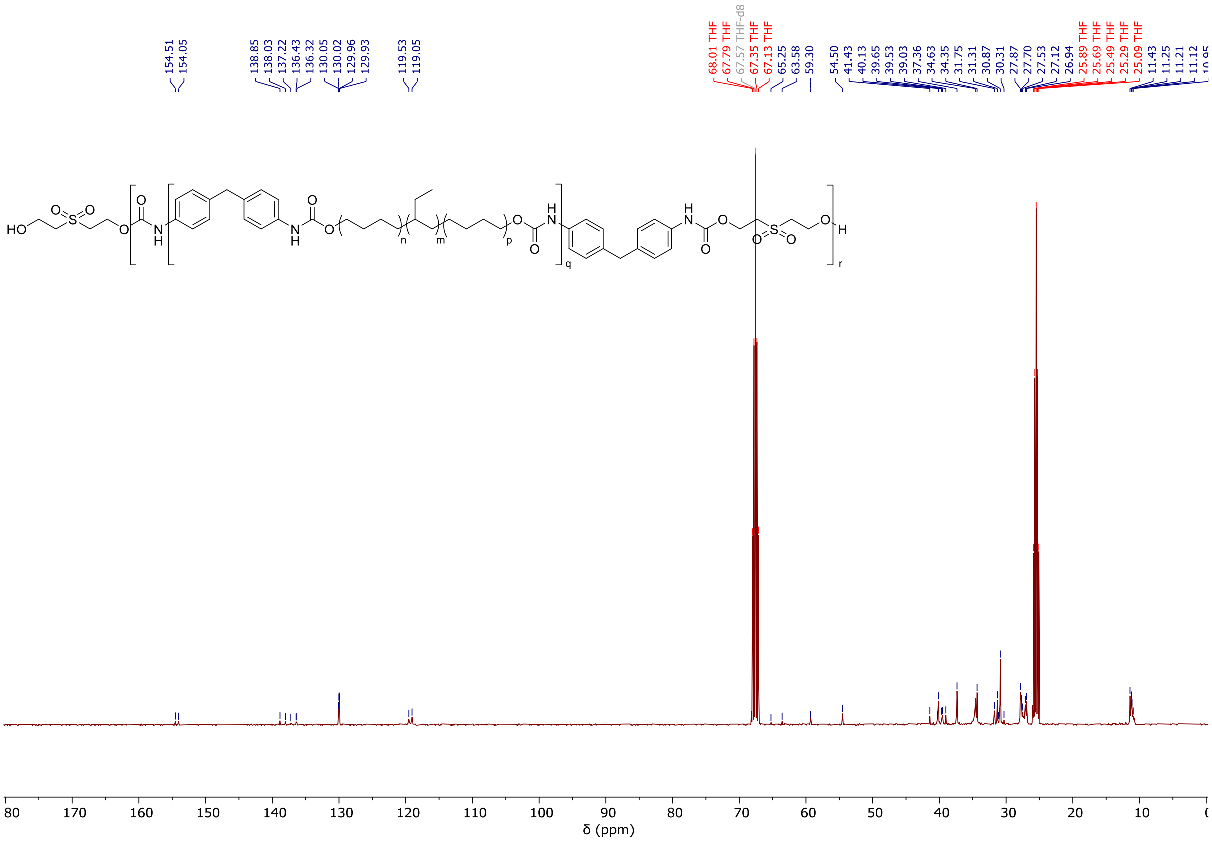

Figure S 10 ^{13}C {H} NMR spectrum of **CEPU1** (100 MHz, THF-d_8, 298 K).

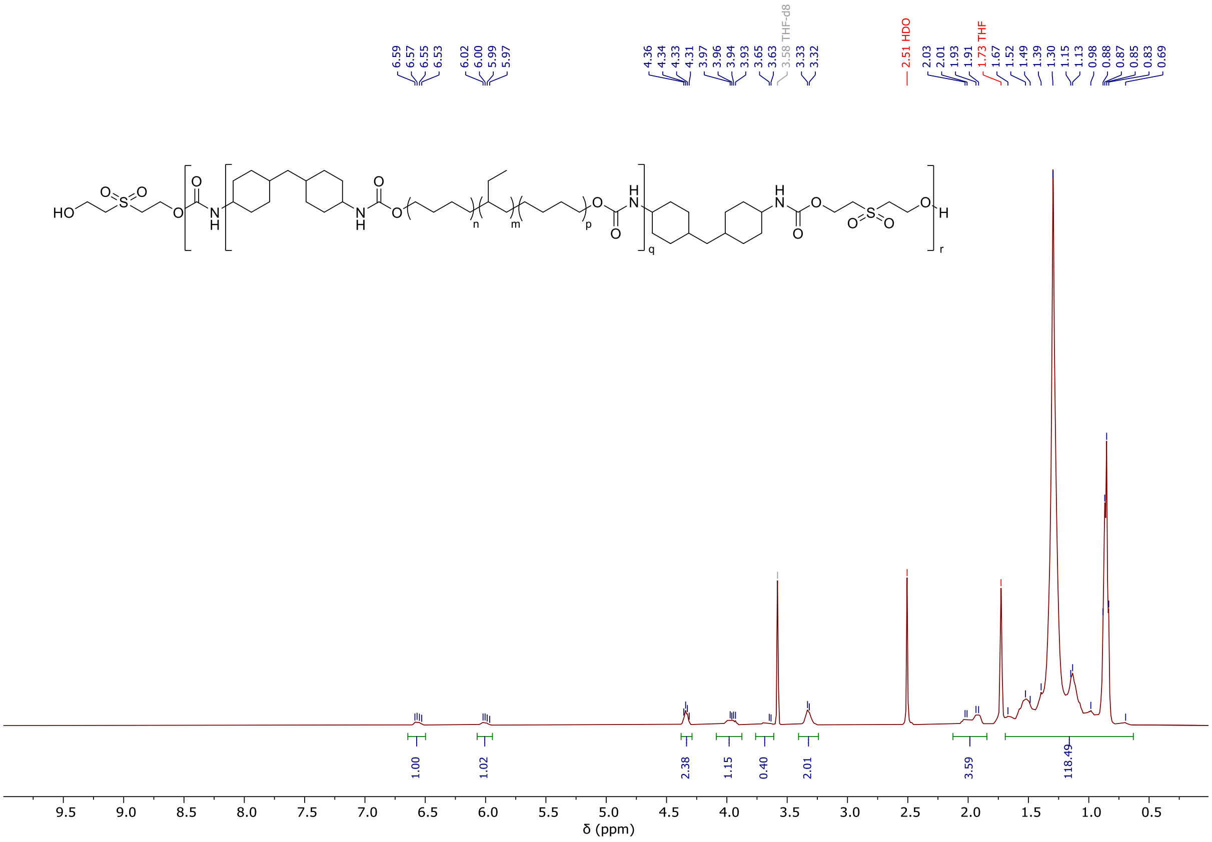

Figure S 11 ^{1}H NMR spectrum of **CEPU2** (400 MHz, THF-d_8, 298 K).

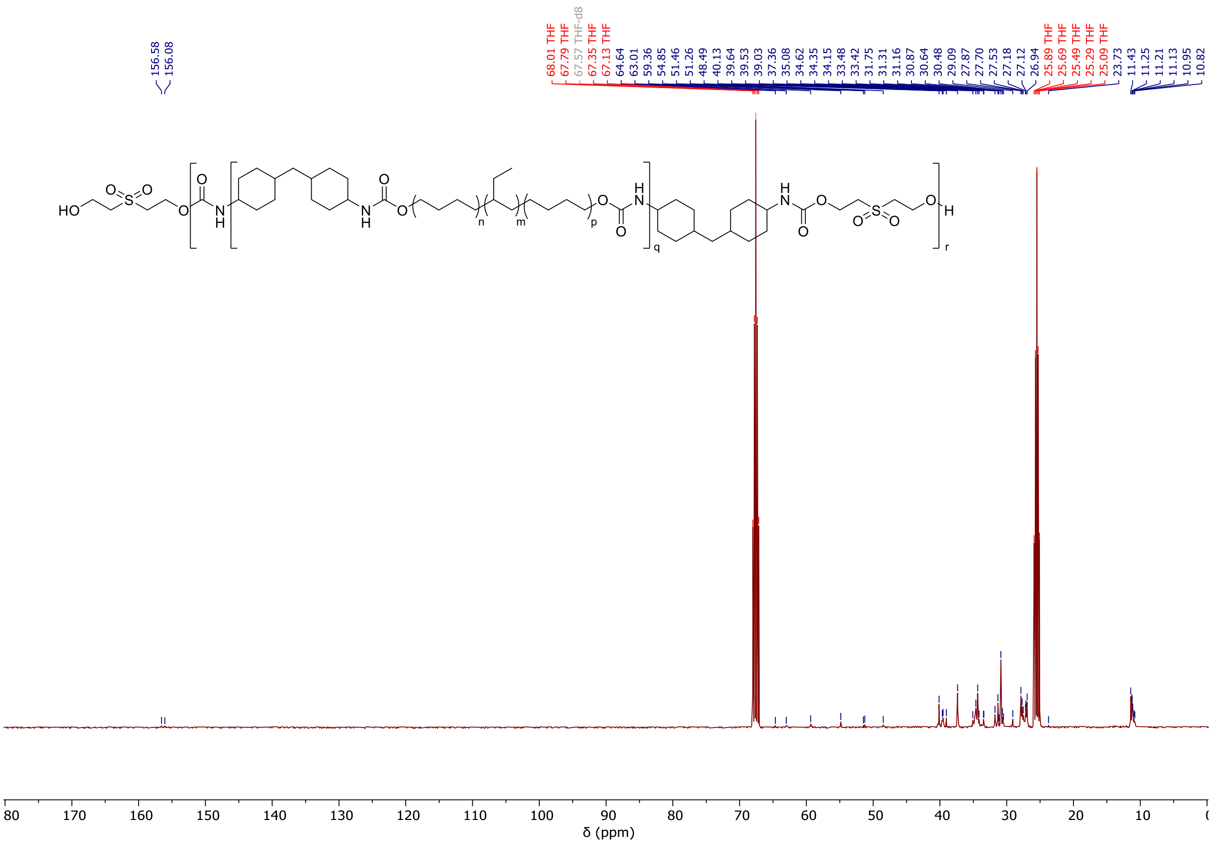

Figure S 12 ^{13}C {H} NMR spectrum of **CEPU2** (100 MHz, THF-d_8, 298 K).

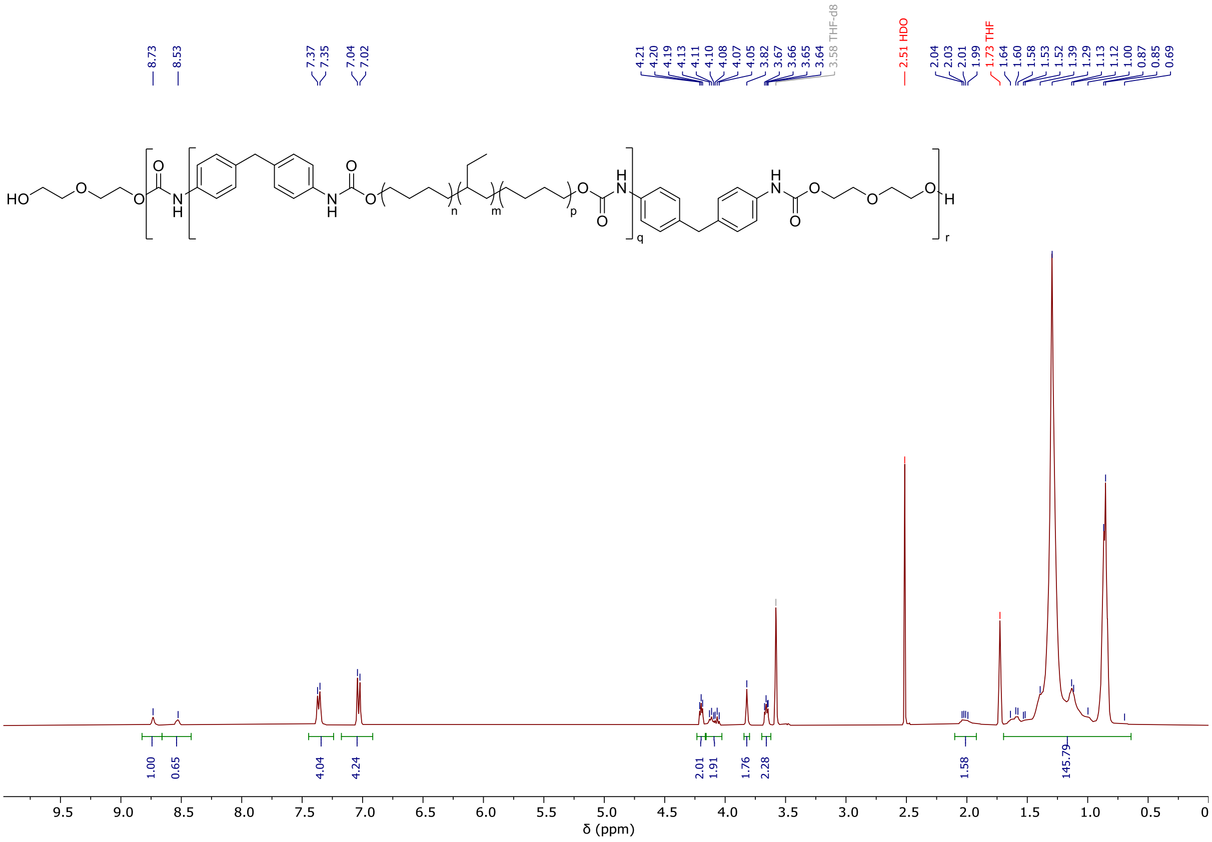

Figure S 13 ^{1}H NMR spectrum of **CEPU3** (400 MHz, THF-d_8, 298 K).

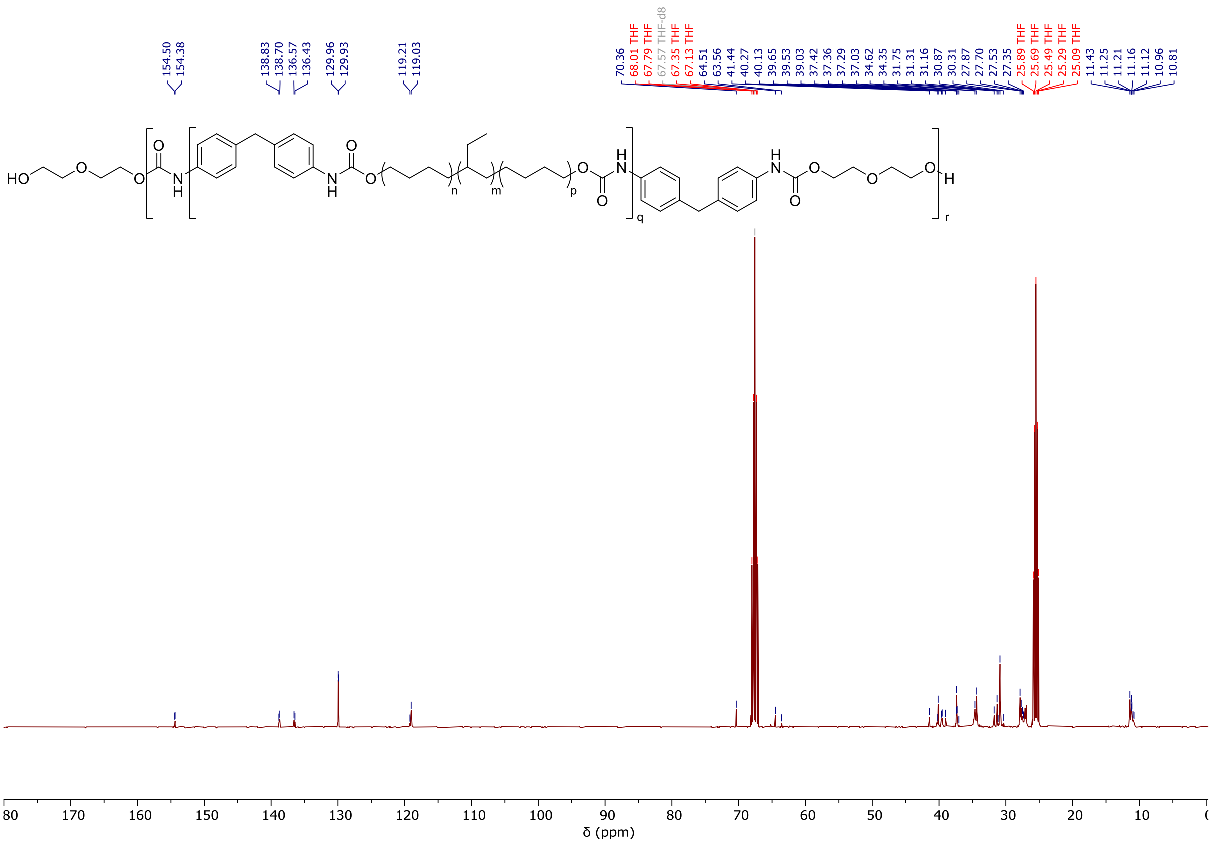

Figure S 14 ^{13}C {H} NMR spectrum of **CEPU3** (100 MHz, THF-d_8, 298 K).

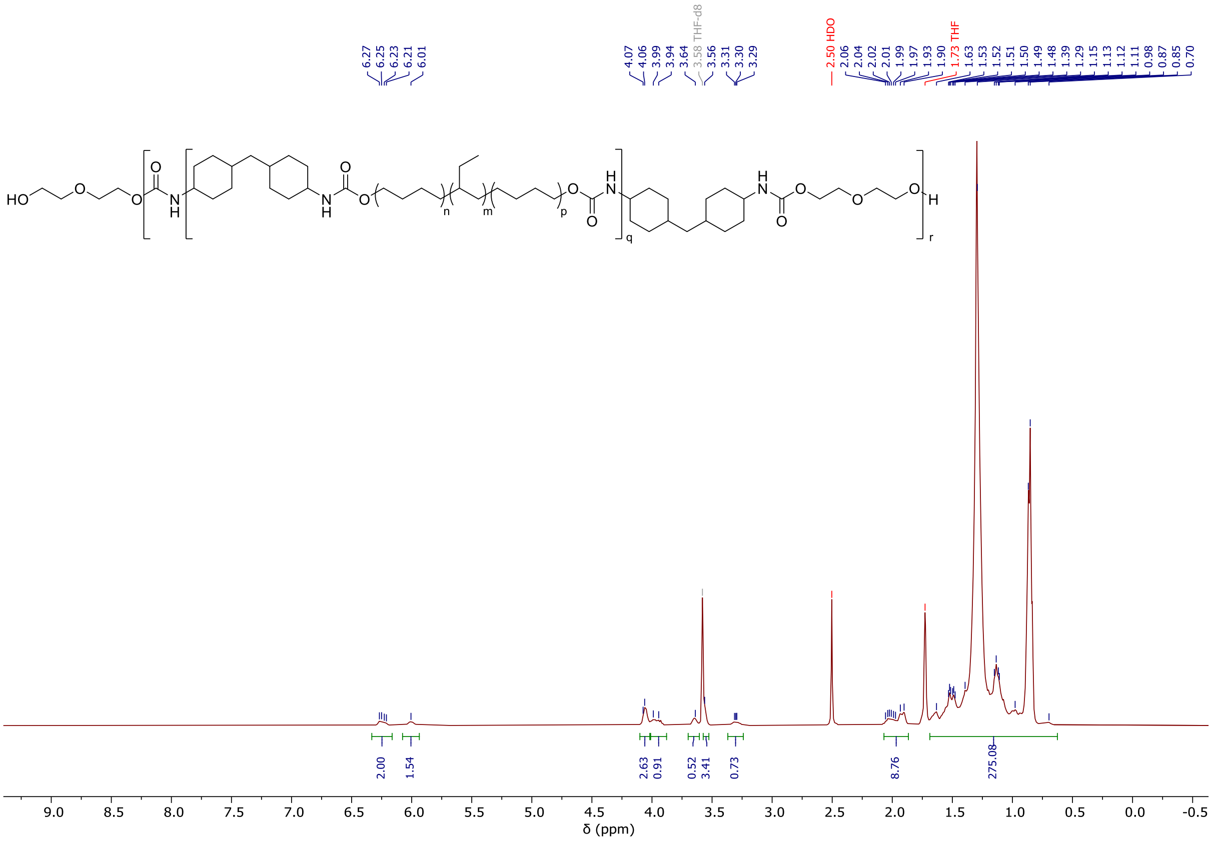

Figure S 15 ^{1}H NMR spectrum of **CEPU4** (400 MHz, THF-d_8, 298 K).

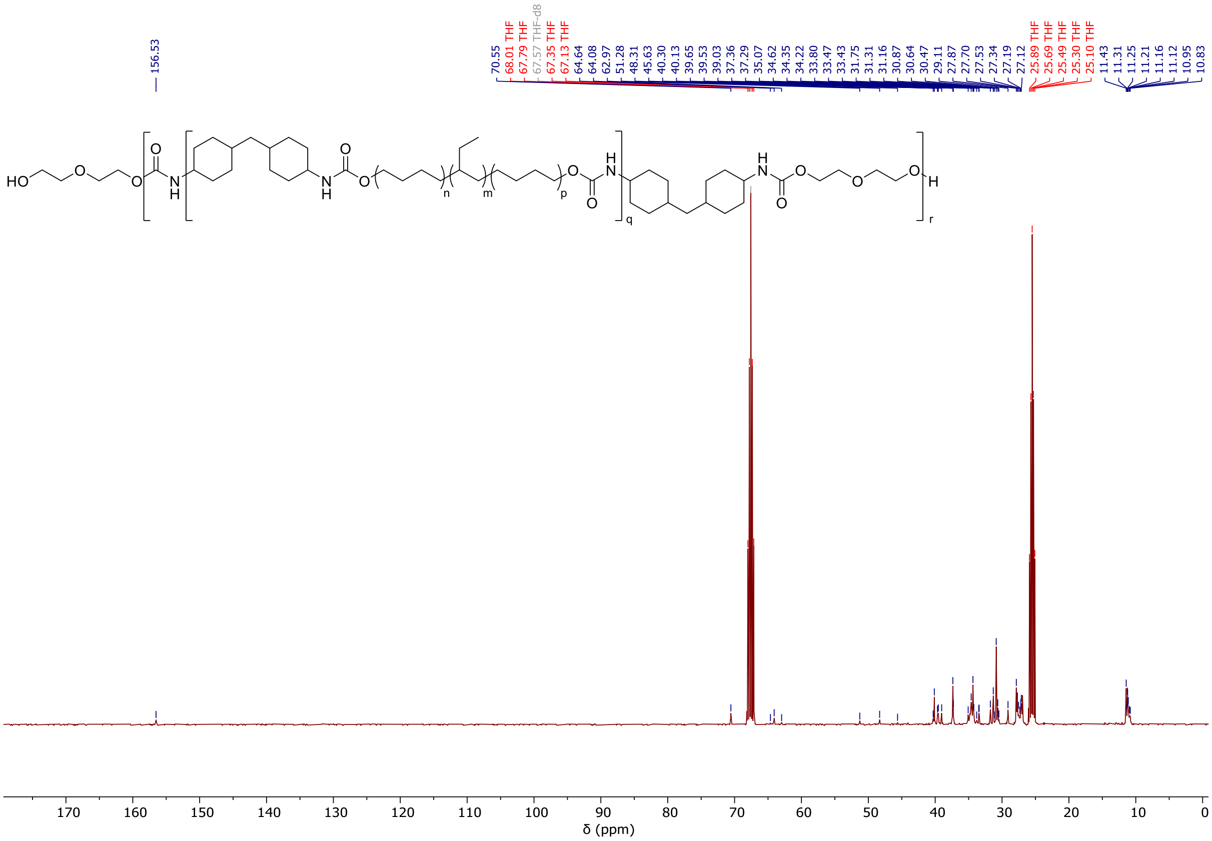

Figure S 16 ^{13}C {H} NMR spectrum of **CEPU4** (100 MHz, THF-d_8, 298 K).

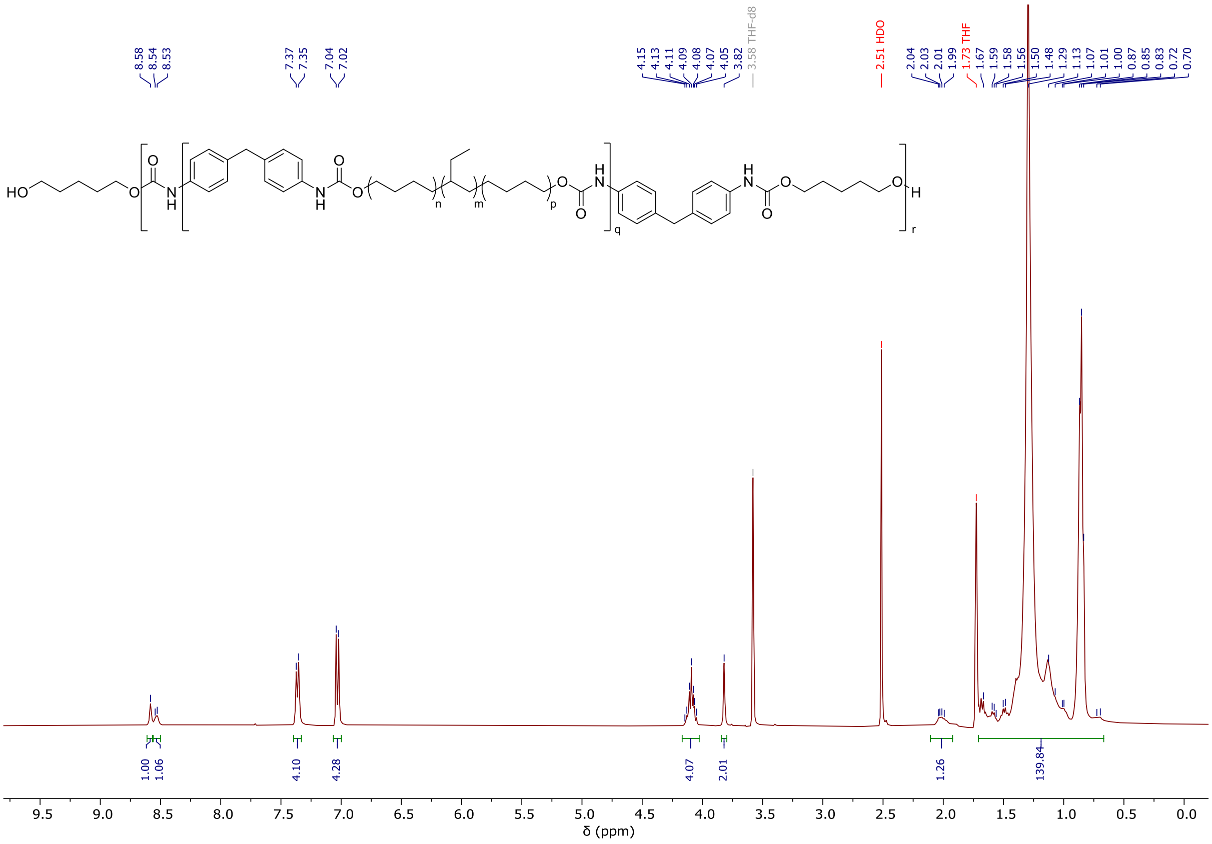

Figure S 17 ^{1}H NMR spectrum of **CEPU5** (400 MHz, THF-d_8, 298 K).

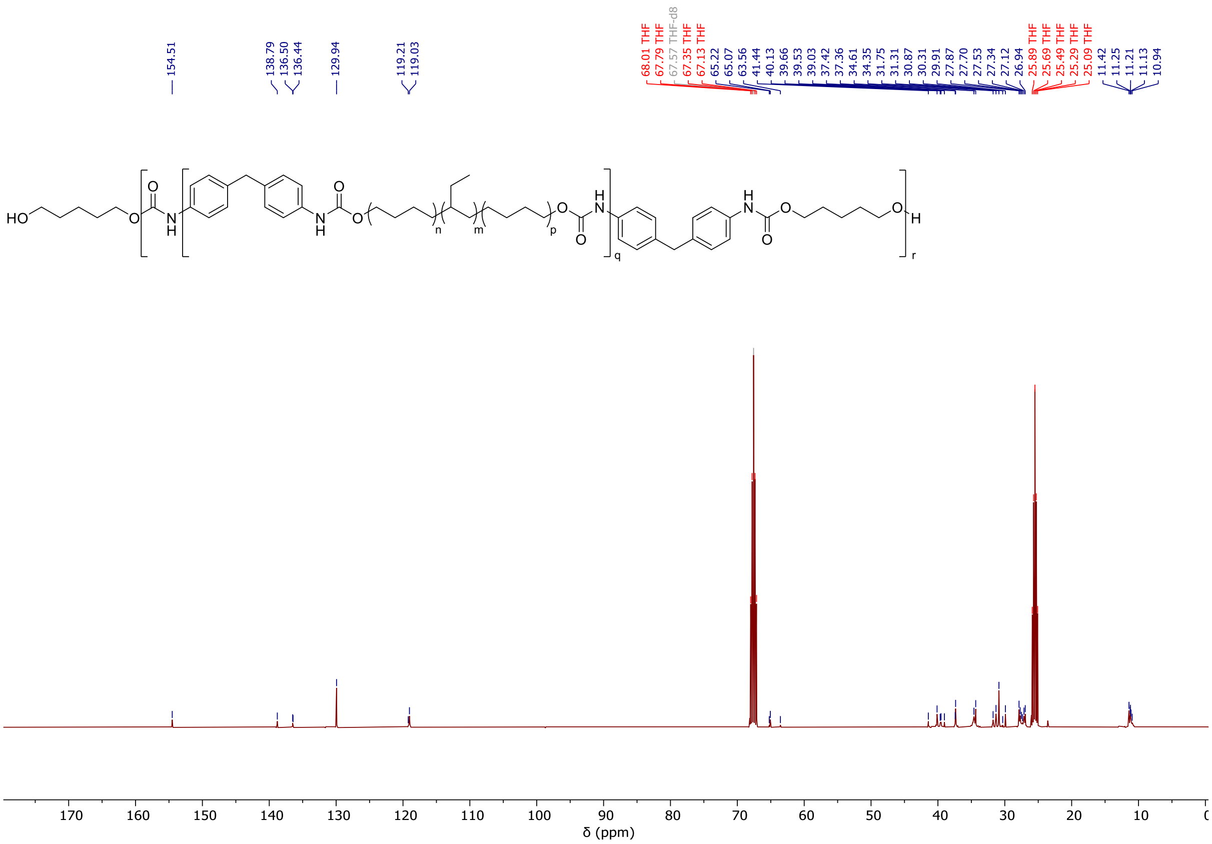

Figure S 18 ^{13}C {H} NMR spectrum of **CEPU5** (100 MHz, THF-d_8, 298 K).

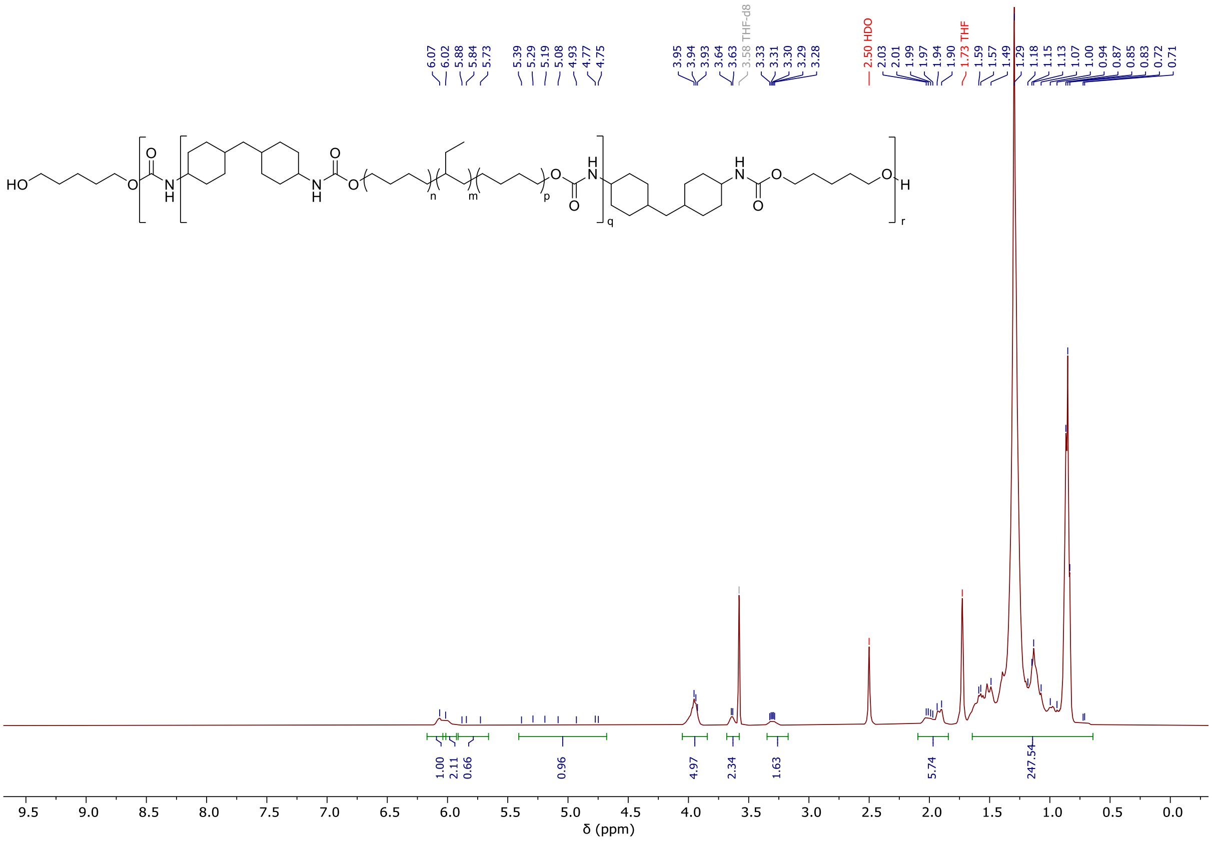

Figure S 19 ^{1}H NMR spectrum of **CEPU6** (400 MHz, THF-d_8, 298 K).

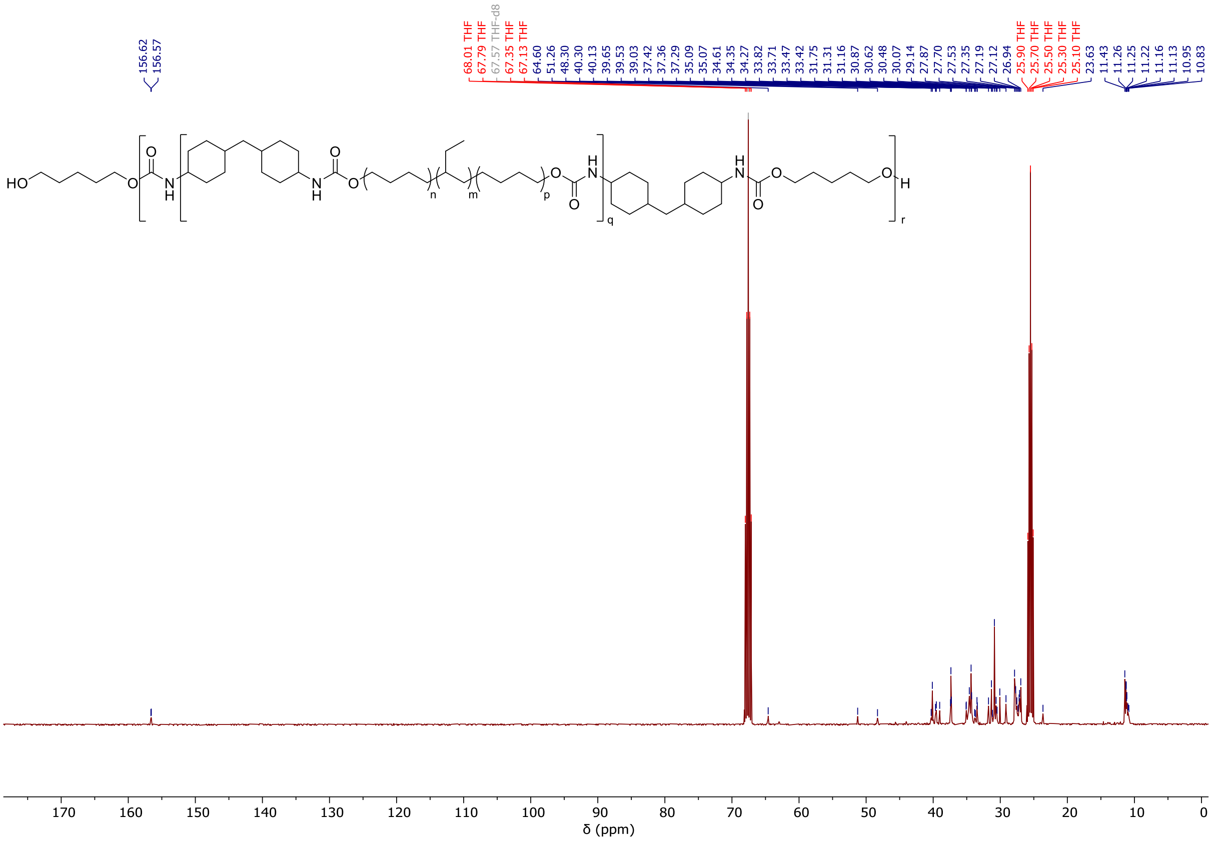

Figure S 20 ^{13}C {H} NMR spectrum of **CEPU6** (100 MHz, THF-d_8, 298 K).

Table S 1 Key ^{1}H NMR and ^{13}C NMR spectroscopic resonances for **CEPU1**-**CEPU6** in THF-d_8.

CEPU adhesive	^{1}H NMR prepolymer urethane resonance (ppm)	^{1}H NMR chain-extender urethane resonance (ppm)	^{1}H NMR chain-extender methylene resonances (ppm)	^{13}C NMR prepolymer urethane resonance (ppm)	^{13}C NMR chain-extender urethane resonance (ppm)
**CEPU1**	8.54	8.82	4.50, 3.43	154.5	154.1
**CEPU2**	6.07-5.95	6.64-6.50	4.33, 3.41-3.26	156.6	156.1
**CEPU3**	8.53	8.73	4.20, 3.70-3.62	154.5	154.4
**CEPU4**	6.01	6.24	4.07, 3.56	156.5	156.4
**CEPU5**	8.54	8.58	4.09	154.4	154.4
**CEPU6**	6.02	6.07	3.95	156.6	156.5

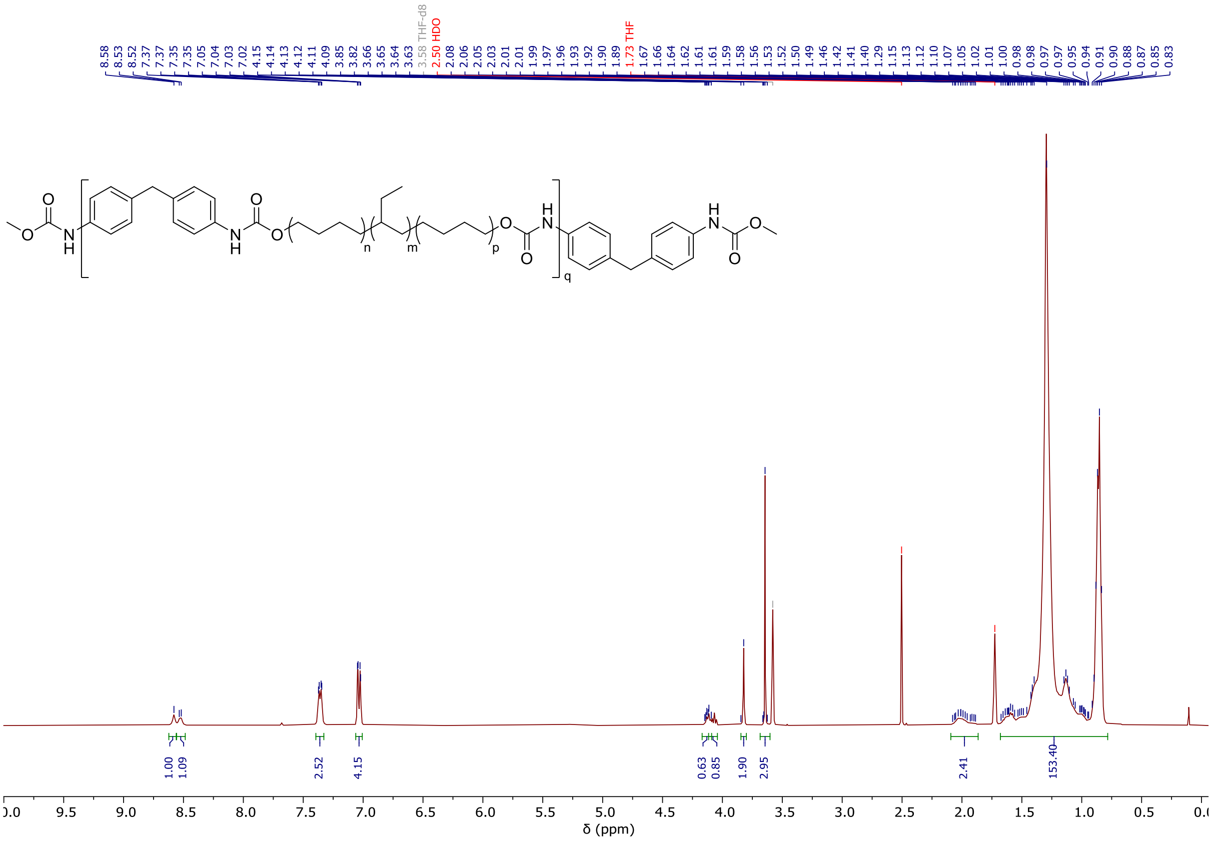

Figure S 21 ^{1}H NMR spectrum of **MeO-PU1** (400 MHz, THF-d_8, 298 K).

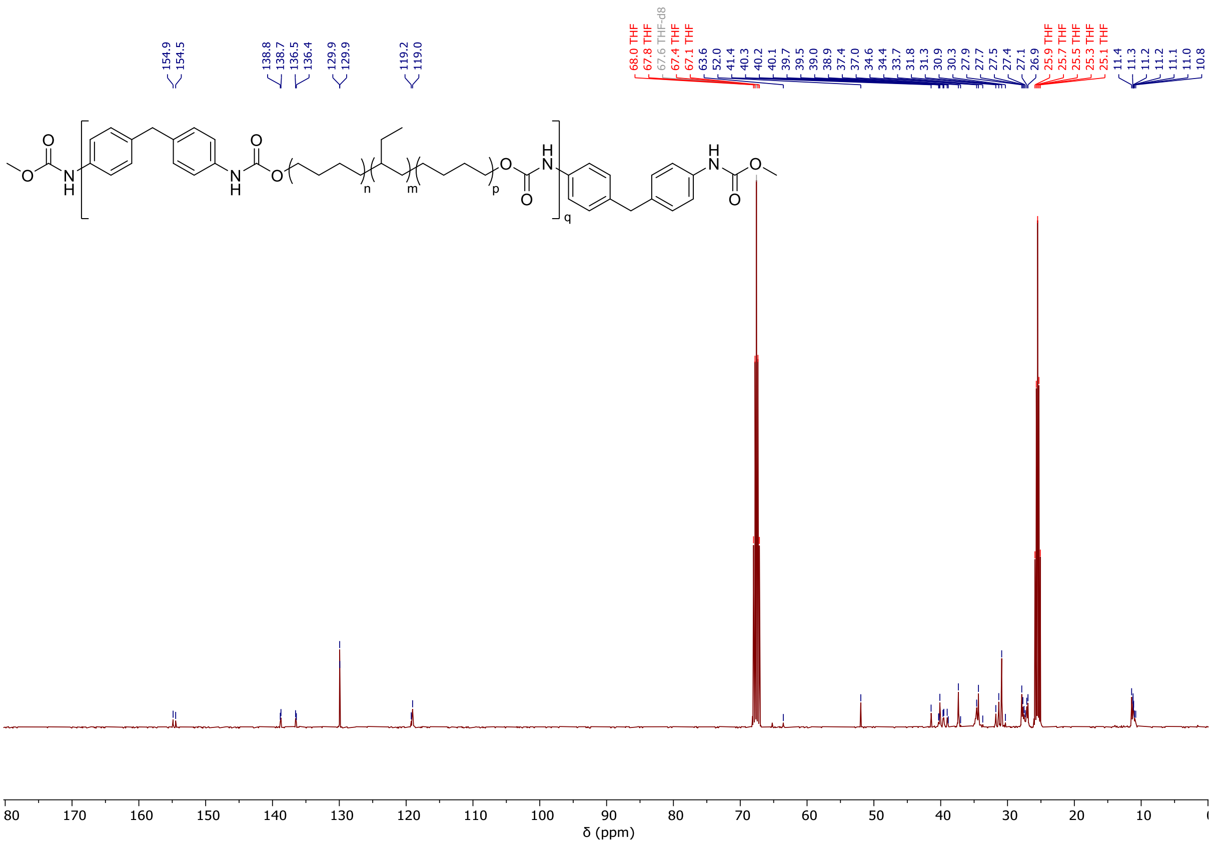

Figure S 22 ^{13}C {H} NMR spectrum of **MeO-PU1** (100 MHz, THF-d_8, 298 K).

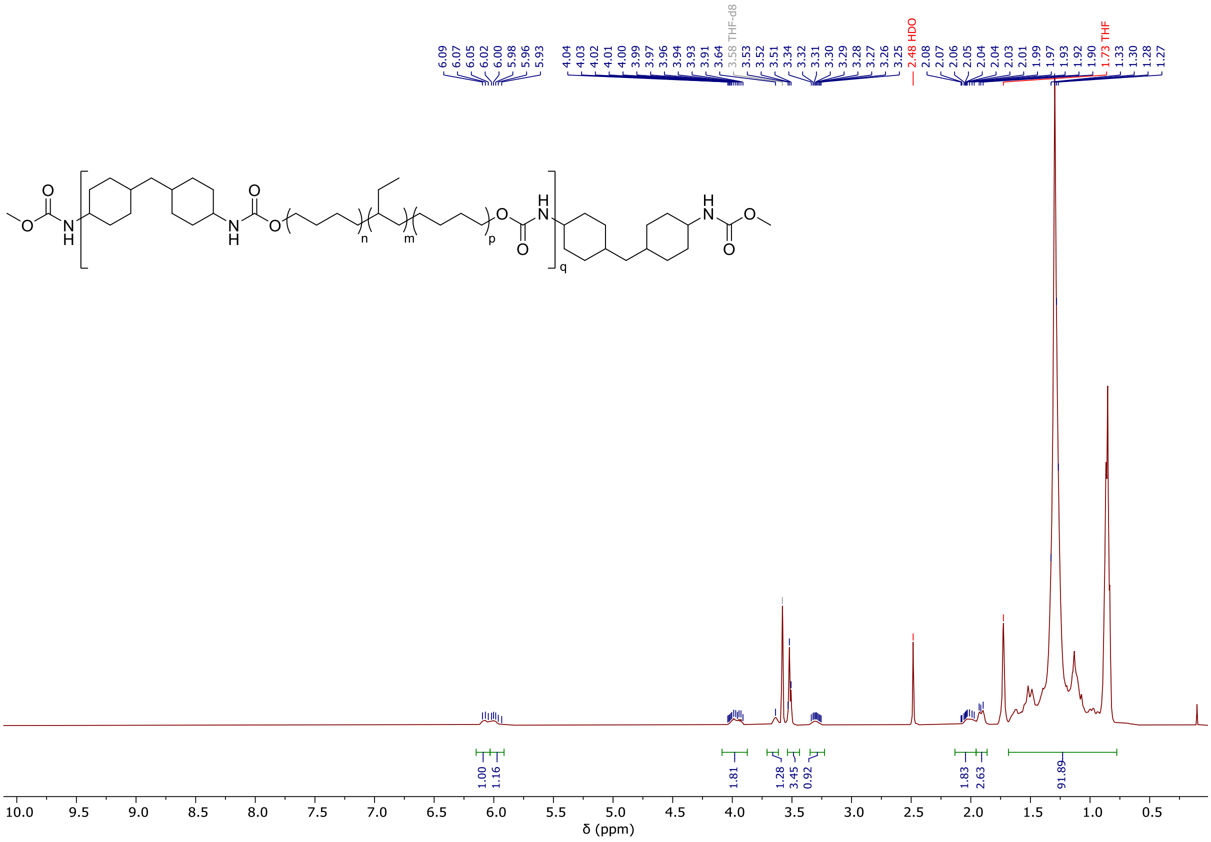

Figure S 23 ^{1}H NMR spectrum of **MeO-PU2** (400 MHz, THF-d_8, 298 K).

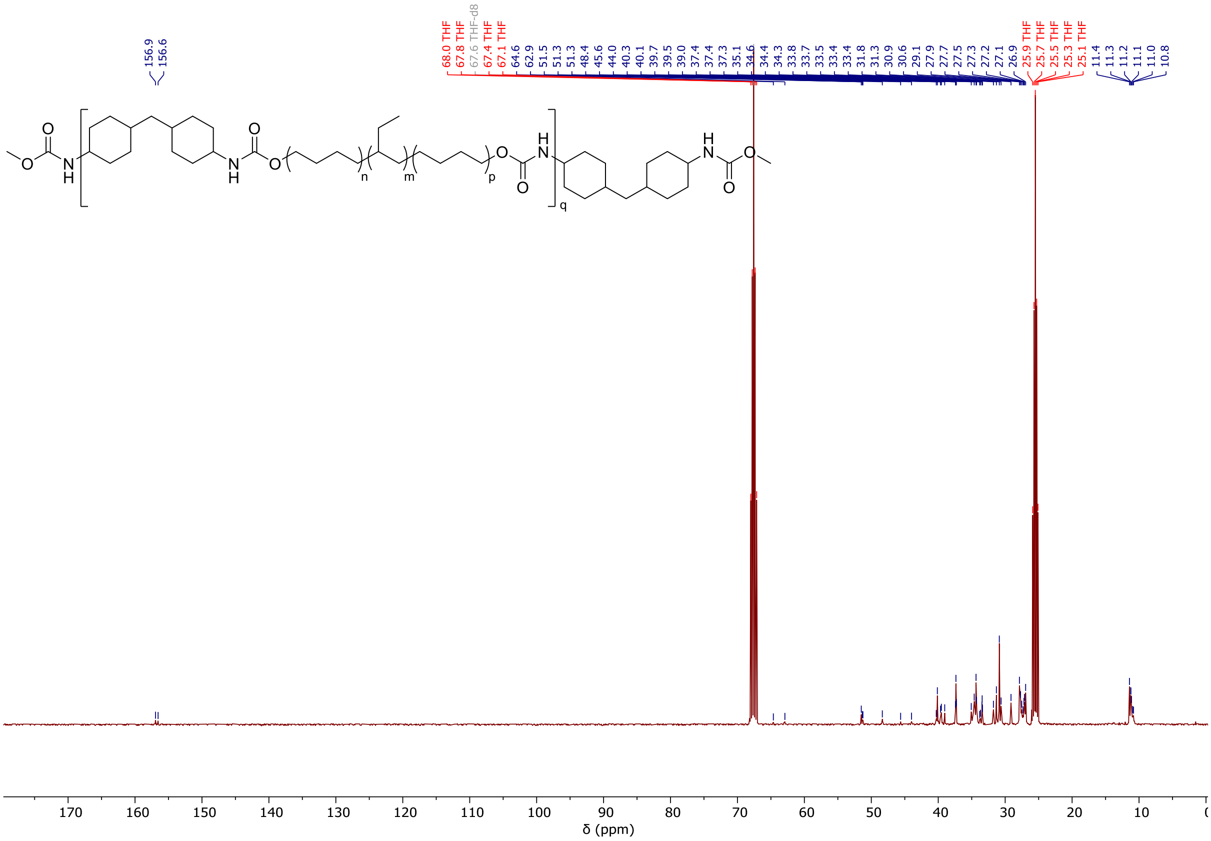

Figure S 24 ^{13}C {H} NMR spectrum of **MeO-PU2** (100 MHz, THF-d_8, 298 K).

Compound **1**

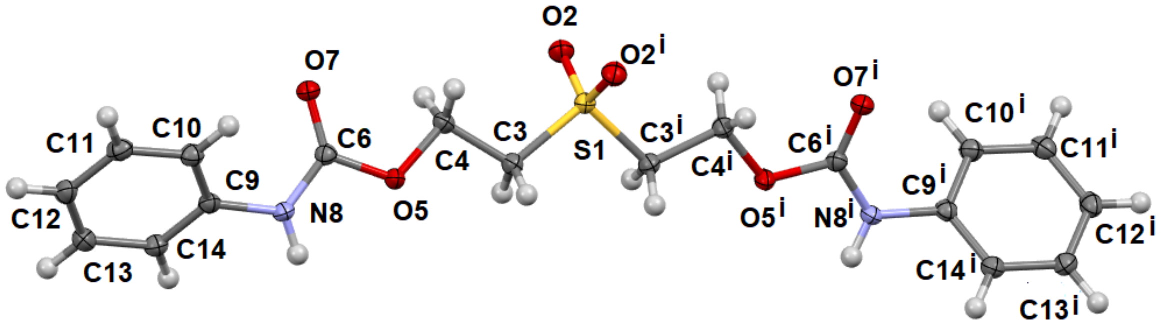

Figure S 25 The molecular structure of **1** obtained from single-crystal X-ray diffraction analysis. Thermal ellipsoids drawn at 50% probability. [symmetry code: (i) 1 – *x*, *y*, ½ – *z*].

Table S 2 Crystallographic details for **1**.

Formula	$C_{18}H_{20}N_2O_6S$
M_r	392.42
Crystal system	monoclinic
Space group	*C* 2/*c*
Z	4
a / Å	28.41439(2)
b / Å	4.98361(1)
c / Å	12.26457(1)
β / °	91.5605(17)
V / Å^3	1736.095(3)
D_{calc} / g cm^{-3}	1.501
Crystal habit	Colourless rod
Crystal dimensions / mm	0.026 × 0.029 × 0.165
Radiation	Cu K_{α} (1.54184 Å)
T / K	100
μ / mm^{-1}	2.021
R(*F*), *Rw*(*F*)	3.33, 4.75
CCDC cif deposition number	CCDC 2279107

Table S 3 Selected bond lengths (Å) and angles (°) in **1**.

S(1) – O(2)	1.4428(10)	O(2) – S(1) – O(2)^{i}	116.95(9)
S(1) – O(2)^{i}	1.4428(10)	O(2) – S(1) – C(3)	109.47(6)
S(1) – C(3)	1.7763(14)	O(2)^{i} – S(1) – C(3)	109.38(6)
S(1) – C(3)^{i}	1.7763(14)	O(2) – S(1) – C(3)^{i}	109.38(6)
C(3) – C(4)	1.5206(18)	O(2)^{i} – S(1) – C(3)^{i}	109.47(6)
C(4) – O(5)	1.4389(16)	C(3) – S(1) – C(3)^{i}	100.99(9)
O(5) – C(6)	1.3673(16)	S(1) – C(3) – C(4)	111.35(10)
C(6) – O(7)	1.2109(17)	C(3) – C(4) – O(5)	104.21(11)
C(6) – N(8)	1.3502(18)	C(4) – O(5) – C(6)	114.03(10)
N(8) – C(9)	1.4272(17)	O(5) – C(6) – O(7)	123.38(12)
C(9) – C(10)	1.3908(19)	O(5) – C(6) – N(8)	109.17(11)
C(9) – C(14)	1.388(2)	O(7) – C(6) – N(8)	127.45(13)
C(10) – C(11)	1.3909(19)	C(6) – N(8) – C(9)	122.40(12)
C(11) – C(12)	1.392(2)	N(8) – C(9) – C(10)	119.96(12)
C(12) – C(13)	1.387(2)	N(8) – C(9) – C(14)	119.41(12)
C(13) – C(14)	1.3931(19)	C(10) – C(9) – C(14)	120.57(13)
		C(9) – C(10) – C(11)	119.67(13)
		C(10) – C(11) – C(12)	120.07(13)
		C(11) – C(12) – C(13)	119.88(13)
		C(12) – C(13) – C(14)	120.37(13)
		C(9) – C(14) – C(13)	119.44(13)

[symmetry code: (i) 1 – *x*, *y*, ½ – *z*].

Table S 4 Hydrogen-bond geometry (Å, °) in **1**.

D–H…A	D–H	H…A	D…A	D–H…A
N(8)^{ii} – H(81)^{ii}...O(7)	0.85(2)	2.16(2)	2.949(2)	155.4(17)

[symmetry code: (ii) *x*, 1 – *y*, *z*].

Table S 5 π-π interactions (Å) in **1**.

C(14)…C(10)^{iii}	3.384(2)
C(13)….C(11)^{iii}	3.392(2)

[symmetry code: (iii) *x*, 1 + *y*, *z*].

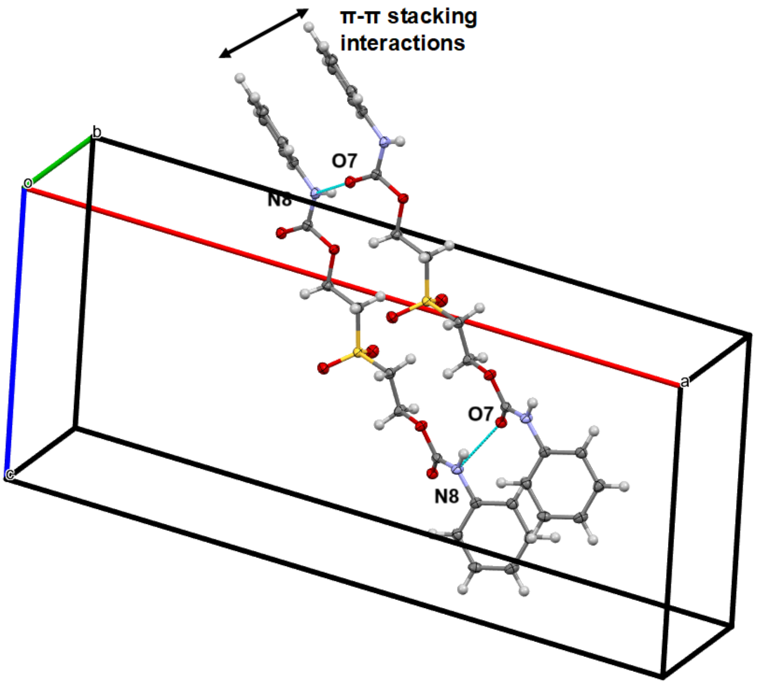

Figure S 26 Packing diagram for compound **1**.

Hydrogen bond (shown as pale- blue lines) between N-H and O=C groups of carbamate groups in adjacent molecules (N(8)…O(7), 2.949(2) Å). π-π stacking interactions occur between the phenyl groups in adjacent molecules.

Compound **2**

Molecule **A**

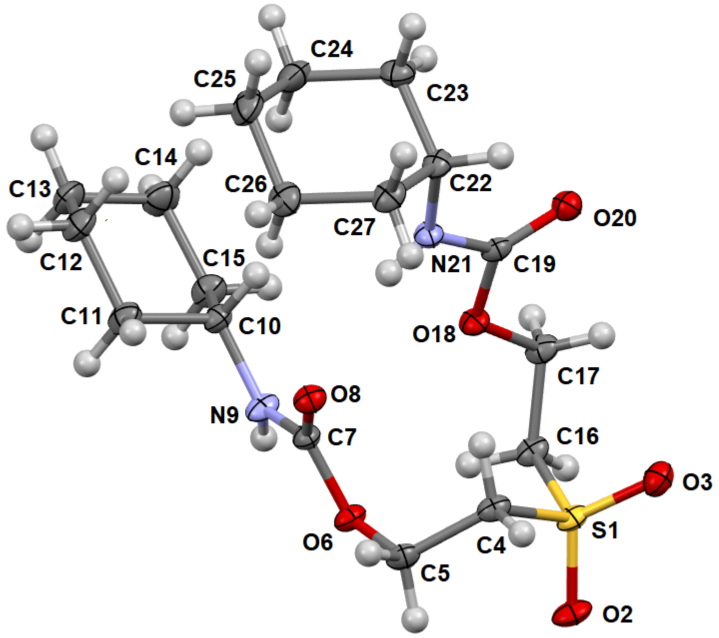

Molecule **B**

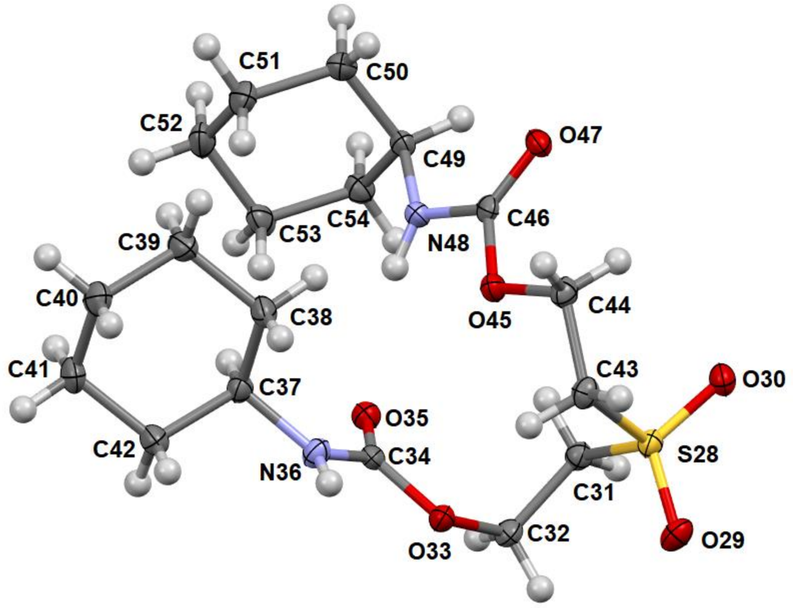

Figure S 27 The asymmetric unit of **2** obtained from single-crystal X-ray diffraction analysis contains two distinct molecules (**A** and **B**). Atom labelling schemes are shown for each of the molecules. Thermal ellipsoids drawn at 50% probability.

Table S 6 Crystallographic details for **2**.

Formula	$C_{36} H_{64} N_4 O_{12} S_2$
M_r	809.06
Crystal system	triclinic
Space group	*P* -1
Z	2
a / Å	10.35289(1)
b / Å	14.32739(1)
c / Å	4.48300(1)
β / °	91.7943(18)
V / Å^3	2030.00(3)
D_{calc} / g cm^{-3}	1.324
Crystal habit	Colourless plate
Crystal dimensions / mm	0.023 × 0.051 × 0.121
Radiation	Cu K_α (1.54184 Å)
T /K	100
μ / mm^{-1}	1.739
R(*F*), *Rw*(*F*)	3.80, 5.29
CCDC cif deposition number	CCDC 2279109

Table S 7 Selected bond lengths (Å) and angles (°) in **2**.

S(1) – O(2)	1.4497(11)	O(2) – S(1) – O(3)	118.13(7)
S(1) – O(3)	1.4417(13)	O(2) – S(1) – C(4)	107.10(7)
S(1) – C(4)	1.7806(15)	O(3) – S(1) – C(4)	107.52(7)
S(1) – C(16)	1.7874(16)	O(2) – S(1) – C(16)	107.34(7)
S(28) – O(29)	1.4471(12)	O(3) – S(1) – C(16)	107.44(7)
S(28) – O(30)	1.4439(12)	C(4) – S(1) – C(16)	109.08(7)
S(28) – C(31)	1.7789(16)	O(29) – S(28) – O(30)	117.97(7)
S(28) – C(43)	1.7836(16)	O(29) – S(28) – C(31)	107.43(7)
O(6) – C(5)	1.4393(18)	O(30) – S(28) – C(31)	107.43(7)
O(6) – C(7)	1.3692(18)	O(29) – S(28) – C(43)	107.44(7)
O(8) – C(7)	1.2198(19)	O(30) – S(28) – C(43)	107.19(7)
O(18) – C(17)	1.4447(18)	C(31) – S(28) – C(43)	109.17(7)
O(18) – C(19)	1.3688(18)	C(5) – O(6) – C(7)	116.13(12)
O(20) – C(19)	1.2159(19)	C(17) – O(18) – C(19)	116.64(12)
O(33) – C(32)	1.4389(19)	C(32) – O(33) – C(34)	116.46(12)
O(33) – C(34)	1.3714(18)	C(44) – O(45) – C(46)	117.01(12)
O(35) – C(34)	1.2138(19)	C(7) – N(9) – C(10)	122.65(13)
O(45) – C(44)	1.4404(18)	C(19) – N(21) – C(22)	120.83(13)
O(45) – C(46)	1.3602(19)	C(34) – N(36) – C(37)	123.58(14)
O(47) – C(46)	1.2225(19)	C(46) – N(48) – C(49)	121.13(13)
N(9) – C(7)	1.333(2)	S(1) – C(4) – C(5)	114.55(11)

N(9) – C(10)	1.457(2)	O(6) – C(5) – C(4)	113.34(12)
N(21) – C(19)	1.339(2)	O(6) – C(7) – O(8)	123.43(14)
N(21) – C(22)	1.4696(19)	O(6) – C(7) – N(9)	109.82(13)
N(36) – C(34)	1.336(2)	O(8) – C(7) – N(9)	126.74(14)
N(36) – C(37)	1.461(2)	N(9) – C(10) – C(11)	112.31(13)
N(48) – C(46)	1.340(2)	N(9) – C(10) – C(15)	108.48(13)
N(48) – C(49)	1.466(2)	C(11) – C(10) – C(15)	111.04(13)
C(4) – C(5)	1.519(2)	C(10) – C(11) – C(12)	110.61(14)
C(10) – C(11)	1.525(2)	C(11) – C(12) – C(13)	111.05(15)
C(10) – C(15)	1.520(2)	C(12) – C(13) – C(14)	111.32(15)
C(11) – C(12)	1.528(2)	C(13) – C(14) – C(15)	111.48(14)
C(12) – C(13)	1.522(3)	C(10) – C(15) – C(14)	112.06(14)
C(13) – C(14)	1.526(3)	S(1) – C(16) – C(17)	113.56(11)
C(14) – C(15)	1.525(2)	O(18) – C(17) – C(16)	107.32(12)
C(16) – C(17)	1.513(2)	O(18) – C(19) – O(20)	123.37(14)
C(22) – C(23)	1.533(2)	O(18) – C(19) – N(21)	109.59(13)
C(22) – C(27)	1.527(2)	O(20) – C(19) – N(21)	127.01(14)
C(23) – C(24)	1.525(2)	N(21) – C(22) – C(23)	110.42(13)
C(24) – C(25)	1.530(3)	N(21) – C(22) – C(27)	109.52(13)
C(25) – C(26)	1.525(3)	C(23) – C(22) – C(27)	110.97(13)
C(26) – C(27)	1.529(2)	C(22) – C(23) – C(24)	112.32(14)
C(31) – C(32)	1.519(2)	C(23) – C(24) – C(25)	111.34(15)
C(37) – C(38)	1.528(2)	C(24) – C(25) – C(26)	110.88(14)
C(37) – C(42)	1.525(2)	C(25) – C(26) – C(27)	111.32(15)
C(38) – C(39)	1.528(2)	C(22) – C(27) – C(26)	112.58(14)
C(39) – C(40)	1.529(2)	S(28) – C(31) – C(32)	114.41(11)
C(40) – C(41)	1.518(3)	O(33) – C(32) – C(31)	113.29(12)
C(41) – C(42)	1.531(2)	O(33) – C(34) – O(35)	123.72(14)
C(43) – C(44)	1.511(2)	O(33) – C(34) – N(36)	108.68(13)
C(49) – C(50)	1.534(2)	O(35) – C(34) – N(36)	127.60(15)
C(49) – C(54)	1.526(2)	N(36) – C(37) – C(38)	110.26(13)
C(50) – C(51)	1.528(2)	N(36) – C(37) – C(42)	109.11(13)
C(51) – C(52)	1.526(3)	C(38) – C(37) – C(42)	110.56(13)
C(52) – C(53)	1.521(3)	C(37) – C(38) – C(39)	111.87(13)
C(53) – C(54)	1.528(2)	C(38) – C(39) – C(40)	110.96(14)
		C(39) – C(40) – C(41)	111.12(14)
		C(40) – C(41) – C(42)	111.62(14)
		C(37) – C(42) – C(41)	112.07(14)
		S(28) – C(43) – C(44)	113.52(11)
		O(45) – C(44) – C(43)	107.04(12)
		O(45) – C(46) – O(47)	123.69(14)
		O(45) – C(46) – N(48)	109.73(13)
		O(47) – C(46) – N(48)	126.56(15)
		N(48) – C(49) – C(50)	110.76(13)
		N(48) – C(49) – C(54)	108.82(12)
		C(50) – C(49) – C(54)	111.12(13)

		C(49) – C(50) – C(51)	112.24(13)
		C(50) – C(51) – C(52)	111.18(15)
		C(51) – C(52) – C(53)	110.74(14)
		C(52) – C(53) – C(54)	110.85(14)
		C(49) – C(54) – C(53)	112.38(13)

Table S 8 Hydrogen-bond and close-contact geometry (Å, °) in **2**.

D–H…A	D–H	H…A	D…A	D–H…A
C(4) – H(41)...O(18)	0.95	2.47	3.142(2)	128
C(5) – H(51)...O(47)	0.95	2.47	3.371(2)	158
C(16) – H(162)...O(6)	0.95	2.47	3.176(2)	131
$C(31)^{ii}$ – $H(311)^{ii}$...O(47)	0.95	2.52	3.359(2)	147
C(31) – H(312)...O(45)	0.95	2.46	3.147(2)	129
$C(32)^{iii}$ – $H(321)^{iii}$...O(19)	0.95	2.60	3.398(2)	142
C(43) – H(432)...O(33)	0.95	2.51	3.206(2)	130
$C(44)^{iv}$ – $H(442)^{iv}$...O(20)	0.95	2.53	3.249(2)	133
N(21) – H(211)...O(8)	0.81(2)	2.18(2)	2.964(2)	165(2)
N(48) – H(481)...O(35)	0.84(2)	2.21(2)	3.025(2)	166.7(20)
$N(36)^{v}$ – $H(361)^{v}$...O(29)	0.81(2)	2.14(2)	2.947(2)	178(2)
$N(9)^{vi}$ – $H(91)^{vi}$...O(2)	0.79(2)	2.15(2)	2.947(2)	178(2)

[symmetry codes: (ii) – *x*, 1 – *y*, – *z*; (iii) *x*, *y*, 1– *z*; (iv) 1– *x*, 1 – *y*, 1– *z*; (v) 1– *x*, 1 – *y*, – *z*; (vi) – *x*, 1 – *y*, 1 – *z*].

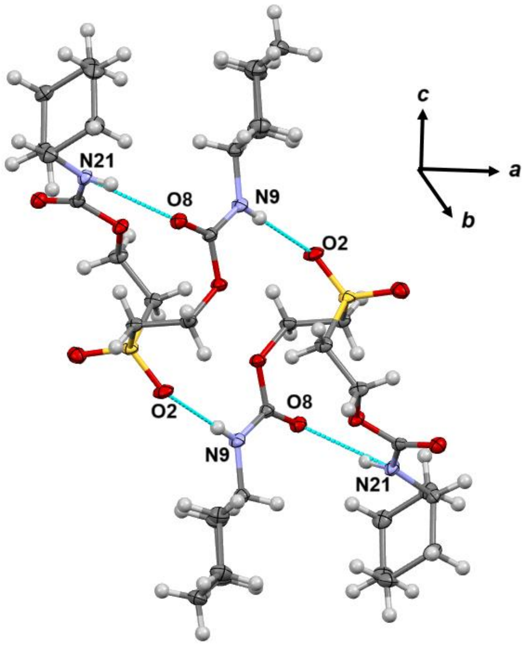

Hydrogen bonding interactions (in pale blue) between adjacent A molecules in Compound **2**

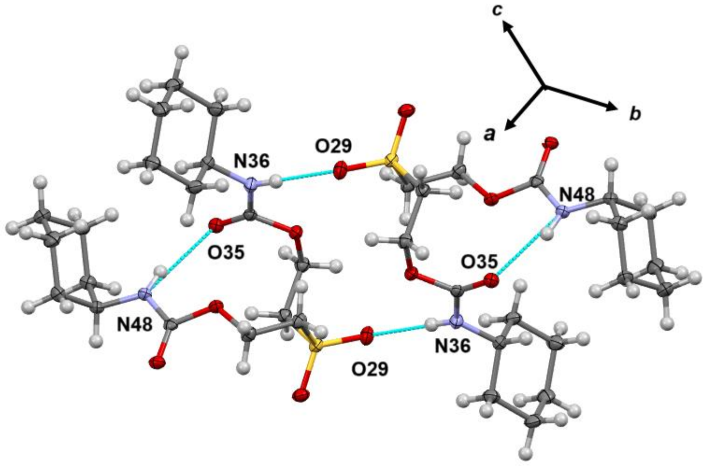

Hydrogen bonding interactions (in pale blue) between adjacent B molecules in Compound **2**

Figure S 28 Packing diagrams for compound **2**.

Compound **3**

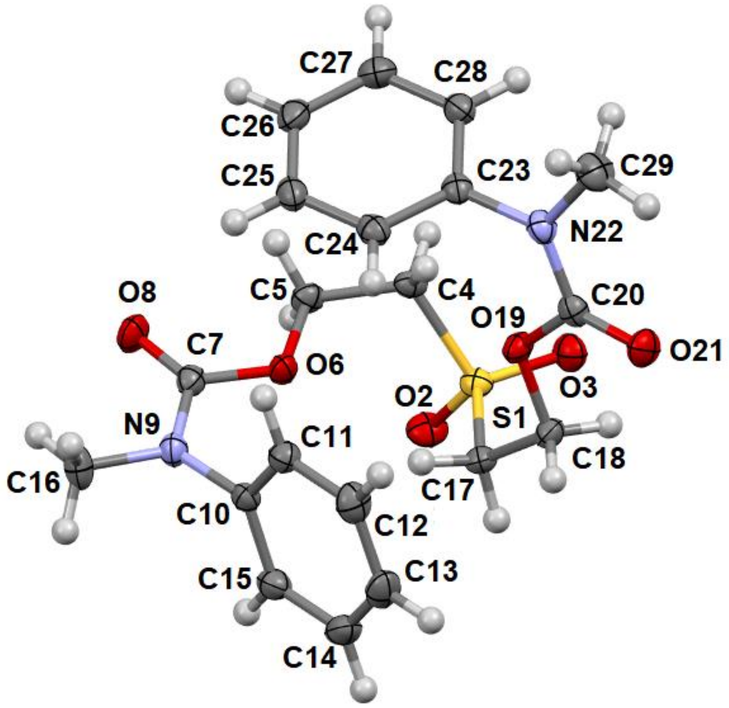

Figure S 29 The molecular structure of **3** obtained from single-crystal X-ray diffraction analysis. Thermal ellipsoids drawn at 50% probability.

Table S 9 Crystallographic details for **3**.

Formula	$C_{20}H_{24}N_2O_6S$
M_r	392.42
Crystal system	orthorhombic
Space group	P bca
Z	8
a / Å	15.96447(1)
b / Å	8.42739(1)
c / Å	30.61785(1)
V / Å^3	4119.289(5)
D_{calc} / g cm^{-3}	1.356
Crystal habit	Colourless plate
Crystal dimensions / mm	0.055 × 0.142 × 0.227
Radiation	Cu K_α (1.54184 Å)
T / K	100
μ / mm^{-1}	1.739
R(*F*), *Rw*(*F*)	2.97, 4.22
CCDC cif deposition number	CCDC 2279110

Table S 10 Selected bond lengths (Å) and angles (°) in **3**.

S(1) – O(2)	1.4462(9)	O(2) – S(1) – O(3)	118.32(6)
S(1) – O(3)	1.4443(9)	O(2) – S(1) – C(4)	107.36(6)
S(1) – C(4)	1.7823(12)	O(3) – S(1) – C(4)	107.72(6)
S(1) – C(17)	1.7871(12)	O(2) – S(1) – C(17)	107.29(6)
C(4) – C(5)	1.5074(17)	O(3) – S(1) – C(17)	107.39(6)
C(5) – O(6)	1.4434(14)	C(4) – S(1) – C(17)	108.43(5)
O(6) – C(7)	1.3525(14)	S(1) – C(4) – C(5)	113.69(8)
C(7) – O(8)	1.2186(14)	C(4) – C(5) – O(6)	107.29(9)
C(7) – N(9)	1.3533(16)	C(5) – O(6) – C(7)	115.20(9)
N(9) – C(10)	1.4397(15)	O(6) – C(7) – O(8)	123.03(11)
N(9) – C(16)	1.4611(16)	O(6) – C(7) – N(9)	111.44(10)
C(10) – C(11)	1.3865(17)	O(8) – C(7) – N(9)	125.52(11)
C(10) – C(15)	1.3883(17)	C(7) – N(9) – C(10)	122.17(10)
C(11) – C(12)	1.3900(18)	C(7) – N(9) – C(16)	119.56(10)
C(12) – C(13)	1.390(2)	C(10) – N(9) – C(16)	117.24(10)
C(13) – C(14)	1.384(2)	N(9) – C(10) – C(11)	119.48(11)
C(14) – C(15)	1.3949(18)	N(9) – C(10) – C(15)	119.97(11)
C(17) – C(18)	1.5101(16)	C(11) – C(10) – C(15)	120.50(11)
C(18) – O(19)	1.4404(14)	C(10) – C(11) – C(12)	119.88(12)
O(19) – C(20)	1.3578(14)	C(11) – C(12) – C(13)	119.83(13)
C(20) – O(21)	1.2143(15)	C(12) – C(13) – C(14)	120.20(12)
C(20) – N(22)	1.3579(16)	C(13) – C(14) – C(15)	120.16(12)
N(22) – C(23)	1.4386(15)	C(10) – C(15) – C(14)	119.41(12)
N(22) – C(29)	1.4570(16)	S(1) – C(17) – C(18)	114.29(8)
C(23) – C(24)	1.3891(17)	C(17) – C(18) – O(19)	107.14(9)
C(23) – C(28)	1.3882(17)	C(18) – O(19) – C(20)	115.47(9)
C(24) – C(25)	1.3886(18)	O(19) – C(20) – O(21)	123.26(11)
C(25) – C(26)	1.3881(19)	O(19) – C(20) – N(22)	110.81(10)
C(26) – C(27)	1.3892(19)	O(21) – C(20) – N(22)	125.91(11)
C(27) – C(28)	1.3923(18)	C(20) – N(22) – C(23)	122.19(10)
		C(20) – N(22) – C(29)	120.02(10)
		C(23) – N(22) – C(29)	117.77(10)
		N(22) – C(23) – C(24)	119.67(11)
		N(22) – C(23) – C(28)	119.72(11)
		C(24) – C(23) – C(28)	120.60(11)
		C(23) – C(24) – C(25)	119.69(12)
		C(24) – C(25) – C(26)	120.00(12)
		C(25) – C(26) – C(27)	120.14(12)
		C(26) – C(27) – C(28)	120.07(12)
		C(23) – C(28) – C(27)	119.44(12)

Table S 11 Hydrogen bond and close-contact geometry (Å, °) in **3**.

D–H…A	D–H	H…A	D…A	D–H…A
C(4)^{i} – H(41)^{i}...O(8)	0.95	2.44	3.1973(18)	136
C(4) – H(42)...O(19)	0.95	2.447	3.1473(18)	131
C(14)^{ii} – H(141)^{ii}...O(21)	0.95	2.58	3.3759(18)	142
C(17) – H(171)...O(6)	0.95	2.46	3.1557(18)	130

[symmetry codes: (i) 1– *x*, *y* –1/2, – *z* + 3/2; (ii) x+1/2, –y + 3/2,1 – z].

Table S 12 π-π interactions (Å) in **3**.

C(24)…C(28)^{iii}	3.681(2)
C(25)….C(27)^{iii}	3.858(2)

[symmetry code: (iii) 1/2 – *x*, 1/2 + *y*, *z*].

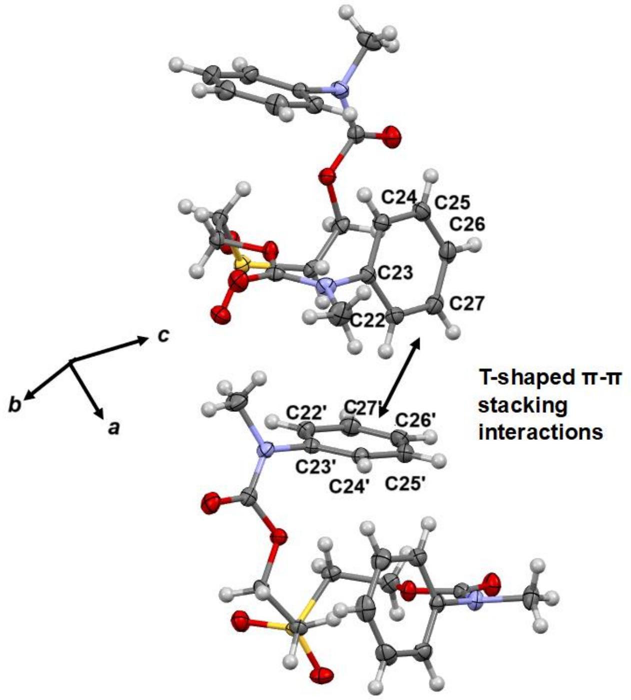

Figure S 30 Packing diagram for compound **3**.

T shaped π-π stacking interactions shown between aromatic rings in adjacent molecules

Compound **4**

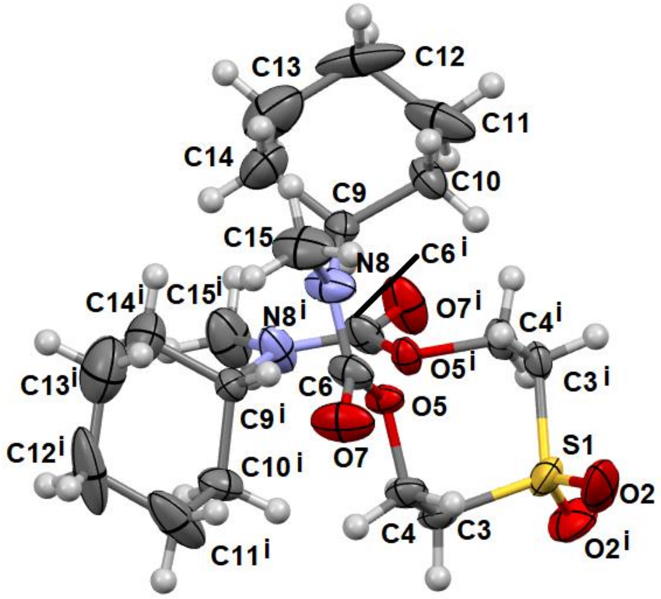

Figure S 31 The molecular structure of **4** obtained from single-crystal X-ray diffraction analysis. Thermal ellipsoids drawn at 50% probability. [symmetry code: (i) 1 – *x*, 1 – *y*, *z*].

Table S 13 Crystallographic details for **4**.

Formula	$C_{18}\,H_{20}\,N_2\,O_6S$
M_r	392.42
Crystal system	orthorhombic
Space group	F d d 2
Z	8
a / Å	28.69545(2)
b / Å	15.85459(1)
c / Å	9.85925(1)
V / Å^3	4485.511(7)
D_{calc} / g cm^{-3}	1.281
Crystal habit	Colourless block
Crystal dimensions / mm	0.070 × 0.081 × 0.151
Radiation	Cu K_α (1.54184 Å)
T / K	100
μ / mm^{-1}	1.598
R(*F*), *Rw*(*F*)	5.96, 6.98
CCDC cif deposition number	CCDC 2279111

Table S 14 Selected bond lengths (Å) and angles (°) in **4**.

S(1) – O(2)	1.437(3)	O(2) – S(1) – O(2)^{i}	119.0(3)
S(1) – O(2)^{i}	1.437(3)	O(2^{i} – S(1) – C(3)^{i}	107.98(17)
S(1) – C(3)	1.778(3)	O(2) – S(1) – C(3)^{i}	106.90(17)
S(1) – C(3)^{i}	1.778(3)	O(2) – S(1) – C(3)	107.98(17)
C(3) – C(4)	1.509(4)	C(3) – S(1) – C(3)^{i}	107.6(2)
C(4) – O(5)	1.446(4)	S(1) – C(3) – C(4)	114.4(2)
O(5) – C(6)	1.359(4)	C(3) – C(4) – O(5)	107.4(2)
C(6) – O(7)	1.225(4)	C(4) – O(5) – C(6)	112.9(2)
C(6) – N(8)	1.341(5)	O(5) – C(6) – O(7)	121.9(3)
N(8) – C(9)	1.492(4)	O(5) – C(6) – N(8)	112.1(3)
N(8) – C(15)	1.467(5)	O(7) – C(6) – N(8)	126.0(3)
C(9) – C(10)	1.507(4)	C(6) – N(8) – C(9)	124.0(3)
C(9) – C(14)	1.501(5)	C(6) – N(8) – C(15)	115.5(3)
C(10) – C(11)	1.544(6)	C(9) – N(8) – C(15)	120.2(3)
C(11) – C(12)	1.529(9)	N(8) – C(9) – C(10)	109.9(3)
C(12) – C(13)	1.537(9)	N(8) – C(9) – C(14)	110.4(3)
C(13) – C(14)	1.465(7)	C(10) – C(9) – C(14)	112.9(3)
		C(9) – C(10) – C(11)	108.7(3)
		C(10) – C(11) – C(12)	111.3(4)
		C(11) – C(12) – C(13)	111.1(4)
		C(12) – C(13) – C(14)	112.6(5)
		C(9) – C(14) – C(13)	109.5(4)

[symmetry code: (i) 1 – *x*, 1 – *y*, *z*].

Table S 15 Hydrogen-bond and close-contact geometry (Å, °) in **4**.

D–H…A	D–H	H…A	D…A	D–H…A
C(3)^{i} – H(31)^{i}...O(5)	0.95	2.48	3.192(6)	132
C(3)^{ii} – H(31)^{ii}...O(7)	0.95	2.36	3.102(6)	135

[symmetry code: (i) 1 – *x*, 1 – *y*, *z*; (ii) 3/4 – *x*, 1/4 + *y*, *z* – 1/4].

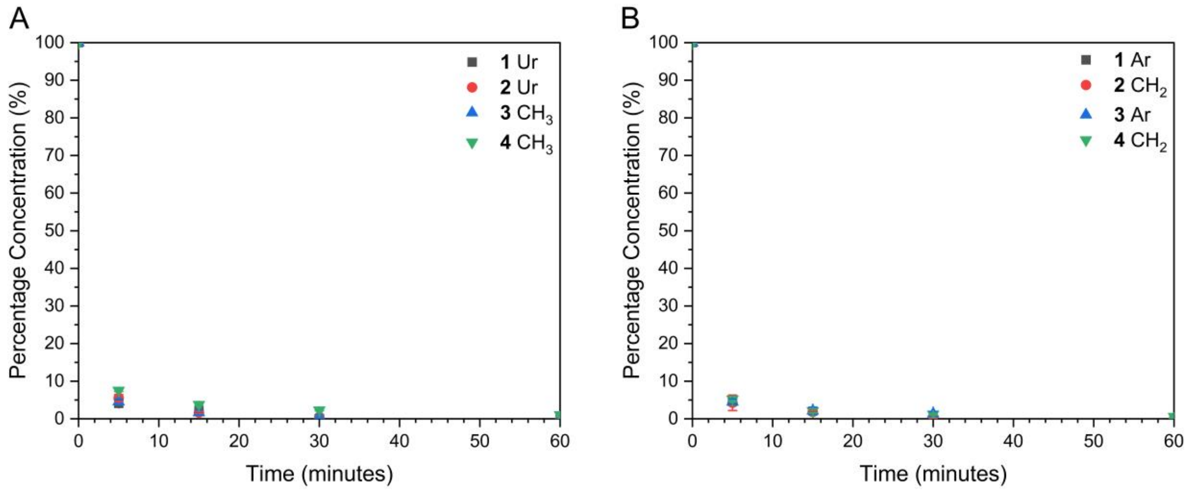

Figure S 32 Degradation kinetics of model urethanes **1-4** calculated from the ^{1}H NMR spectra (20 °C) obtained following the addition of 5 molar equiv. of 1 M TBAF in THF to a 10 mg mL^{-1} solution of model urethane in MeCN-d_3. **A** Urethane hydrogen resonance (**1** and **2**) and N-methyl urethane hydrogen resonance (**3** and **4**); **B** Aromatic hydrogen resonance (**1** and **3**) and methylene resonance (**2** and **4**). The error shown is the standard deviation between the three repeats for each sample.

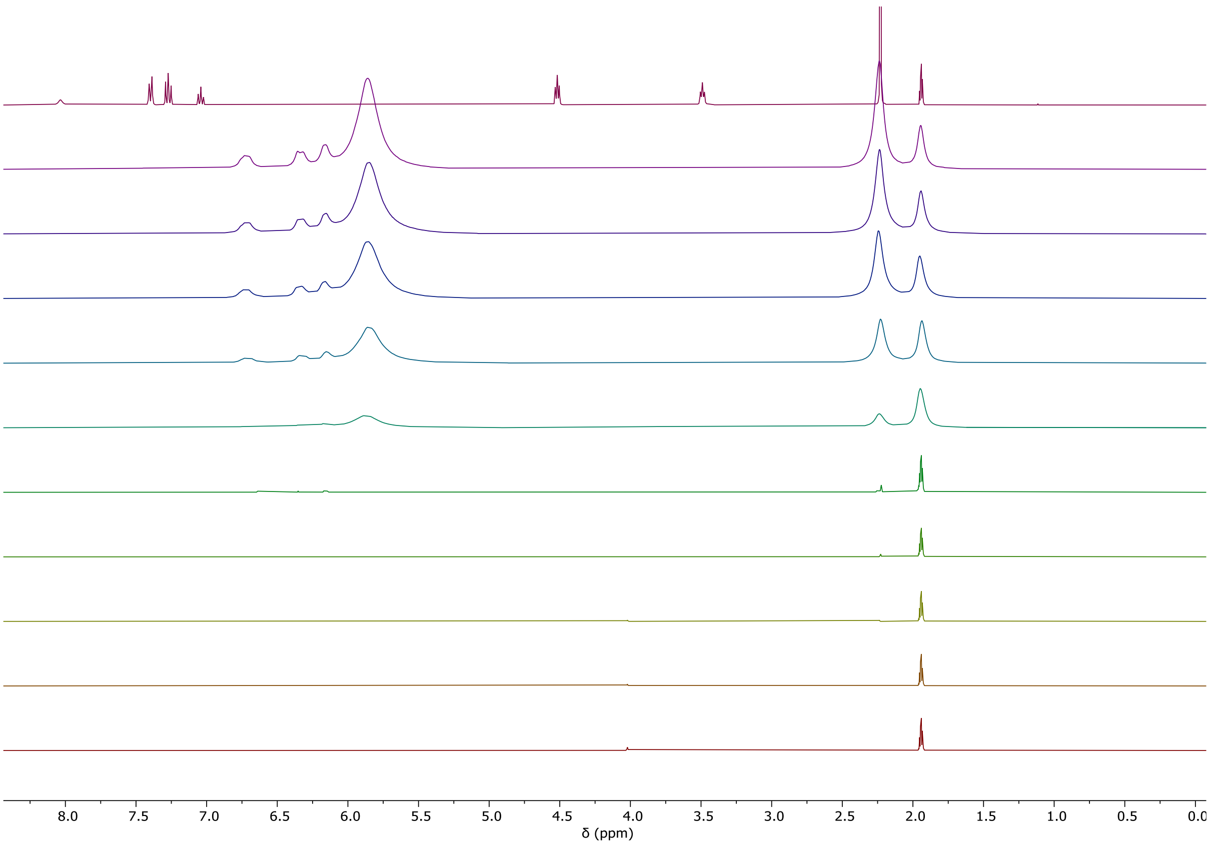

Figure S 33 ^{1}H NMR spectra recorded overtime following the addition of 5 molar equiv. of 40 wt.% NaOD in D_2O to a 10 mg mL^{-1} solution of model urethane **1** (400 MHz, MeCN-d_3). Descending from T=0 minutes to T=10080 minutes (1 week).

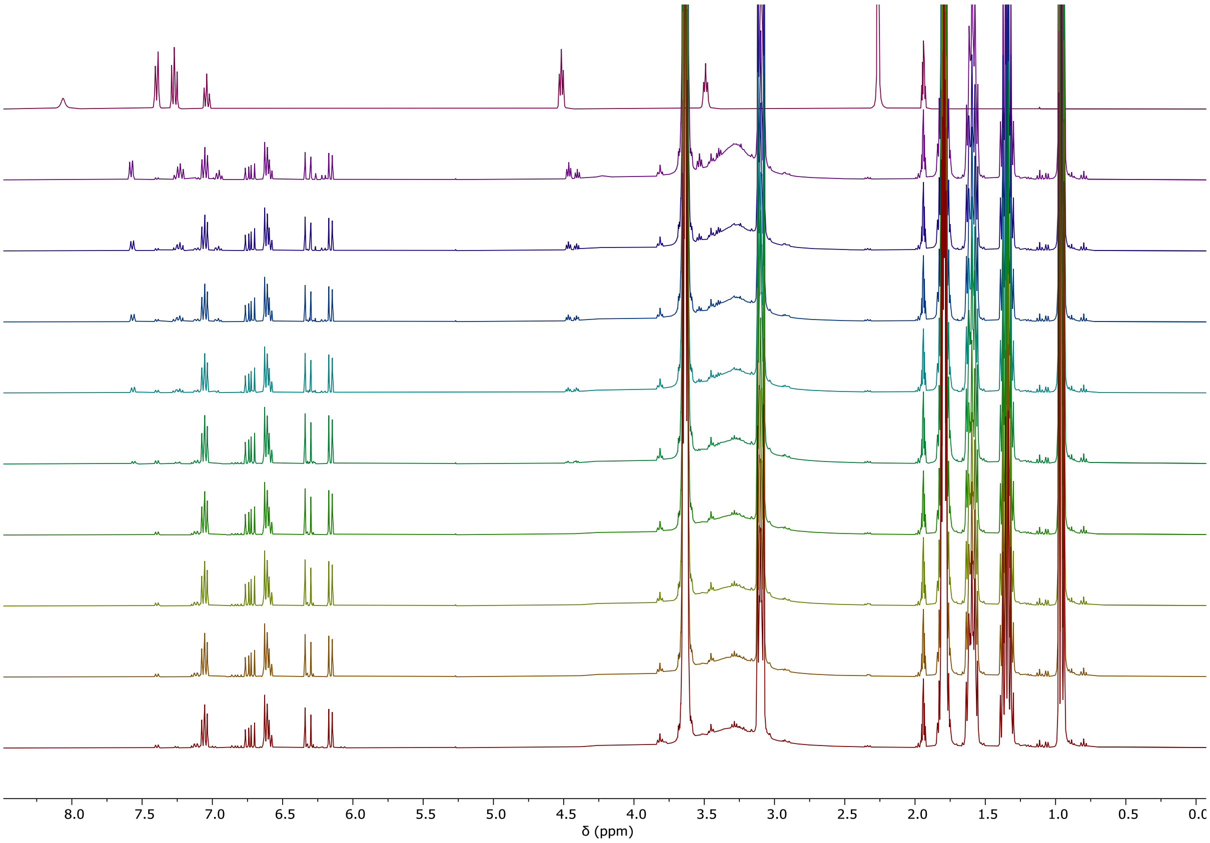

Figure S 34 ^{1}H NMR spectra recorded overtime following the addition of 5 molar equiv. of 1 M TBAF in THF to a 10 mg mL^{-1} solution of model urethane **1** (400 MHz, MeCN-d_3). Descending from T=0 minutes to T=10080 minutes (1 week).

Table S 16 Degradation kinetics of model urethanes **1** calculated from the ^{1}H NMR spectra (20 °C) obtained following the addition of 5 molar equiv. of 40 wt.% NaOD in D_2O or 1 M TBAF in THF to a 10 mg mL^{-1} solution of model urethane in MeCN-d_3. The error shown is the standard deviation between the three repeats for each sample.

Time (min)	Percentage concentration urethane H NaOD (%)	Percentage concentration aromatic H NaOD (%)	Percentage concentration urethane H TBAF (%)	Percentage concentration aromatic H TBAF (%)
0	100 ± 0.00	100 ± 0.00	100 ± 0.00	100 ± 0.00
5	3.26 ± 0.72	3.21 ± 1.21	4.09 ± 0.81	4.60 ± 0.65
15	2.16 ± 0.39	1.48 ± 0.34	2.04 ± 0.40	2.07 ± 0.31
30	1.04 ± 0.85	0.49 ± 0.40	-	-
60	-	-	-	-
180	-	-	-	-
720	-	-	-	-
1440	-	-	-	-
2880	-	-	-	-
4320	-	-	-	-
10080	-	-	-	-

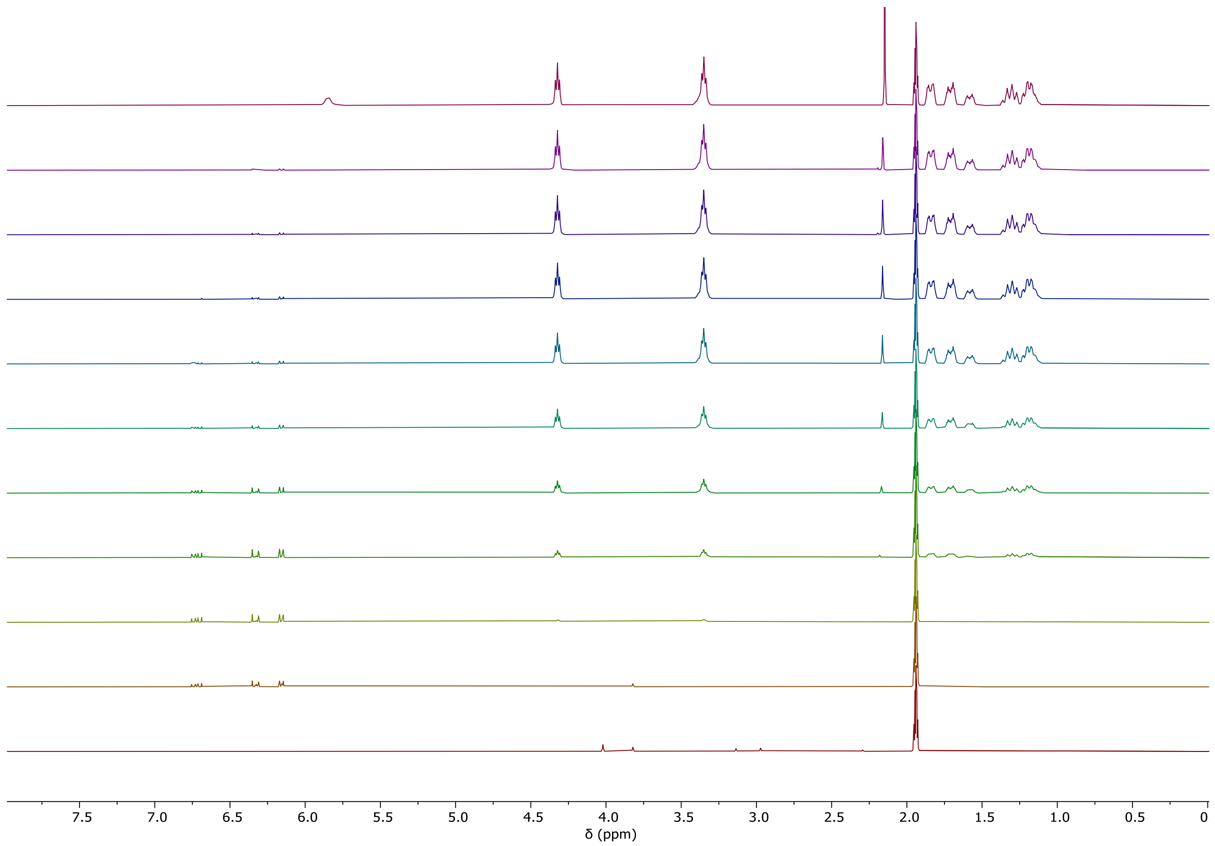

Figure S 35 ^{1}H NMR spectra recorded overtime following the addition of 5 molar equiv. of 40 wt.% NaOD in D_2O to a 10 mg mL^{-1} solution of model urethane **2** (400 MHz, MeCN-d_3). Descending from T=0 minutes to T=10080 minutes (1 week).

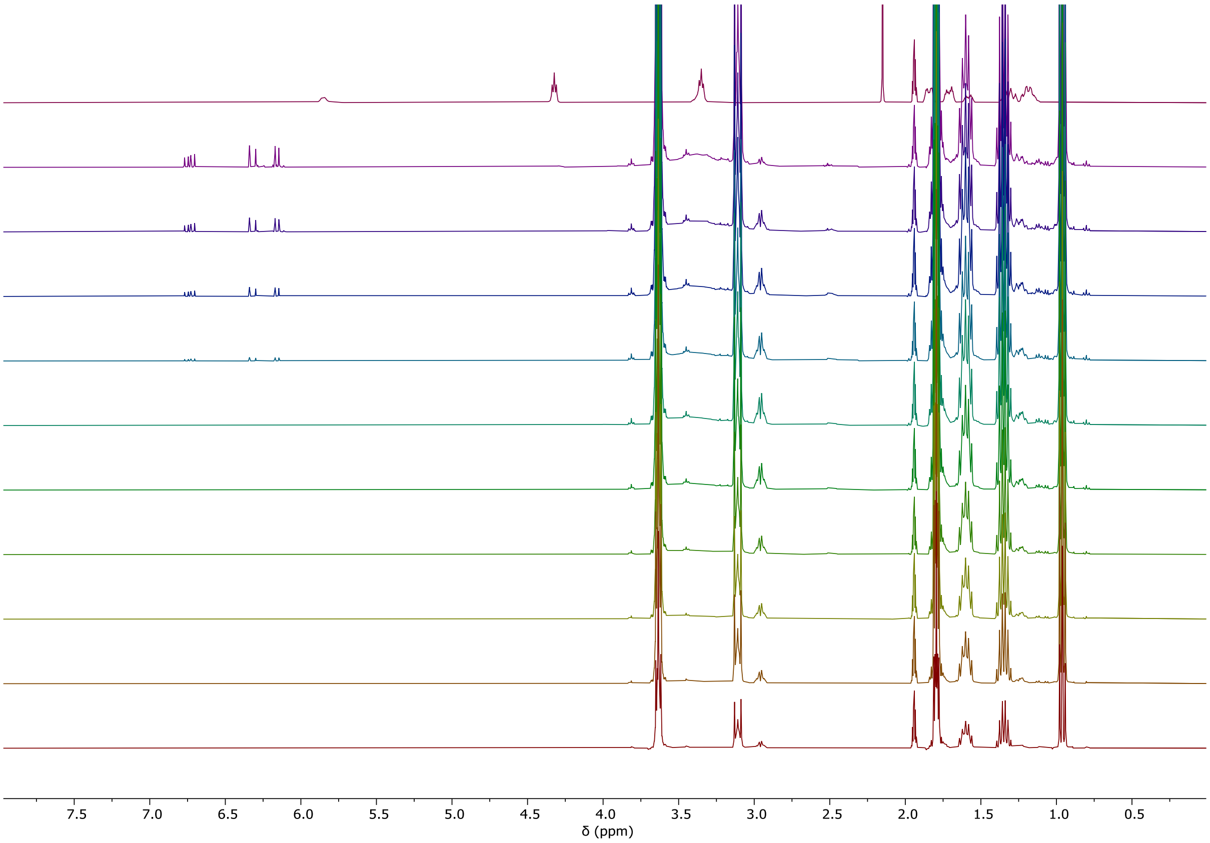

Figure S 36 ^{1}H NMR spectra recorded overtime following the addition of 5 molar equiv. of 1 M TBAF in THF to a 10 mg mL^{-1} solution of model urethane **2** (400 MHz, MeCN-d_3). Descending from T=0 minutes to T=10080 minutes (1 week).

Table S 17 Degradation kinetics of model urethanes **2** calculated from the ^{1}H NMR spectra (20 °C) obtained following the addition of 5 molar equiv. of 40 wt.% NaOD in D_2O or 1 M TBAF in THF to a 10 mg mL^{-1} solution of model urethane in MeCN-d_3. The error shown is the standard deviation between the three repeats for each sample.

Time (min)	Percentage concentration urethane H NaOD (%)	Percentage concentration methylene H NaOD (%)	Percentage concentration urethane H TBAF (%)	Percentage concentration methylene H TBAF (%)
0	100 ± 0.00	100 ± 0.00	100 ± 0.00	100 ± 0.00
5	19.29 ± 9.62	97.25 ± 1.15	5.55 ± 0.90	4.27 ± 2.05
15	13.62 ± 5.10	91.16 ± 1.79	1.64 ± 0.28	1.82 ± 0.44
30	12.18 ± 4.69	85.08 ± 2.84	0.52 ± 0.42	0.61 ± 0.50
60	10.02 ± 4.03	78.98 ± 3.73	-	-
180	6.45 ± 2.63	61.31 ± 4.66	-	-
720	2.14 ± 1.00	37.23 ± 5.34	-	-
1440	0.71 ± 0.58	27.78 ± 5.66	-	-
2880	-	19.55 ± 5.59	-	-
4320	-	15.59 ± 5.41	-	-
10080	-	7.95 ± 4.16	-	-

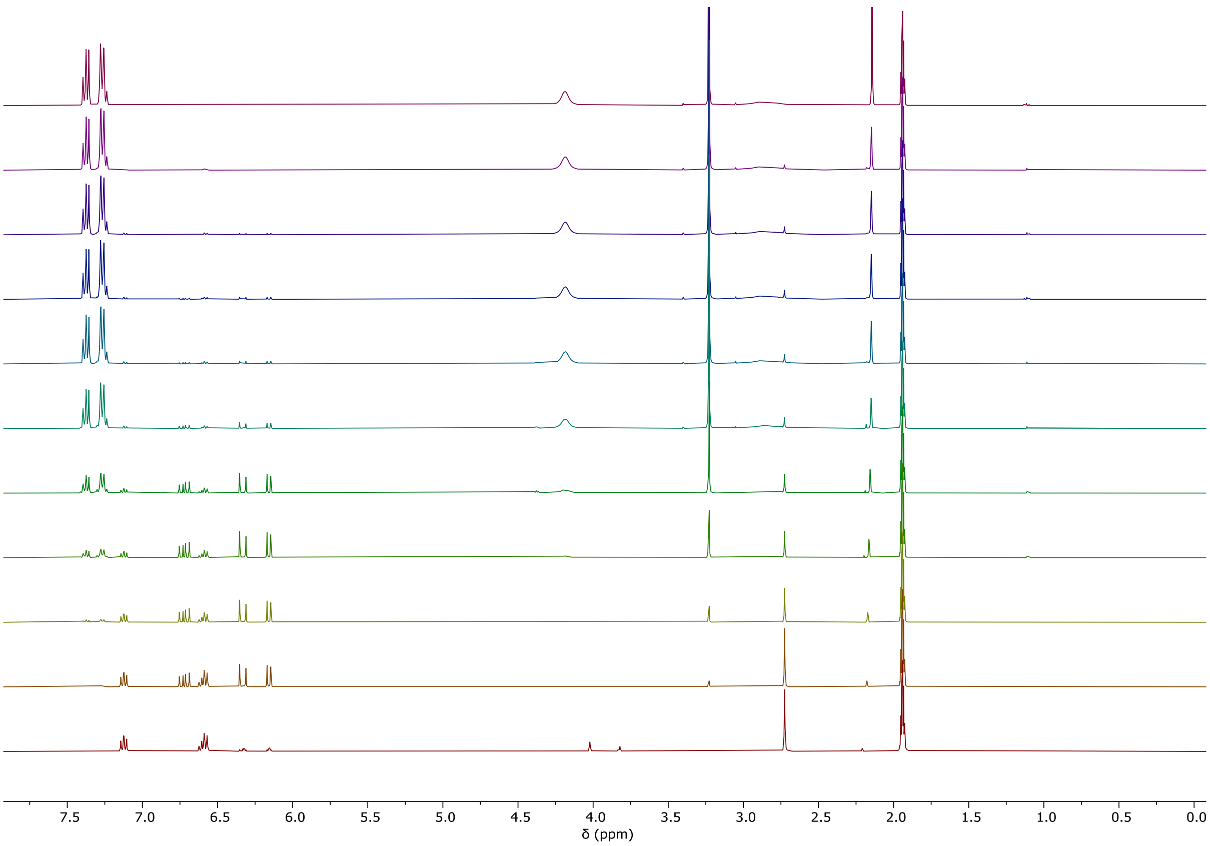

Figure S 37 ^{1}H NMR spectra recorded overtime following the addition of 5 molar equiv. of 40 wt.% NaOD in D_2O to a 10 mg mL^{-1} solution of model urethane **3** (400 MHz, MeCN-d_3). Descending from T=0 minutes to T=10080 minutes (1 week).

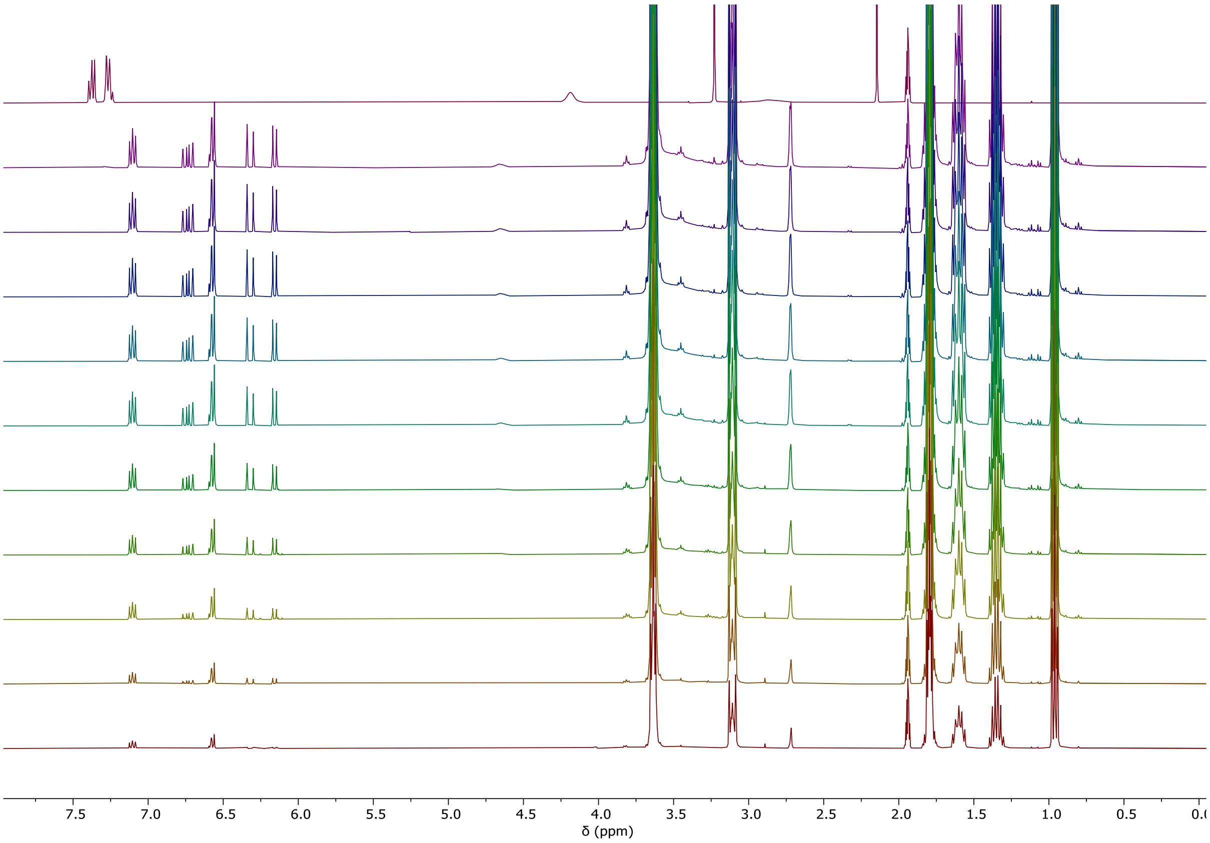

Figure S 38 ^{1}H NMR spectra recorded overtime following the addition of 5 molar equiv. of 1 M TBAF in THF to a 10 mg mL^{-1} solution of model urethane **3** (400 MHz, MeCN-d_3). Descending from T=0 minutes to T=10080 minutes (1 week).

Table S 18 Degradation kinetics of model urethanes **3** calculated from the ^{1}H NMR spectra (20 °C) obtained following the addition of 5 molar equiv. of 40 wt.% NaOD in D_2O or 1 M TBAF in THF to a 10 mg mL^{-1} solution of model urethane in MeCN-d_3. The error shown is the standard deviation between the three repeats for each sample.

Time (min)	Percentage concentration urethane CH_3 NaOD (%)	Percentage concentration aromatic H NaOD (%)	Percentage concentration urethane CH_3 TBAF (%)	Percentage concentration aromatic H TBAF (%)
0	100 ± 0.00	100 ± 0.00	100 ± 0.00	100 ± 0.00
5	94.26 ± 0.80	94.56 ± 1.12	4.55 ± 0.66	4.50 ± 0.74
15	90.89 ± 1.54	90.32 ± 1.47	1.65 ± 0.60	2.10 ± 0.98
30	86.93 ± 1.67	86.99 ± 1.45	0.62 ± 0.50	1.35 ± 0.56
60	81.97 ± 2.08	81.84 ± 2.20	-	-
180	65.93 ± 3.54	65.50 ± 3.97	-	-
720	21.96 ± 2.24	24.28 ± 3.21	-	-
1440	7.52 ± 1.28	9.11 ± 1.53	-	-
2880	2.57 ± 0.42	3.34 ± 0.51	-	-
4320	2.59 ± 1.40	1.21 ± 0.25	-	-
10080	-	-	-	-

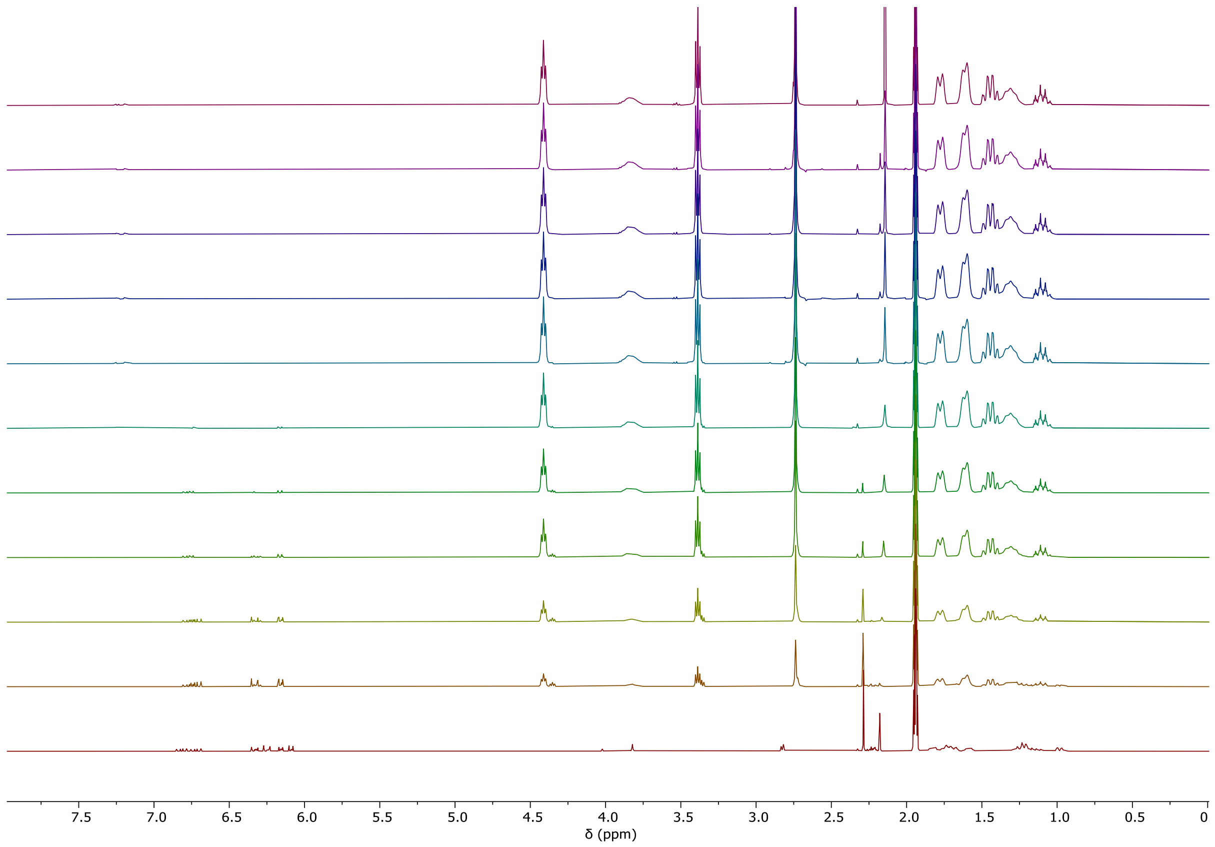

Figure S 39 ^{1}H NMR spectra recorded overtime following the addition of 5 molar equiv. of 40 wt.% NaOD in D_2O to a 10 mg mL^{-1} solution of model urethane **4** (400 MHz, MeCN-d_3). Descending from T=0 minutes to T=10080 minutes (1 week).

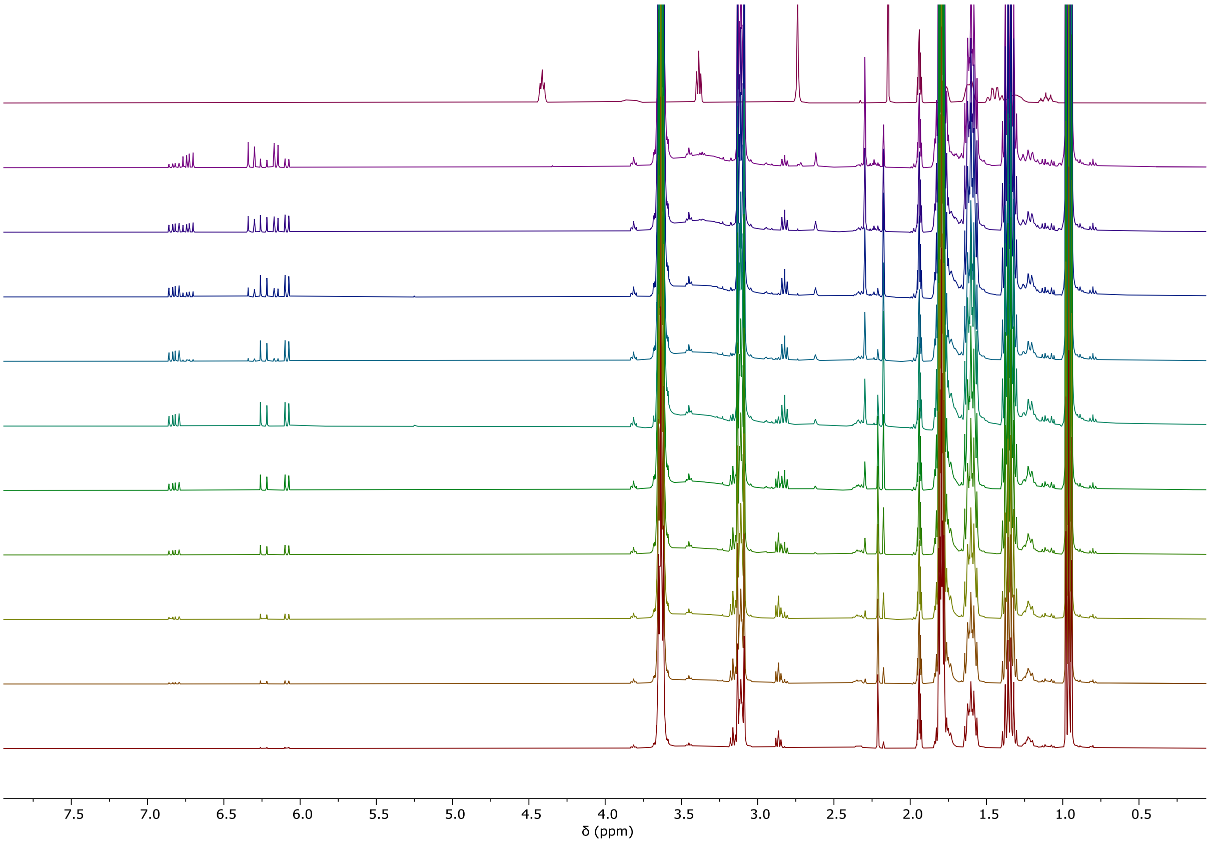

Figure S 40 ^{1}H NMR spectra recorded overtime following the addition of 5 molar equiv. of 1 M TBAF in THF to a 10 mg mL^{-1} solution of model urethane **4** (400 MHz, MeCN-d_3). Descending from T=0 minutes to T=10080 minutes (1 week).

Table S 19 Degradation kinetics of model urethanes **4** calculated from the ^{1}H NMR spectra (20 °C) obtained following the addition of 5 molar equiv. of 40 wt.% NaOD in D_2O or 1 M TBAF in THF to a 10 mg mL^{-1} solution of model urethane in MeCN-d_3. The error shown is the standard deviation between the three repeats for each sample.

Time (min)	Percentage concentration urethane CH_3 NaOD (%)	Percentage concentration methylene H NaOD (%)	Percentage concentration urethane CH_3 TBAF (%)	Percentage concentration methylene H TBAF (%)
0	100 ± 0.00	100 ± 0.00	100 ± 0.00	100 ± 0.00
5	97.93 ± 0.18	96.96 ± 0.66	7.56 ± 0.30	5.22 ± 0.68
15	94.61 ± 0.49	94.50 ± 1.34	3.78 ± 0.30	2.15 ± 0.51
30	92.13 ± 0.46	92.37 ± 1.97	2.31 ± 0.17	1.22 ± 0.24
60	88.60 ± 0.82	88.94 ± 2.71	1.05 ± 0.46	0.61 ± 0.25
180	78.65 ± 1.18	79.06 ± 3.45	-	-
720	58.73 ± 3.32	61.03 ± 7.69	-	-
1440	49.82 ± 3.74	56.59 ± 11.45	-	-
2880	30.59 ± 3.00	34.88 ± 9.48	-	-
4320	17.34 ± 2.36	22.19 ± 8.22	-	-
10080	0.20 ± 0.17	0.18 ± 0.15	-	-

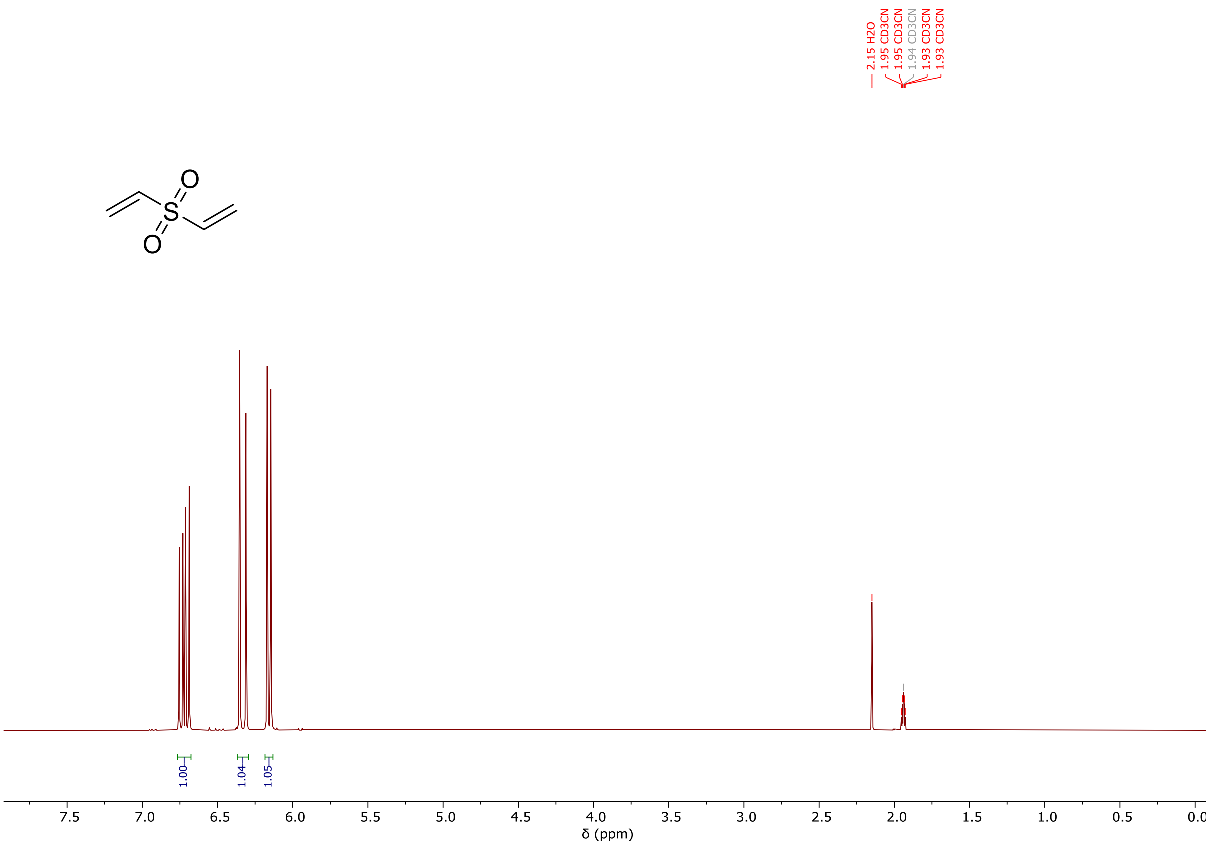

Figure S 41 ^{1}H NMR spectrum of divinyl sulfone (400 MHz, MeCN-d_3, 298 K).

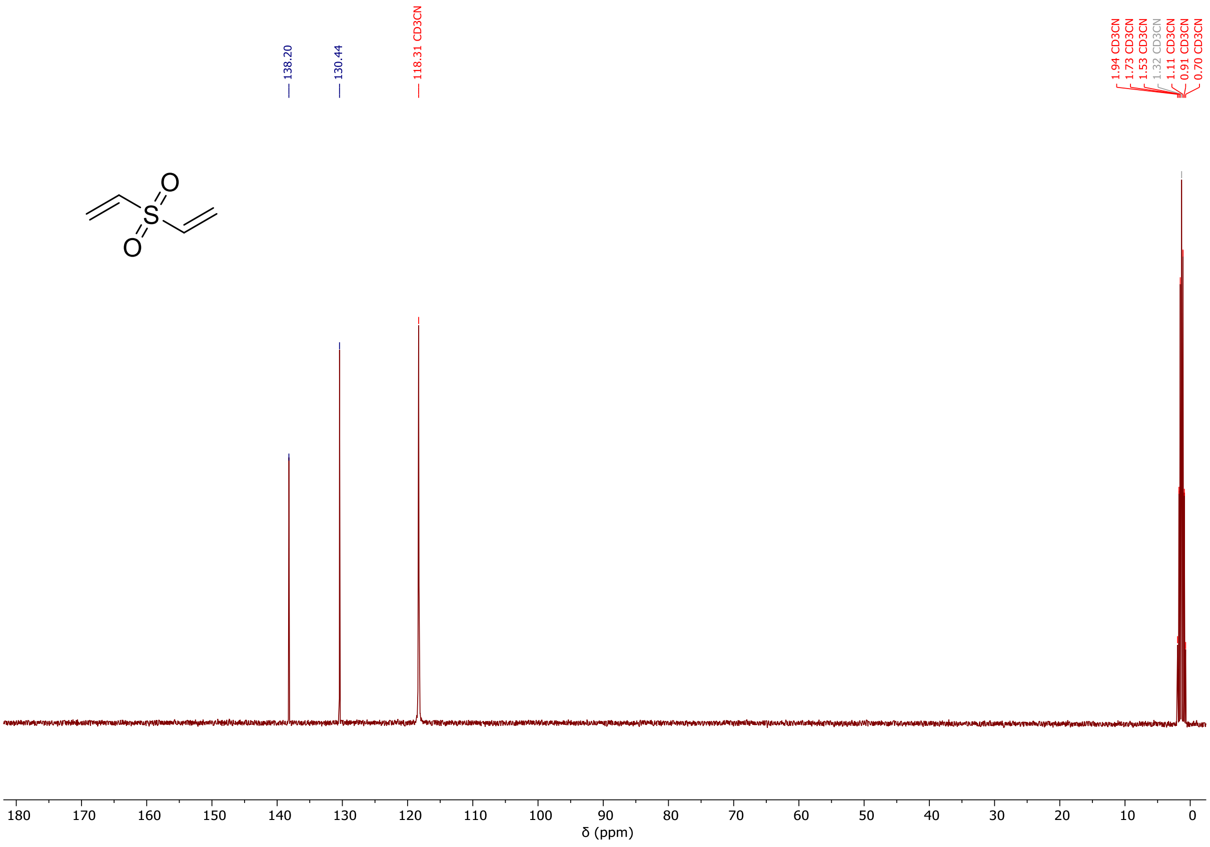

Figure S 42 ^{13}C {H} NMR spectrum of divinyl sulfone (100 MHz, MeCN-d_3, 298 K).

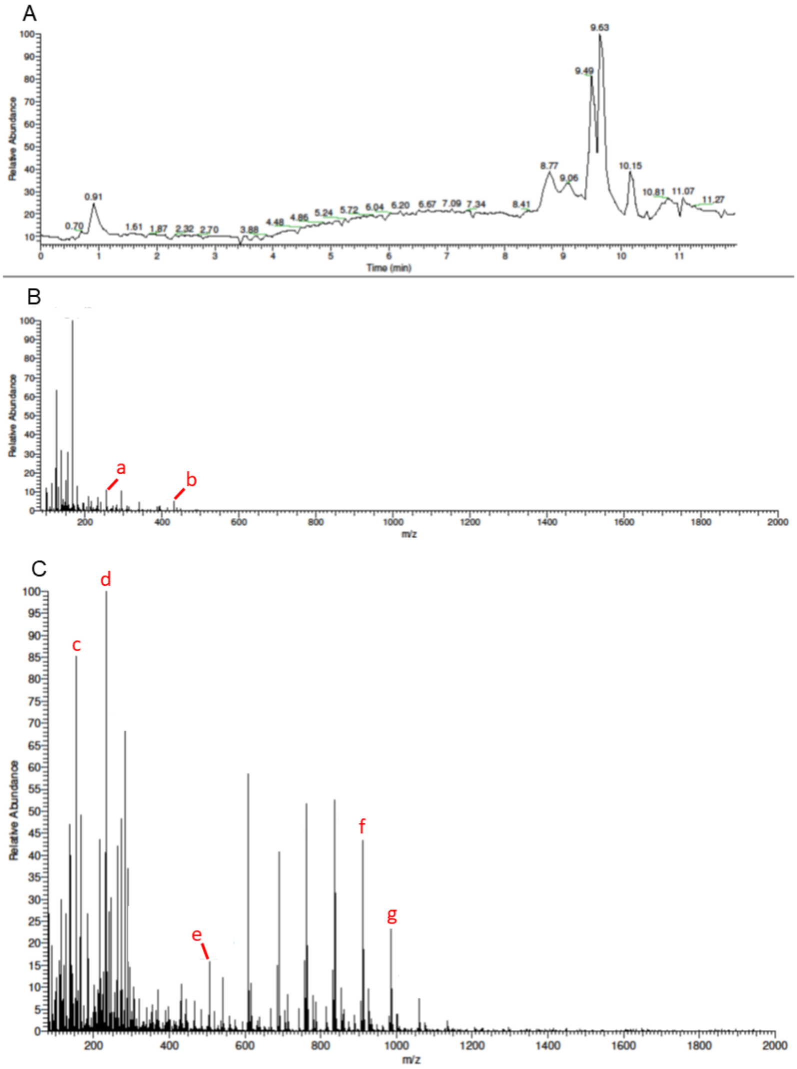

Figure S 43 LC-MS of the solution state degradation of **1** (10 mg mL^{-1} in MeCN-d_3) with 40 wt.% $NaOD_{(aq)}$ (5 equiv.). **A** LC eluogram, **B** LC-MS at RT=0.91 min, **C** LC-MS at RT=0.67 min.

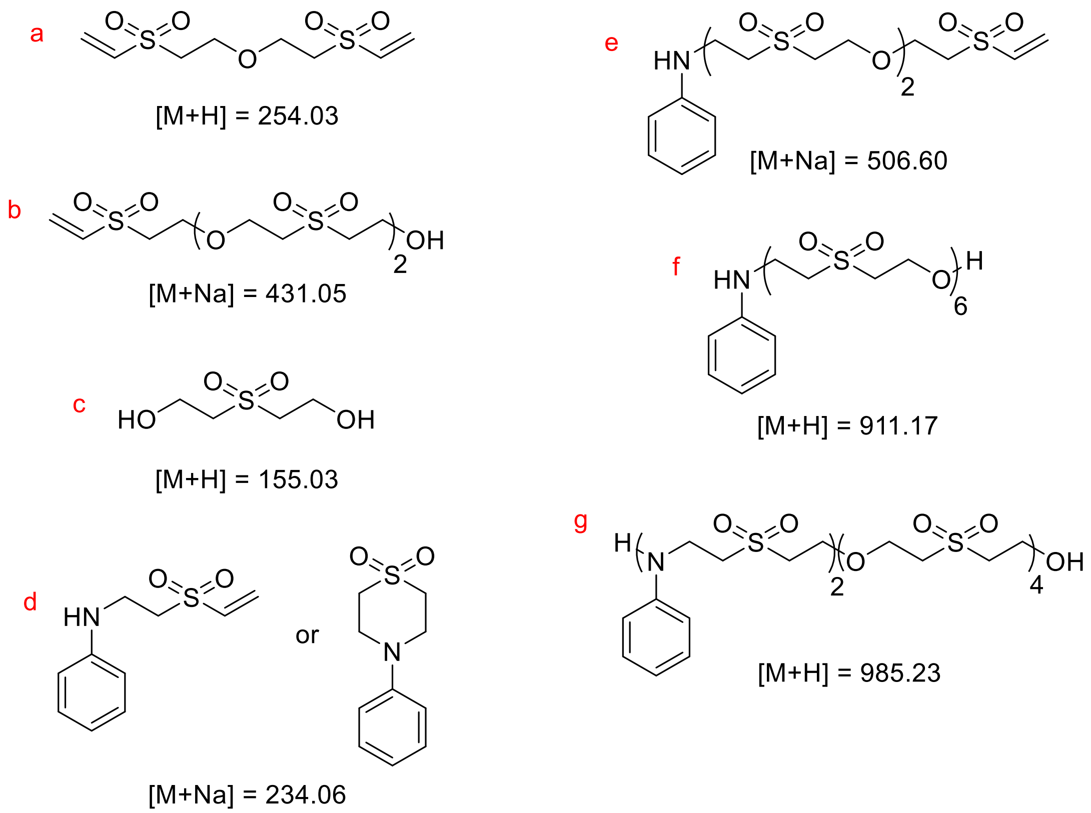

Figure S 44 Structures of secondary degradation products from the NaOD degradation of **1** observed in LC-MS.

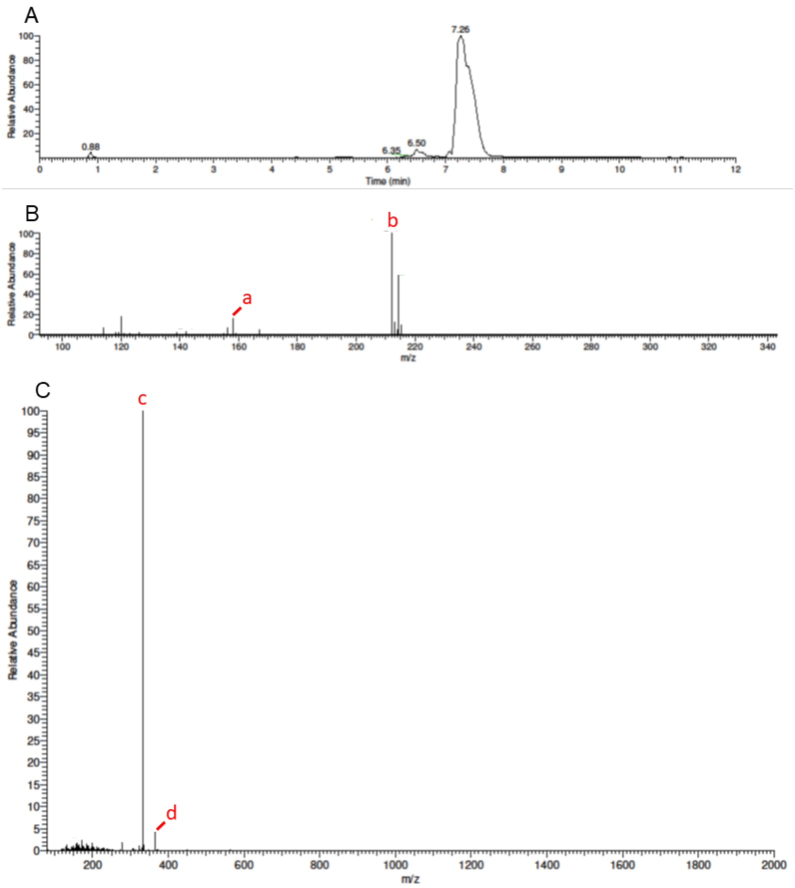

Figure S 45 LC-MS of the solution state degradation of **1** (10 mg mL^{-1} in MeCN-d_3) with 1 M TBAF in THF (5 equiv.). **A** LC eluogram, **B** LC-MS at RT=6.55 min, **C** LC-MS at RT=0.69 min.

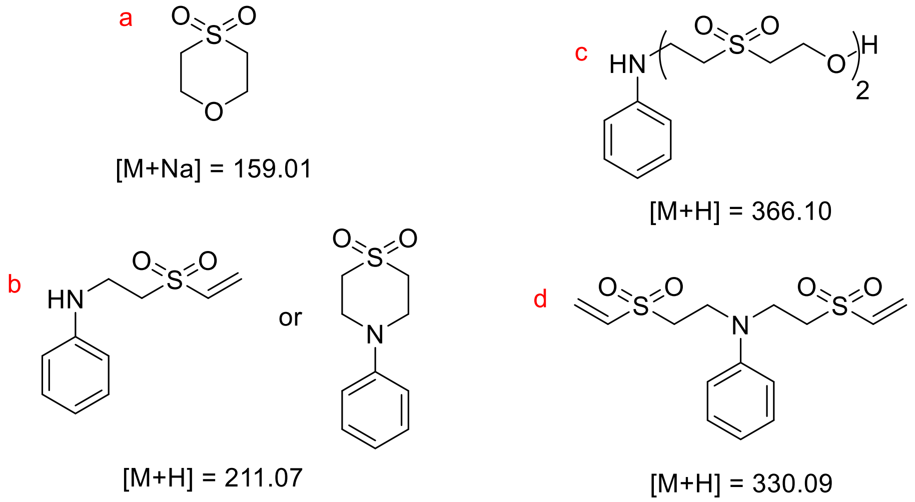

Figure S 46 Structures of secondary degradation products from the TBAF degradation of **1** observed in LC-MS.

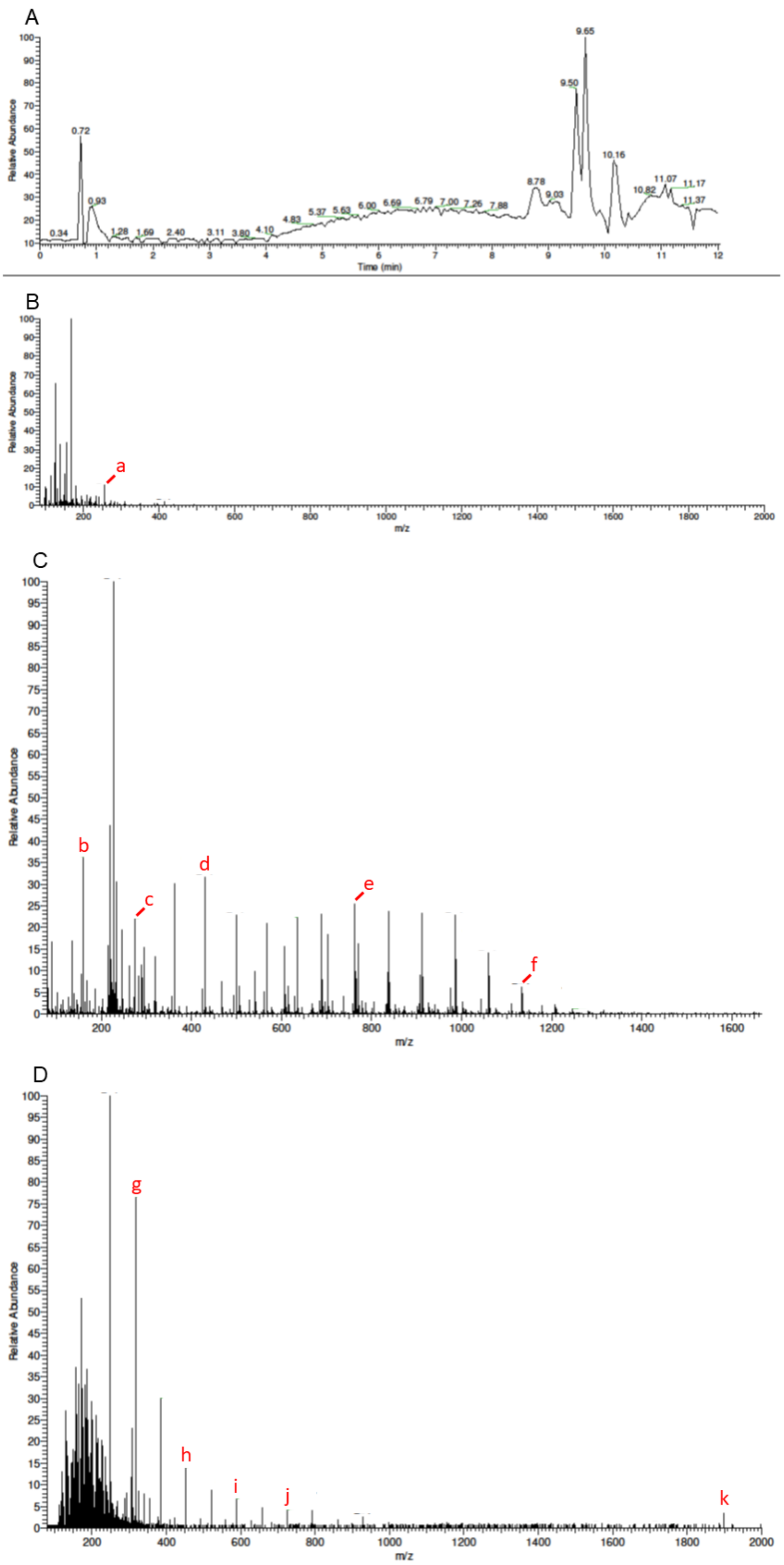

Figure S 47 LC-MS of the solution state degradation of **2** (10 mg mL^{-1} in MeCN-d_3) with 40 wt.% $NaOD_{(aq)}$ (5 equiv.). **A** LC eluogram, **B** LC-MS at RT=0.93 min, **C** LC-MS at RT=0.68 min, **D** LC-MS at RT=0.66 min.

a

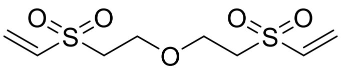

[M+H] = 254.03

b

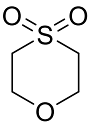

[M+H] = 159.01

c

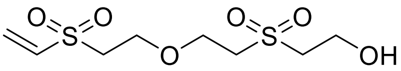

[M+H] = 273.04

d

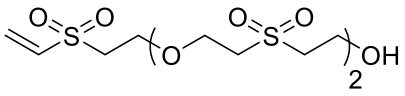

[M+H] = 431.05

e

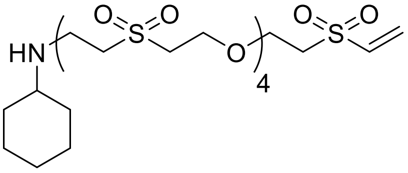

[M+H] = 762.20

f

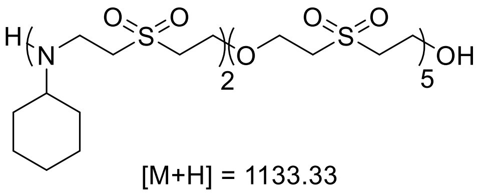

[M+H] = 1133.33

g

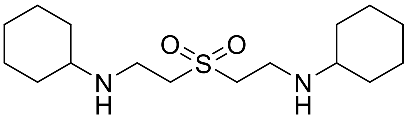

[M+H] = 317.22

h

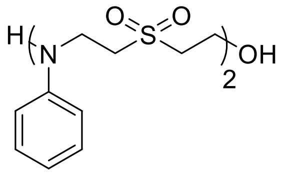

[M+H] = 453.24

i

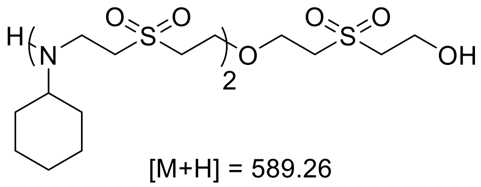

[M+H] = 589.26

j

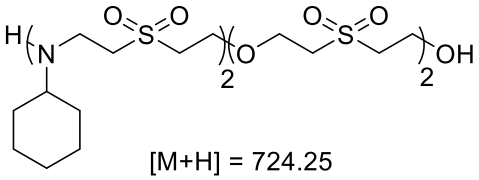

[M+H] = 724.25

k

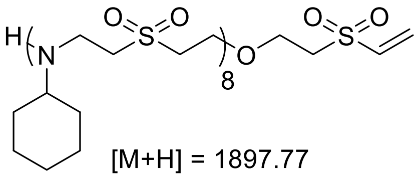

[M+H] = 1897.77

Figure S 48 Structures of secondary degradation products from the NaOD degradation of **2** observed in LC-MS.

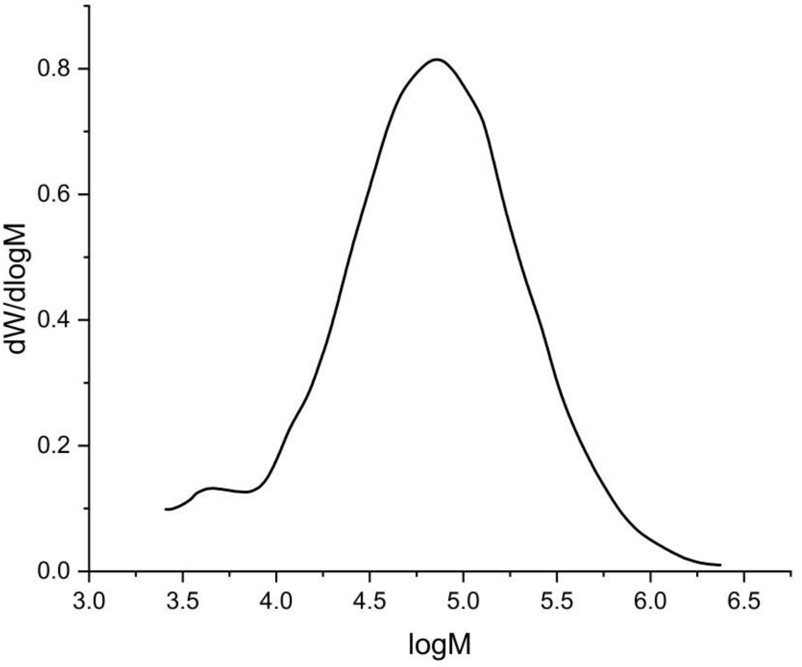

Figure S 49 GPC eluogram of **CEPU1** in THF.

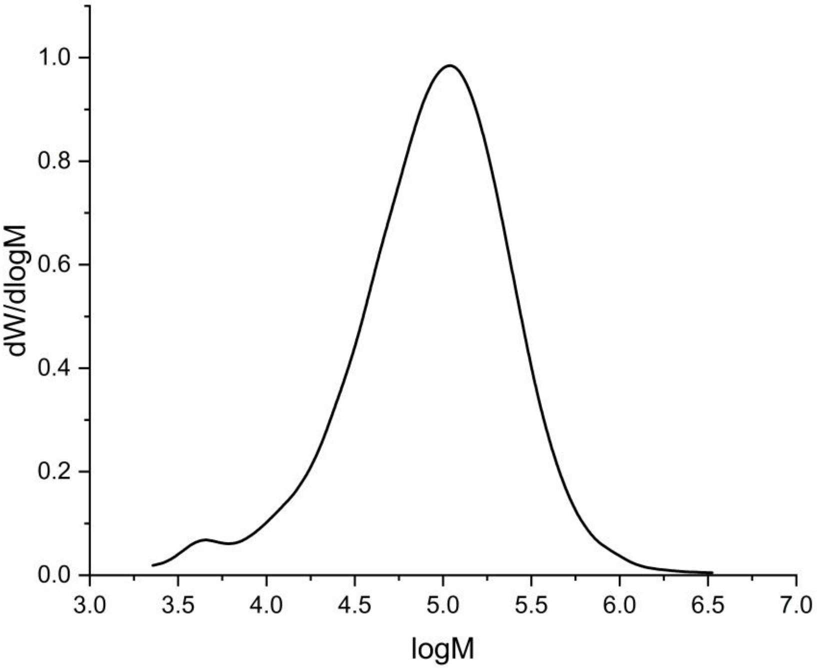

Figure S 50 GPC eluogram of **CEPU2** in THF.

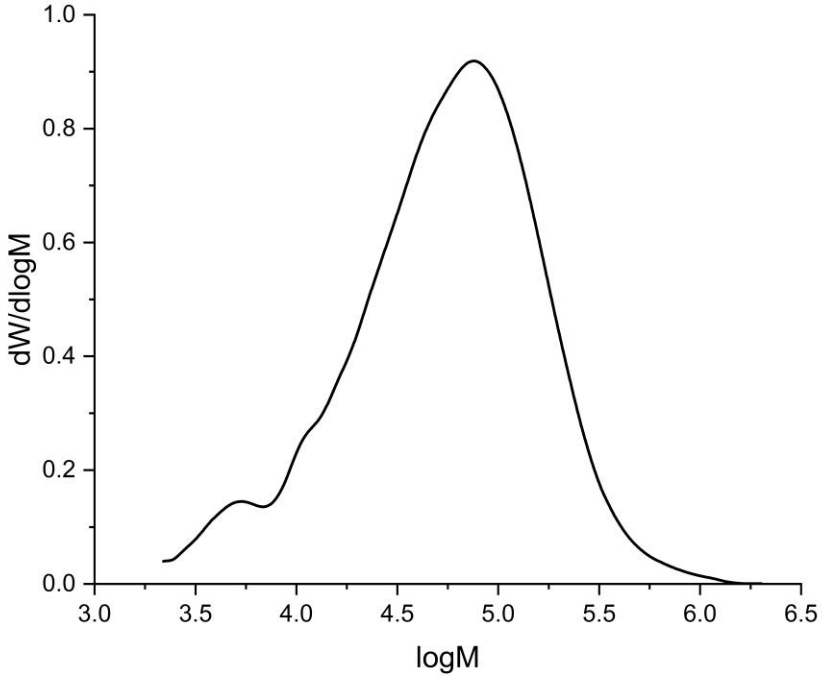

Figure S 51 GPC eluogram of **CEPU3** in THF.

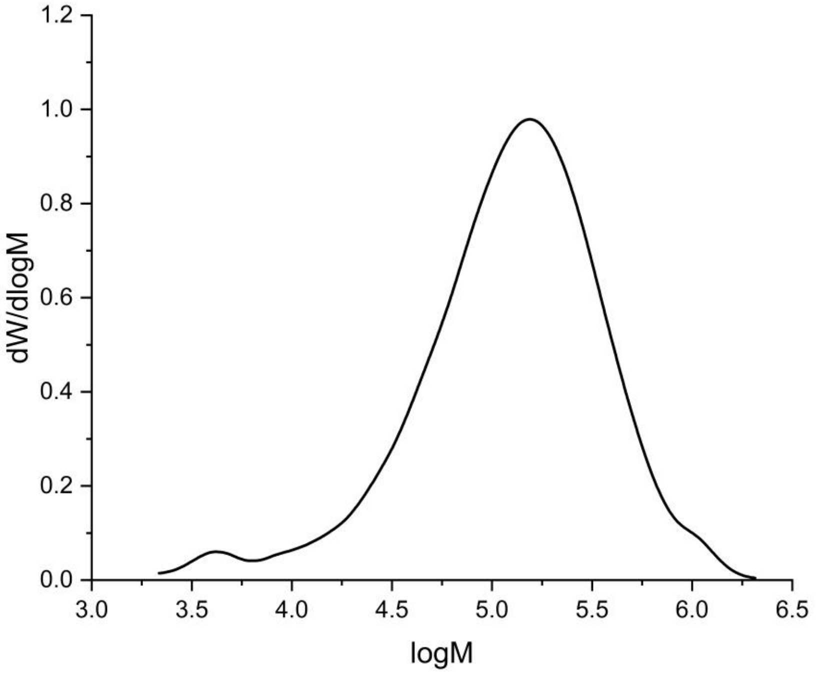

Figure S 52 GPC eluogram of **CEPU4** in THF.

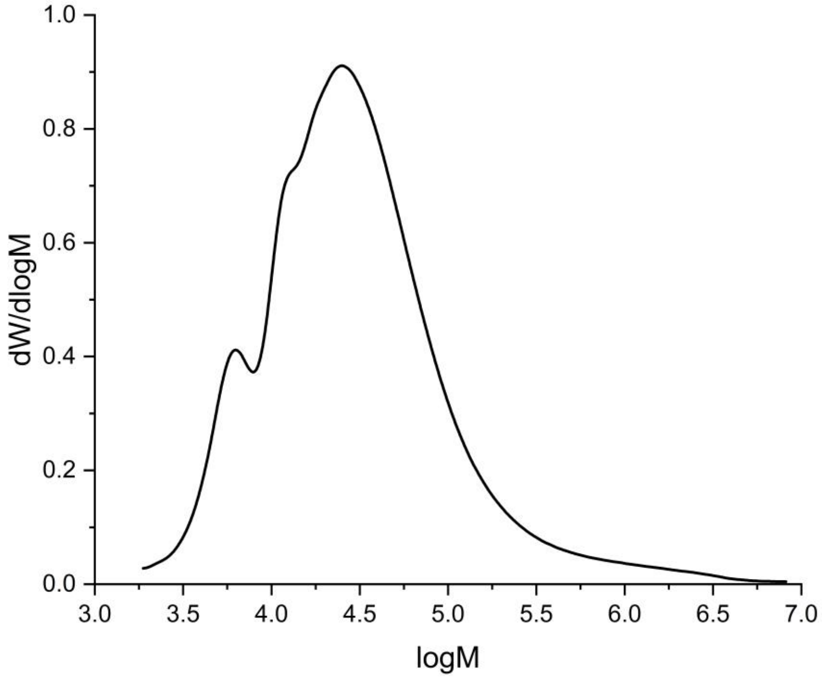

Figure S 53 GPC eluogram of **CEPU5** in THF.

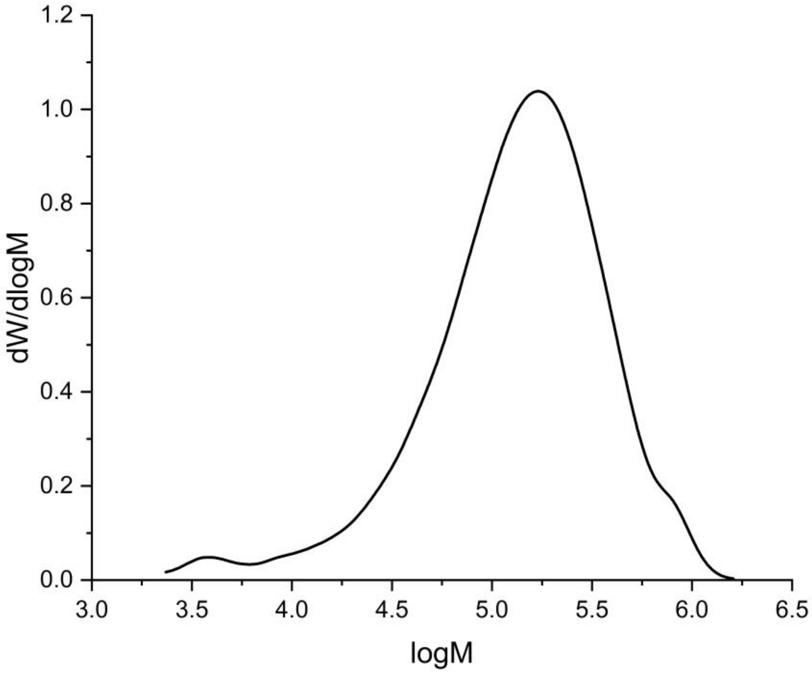

Figure S 54 GPC eluogram of **CEPU6** in THF.

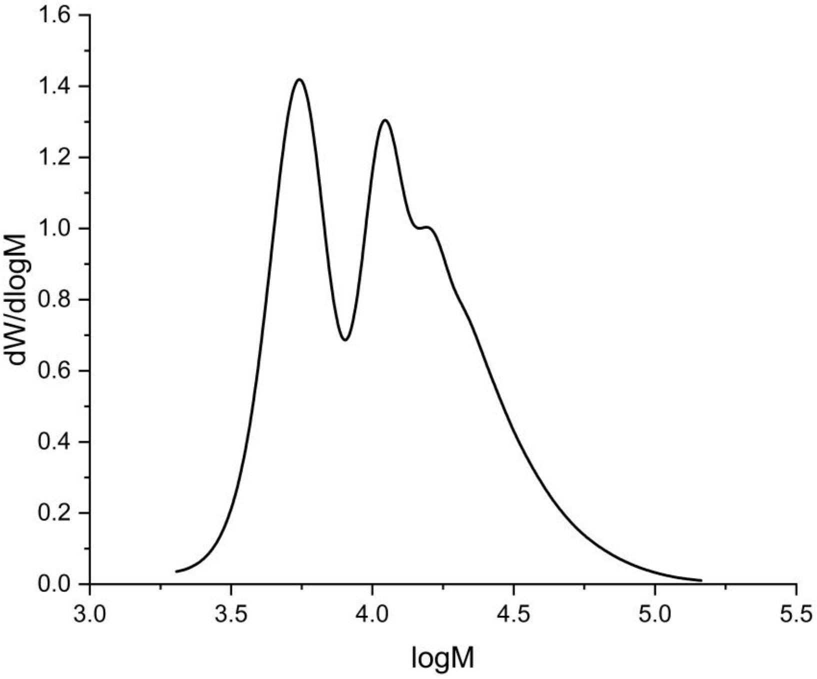

Figure S 55 GPC eluogram of **MeO-PU1** in THF.

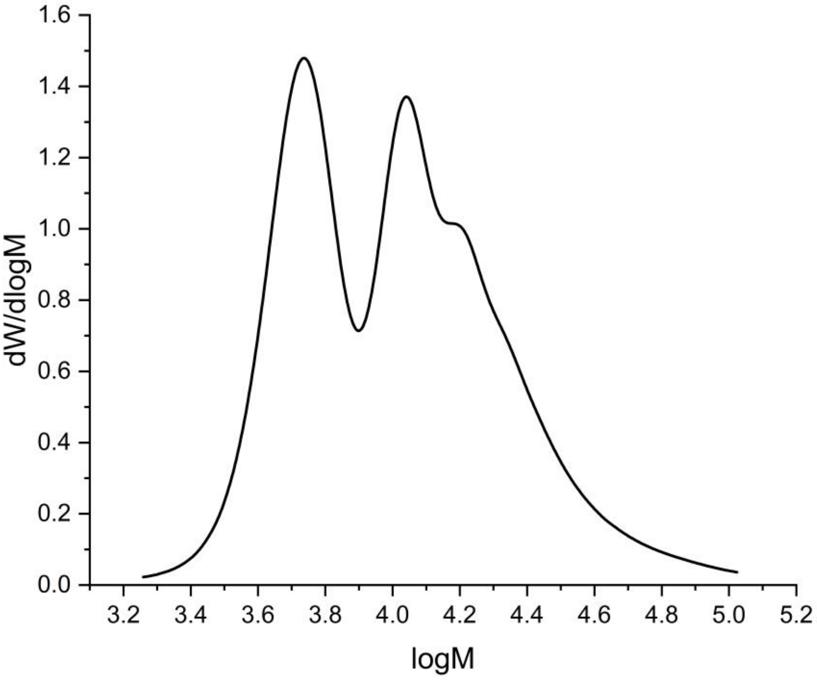

Figure S 56 GPC eluogram of **MeO-PU2** in THF.

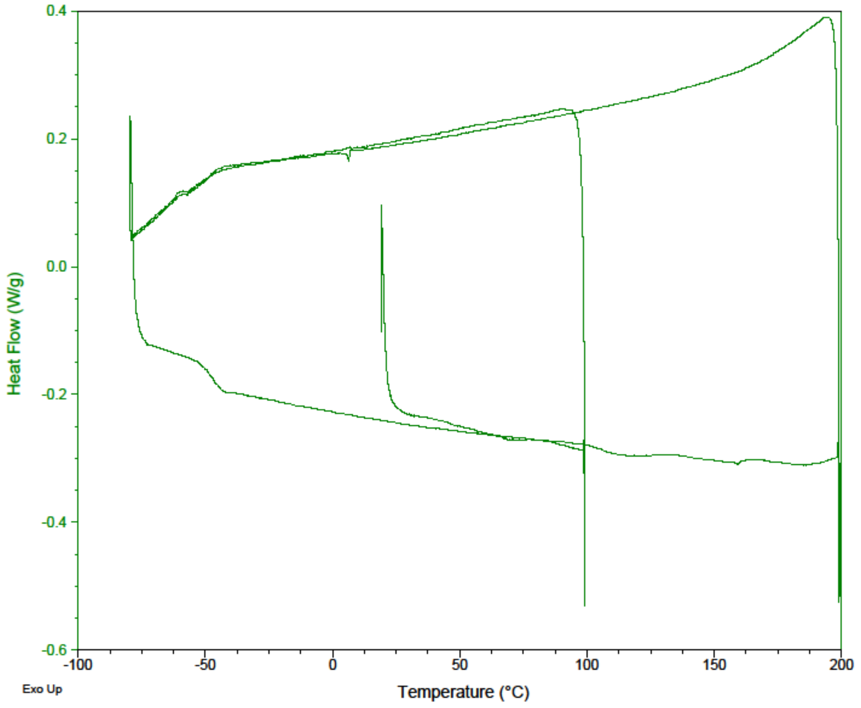

Figure S 57 DSC thermogram of **CEPU1** showing the 1st and 2nd heating and cooling cycles at 10 °C min^{-1}.

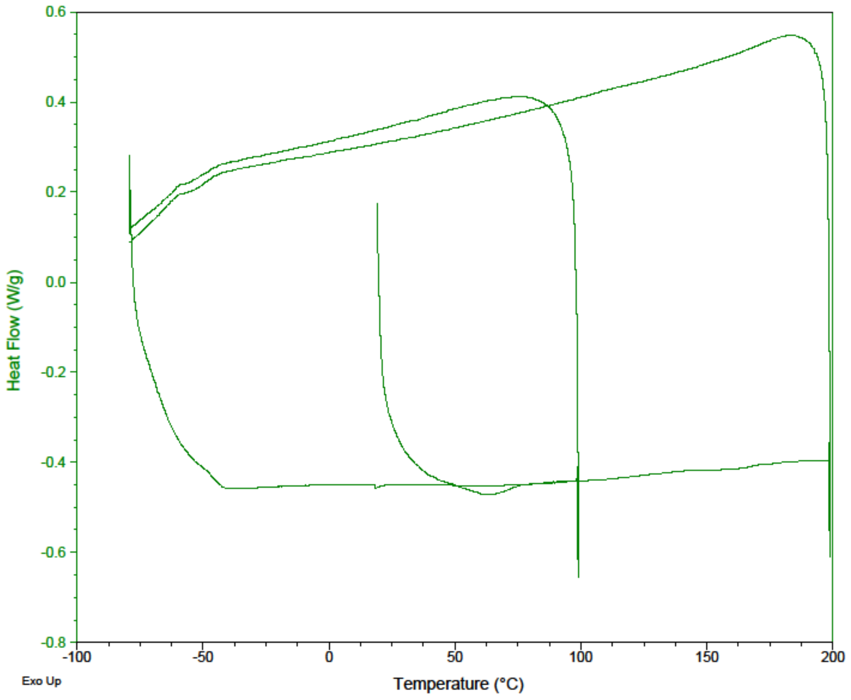

Figure S 58 DSC thermogram of **CEPU2** showing the 1st and 2nd heating and cooling cycles at 10 °C min^{-1}.

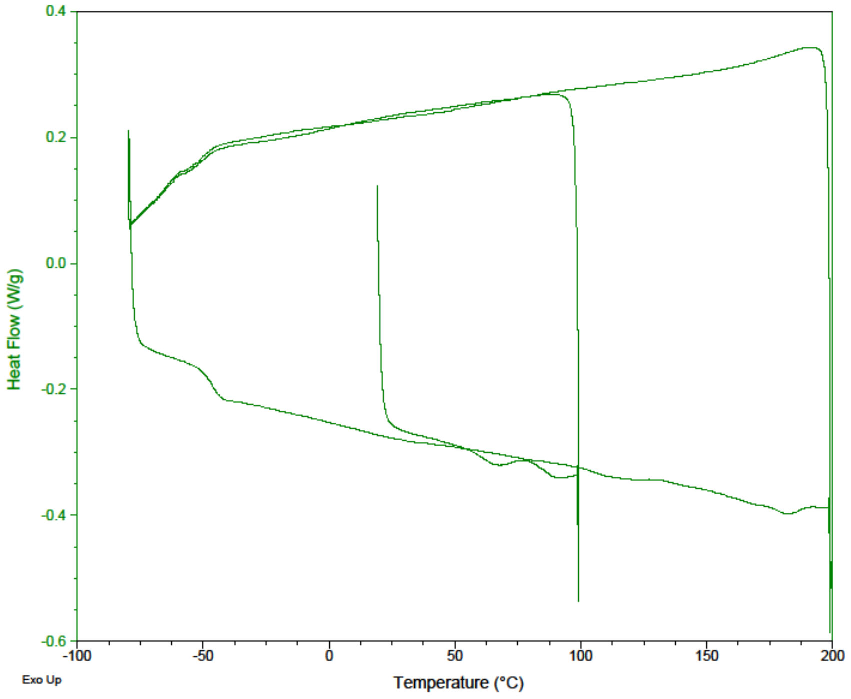

Figure S 59 DSC thermogram of **CEPU3** showing the 1st and 2nd heating and cooling cycles at 10 °C min^{-1}.

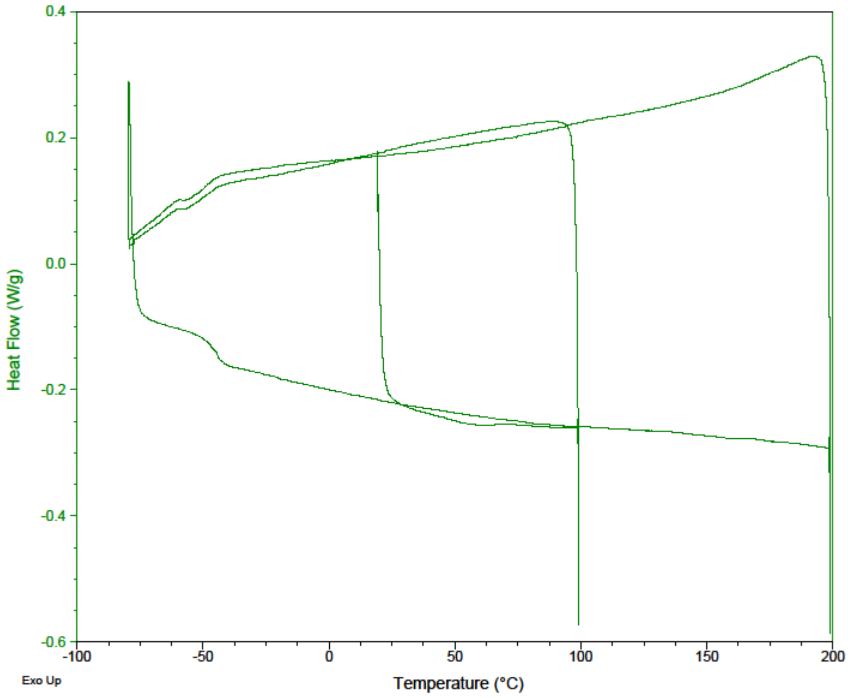

Figure S 60 DSC thermogram of **CEPU4** showing the 1st and 2nd heating and cooling cycles at 10 °C min^{-1}.

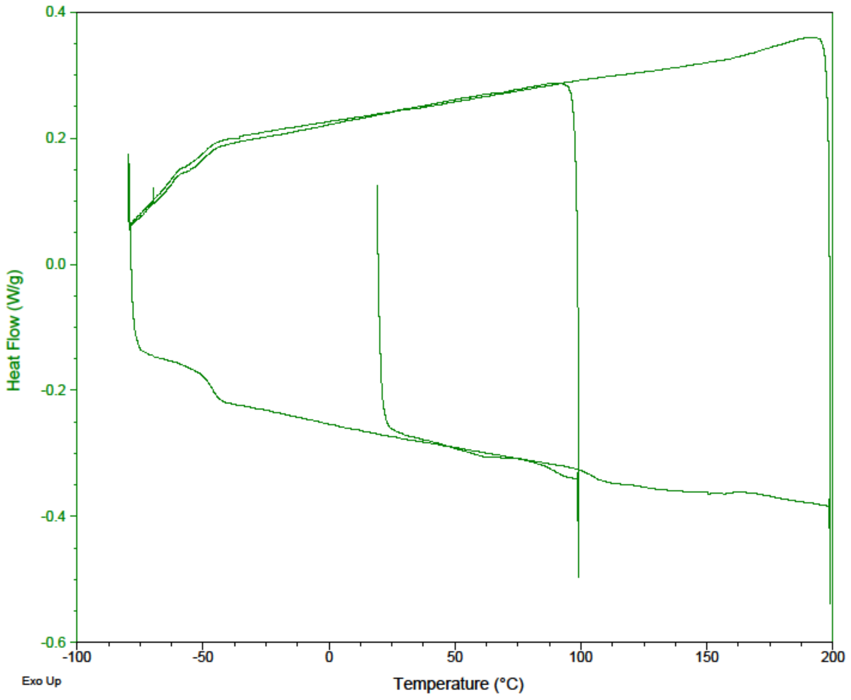

Figure S 61 DSC thermogram of **CEPU5** showing the 1st and 2nd heating and cooling cycles at 10 °C min^{-1}.

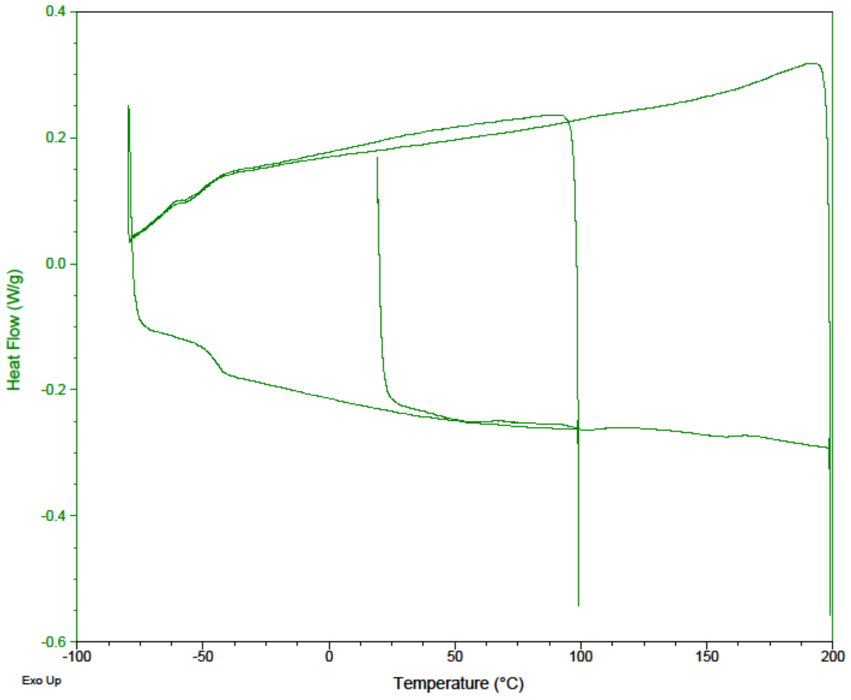

Figure S 62 DSC thermogram of **CEPU6** showing the 1st and 2nd heating and cooling cycles at 10 °C min^{-1}.

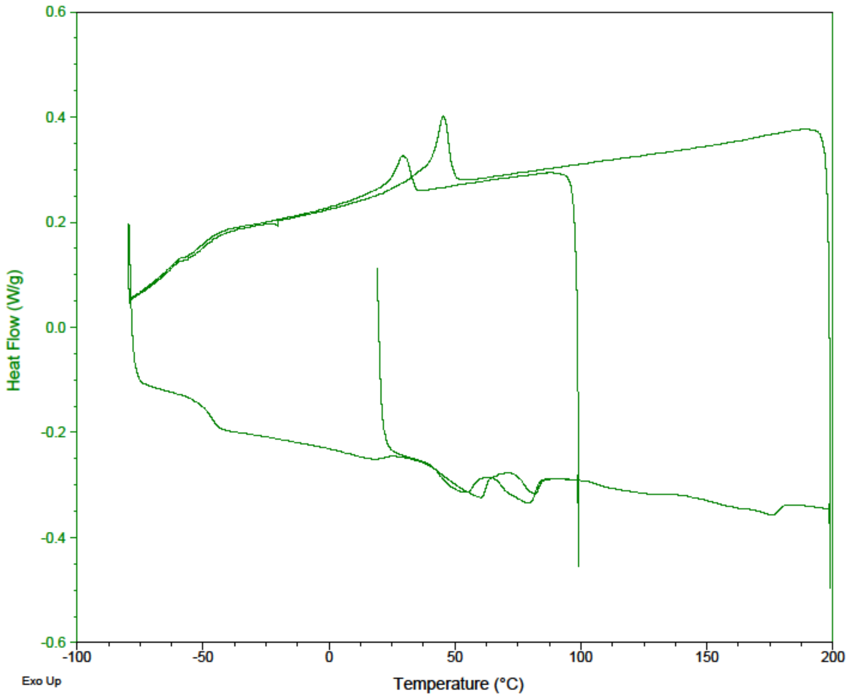

Figure S 63 DSC thermogram of **MeO-PU1** showing the 1st and 2nd heating and cooling cycles at 10 °C min^{-1}.

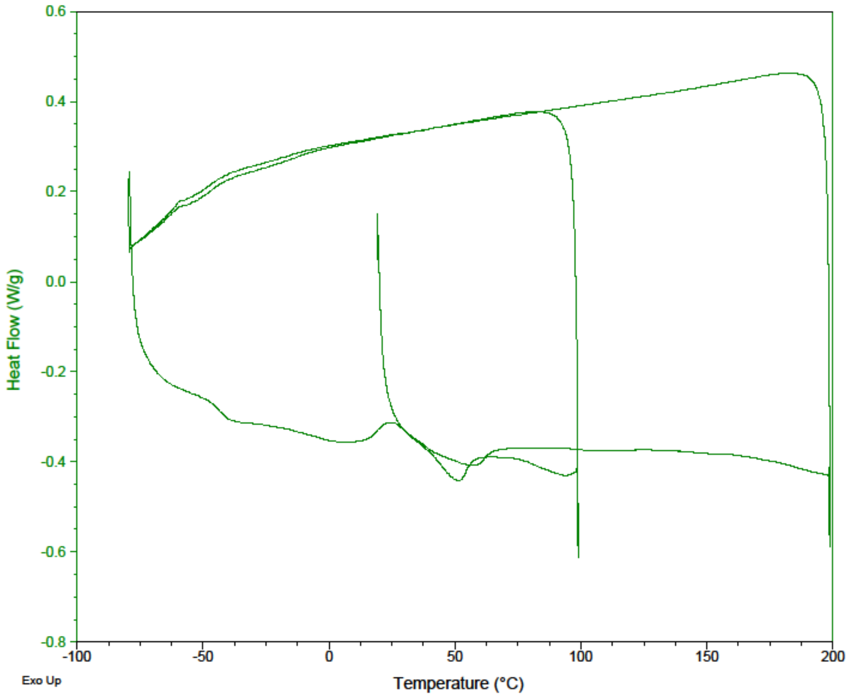

Figure S 64 DSC thermogram of **MeO-PU2** showing the 1st and 2nd heating and cooling cycles at 10 °C min^{-1}.

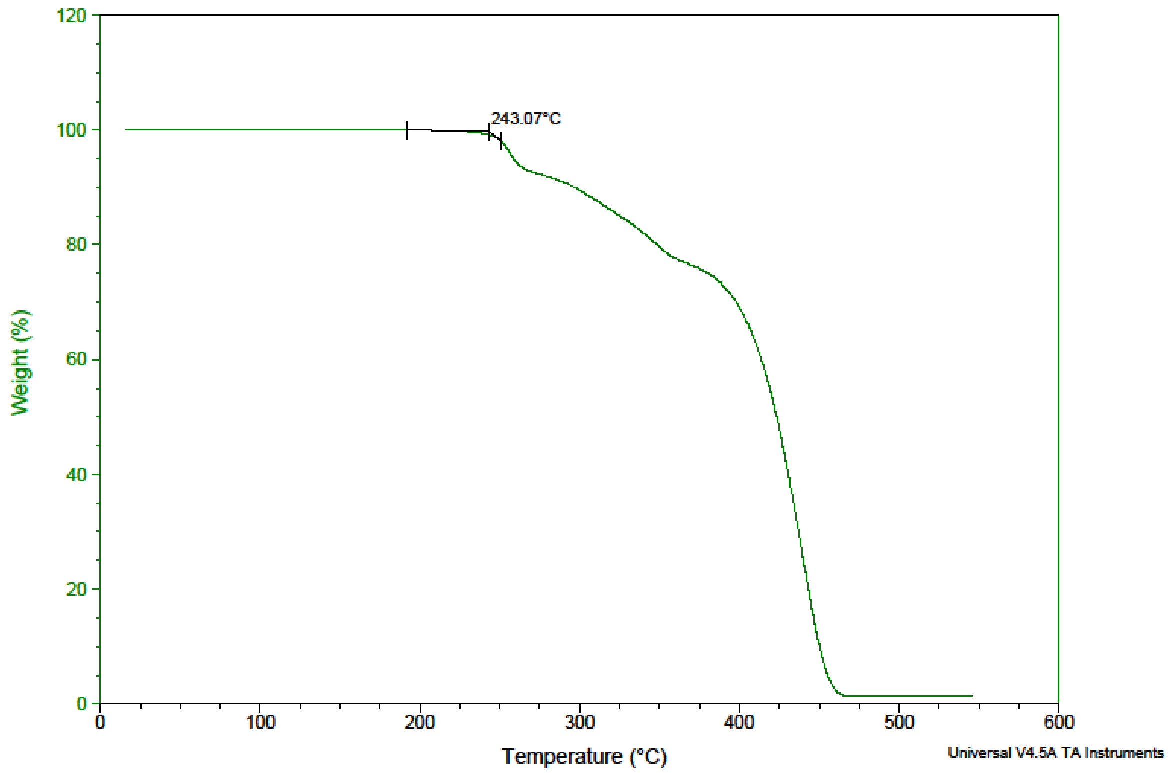

Figure S 65 TGA thermogram of **CEPU1** at 10 °C min^{-1} under nitrogen.

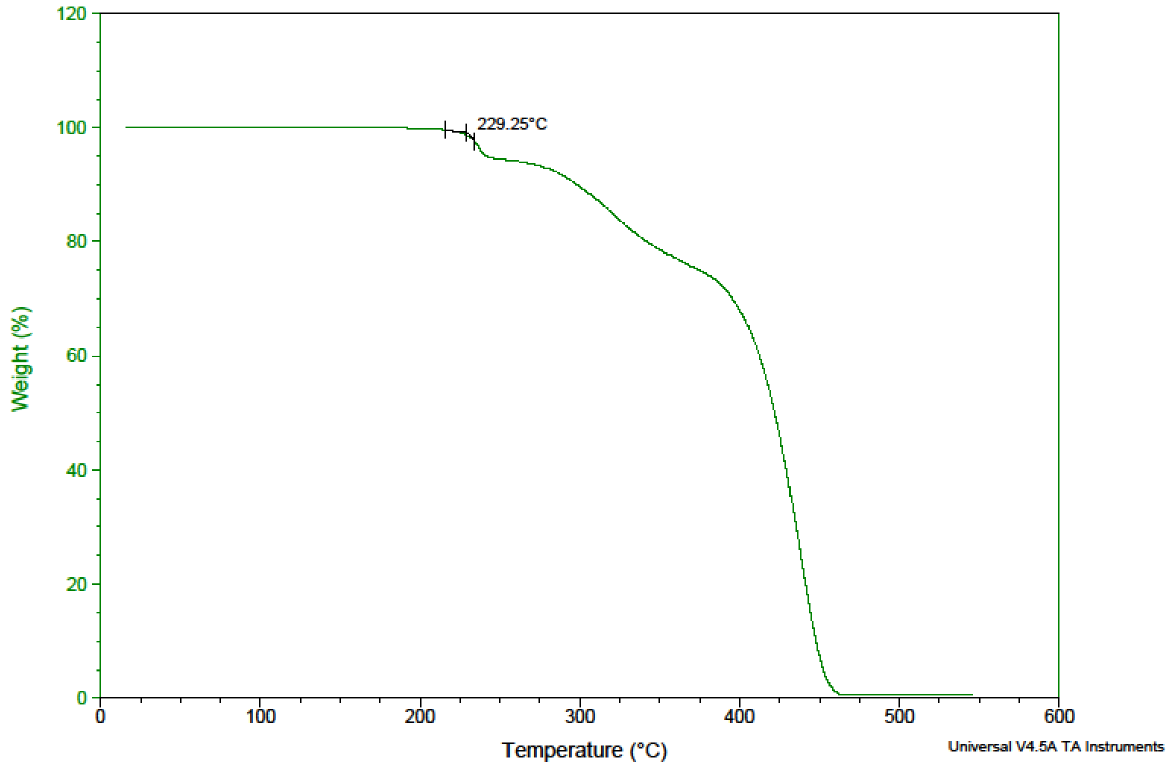

Figure S 66 TGA thermogram of **CEPU2** at 10 °C min^{-1} under nitrogen.

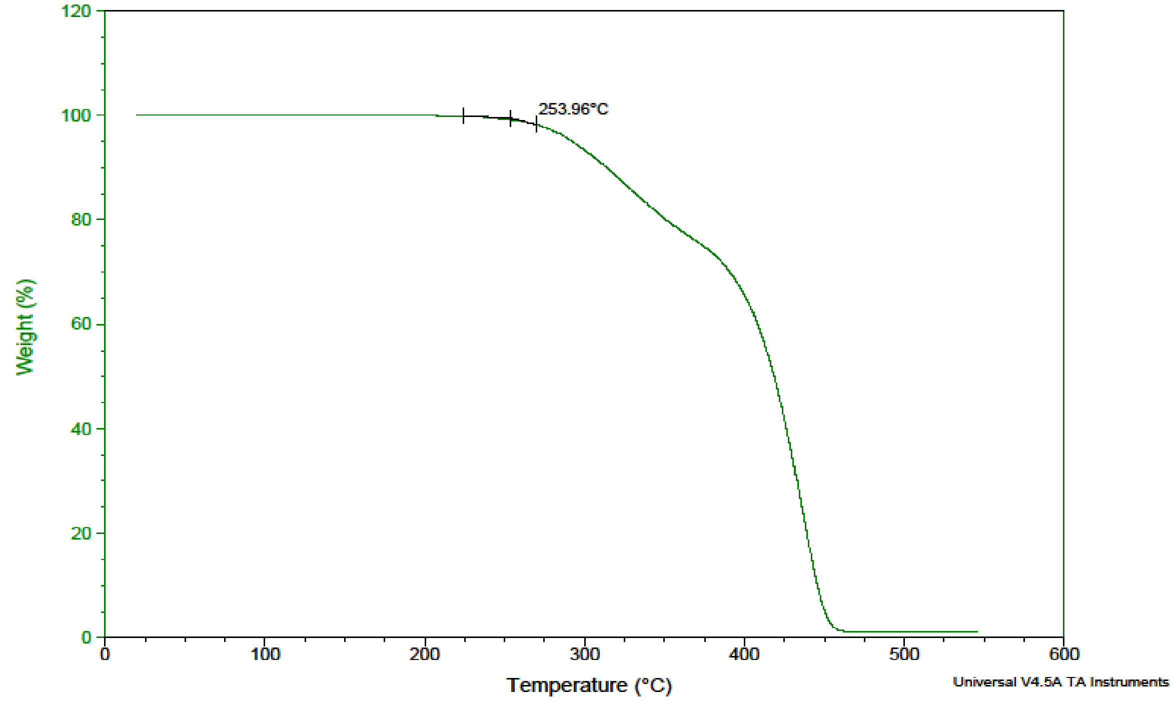

Figure S 67 TGA thermogram of **CEPU3** at 10 °C min^{-1} under nitrogen.

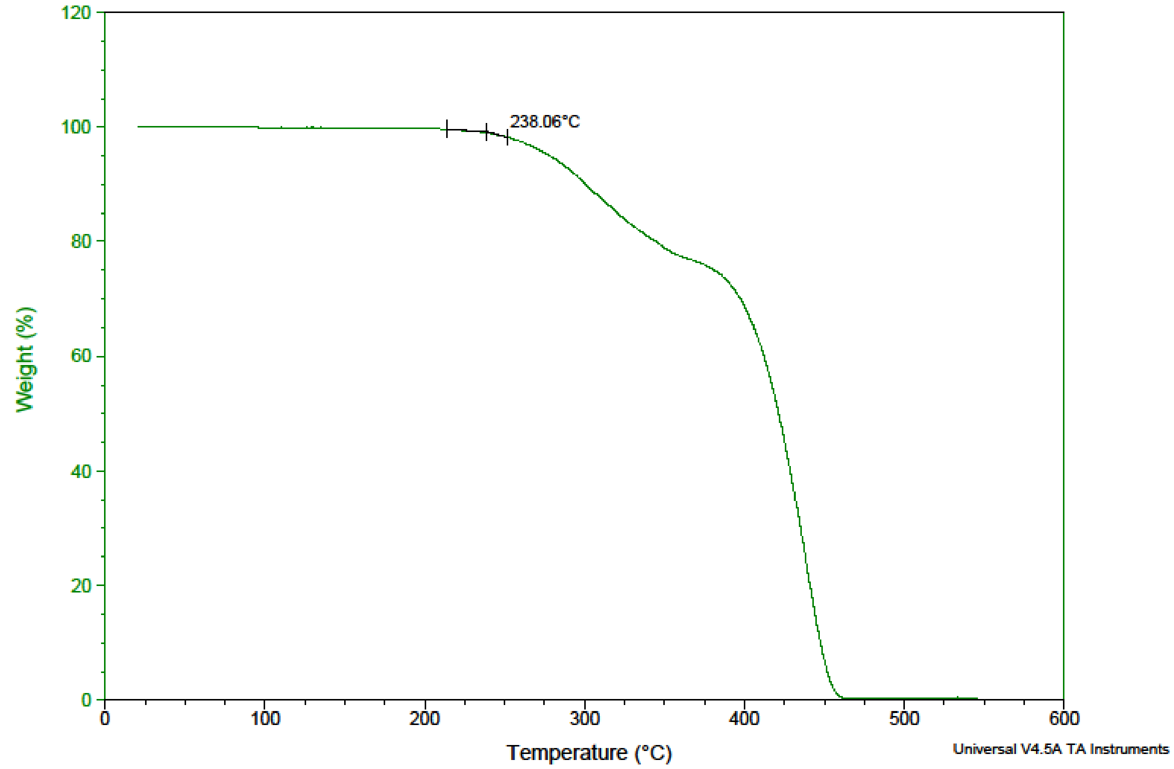

Figure S 68 TGA thermogram of **CEPU4** at 10 °C min^{-1} under nitrogen.

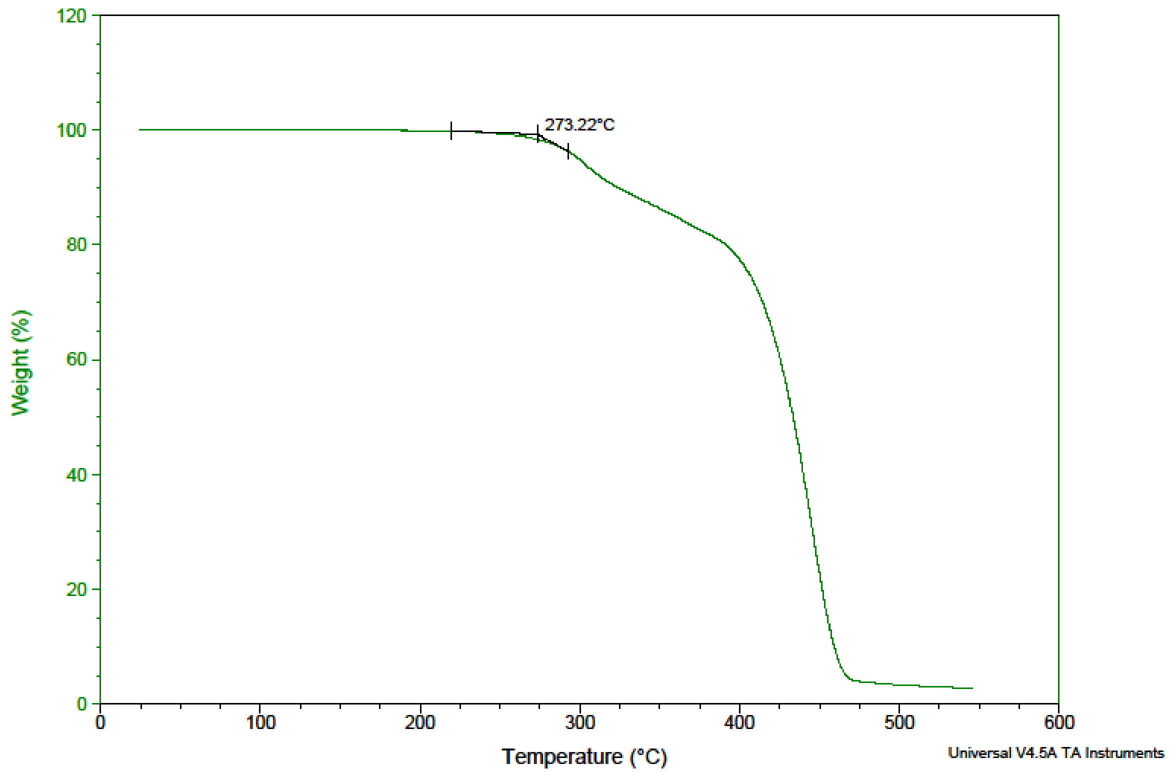

Figure S 69 TGA thermogram of **CEPU5** at 10 °C min^{-1} under nitrogen.

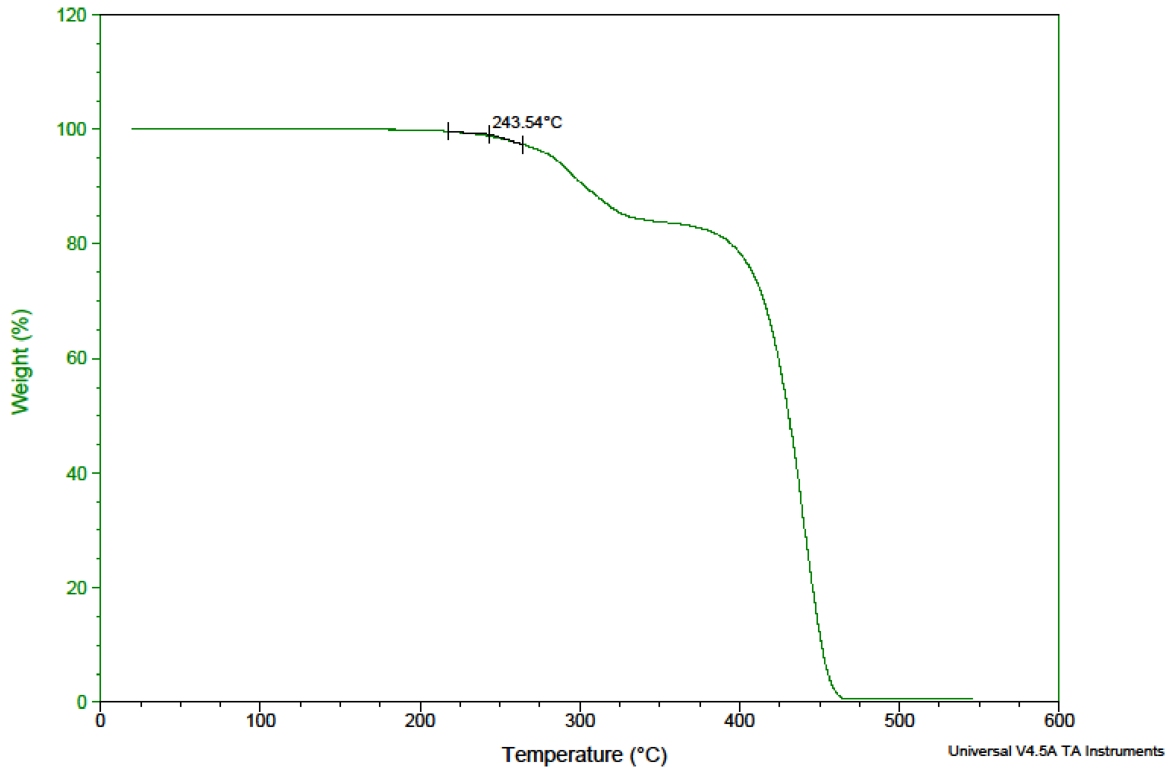

Figure S 70 TGA thermogram of **CEPU6** at 10 °C min^{-1} under nitrogen.

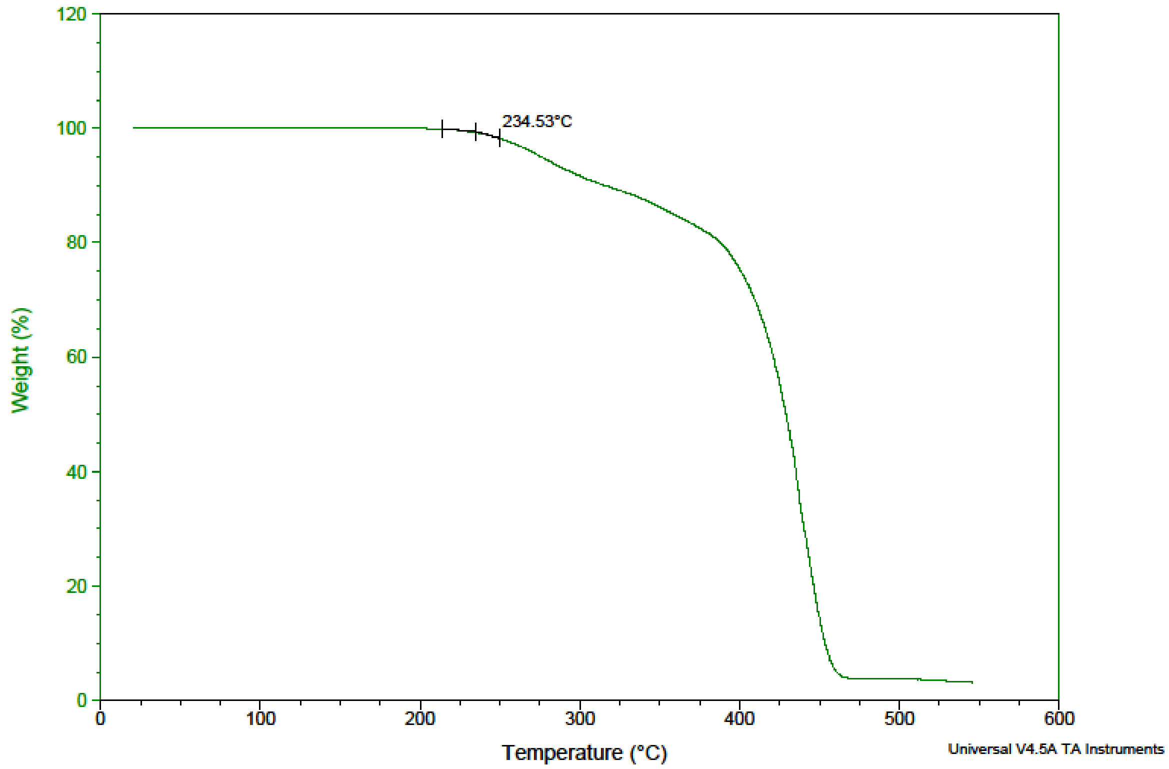

Figure S 71 TGA thermogram of **MeO-PU1** at 10 °C min^{-1} under nitrogen.

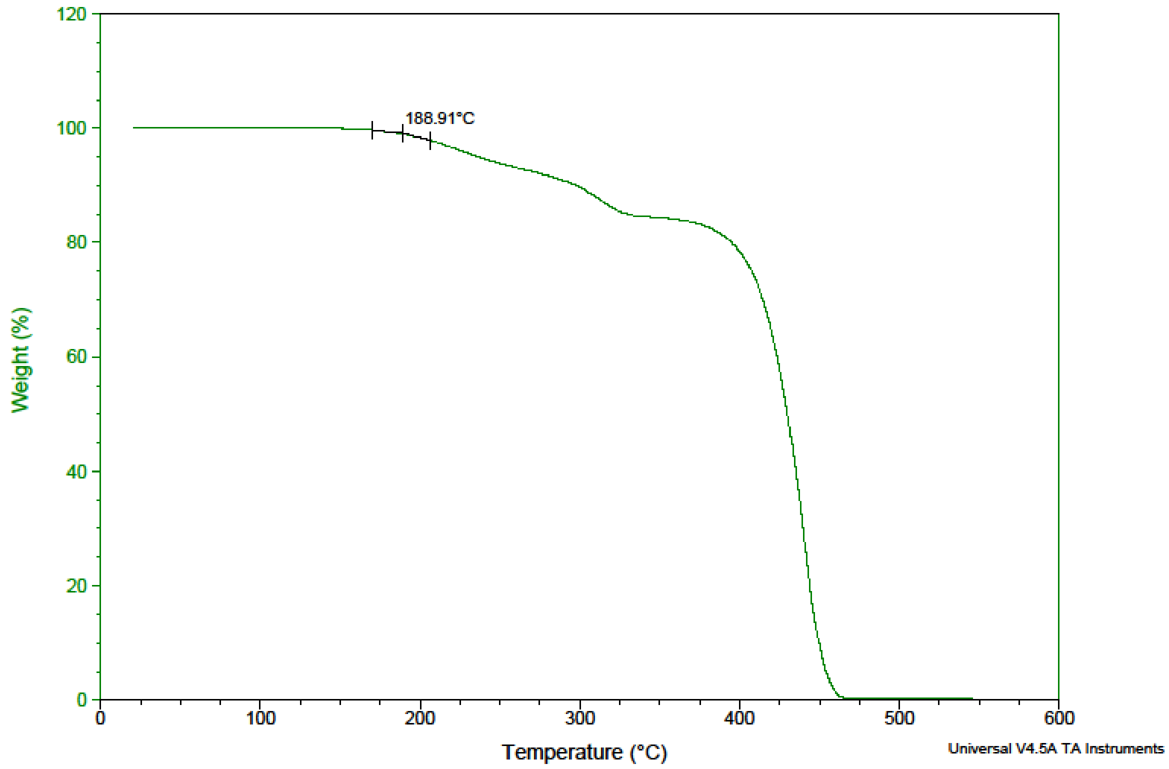

Figure S 72 TGA thermogram of **MeO-PU2** at 10 °C min^{-1} under nitrogen.

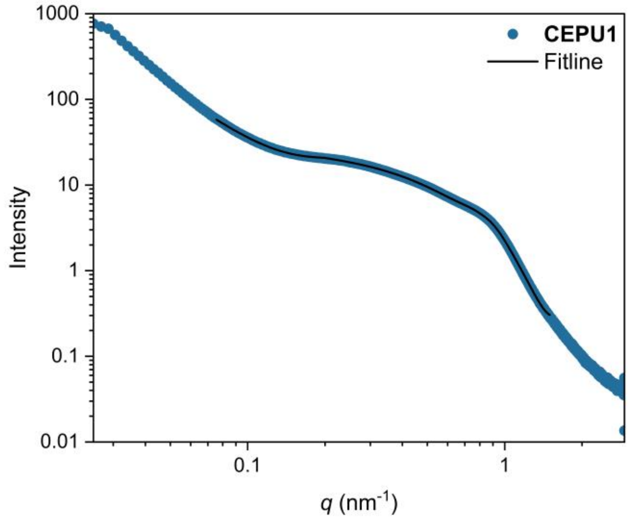

Figure S 73 SAXS profile of **CEPU1** and corresponding fitline at 20 °C.

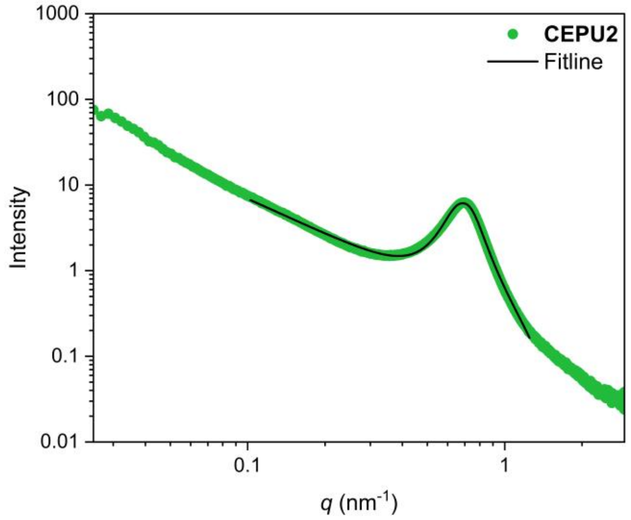

Figure S 74 SAXS profile of **CEPU2** and corresponding fitline at 20 °C.

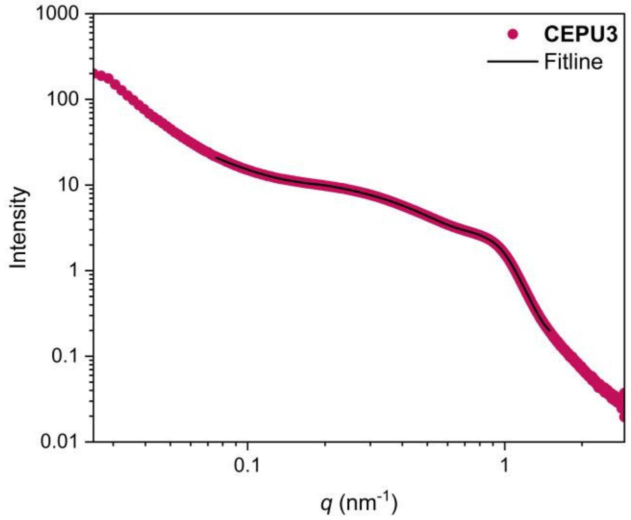

Figure S 75 SAXS profile of **CEPU3** and corresponding fitline at 20 °C.

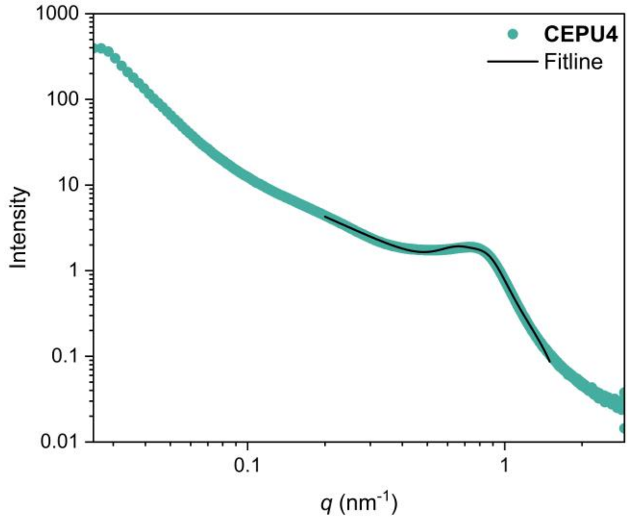

Figure S 76 SAXS profile of **CEPU4** and corresponding fitline at 20 °C.

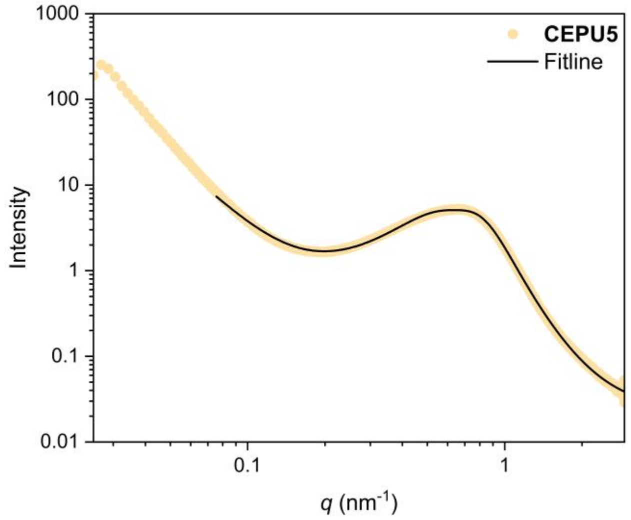

Figure S 77 SAXS profile of **CEPU5** and corresponding fitline at 20 °C.

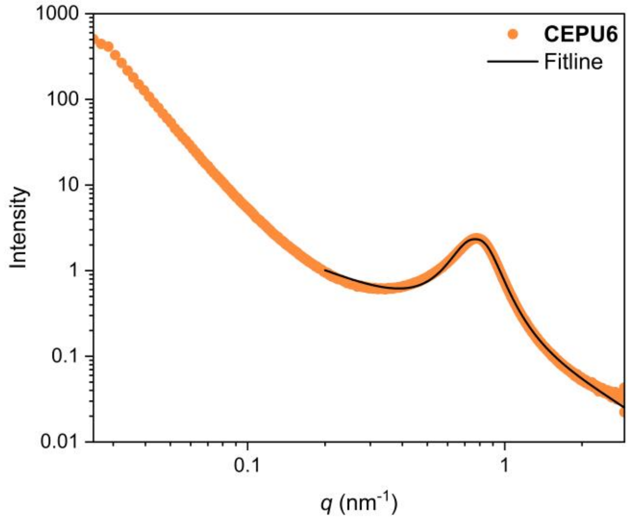

Figure S 78 SAXS profile of **CEPU6** and corresponding fitline at 20 °C.

Table S 20 q_{max} and corresponding *d*-spacing for **CEPU1**-**CEPU6** at 20 °C.

CEPU	q_{max} (nm^{-1})	*d*-spacing (nm)
**CEPU1**	0.2; 0.8	31.4; 7.9
**CEPU2**	0.7	9.0
**CEPU3**	0.2; 0.9	31.4; 7.0
**CEPU4**	0.7	9.0
**CEPU5**	0.6	10.5
**CEPU6**	0.8	7.9

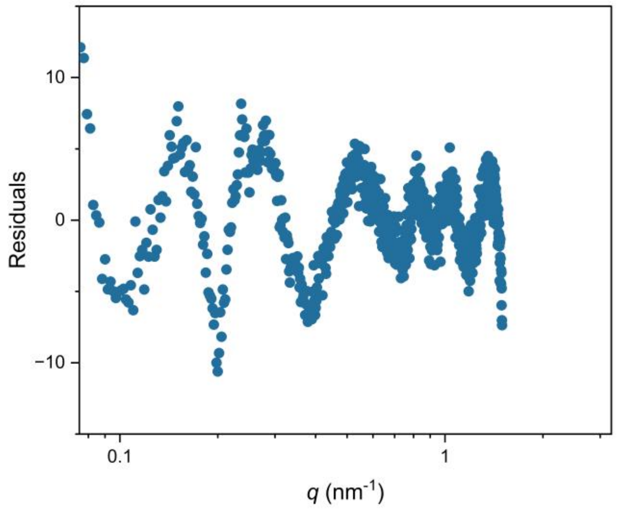

Figure S 79 SAXS fitting residuals for **CPEU1** at 20 °C.

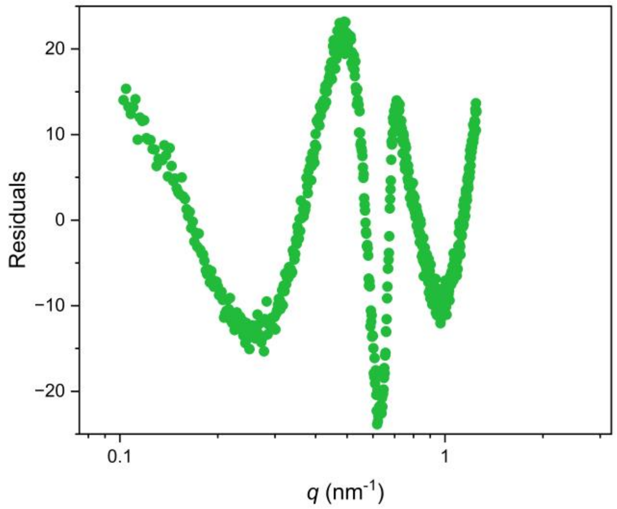

Figure S 80 SAXS fitting residuals for **CPEU2** at 20 °C.

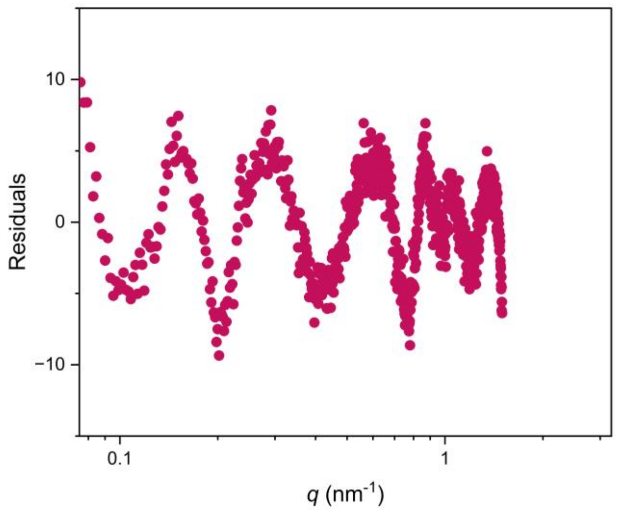

Figure S 81 SAXS fitting residuals for **CPEU3** at 20 °C.

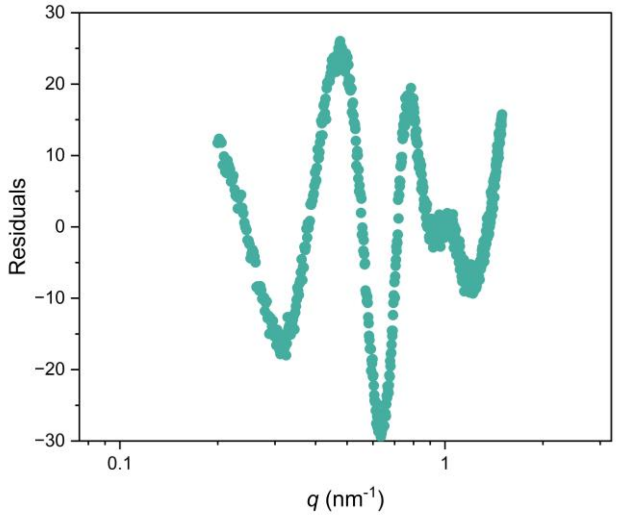

Figure S 82 SAXS fitting residuals for **CPEU4** at 20 °C.

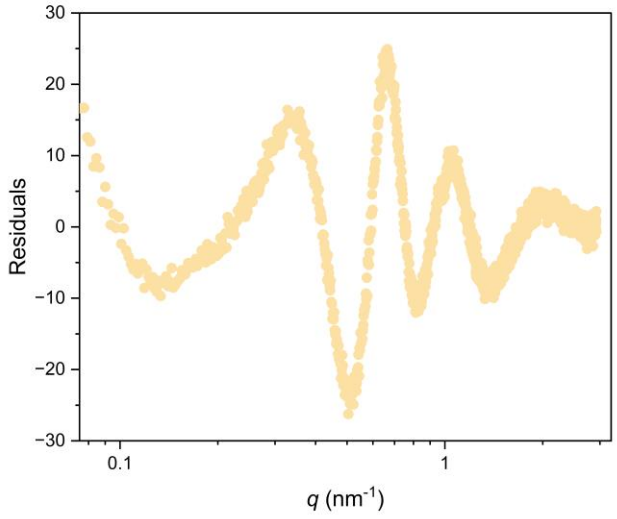

Figure S 83 SAXS fitting residuals for **CPEU5** at 20 °C.

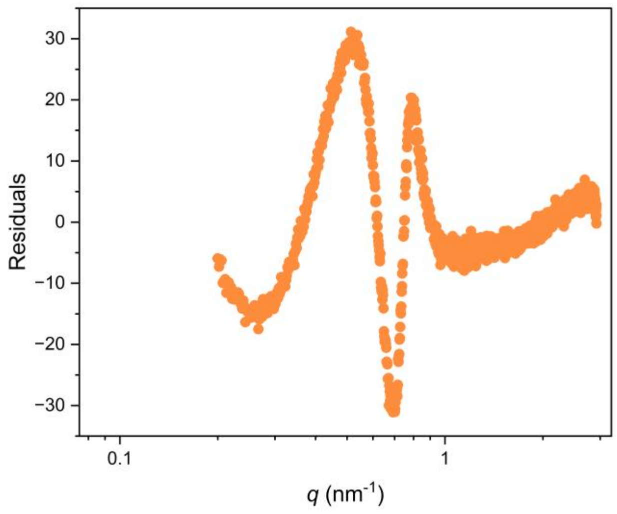

Figure S 84 SAXS fitting residuals for **CPEU6** at 20 °C.

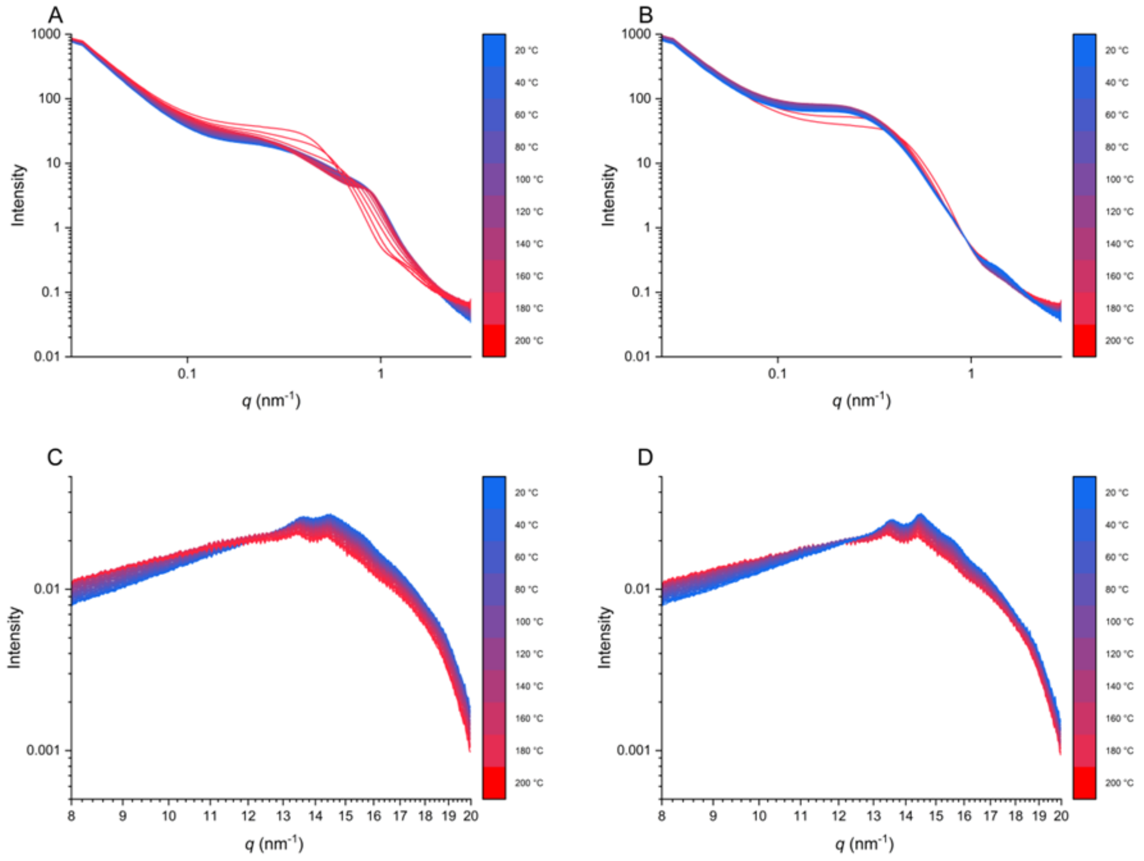

Figure S 85 VT-SAXS (**A** and **B**) and VT-WAXS (**C** and **D**) profiles of **CEPU1** at a heating and cooling rate of 10 °C; **A** and **C** – heating cycle from 20-200 °C, **B** and **D** – cooling cycle from 200-20 °C.

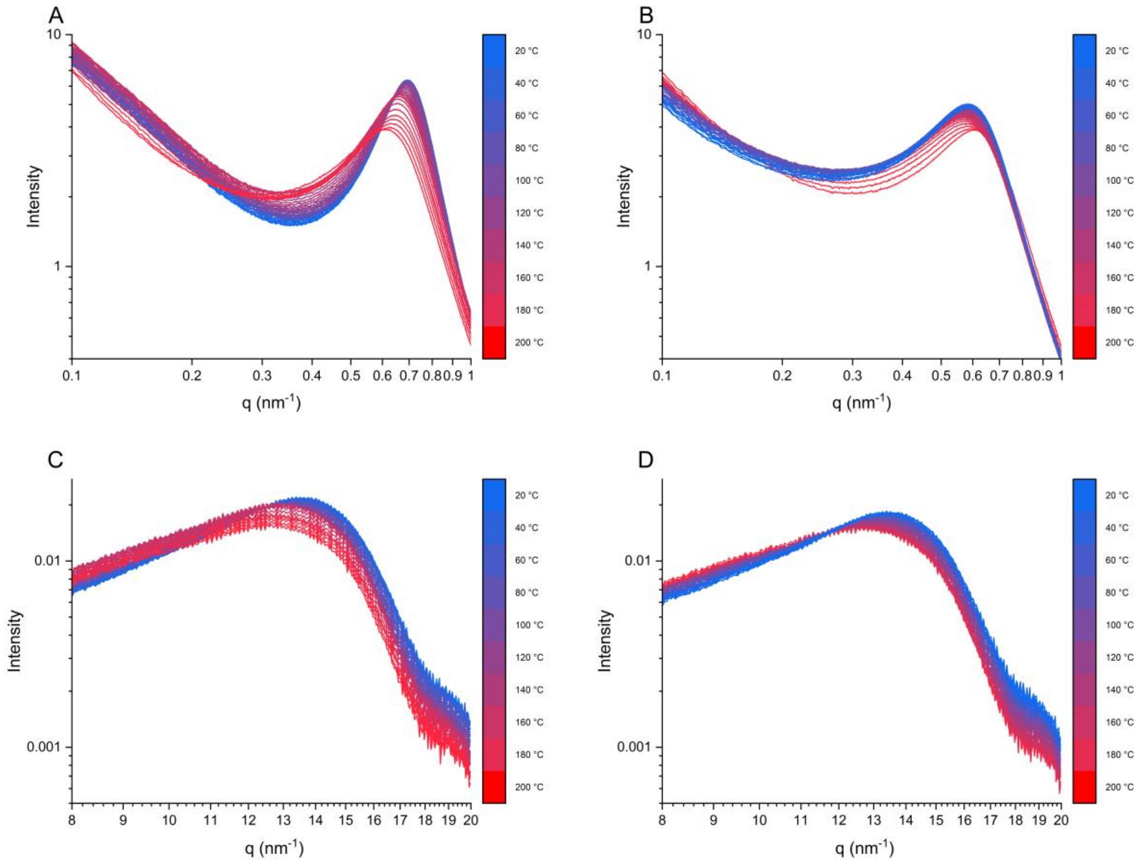

Figure S 86 VT-SAXS (**A** and **B**) and VT-WAXS (**C** and **D**) profiles of **CEPU2** at a heating and cooling rate of 10 °C; **A** and **C** – heating cycle from 20-200 °C, **B** and **D** – cooling cycle from 200-20 °C.

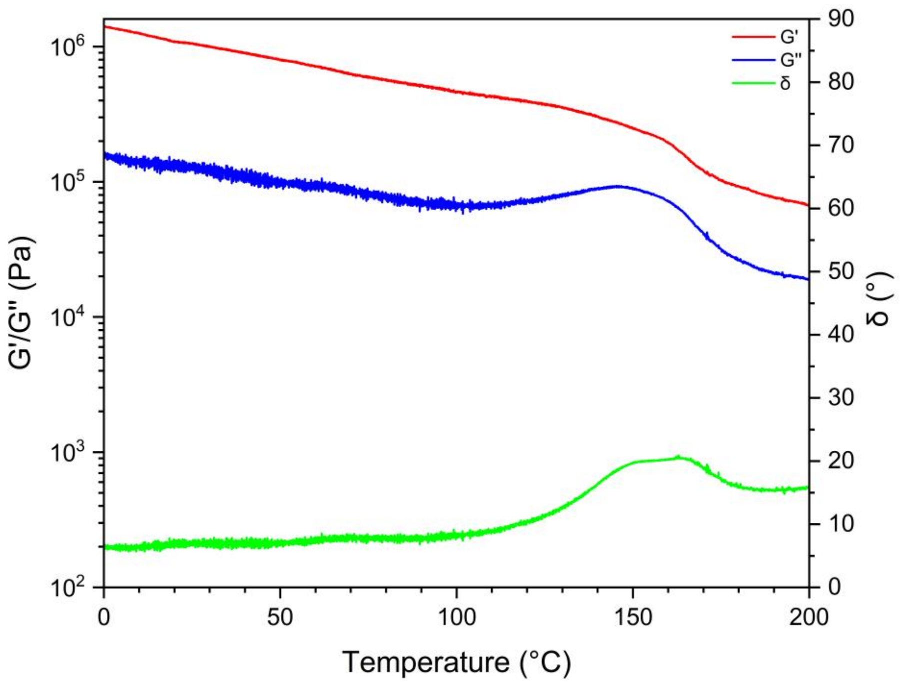

Figure S 87 Temperature sweep analysis of **CEPU1** over a temperature regime of 0 °C to 200 °C, using a normal force of 1 N and a frequency of 1 Hz. With G' G' (red), G'' (blue), and phase shift (δ) (green) against temperature.

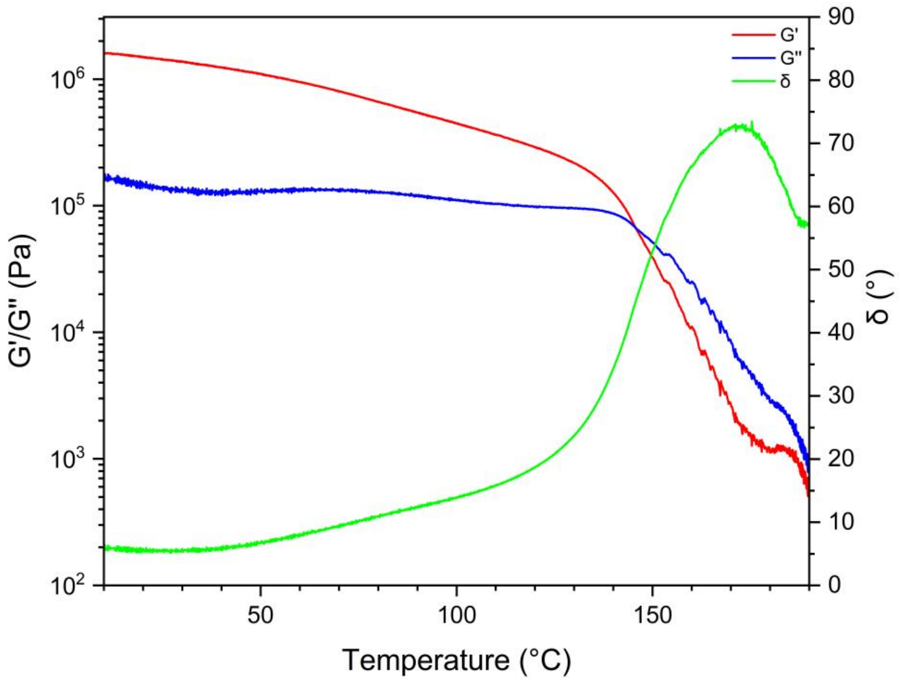

Figure S 88 Temperature sweep analysis of **CEPU2** over a temperature regime of 10 °C to 190 °C, using a normal force of 1 N and a frequency of 1 Hz. With G' (red), G'' (blue), and phase shift (δ) (green) against temperature.

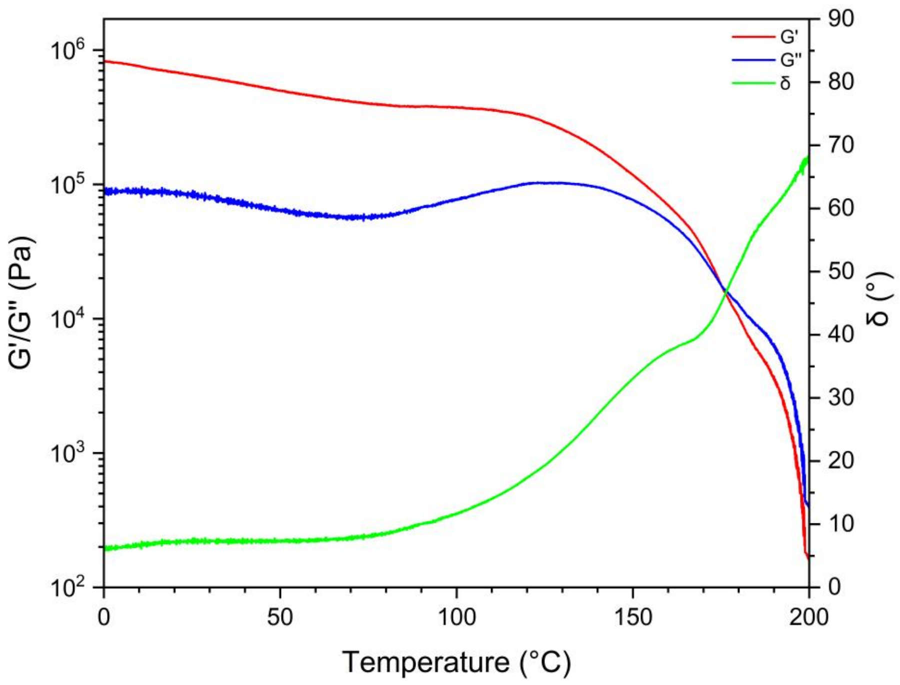

Figure S 89 Temperature sweep analysis of **CEPU3** over a temperature regime of 0 °C to 200 °C, using a normal force of 1 N and a frequency of 1 Hz. With G' (red), G'' (blue), and phase shift (δ) (green) against temperature.

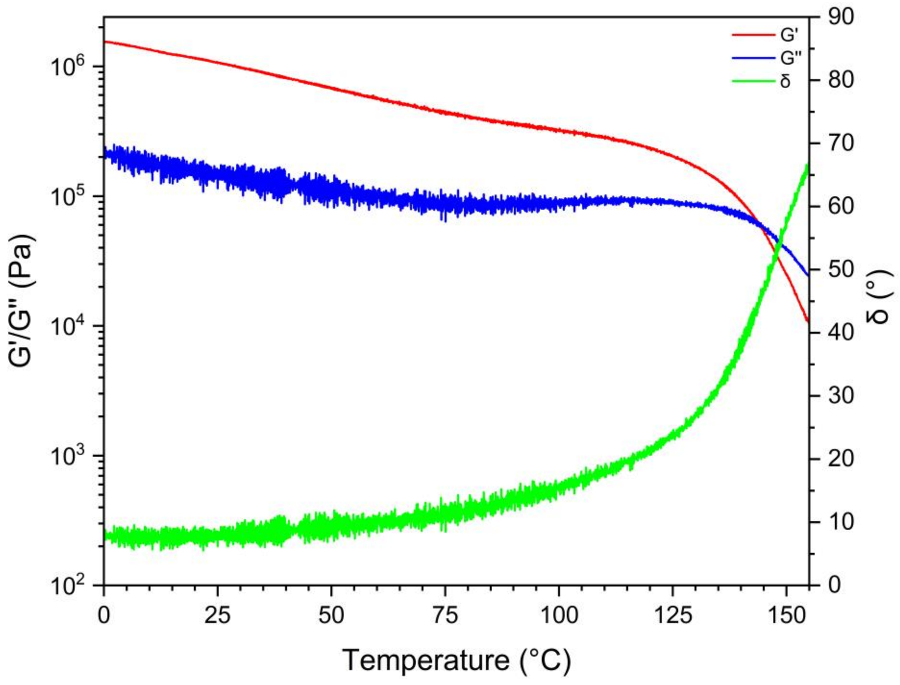

Figure S 90 Temperature sweep analysis of **CEPU4** over a temperature regime of 0 °C to 160 °C, using a normal force of 1 N and a frequency of 1 Hz. With G' (red), G'' (blue), and phase shift (δ) (green) against temperature.

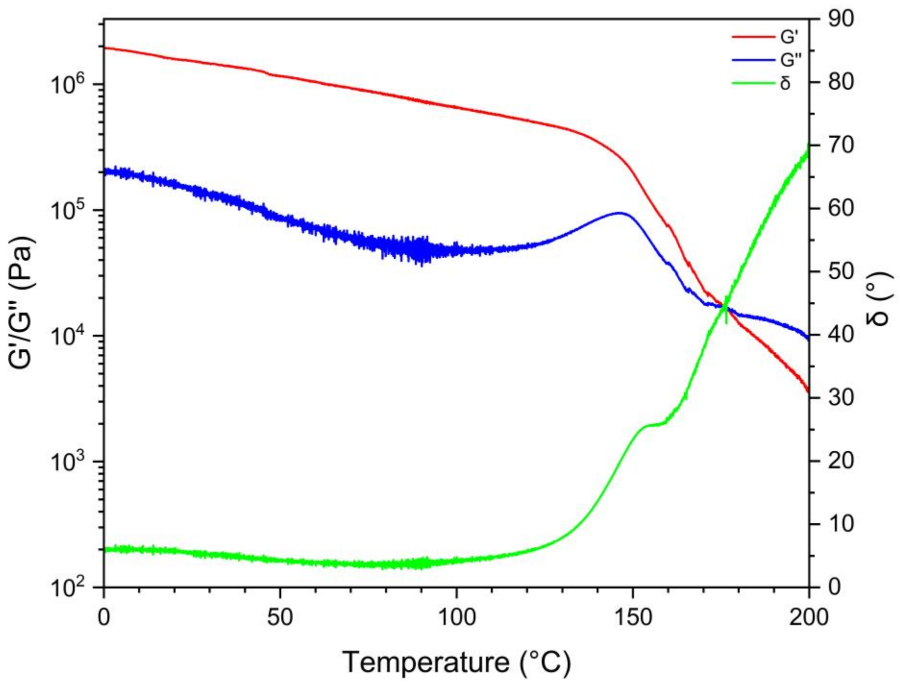

Figure S 91 Temperature sweep analysis of **CEPU5** over a temperature regime of 0 °C to 200 °C, using a normal force of 1 N and a frequency of 1 Hz. With G' (red), G'' (blue), and phase shift (δ) (green) against temperature.

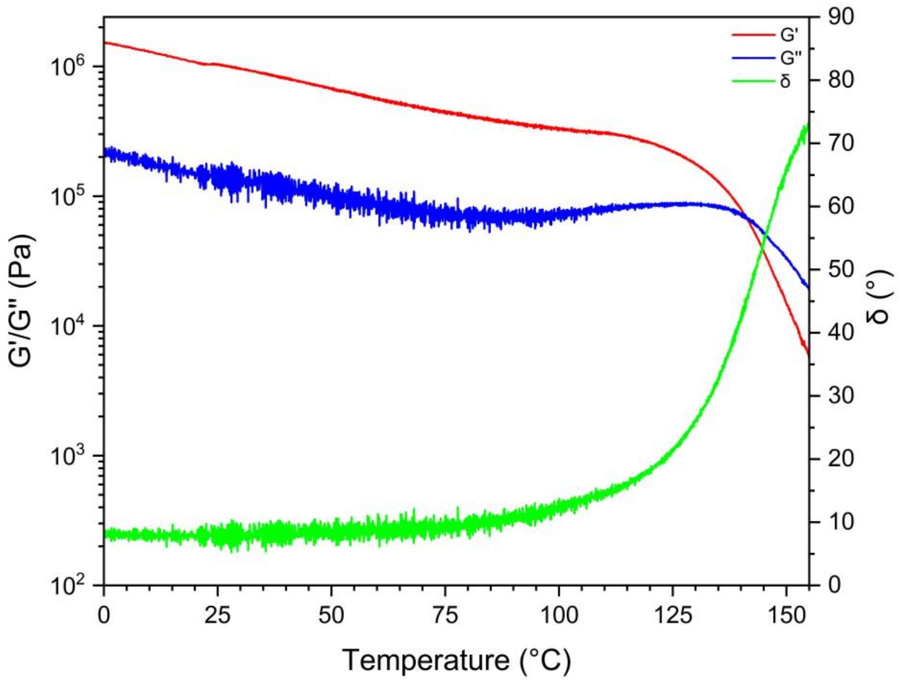

Figure S 92 Temperature sweep analysis of **CEPU6** over a temperature regime of 0 °C to 160 °C, using a normal force of 1 N and a frequency of 1 Hz. With G' (red), G'' (blue), and phase shift (δ) (green) against temperature.

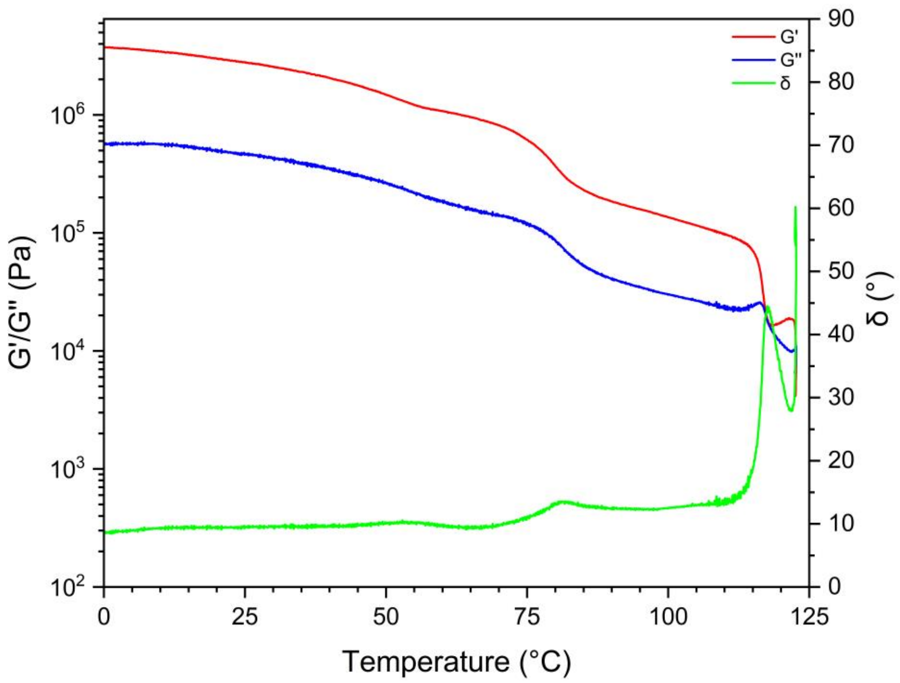

Figure S 93 Temperature sweep analysis of **MeO-PU1** over a temperature regime of 0 °C to 122 °C, using a normal force of 1 N and a frequency of 1 Hz. With G' (red), G'' (blue), and phase shift (δ) (green) against temperature.

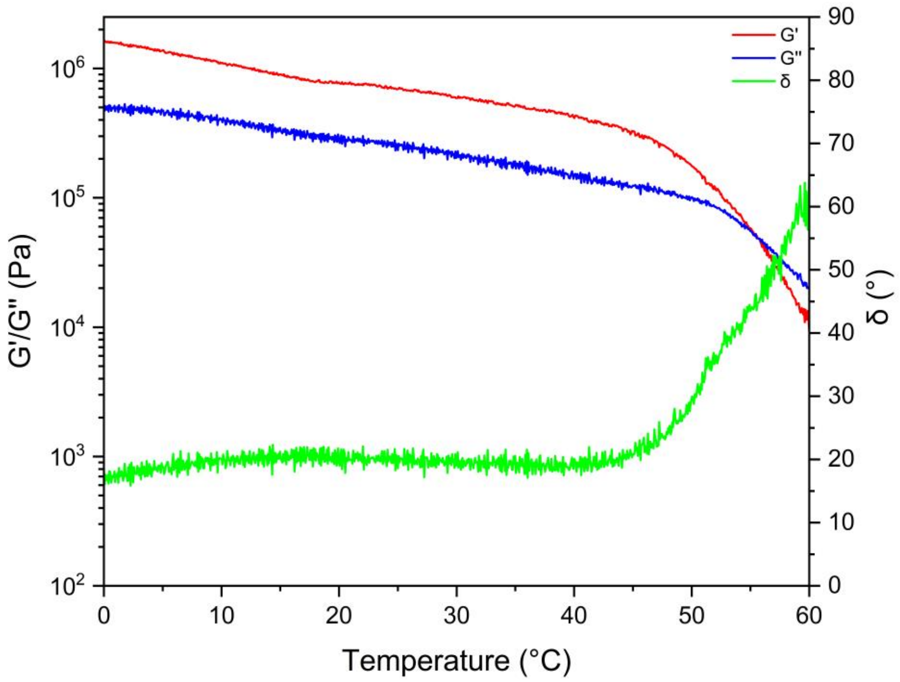

Figure S 94 Temperature sweep analysis of **MeO-PU2** over a temperature regime of 0 °C to 60 °C, using a normal force of 1 N and a frequency of 1 Hz. With G' (red), G'' (blue), and phase shift (δ) (green) against temperature.

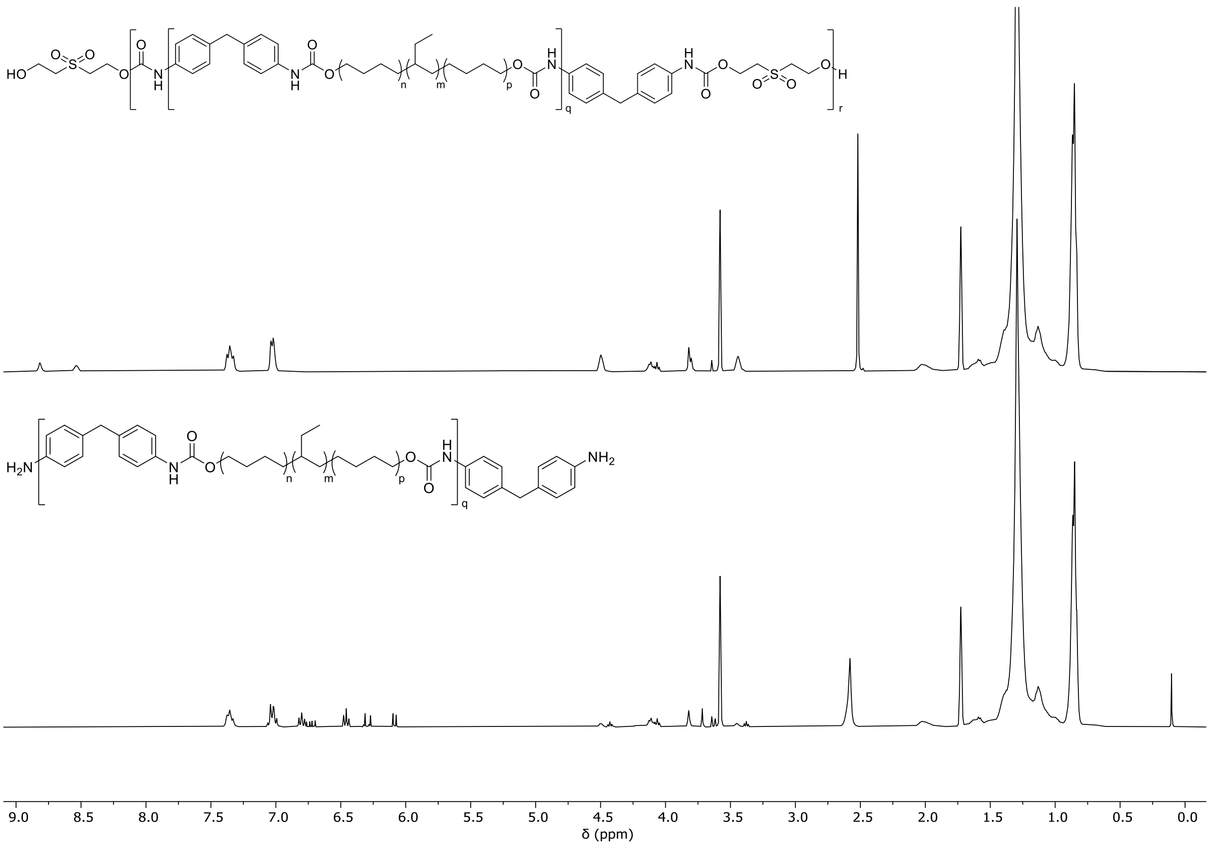

Figure S 95 ^{1}H NMR spectra showing the solution degradation of **CEPU1** with 40 wt.% NaOD in D_2O, (400 MHz, THF-d_8). Top spectrum shows the pristine CEPU, bottom spectrum shows the degraded CEPU.

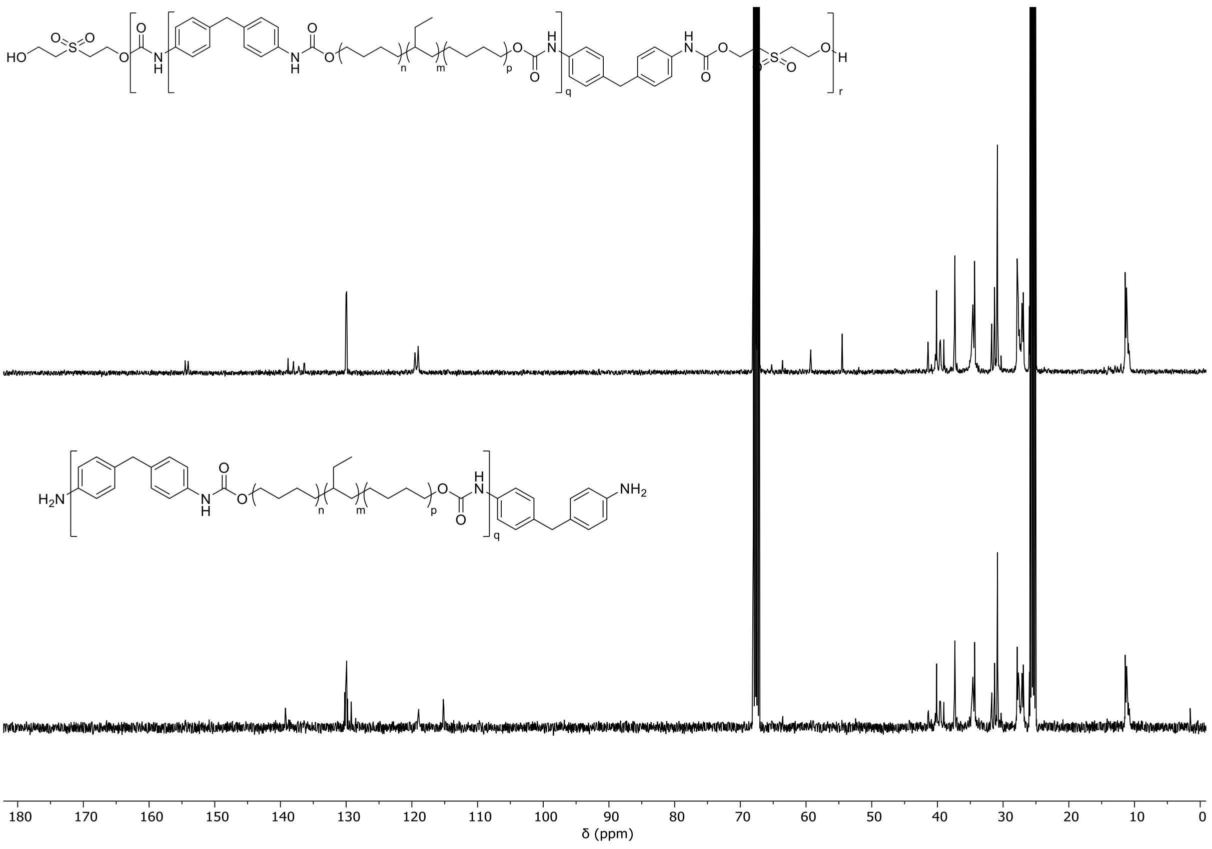

Figure S 96 ^{13}C {H} NMR spectra showing the solution degradation of **CEPU1** with 40 wt.% NaOD in D_2O, (400 MHz, THF-d_8). Top spectrum shows the pristine CEPU, bottom spectrum shows the degraded CEPU.

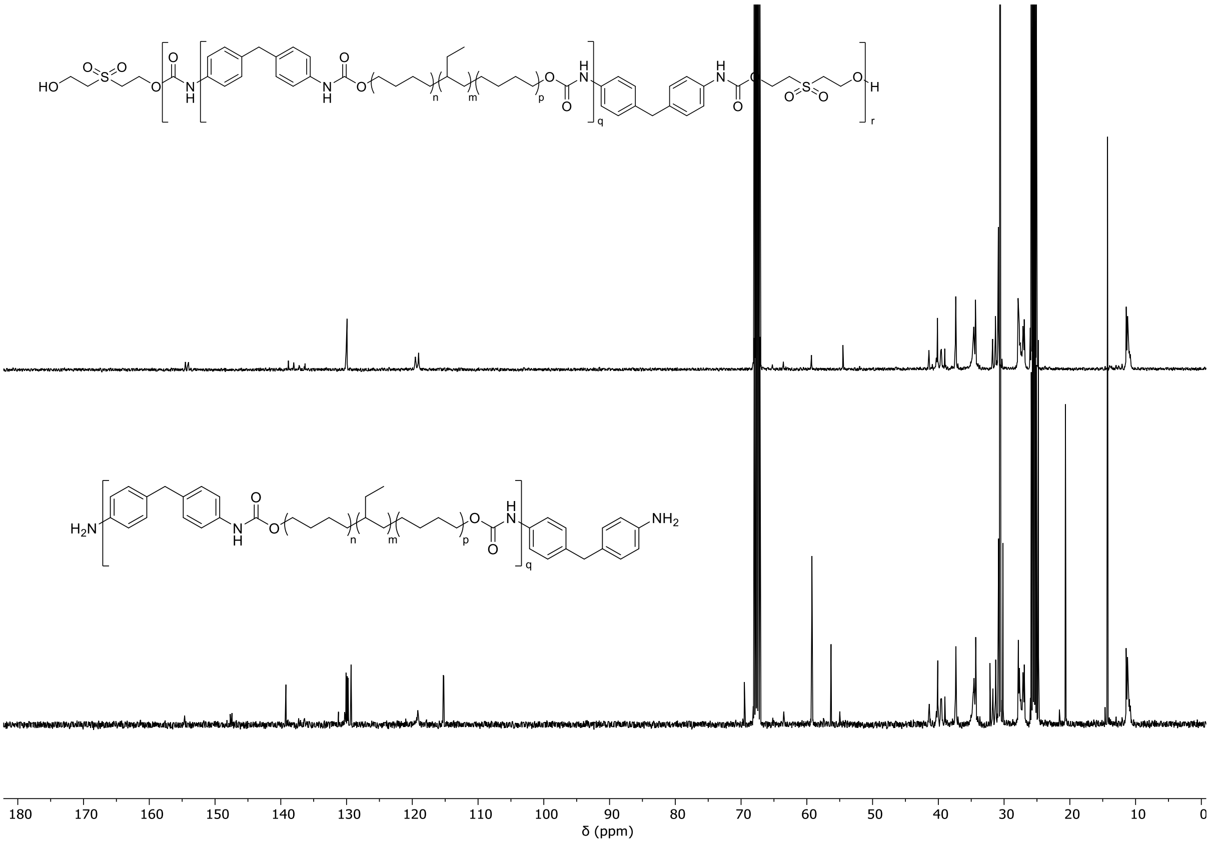

Figure S 97 ^{13}C {H} NMR spectra showing the solution degradation of **CEPU1** with 1M TBAF in acetone, (400 MHz, THF-d_8). Top spectrum shows the pristine CEPU, bottom spectrum shows the degraded CEPU.

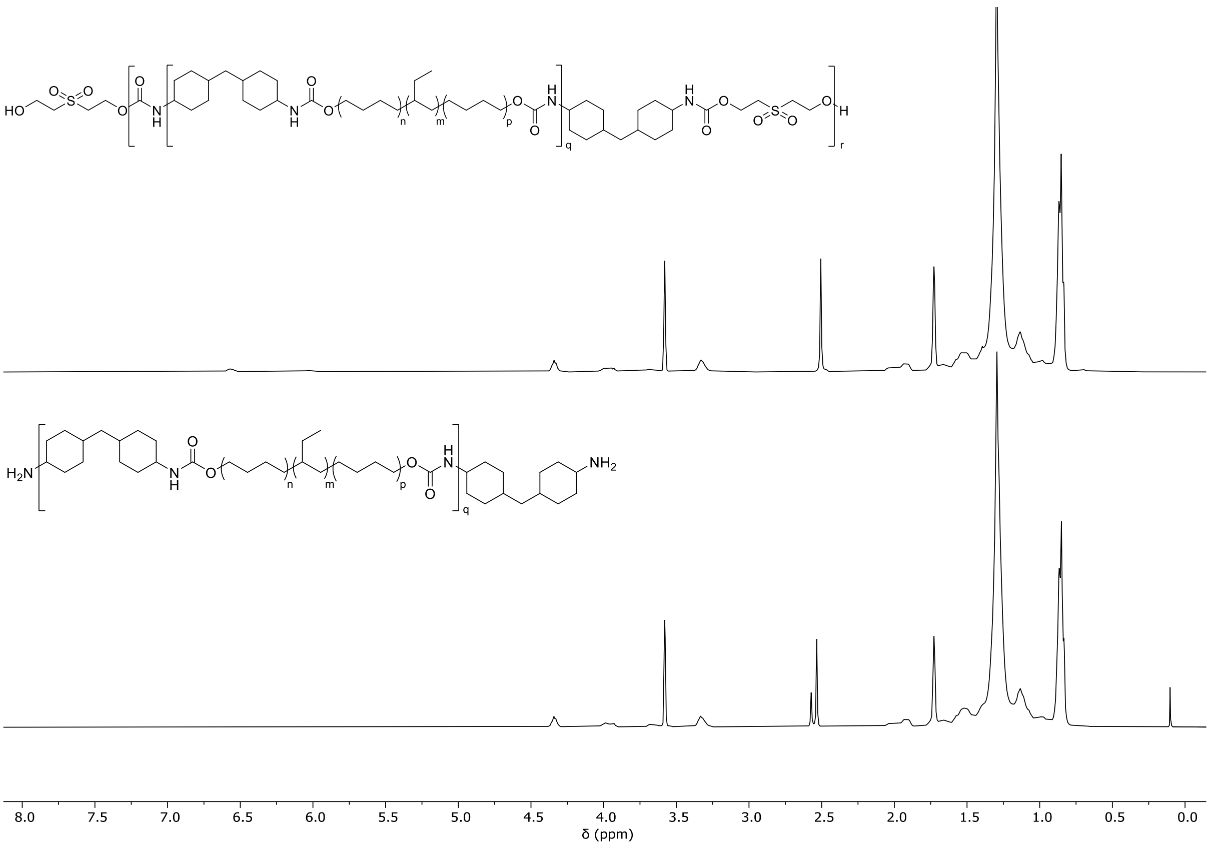

Figure S 98 ^{1}H NMR spectra showing the solution degradation of **CEPU2** with 40 wt.% NaOD in D_2O, (400 MHz, THF-d_8). Top spectrum shows the pristine CEPU, bottom spectrum shows the degraded CEPU.

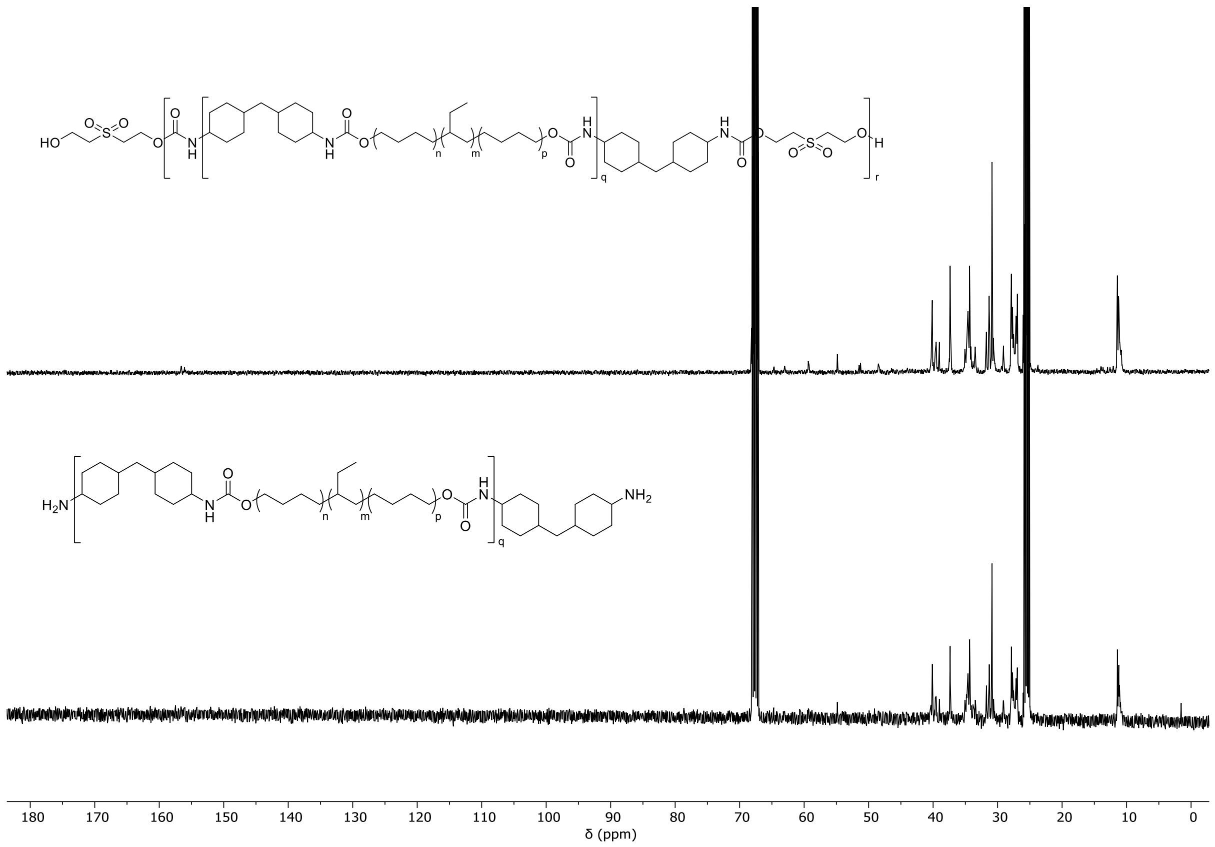

Figure S 99 ^{13}C {H} NMR spectra showing the solution degradation of **CEPU2** with 40 wt.% NaOD in D_2O, (400 MHz, THF-*d_8*). Top spectrum shows the pristine CEPU, bottom spectrum shows the degraded CEPU.

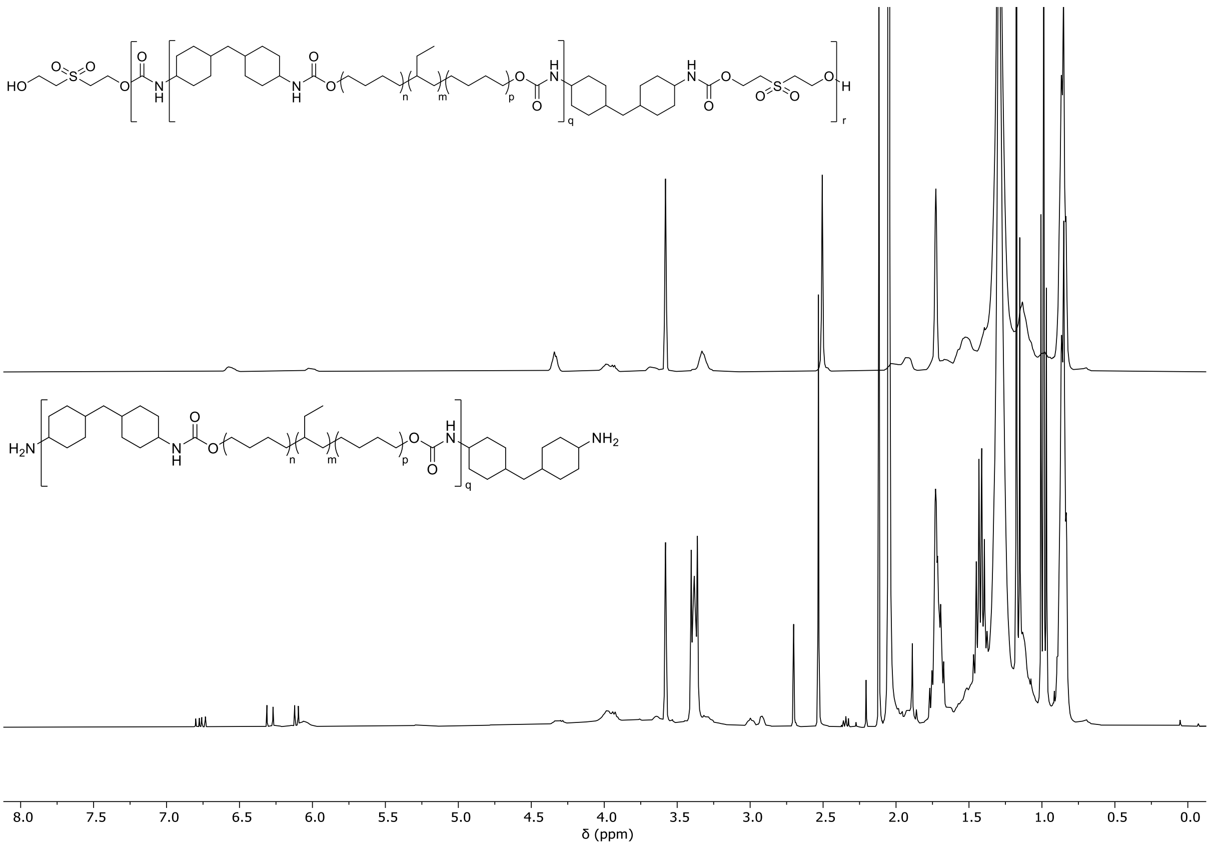

Figure S 100 ^{1}H NMR spectra showing the solution degradation of **CEPU2** with 1M TBAF in acetone, (400 MHz, THF-d_8). Top spectrum shows the pristine CEPU, bottom spectrum shows the degraded CEPU.

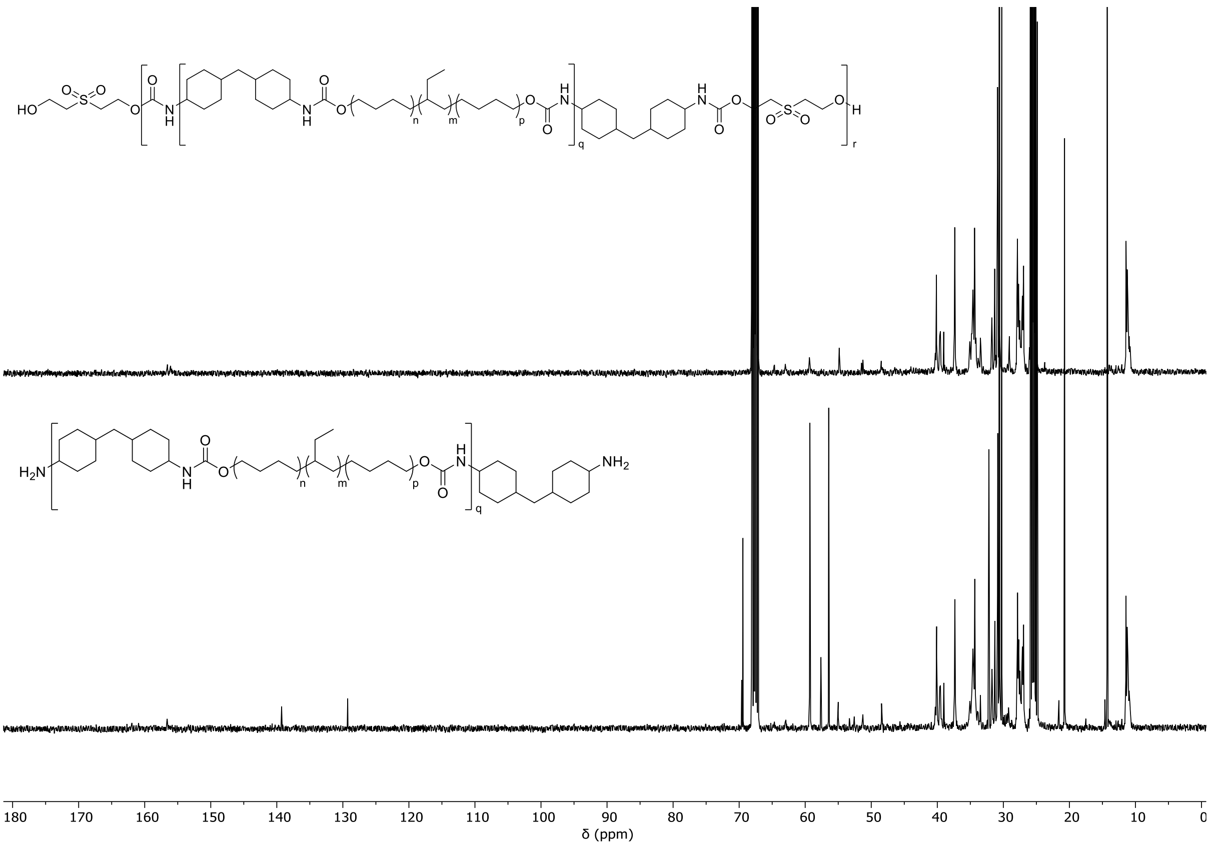

Figure S 101 ^{13}C {H} NMR spectra showing the solution degradation of **CEPU2** with 1M TBAF in acetone, (400 MHz, THF-*d₈*). Top spectrum shows the pristine CEPU, bottom spectrum shows the degraded CEPU.

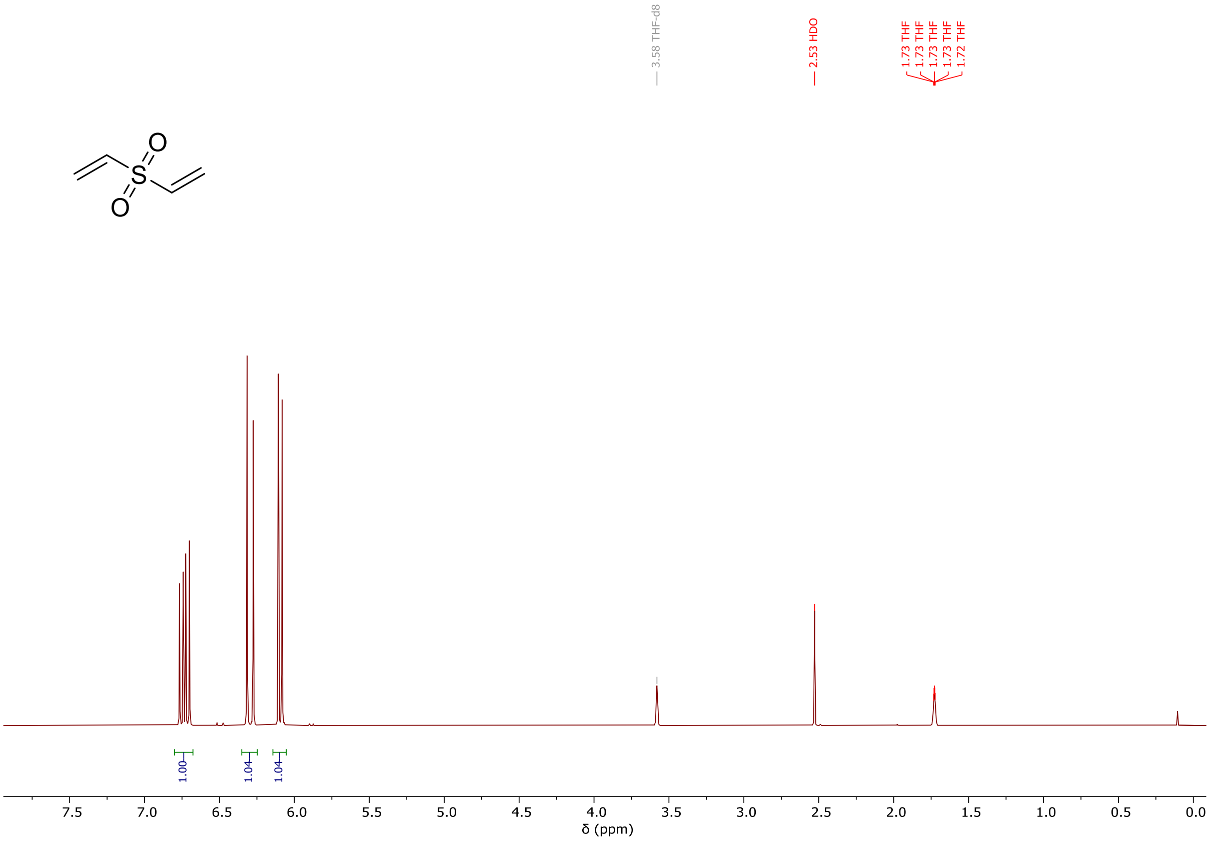

Figure S 102 ^{1}H NMR spectrum of divinyl sulfone (400 MHz, THF-d_8, 298 K).

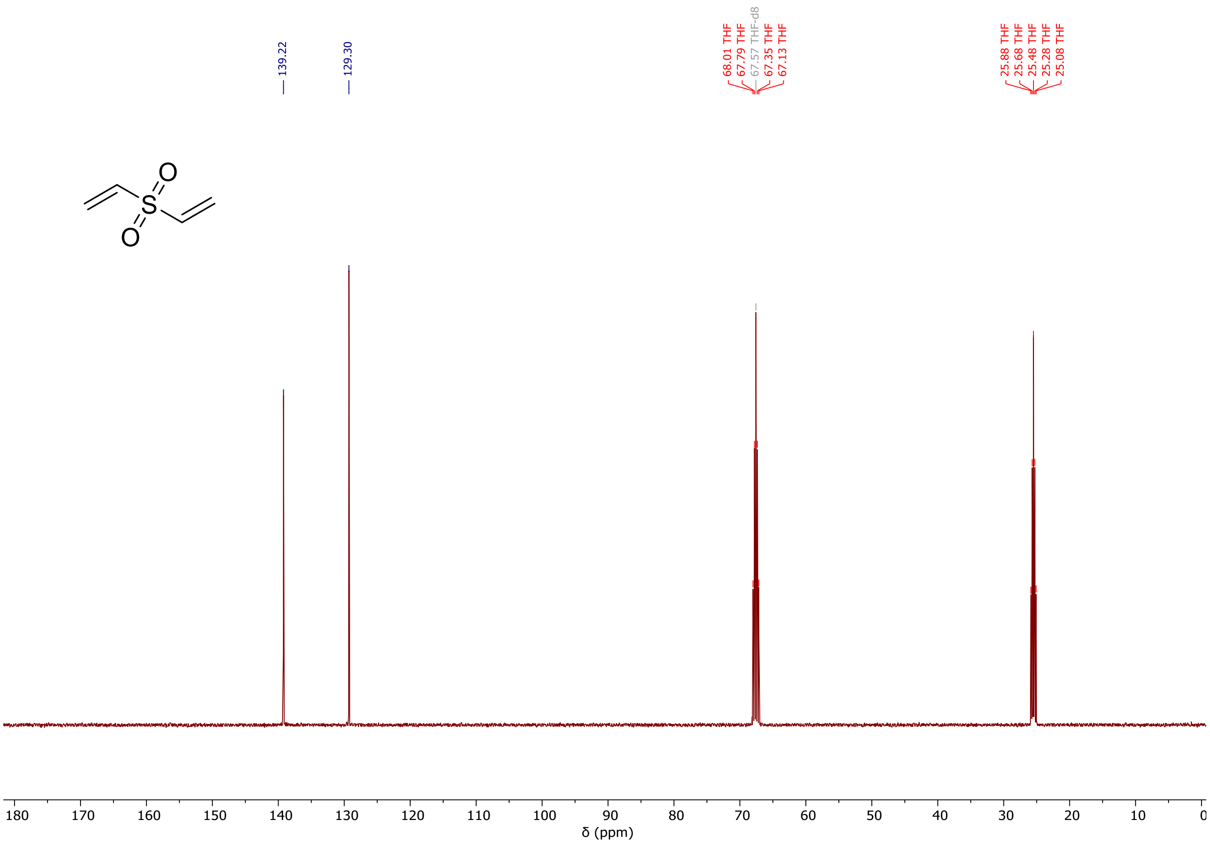

Figure S 103 ^{13}C {H} NMR spectrum of divinyl sulfone (400 MHz, THF-d_8, 298 K).

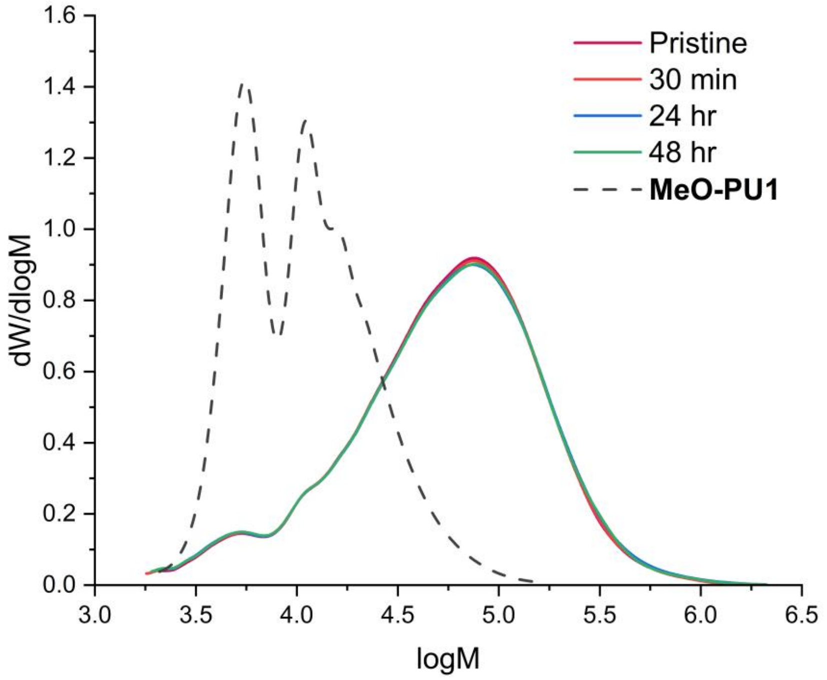

Figure S 104 GPC eluogram of **CEPU3** in THF as a pristine sample and 30 min, 24 hr, and 48 hr post addition of TBAF.

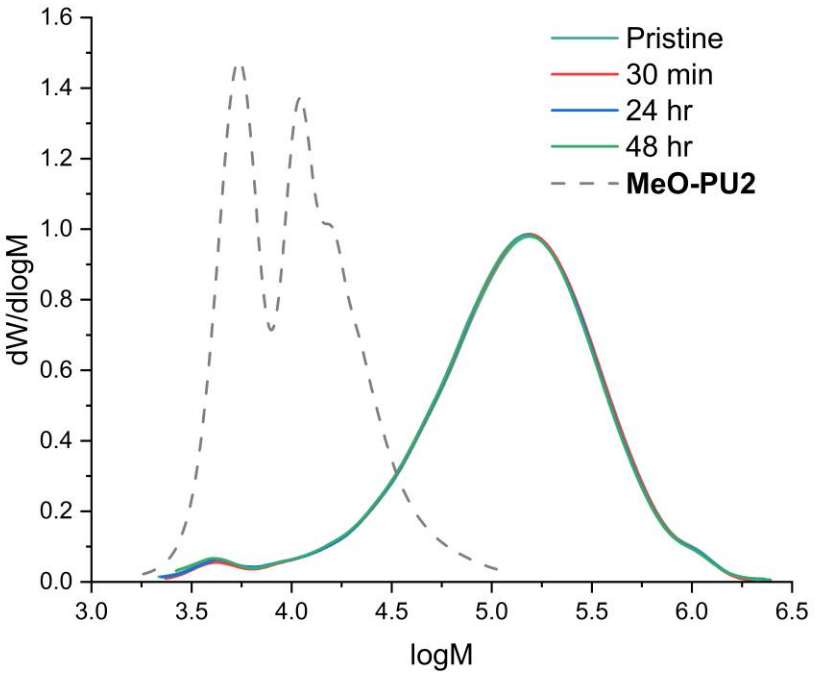

Figure S 105 GPC eluogram of **CEPU4** in THF as a pristine sample and 30 min, 24 hr, and 48 hr post addition of TBAF.

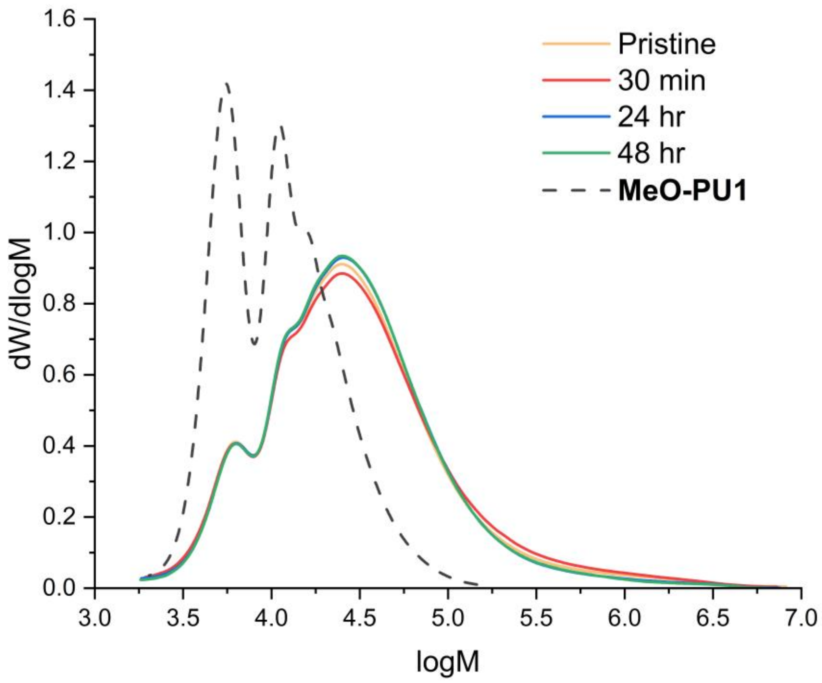

Figure S 106 GPC eluogram of **CEPU5** in THF as a pristine sample and 30 min, 24 hr, and 48 hr post addition of TBAF.

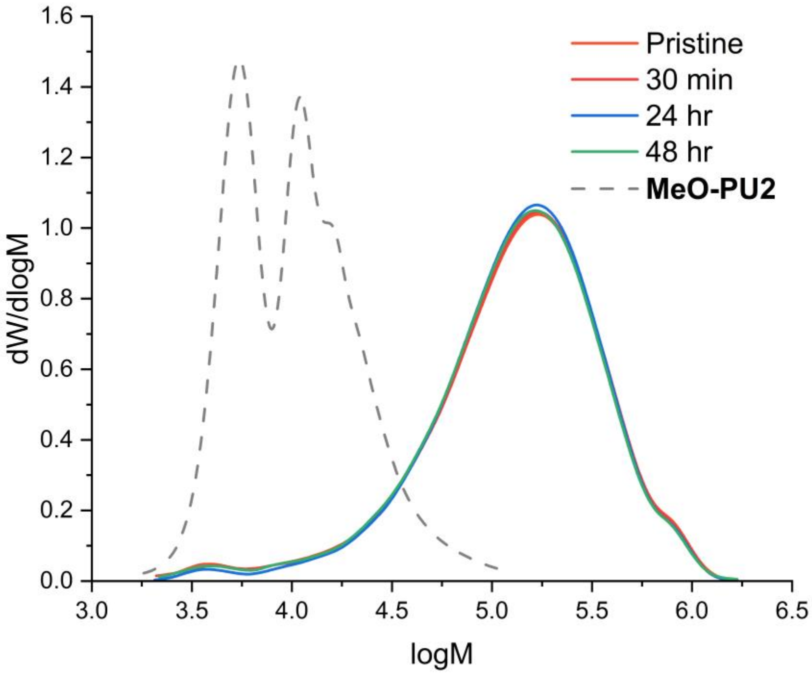

Figure S 107 GPC eluogram of **CEPU6** in THF as a pristine sample and 30 min, 24 hr, and 48 hr post addition of TBAF.

Table S 21 M_n and M_w of **CEPU1-CEPU6** as pristine samples and 30 min, 24 hr, and 48 hr post addition of TBAF acquired from a THF GPC; the recorded are averages of three separate samples of each CEPU. The error shown is the standard deviation between the three repeats of each sample.

CEPU	TBAF Exposure Time	M_n (g mol^{-1})	M_w (g mol^{-1})	Đ
**CEPU1**	Pristine	30100 ± 250	124900 ± 0	4.15
	30 minutes	7700 ± 0	12000 ± 0	1.56
	24 hours	7700 ± 0	12100 ± 0	1.57
	48 hours	7700 ± 0	12200 ± 0	1.58
**CEPU2**	Pristine	44700 ± 200	140400 ± 700	3.14
	30 minutes	7800 ± 200	15600 ± 500	2.00
	24 hours	7700 ± 0	14000 ± 0	1.82
	48 hours	8100 ± 0	15000 ± 200	1.85
**CEPU3**	Pristine	27700 ± 0	86900 ± 1600	3.14
	30 minutes	27800 ± 100	89400 ± 300	3.22
	24 hours	27600 ± 300	90300 ± 1100	3.27
	48 hours	26000 ± 200	90500 ± 900	3.48
**CEPU4**	Pristine	57400 ± 500	197000 ± 300	3.43
	30 minutes	57800 ± 800	195600 ± 300	3.38
	24 hours	56900 ± 100	194600 ± 300	3.42
	48 hours	56500 ± 200	193500 ± 300	3.42
**CEPU5**	Pristine	26600 ± 100	90900 ± 900	3.42
	30 minutes	26800 ± 0	93400 ± 900	3.49
	24 hours	27000 ± 100	91000 ± 1600	3.37
	48 hours	27000 ± 100	89600 ± 700	3.32
**CEPU6**	Pristine	64800 ± 800	199500 ± 100	3.08
	30 minutes	63200 ± 1100	199000 ± 200	3.15
	24 hours	66700 ± 1600	198300 ± 100	2.97
	48 hours	61100 ± 2000	197000 ± 800	3.22
**MeO-PU1**	Pristine	8500 ± 0	16300 ± 600	1.92
**MeO-PU2**	Pristine	7900 ± 100	13700 ± 300	1.73

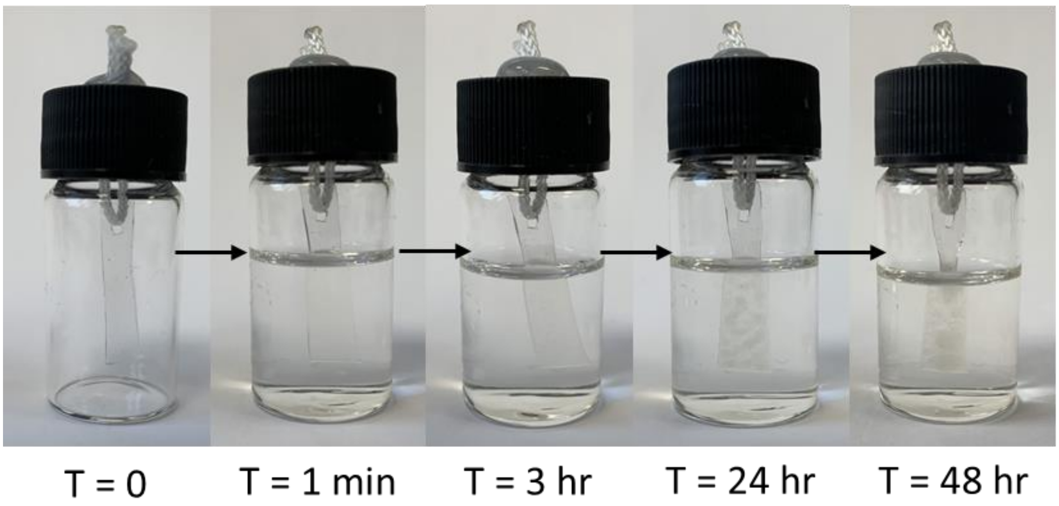

Figure S 108 Solid state degradation of **CEPU2** film using 1 M TBAF in acetone at room temperature over time.

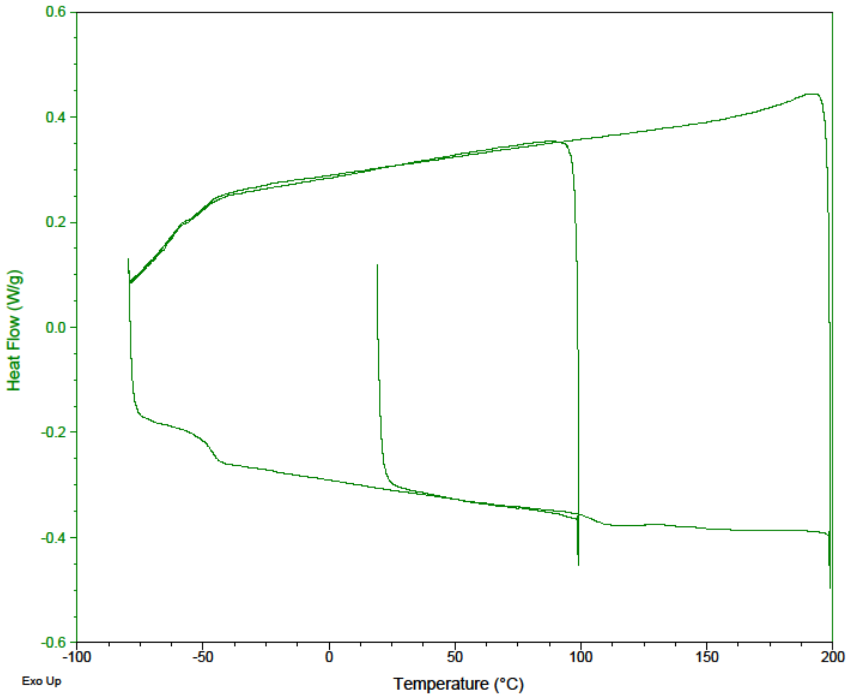

Figure S 109 DSC thermogram of **CEPU1** after 24 hours of solid state degradation with 40 wt.% $NaOH_{(aq)}$ at 50 °C showing the 1st and 2nd heating and cooling cycles at 10 °C min^{-1}.

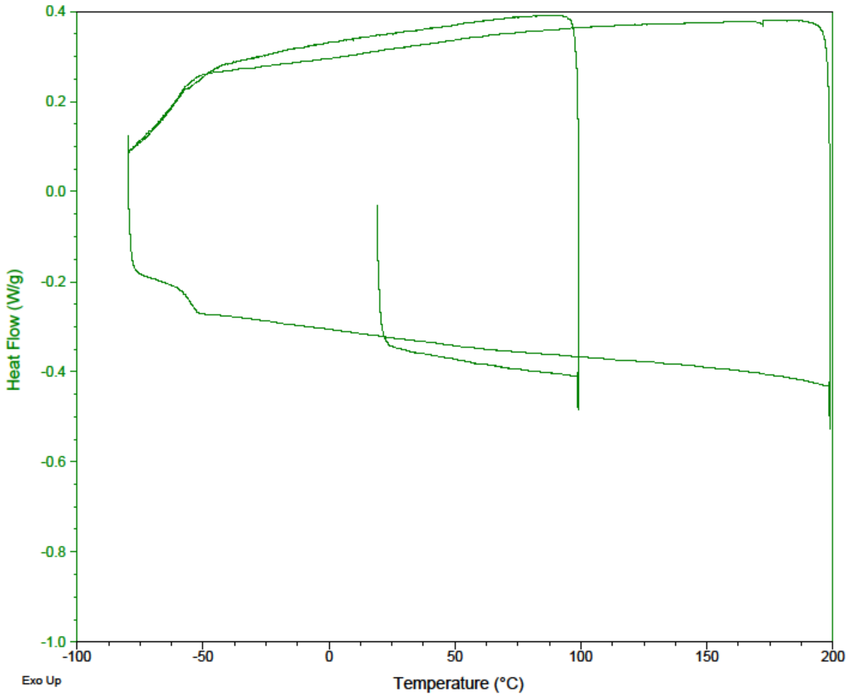

Figure S 110 DSC thermogram of **CEPU1** after 24 hours of solid state degradation with 1 M TBAF in acetone at room temperature showing the 1st and 2nd heating and cooling cycles at 10 °C min^{-1}.

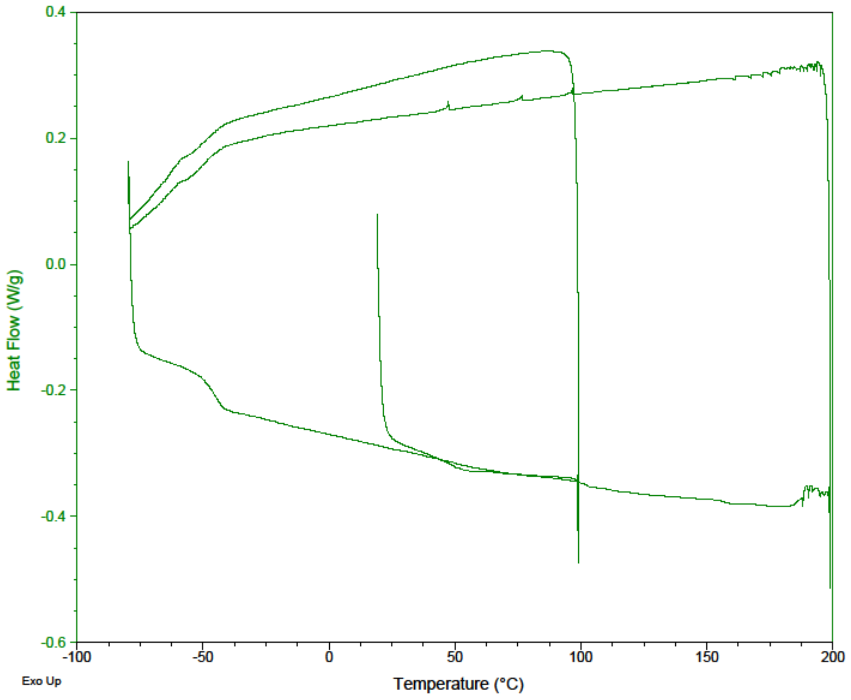

Figure S 111 DSC thermogram of **CEPU2** after 24 hours of solid state degradation with 40 wt.% $NaOH_{(aq)}$ at 50 °C showing the 1st and 2nd heating and cooling cycles at 10 °C min^{-1}.

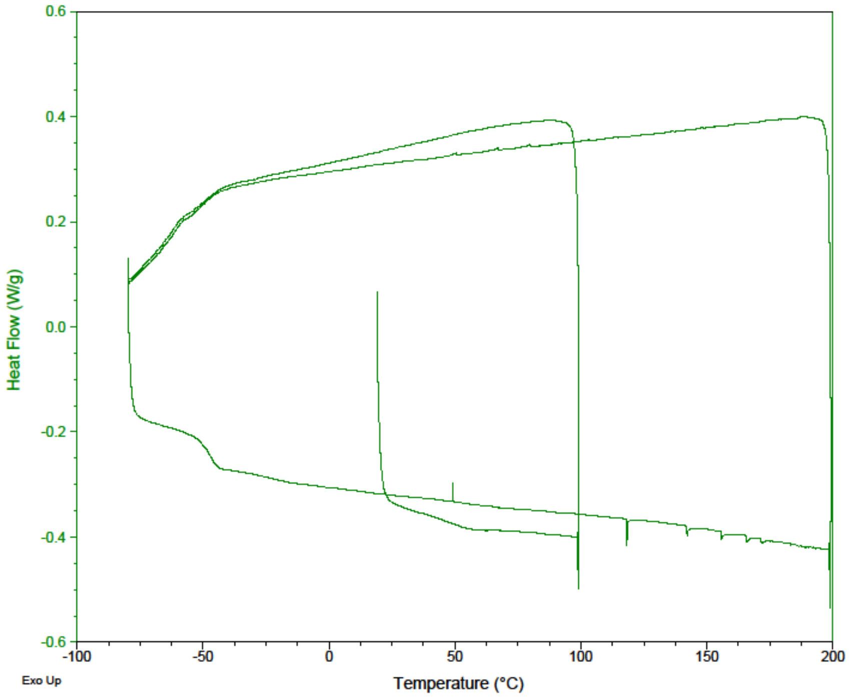

Figure S 112 DSC thermogram of **CEPU2** after 24 hours of solid state degradation with 1 M TBAF in acetone at room temperature showing the 1st and 2nd heating and cooling cycles at 10 °C min^{-1}.

Table S 22 Thermal properties of **CEPU1** and **CEPU2** post degradation and the thermal properties of **MeO-PU1** and **MeO-PU2**.

CEPU	Base	T_g (°C)[c]	T_m (°C)[a]	T_c (°C)[b]	T_m (°C)[c]	T_c (°C)[c]	T_c (°C)[d]
**CEPU1**	NaOH	-46.0	-	-	99.8	-	-
	TBAF	-47.1	-	-	-	-	-
**CEPU2**	NaOH	-45.7	-	-	-	-	-
	TBAF	-46.5	-	-	-	-	-
**MeO-PU1**	-	-45.6	41.2; 66.0	34.0	38.2; 75.1	-	49.1
**MeO-PU2**	-	-44.3	38.3	-	37.4	16.8	-

[a] First heating run 10 °C min^{-1}; [b] First cooling run 10 °C min^{-1}; [c] Second heating run 10 °C min^{-1}; [d] Second cooling run 10 °C min^{-1}.

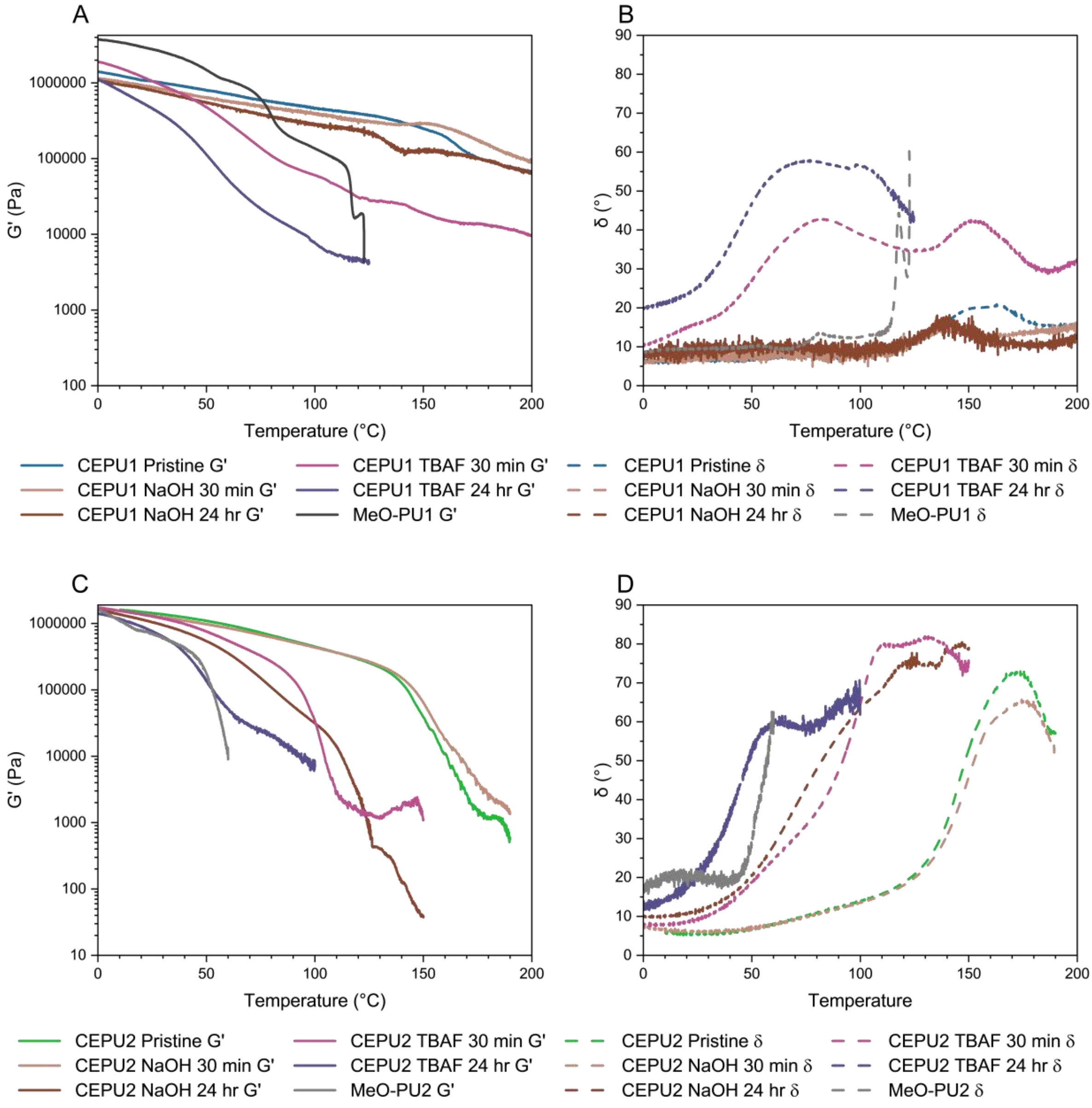

Figure S 113 Temperature sweep analysis of solid state degraded CEPUs using 40 wt.% $NaOH_{(aq)}$ or 1M TBAF in acetone over a temperature regime of 0 °C–200 °C, using a normal force of 1 N and a frequency of 1 Hz. **A CEPU1** G' *versus* temperature, **B CEPU1** phase shift (δ) *versus* temperature. **C CEPU2** G' *versus* temperature, **D CEPU2** phase shift (δ) *versus* temperature.

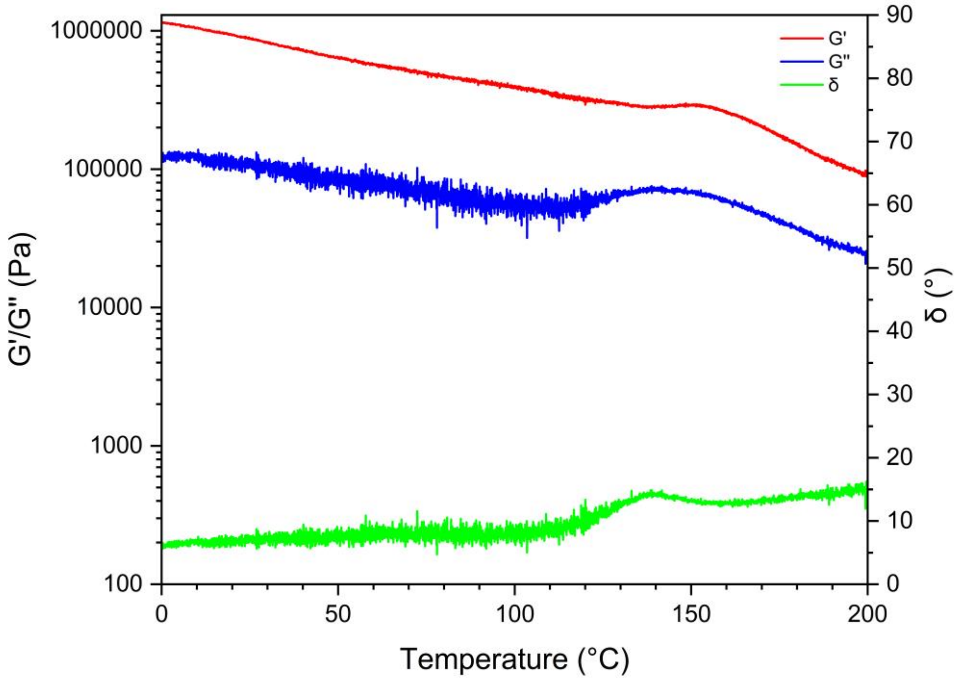

Figure S 114 Temperature sweep analysis of **CEPU1** after 30 minutes of solid state degradation with 40 wt.% $NaOH_{(aq)}$ at 50 °C over a temperature regime of 0 °C to 200 °C, using a normal force of 1 N and a frequency of 1 Hz. With G' (blue), G'' (red), and phase shift (δ) (green) against temperature.

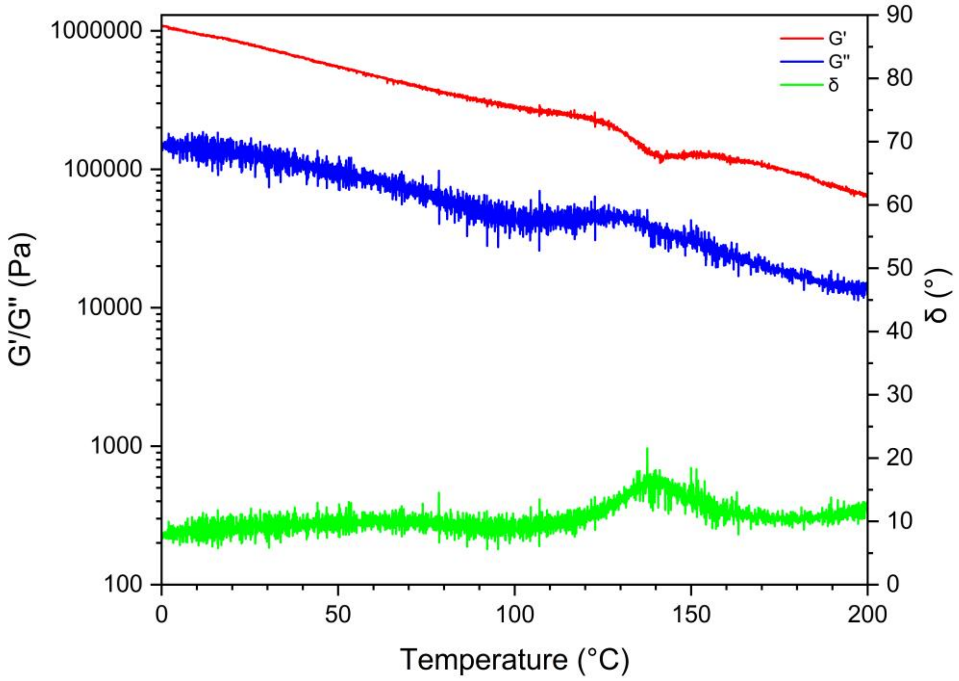

Figure S 115 Temperature sweep analysis of **CEPU1** after 24 hours of solid state degradation with 40 wt.% $NaOH_{(aq)}$ at 50 °C over a temperature regime of 0 °C to 200 °C, using a normal force of 1 N and a frequency of 1 Hz. With G' (blue), G'' (red), and phase shift (δ) (green) against temperature.

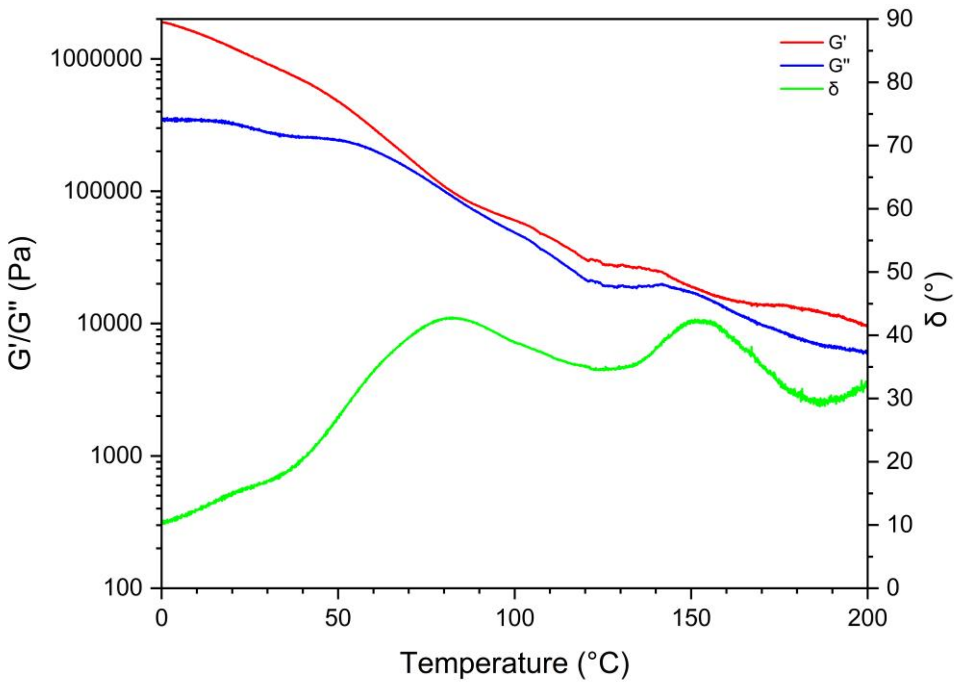

Figure S 116 Temperature sweep analysis of **CEPU1** after 30 minutes of solid state degradation with 1 M TBAF in acetone at room temperature over a temperature regime of 0 °C to 200 °C, using a normal force of 1 N and a frequency of 1 Hz. With G’ (blue), G’’ (red), and phase shift (δ) (green) against temperature.

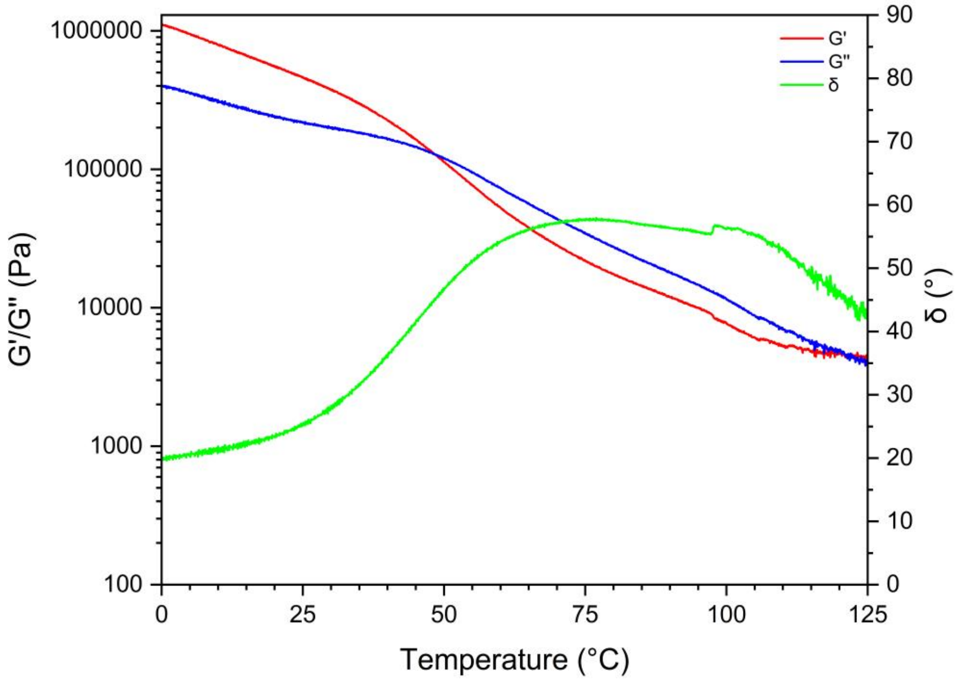

Figure S 117 Temperature sweep analysis of **CEPU1** after 24 hours of solid state degradation with 1 M TBAF in acetone at room temperature over a temperature regime of 0 °C to 125 °C, using a normal force of 1 N and a frequency of 1 Hz. With G’ (blue), G’’ (red), and phase shift (δ) (green) against temperature.

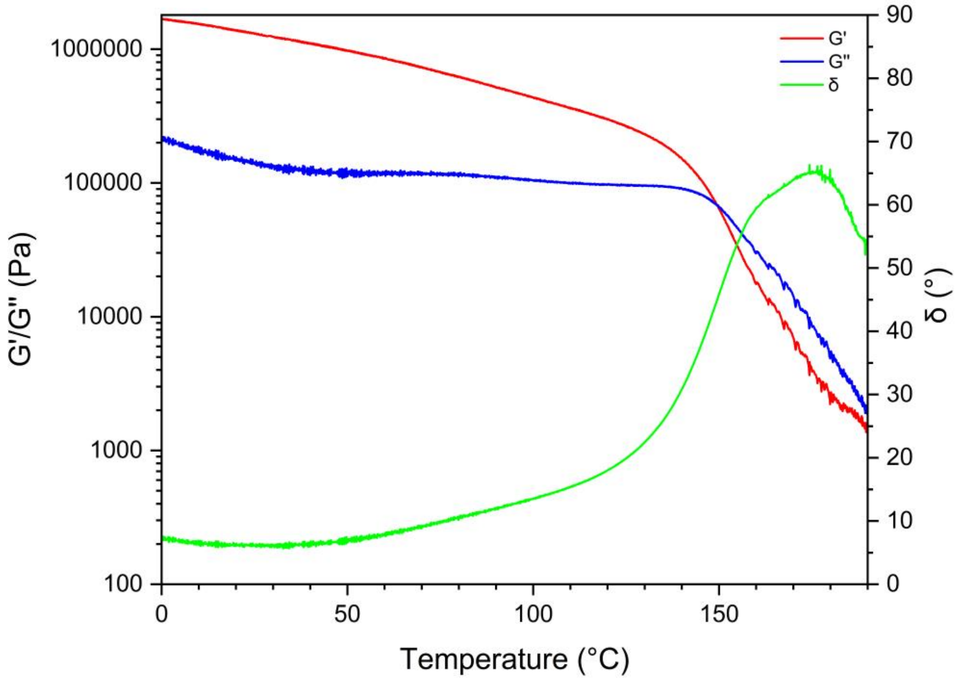

Figure S 118 Temperature sweep analysis of **CEPU2** after 30 minutes of solid state degradation with 40 wt.% $NaOH_{(aq)}$ at 50 °C over a temperature regime of 0 °C to 190 °C, using a normal force of 1 N and a frequency of 1 Hz. With G' (blue), G'' (red), and phase shift (δ) (green) against temperature.

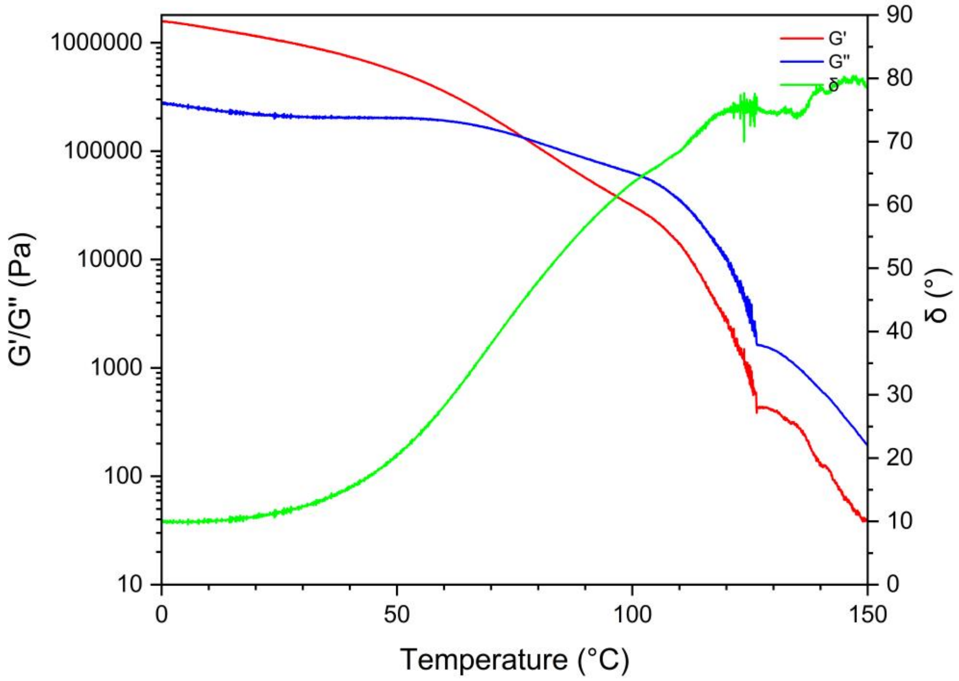

Figure S 119 Temperature sweep analysis of **CEPU2** after 24 hours of solid state degradation with 40 wt.% $NaOH_{(aq)}$ at 50 °C over a temperature regime of 0 °C to 150 °C, using a normal force of 1 N and a frequency of 1 Hz. With G' (blue), G'' (red), and phase shift (δ) (green) against temperature.

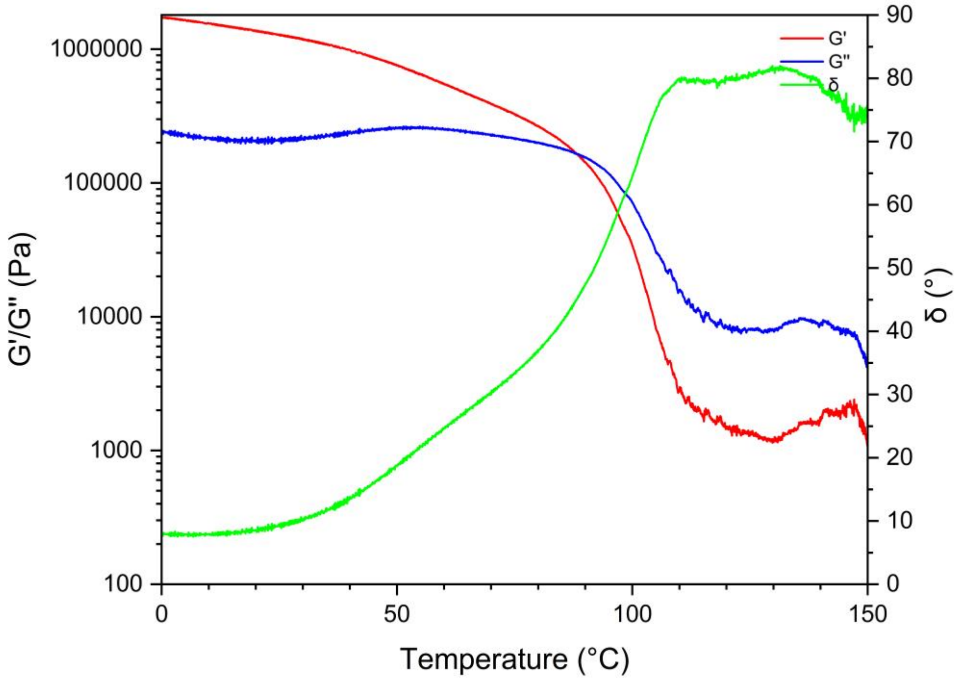

Figure S 120 Temperature sweep analysis of **CEPU2** after 30 minutes of solid state degradation with 1 M TBAF in acetone at room temperature over a temperature regime of 0 °C to 150 °C, using a normal force of 1 N and a frequency of 1 Hz. With G’ (blue), G’’ (red), and phase shift (δ) (green) against temperature.

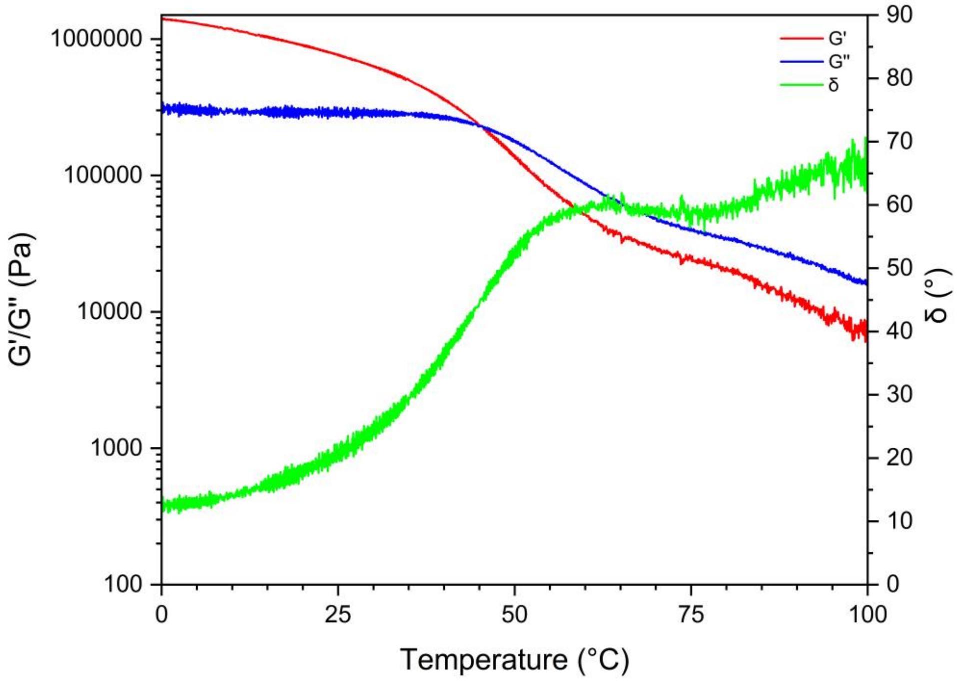

Figure S 121 Temperature sweep analysis of **CEPU2** after 24 hours of solid state degradation with 1 M TBAF in acetone at room temperature over a temperature regime of 0 °C to 100 °C, using a normal force of 1 N and a frequency of 1 Hz. With G’ (blue), G’’ (red), and phase shift (δ) (green) against temperature.

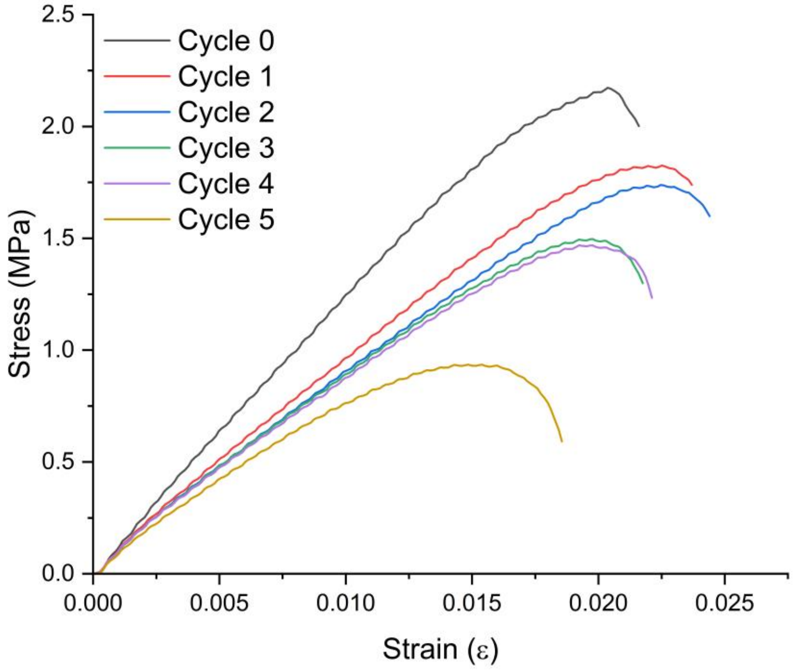

Figure S 122 Representative stress-strain curves of the adhered **CEPU1** to aluminium over five re-adhesion cycles.

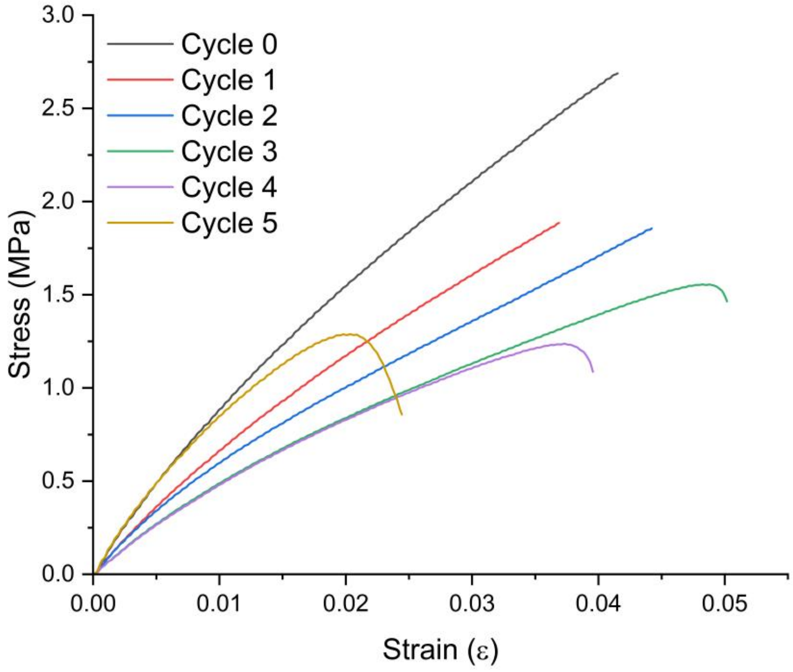

Figure S 123 Representative stress-strain curves of the adhered **CEPU2** to aluminium over five re-adhesion cycles.

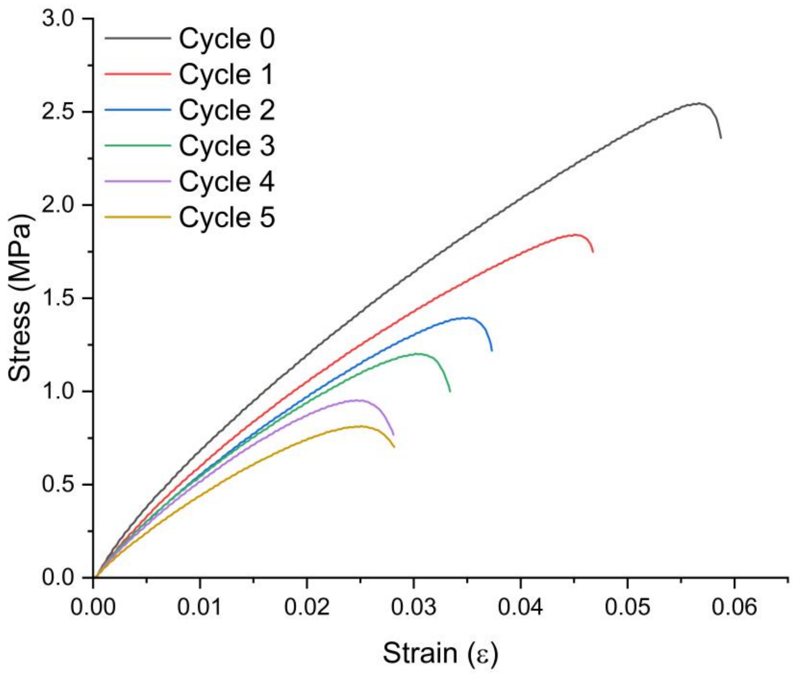

Figure S 124 Representative stress-strain curves of the adhered **CEPU3** to aluminium over five re-adhesion cycles.

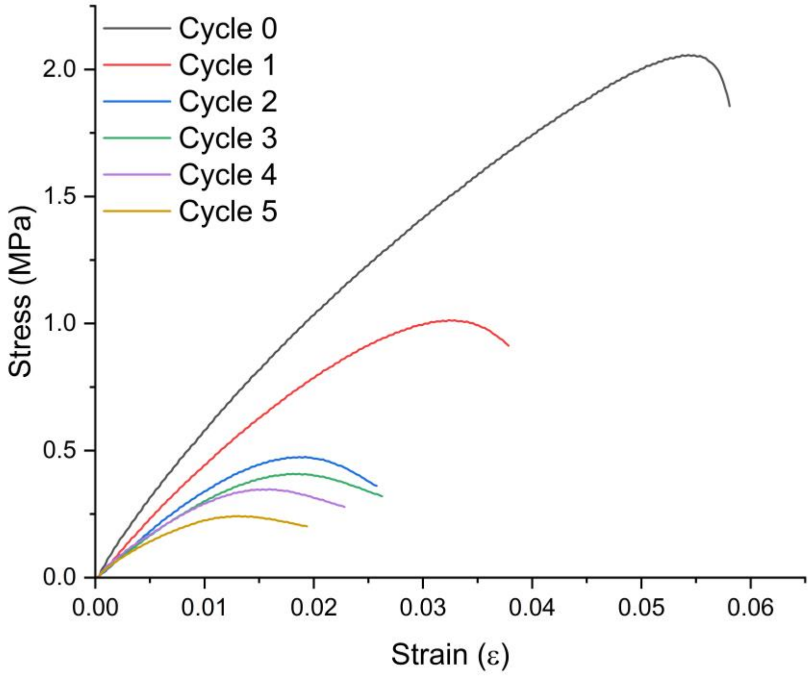

Figure S 125 Representative stress-strain curves of the adhered **CEPU4** to aluminium over five re-adhesion cycles.

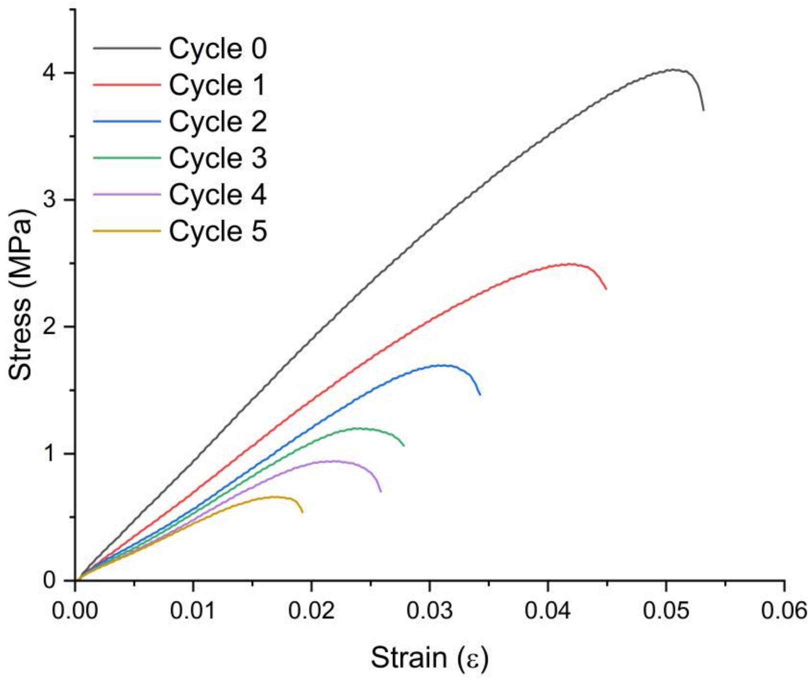

Figure S 126 Representative stress-strain curves of the adhered **CEPU5** to aluminium over five re-adhesion cycles.

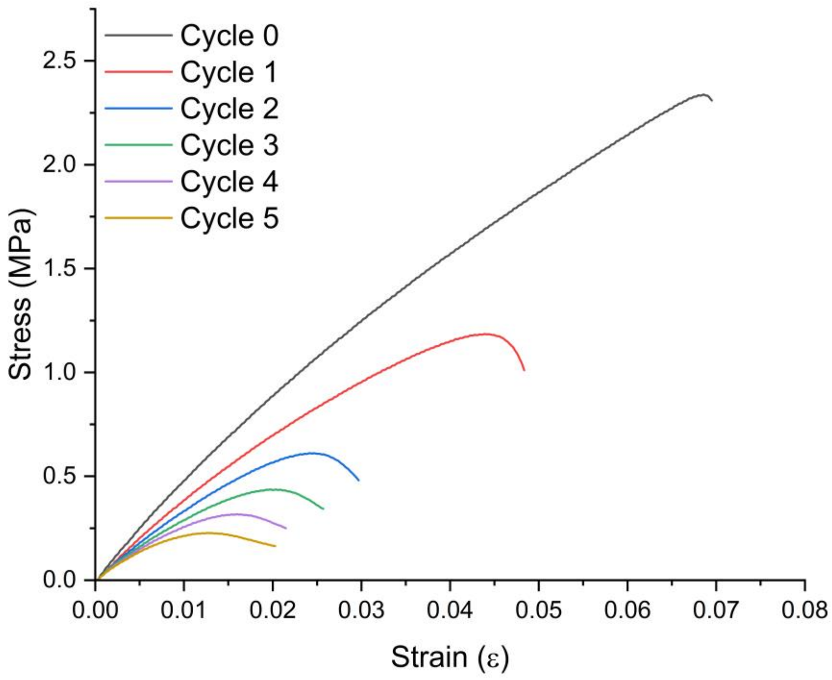

Figure S 127 Representative stress-strain curves of the adhered **CEPU6** to aluminium over five re-adhesion cycles.

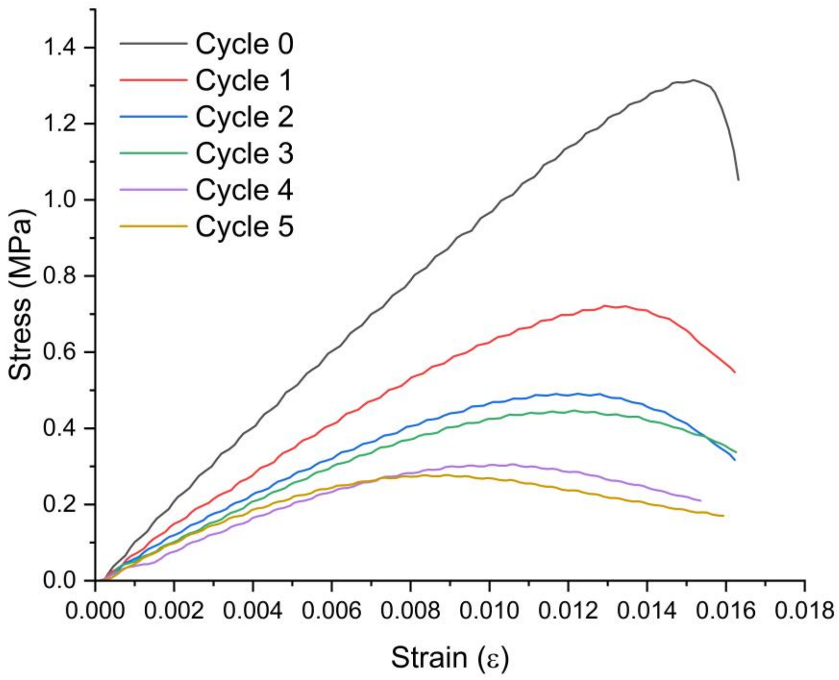

Figure S 128 Representative stress-strain curves of the adhered **CEPU1** to glass over five re-adhesion cycles.

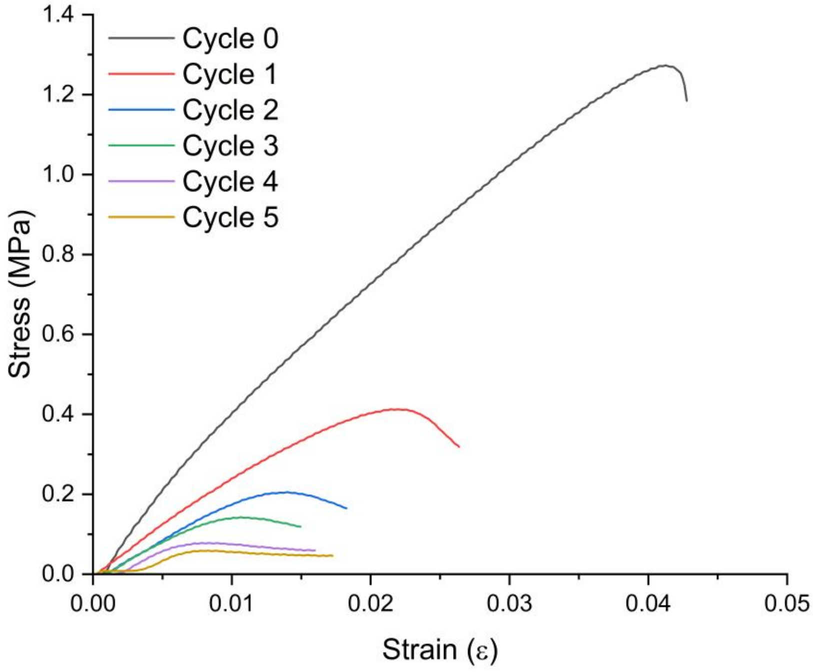

Figure S 129 Representative stress-strain curves of the adhered **CEPU2** to glass over five re-adhesion cycles.

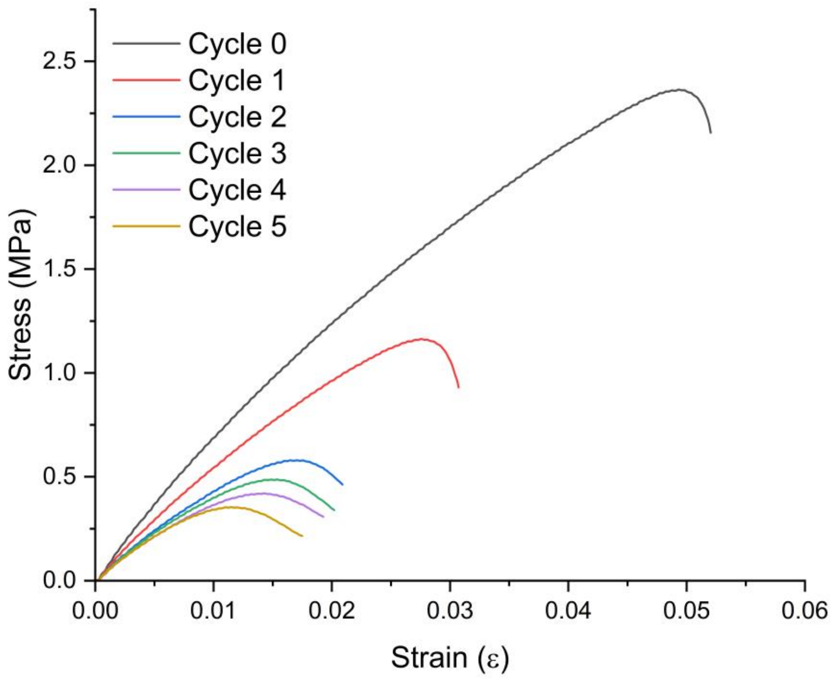

Figure S 130 Representative stress-strain curves of the adhered **CEPU3** to glass over five re-adhesion cycles.

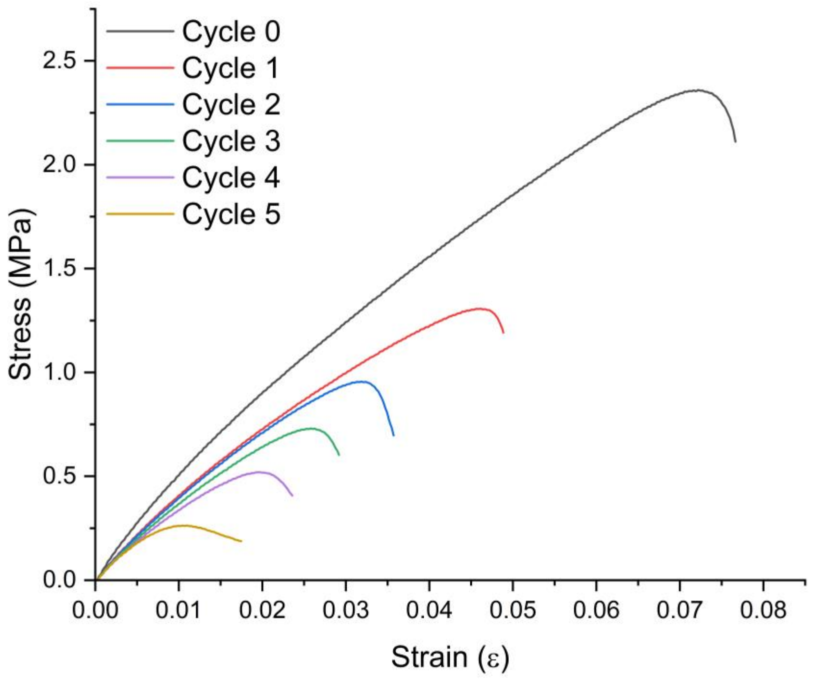

Figure S 131 Representative stress-strain curves of the adhered **CEPU4** to glass over five re-adhesion cycles.

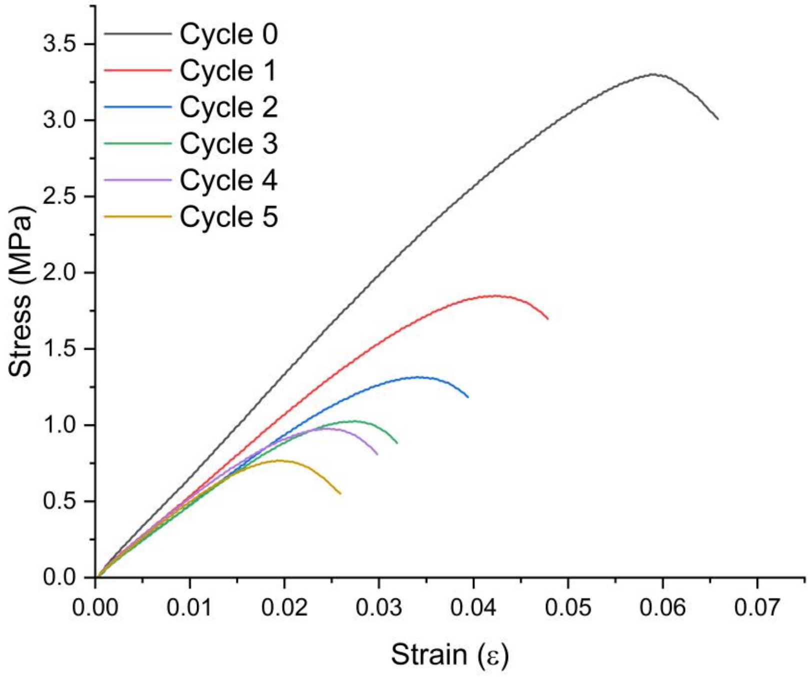

Figure S 132 Representative stress-strain curves of the adhered **CEPU5** to glass over five re-adhesion cycles.

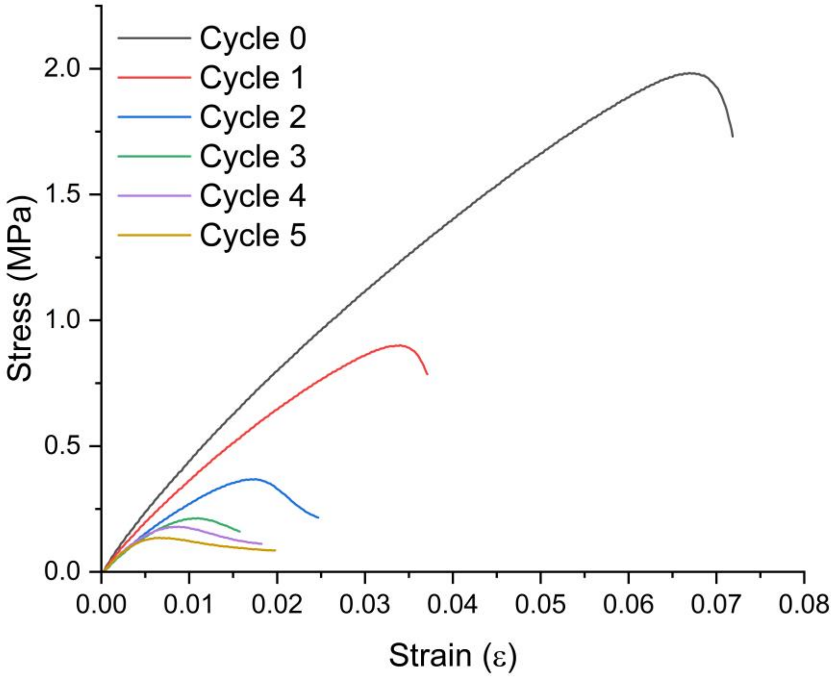

Figure S 133 Representative stress-strain curves of the adhered **CEPU6** to glass over five re-adhesion cycles.

Table S 23 Shear strength of **CEPU1-CEPU6** over five re-adhesion cycles on Aluminium. The error shown is the standard deviation between the three repeats of each sample.

CEPU adhesive	Shear Strength (MPa)					
	Cycle 0	Cycle 1	Cycle 2	Cycle 3	Cycle 4	Cycle 5
CEPU1	2.18 ± 0.4	1.84 ± 0.3	1.48 ± 0.2	1.21 ± 0.2	1.15 ± 0.2	0.85 ± 0.1
CEPU2	2.78 ± 0.1	1.85 ± 0.0	1.83 ± 0.0	1.67 ± 0.1	1.35 ± 0.1	1.00 ± 0.2
CEPU3	2.60 ± 0.	1.83 ± 0.1	1.48 ± 0.2	1.30 ± 0.2	1.15 ± 0.2	0.99 ± 0.2
CEPU4	2.24 ± 0.2	1.08 ± 0.1	0.58 ± 0.1	0.40 ± 0.1	0.32 ± 0.1	0.25 ± 0.0
CEPU5	3.82 ± 0.2	2.51 ± 0.1	1.63 ± 0.0	1.13 ± 0.0	0.92 ± 0.0	0.74 ± 0.0
CEPU6	2.20 ± 0.1	1.01 ± 0.1	0.55 ± 0.1	0.39 ± 0.1	0.29 ± 0.0	0.24 ± 0.0

Table S 24 Shear strength of **CEPU1**-**CEPU6** over five re-adhesion cycles on Glass. The error shown is the standard deviation between the three repeats of each sample.

CEPU adhesive	Shear Strength (MPa)					
	Cycle 0	Cycle 1	Cycle 2	Cycle 3	Cycle 4	Cycle 5
CEPU1	1.13 ± 0.1	0.72 ± 0.1	0.49 ± 0.1	0.38 ± 0.1	0.28 ± 0.0	0.26 ± 0.1
CEPU2	1.15 ± 0.0	0.41 ± 0.0	0.19 ± 0.0	0.13 ± 0.0	0.07 ± 0.0	0.05 ± 0.0
CEPU3	2.35 ± 0.1	1.00 ± 0.1	0.61 ± 0.1	0.49 ± 0.0	0.41 ± 0.0	0.37 ± 0.0
CEPU4	2.27 ± 0.1	1.59 ± 0.2	1.00 ± 0.0	0.69 ± 0.0	0.51 ± 0.1	0.33 ± 0.1
CEPU5	3.35 ± 0.1	1.90 ± 0.1	1.38 ± 0.1	1.01 ± 0.1	0.95 ± 0.0	0.72 ± 0.0
CEPU6	1.71 ± 0.2	0.65 ± 0.1	0.30 ± 0.0	0.21 ± 0.0	0.19 ± 0.0	0.17 ± 0.0

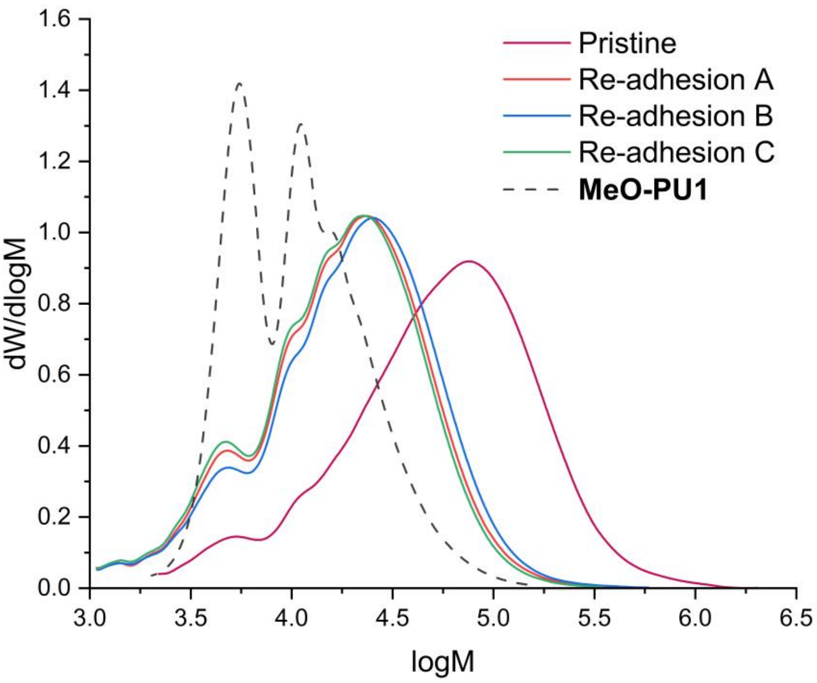

Figure S 134 GPC eluogram of **CEPU3** in THF after five re-adhesion cycles to glass.

Table S 25 GPC molecular weight and dispersity data for **CEPU3** after five re-adhesion cycles to glass; the order of the data in the table is as follows: pristine **CEPU3**; **CEPU3** after five re-adhesion cycles; % loss. The error shown is the standard deviation between the three repeats of each sample. The percentage error shown is the standard error between the pristine and re-adhered averages for each sample.

M_n (g mol^{-1})	M_w (g mol^{-1})	Đ
30900 ± 300	181200 ± 1500	5.86
10900 ± 300	27000 ± 1100	2.48
65 ± 2	85 ± 4	

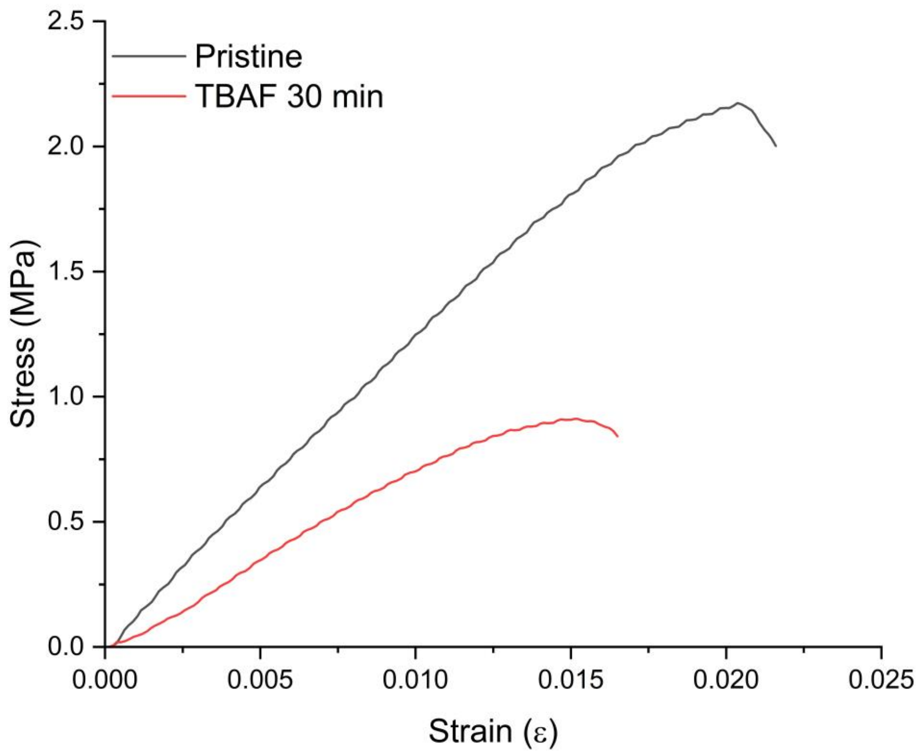

Figure S 135 Representative stress-strain curve of the adhered **CEPU1** to aluminium after 30 minutes of solid-state degradation with 1 M TBAF in acetone at room temperature.

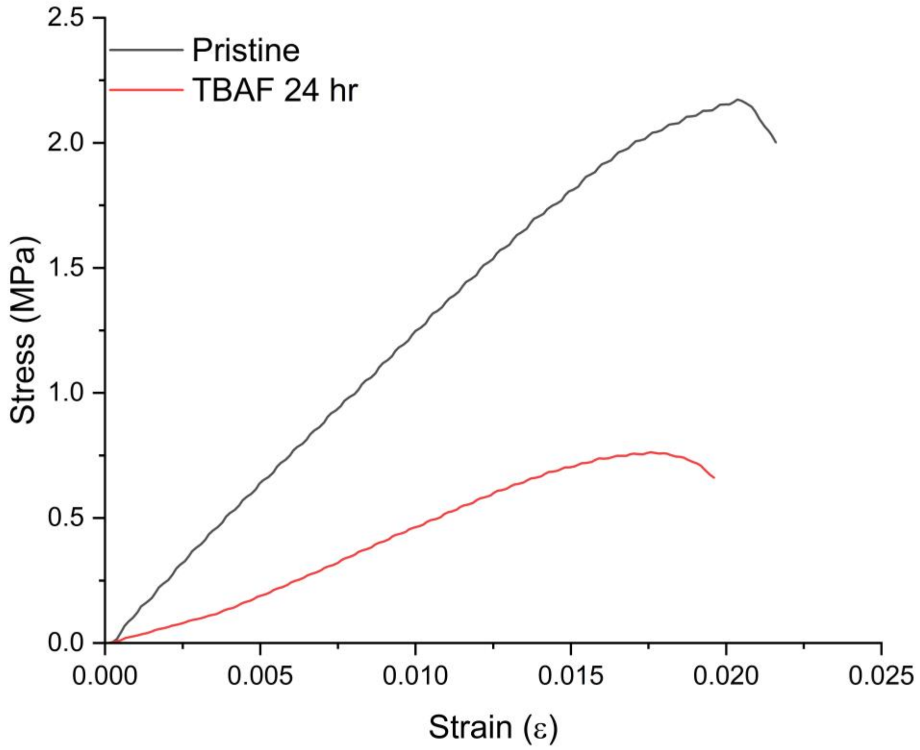

Figure S 136 Representative stress-strain curve of the adhered **CEPU1** to aluminium after 24 hours of solid-state degradation with 1 M TBAF in acetone at room temperature.

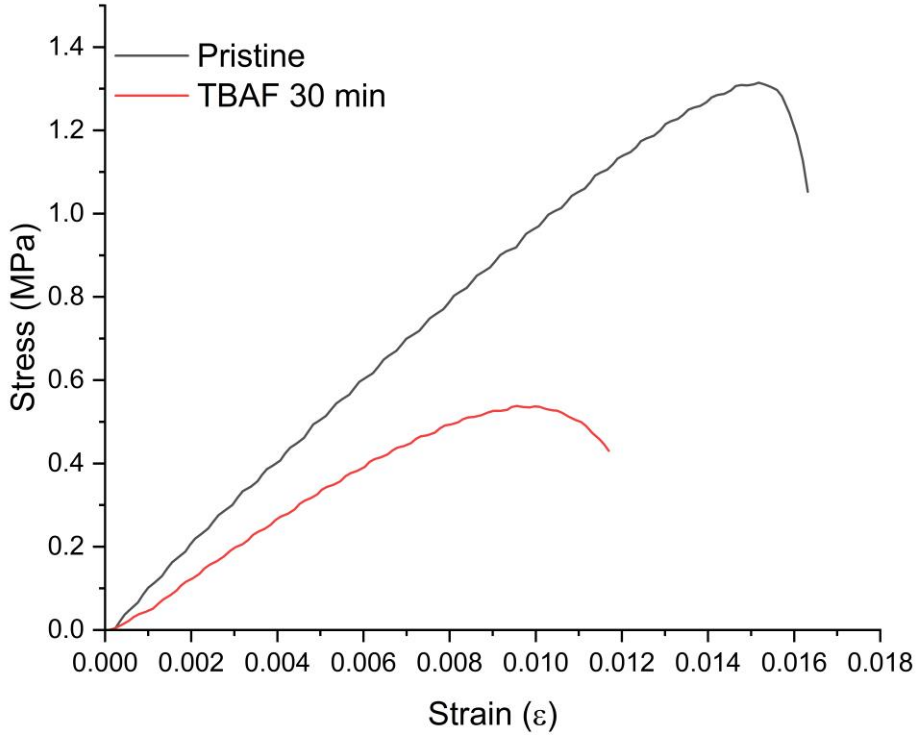

Figure S 137 Representative stress-strain curve of the adhered **CEPU1** to glass after 30 minutes of solid-state degradation with 1 M TBAF in acetone at room temperature.

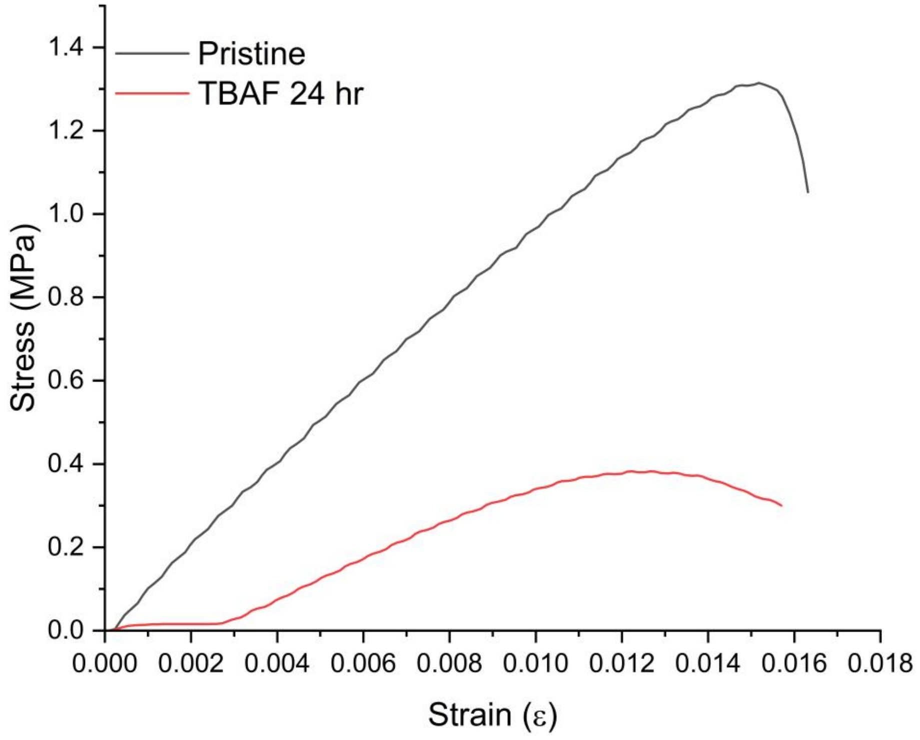

Figure S 138 Representative stress-strain curve of the adhered **CEPU1** to glass after 24 hours of solid-state degradation with 1 M TBAF in acetone at room temperature.

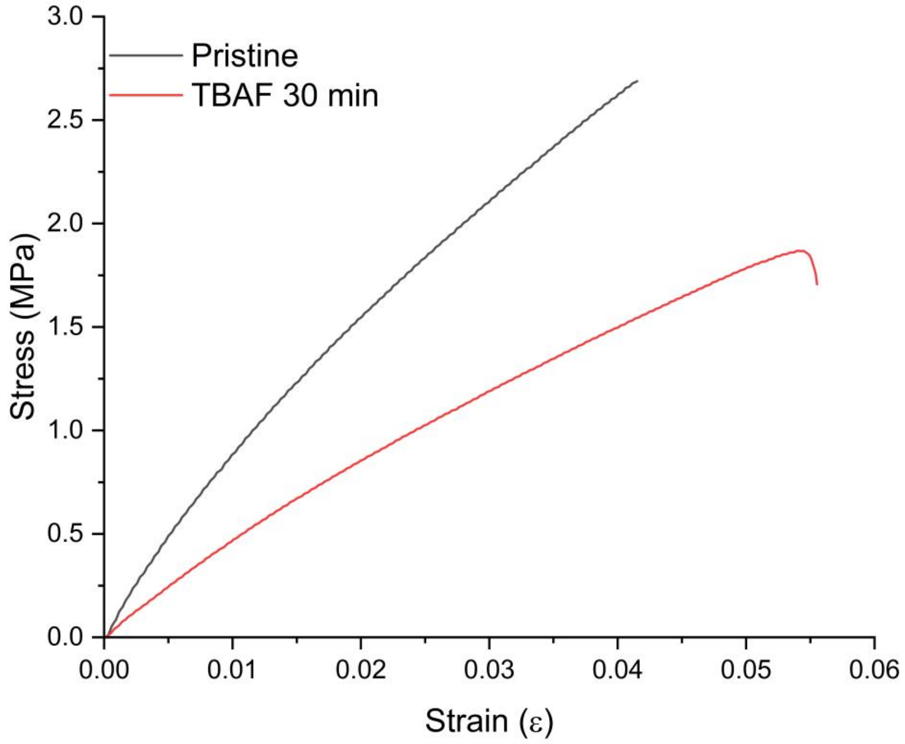

Figure S 139 Representative stress-strain curve of the adhered **CEPU2** to aluminium after 30 minutes of solid-state degradation with 1 M TBAF in acetone at room temperature.

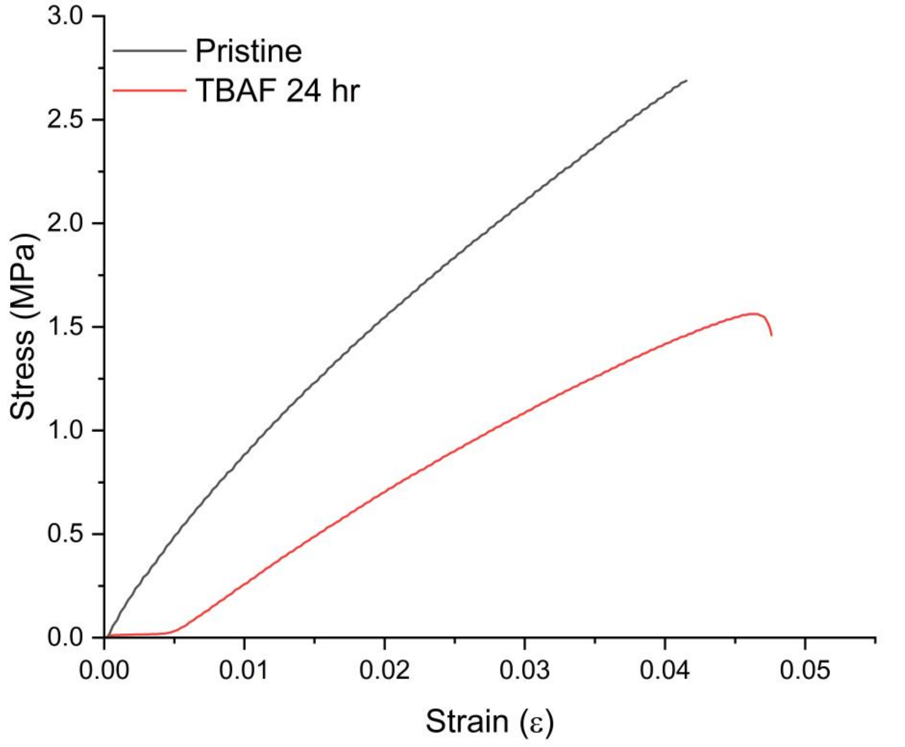

Figure S 140 Representative stress-strain curve of the adhered **CEPU2** to aluminium after 24 hours of solid-state degradation with 1 M TBAF in acetone at room temperature.

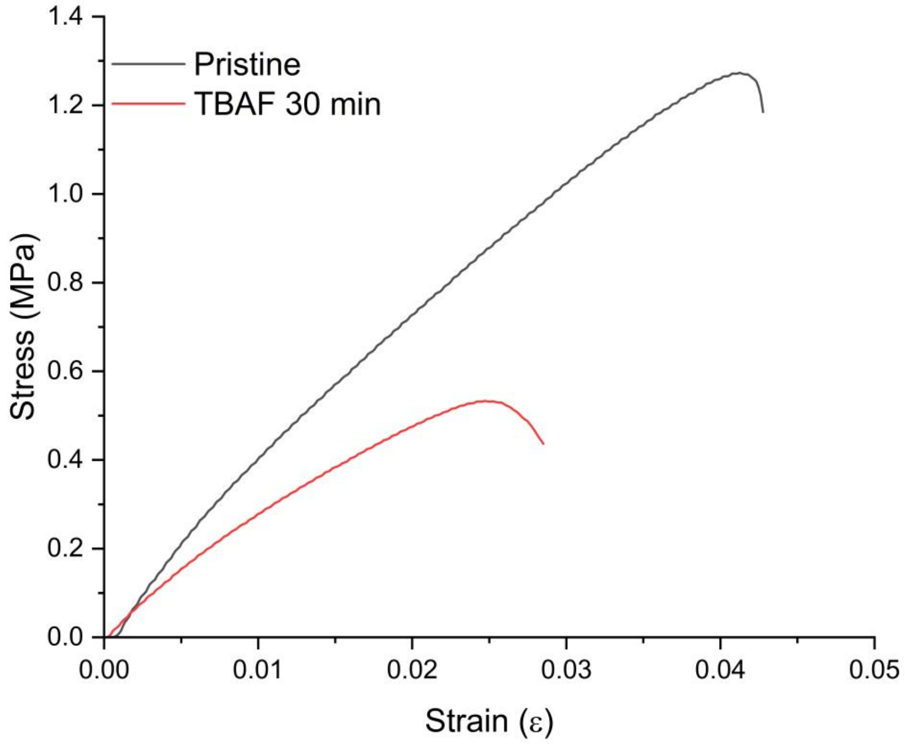

Figure S 141 Representative stress-strain curve of the adhered **CEPU2** to glass after 30 minutes of solid-state degradation with 1 M TBAF in acetone at room temperature.

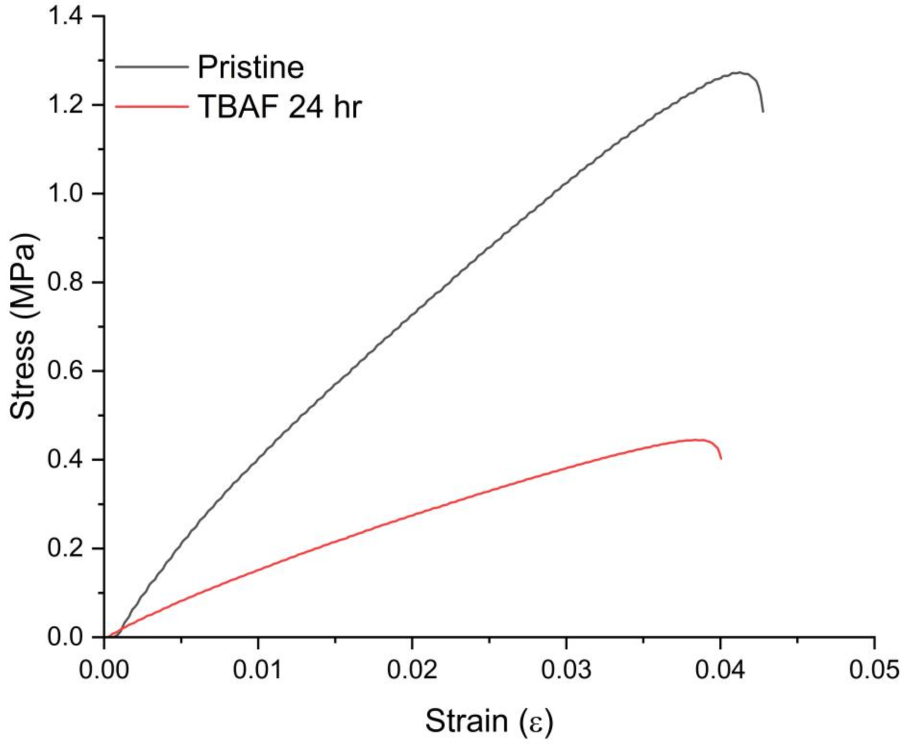

Figure S 142 Representative stress-strain curve of the adhered **CEPU2** to glass after 24 hours of solid-state degradation with 1 M TBAF in acetone at room temperature.

Table S 26 Shear strength of **CEPU1** and **CEPU2** on aluminium and glass after exposure to 1 M TBAF in acetone for 30 minutes and 24 hours; the order of the data in the table for each entry is as follows: shear strength; % loss in shear strength. The error shown is the standard deviation between the three repeats of each sample. The percentage error shown is the standard error between the pristine and degraded averages for each sample.

CEPU adhesive	Substrate	Pristine	30 min TBAF	24 hr TBAF
**CEPU1**	Aluminium	2.18 ± 0.4 MPa<br>-	0.87 ± 0.0 MPa<br>60 ± 12%	0.76 ± 0.0 MPa<br>65 ± 14%
	Glass	1.13 ± 0.1 MPa<br>-	0.52 ± 0.0 MPa<br>54 ± 6%	0.39 ± 0.0 MPa<br>65 ± 7%
**CEPU2**	Aluminium	2.78 ± 0.1 MPa<br>-	1.77 ± 0.2 MPa<br>36 ± 3%	1.47 ± 0.1 MPa<br>47 ± 3%
	Glass	1.15 ± 0.0 MPa<br>-	0.55 ± 0.0 MPa<br>52 ± 2%	0.43 ± 0.0 MPa<br>63 ± 6%

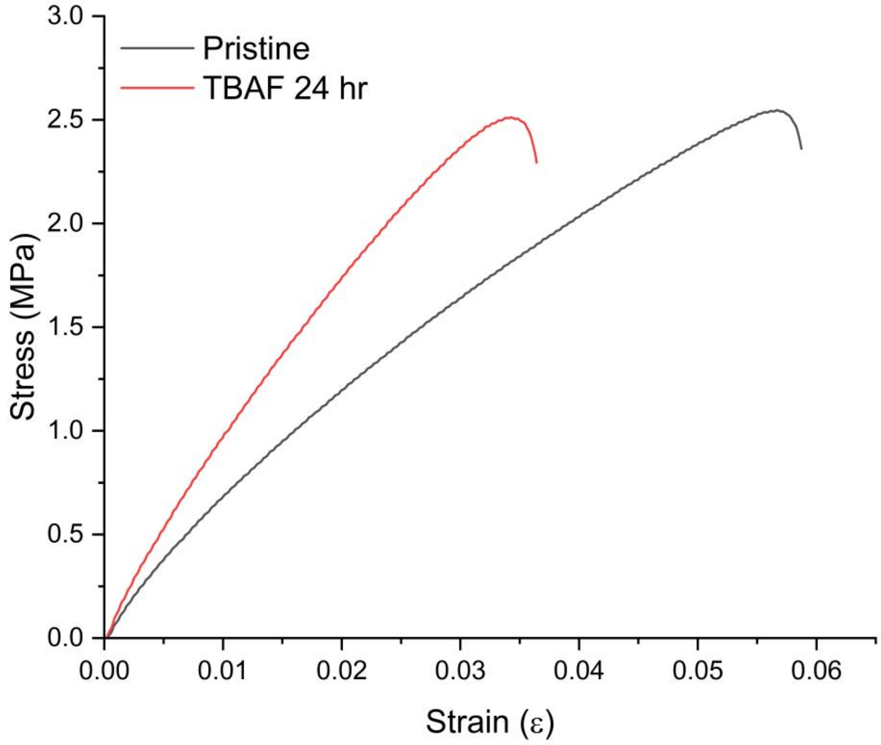

Figure S 143 Representative stress-strain curve of the adhered **CEPU3** to aluminium after 24 hours of solid-state degradation with 1 M TBAF in acetone at room temperature.

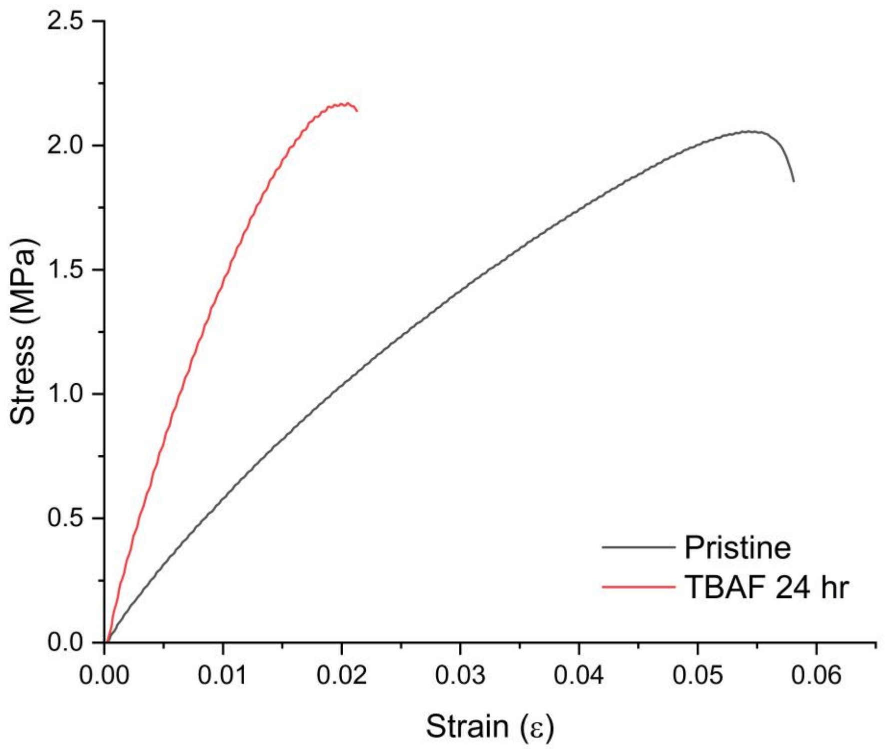

Figure S 144 Representative stress-strain curve of the adhered **CEPU4** to aluminium after 24 hours of solid-state degradation with 1 M TBAF in acetone at room temperature.

Table S 27 Shear strength of **CEPU3** and **CEPU4** on aluminium after exposure to 1 M TBAF in acetone for 24 hours; the order of the data in the table for each entry is as follows: shear strength; % loss in shear strength. The error shown is the standard deviation between the three repeats of each sample. The percentage error shown is the standard error between the pristine and degraded averages for each sample.

CEPU adhesive	Substrate	Pristine	24 hr TBAF
**CEPU3**	Aluminium	2.60 ± 0.1 MPa	2.53 ± 0.2 MPa
		-	3 ± 1%
**CEPU4**	Aluminium	2.24 ± 0.2 MPa	2.21 ± 0.1 MPa
		-	1 ± 0%

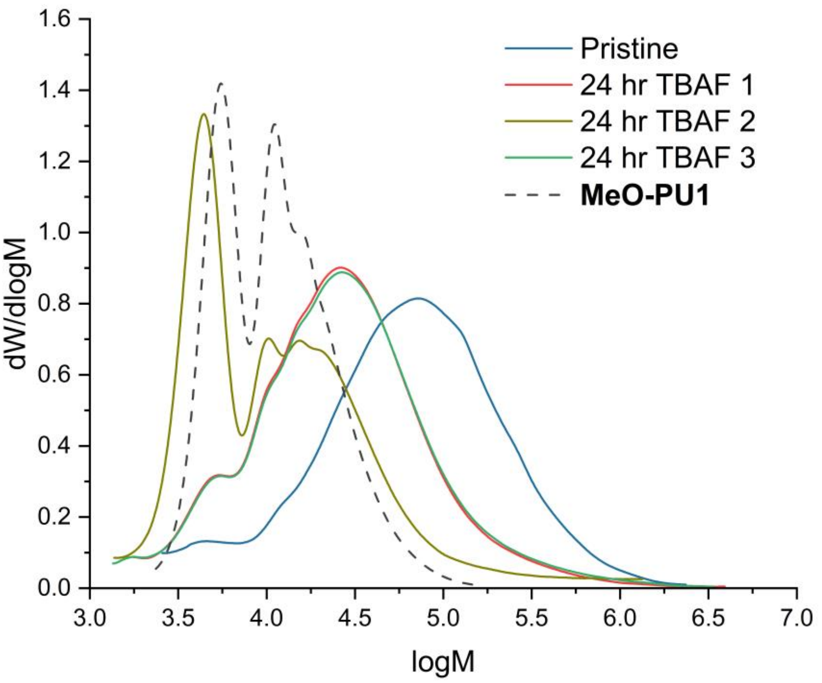

Figure S 145 GPC eluograms of **CEPU1** in THF after 24 hours of solid-state degradation with 1 M TBAF in acetone at room temperature when adhered to glass.

Table S 28 GPC molecular weight and dispersity data for adhered **CEPU1** to glass after 24 hours of solid-state degradation with 1 M TBAF in acetone at room temperature; the order of the data in the table is as follows: pristine **CEPU1**; **CEPU1** after 24 hour solid-state degradation; % loss. The error shown is the standard deviation between the three repeats of each sample. The percentage error shown is the standard error between the pristine and 24 hour solid-state degradation averages for each sample.

M_n (g mol^{-1})	M_w (g mol^{-1})	Đ
30100 ± 300	124900 ± 0	4.15
11400 ± 1800	50400 ± 7500	4.41
62 ± 10	60 ± 9	
